# Integrated Analysis of Mutation Data from Various Sources Identifies Key Genes and Signaling Pathways in Hepatocellular Carcinoma

**DOI:** 10.1371/journal.pone.0100854

**Published:** 2014-07-02

**Authors:** Yuannv Zhang, Zhaoping Qiu, Lin Wei, Ruqi Tang, Baofeng Lian, Yingjun Zhao, Xianghuo He, Lu Xie

**Affiliations:** 1 State Key Laboratory of Oncogenes and Related Genes, Shanghai Cancer Institute, Renji Hospital, Shanghai Jiao Tong University School of Medicine, Shanghai, China; 2 Shanghai Center for Bioinformation Technology, Shanghai Academy of Science and Technology, Shanghai, China; Chinese University of Hong Kong, China

## Abstract

**Background:**

Recently, a number of studies have performed genome or exome sequencing of hepatocellular carcinoma (HCC) and identified hundreds or even thousands of mutations in protein-coding genes. However, these studies have only focused on a limited number of candidate genes, and many important mutation resources remain to be explored.

**Principal Findings:**

In this study, we integrated mutation data obtained from various sources and performed pathway and network analysis. We identified 113 pathways that were significantly mutated in HCC samples and found that the mutated genes included in these pathways contained high percentages of known cancer genes, and damaging genes and also demonstrated high conservation scores, indicating their important roles in liver tumorigenesis. Five classes of pathways that were mutated most frequently included (a) proliferation and apoptosis related pathways, (b) tumor microenvironment related pathways, (c) neural signaling related pathways, (d) metabolic related pathways, and (e) circadian related pathways. Network analysis further revealed that the mutated genes with the highest betweenness coefficients, such as the well-known cancer genes TP53, CTNNB1 and recently identified novel mutated genes GNAL and the ADCY family, may play key roles in these significantly mutated pathways. Finally, we highlight several key genes (e.g., RPS6KA3 and PCLO) and pathways (e.g., axon guidance) in which the mutations were associated with clinical features.

**Conclusions:**

Our workflow illustrates the increased statistical power of integrating multiple studies of the same subject, which can provide biological insights that would otherwise be masked under individual sample sets. This type of bioinformatics approach is consistent with the necessity of making the best use of the ever increasing data provided in valuable databases, such as TCGA, to enhance the speed of deciphering human cancers.

## Introduction

Hepatocellular carcinoma (HCC) is the third most frequent cause of cancer-related mortality worldwide and is usually associated with specific risk factors, including hepatitis B or C infection, high alcohol intake, hemochromatosis or nonalcoholic fatty liver disease caused by obesity and insulin resistance [1]. Despite its global importance, our knowledge of the genomic alterations implicated in HCC initiation and progression is still fragmentary and the key drivers of tumorigenesis remain poorly understood, which limit the development of targeted therapy for HCC [2], [3]. To complete the mutation landscape of HCC, a number of studies have recently performed genome or exome sequencing of HCC and identified hundreds or even thousands of mutations in protein-coding genes [1], [4], [5], [6], [7]. These studies have confirmed some important alterations (e.g., mutations in CTNNB1, AXIN1, TP53, CDKN2A, etc.) and more importantly, revealed novel alterations (e.g., mutations in ARID2, ARID1A, NRF2, etc.) that have refined our knowledge of the mutational landscape and the related signaling pathways involved in liver carcinogenesis.

However, although these studies demonstrated the high complexity and heterogeneity of HCC genomes, each study has only focused on a limited number of candidate mutated genes; thus, a large amount of important mutation resources remain to be explored. In addition, the number of HCC samples of each study is limited. Thus, integrating these data sources may increase the statistical power to depict the HCC mutation landscape and to pinpoint novel dominant cancer genes and signaling pathways in HCC pathogenesis. It is becoming clear that individual tumors of the same histopathological subtype may show distinct genetic alterations, but the affected pathways in different tumors are similar [8]. This phenomenon can also be described as “universality in module, diversity on the genetic level” [9]. Thus, elucidating genetic alterations at the pathway level may reveal common features of different individuals. Thus, with the emergence of abundant mutation data sources of HCC, it is rational to systemically evaluate these mutated genes and the related signaling pathways involved in liver carcinogenesis.

In this study, we first compared four sets of mutated genes from different sources, and found that although only a minority (32%–37%) of mutated genes from the lesser samples (∼10) reoccur in the larger samples (∼90), a majority (67%–100%) of the significantly mutated pathways from the lesser samples reoccurred in the larger samples. Next, we integrated these mutated genes and identified 113 significantly mutated pathways. Several lines of evidence indicate that the mutated genes included in these significantly mutated pathways were more likely to be cancer genes. Network analysis further revealed that the mutated genes with the highest betweenness coefficients may play a key role in these signaling pathways. Finally, we evaluated the clinical significance of key genes and pathways.

## Results

### Comparing four sets of mutated genes from different sources

We collected four sets of mutation data from the International Cancer Genome Consortium (ICGC) [10], Kan et al. [11], Li et al. [6] and Huang et al. [5], which surveyed 99, 88, 10 and 10 HCC samples, respectively. Data obtained from the ICGC and Kan et al. with larger samples detected 4376 and 3702 mutated genes, respectively, whereas data obtained from Li et al. and Huang et al. contained smaller samples that detected 398 and 347 mutated genes, respectively. By comparing the four sets of mutated genes, we found that approximately 35% of the mutated genes identified from the smaller samples were also detected in the larger samples, while only 3% of the mutated genes identified from the larger samples were also detected in the smaller samples (Table 1). This result indicated the diversity of mutated genes among distinct tumor samples and also revealed that larger samples provide better coverage for the detection of potentially mutated genes in a tumor type.

**Table 1:** Overlap of four sets of mutated genes.

	ICGC	Kan et al.	Li et al.	Huang et al.
ICGC	4376	0.32	**0.03**	**0.03**
Kan et al.	0.38	3702	**0.04**	**0.03**
Li et al.	**0.37**	**0.35**	398	0.05
Huang et al.	**0.33**	**0.32**	0.05	347

Note: The diagonal is the number of mutated genes from four sources. The percentages above (or below) the diagonal represent the number of overlapping genes divided by the number of the longer (or shorter) set of mutated genes. The values in bold font are the comparison results between the larger and smaller sample sizes.

To compare the biological pathways of these four sets of mutated genes, we analyzed these mutated genes using two popular methods—the pathway coverage method [1], [12], [13] and the hypergeometric distribution model [14] (see Materials and Methods for more information). The results obtained using the pathway coverage method showed that with a 5% false discovery rate (FDR) control, 99 and 75 significantly mutated pathways were identified from data obtained from the ICGC and Kan et al., while only 36 and 3 significantly mutated pathways were identified from data obtained from Li et al. and Huang et al., respectively. By comparing these four sets of pathways, we found that 67%–100% of significantly mutated pathways identified from the smaller samples were also detected in the larger samples, while only 3%–41% of significantly mutated pathways identified from the larger samples were also detected in the smaller samples (Table 2). Similar results were obtained using the hypergeometric distribution model (Table S1). These results demonstrated that most of the significantly mutated pathways from the smaller samples reoccurred in the larger samples, indicating that common features can be characterized across different tumors at the pathway level despite the high heterogeneity at the gene level. Given that the results drawn from larger samples may reflect the overall trend, we integrated four sets of mutated genes in the following analyses.

**Table 2:** Overlap of four sets of significant pathways obtained using the pathway coverage method.

	ICGC	Kan et al.	Li et al.	Huang et al.
ICGC	99	0.71	**0.31**	**0.03**
Kan et al.	0.93	75	**0.41**	**0.03**
Li et al.	**0.86**	**0.86**	36	0
Huang et al.	**1**	**0.67**	0	3

Note: The diagonal is the number of significant pathways. The percentages above (or below) the diagonal represent the number of the overlapping pathways divided by the number of the longer (or shorter) set of pathways. The values in bold font are the comparison result between the larger and smaller sample sizes.

### Integrative analysis identified key genes and pathways

**Figure 1:**
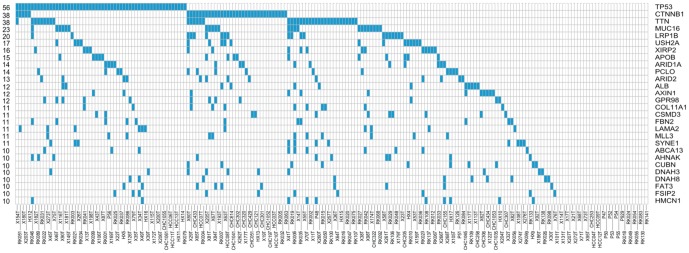
Overview of genes with mutations in at least 10 of 207 patient samples. The heatmap shows genes (rows) and tumors (columns) with mutations (blue). The number of events per gene is indicated to the left.

We integrated these four mutation profiles and obtained 7017 mutated genes (Table S2), among which 28 genes were mutated in no less than 10 samples (Figure 1).

Next, we analyzed the integrated mutated genes using the pathway coverage method and hypergeometric distribution model and compared the results obtained using these two methods. We found that with a 5% FDR control, 113 and 156 significantly mutated pathways were identified using the pathway coverage method and hypergeometric distribution model, respectively, and 112 pathways overlapped. Importantly, mutations of 156 pathways identified using the hypergeometric distribution model covered an average of 23% of the HCC tumor samples, whereas mutations of 113 pathways identified using the pathway coverage method covered an average of 29% of the HCC tumor samples. This result showed that the mutated genes included using the pathway coverage method better represented the tumor sample coverage. Thus, we only adopted the results obtained using the pathway coverage method for subsequent analyses.

**Figure 2:**
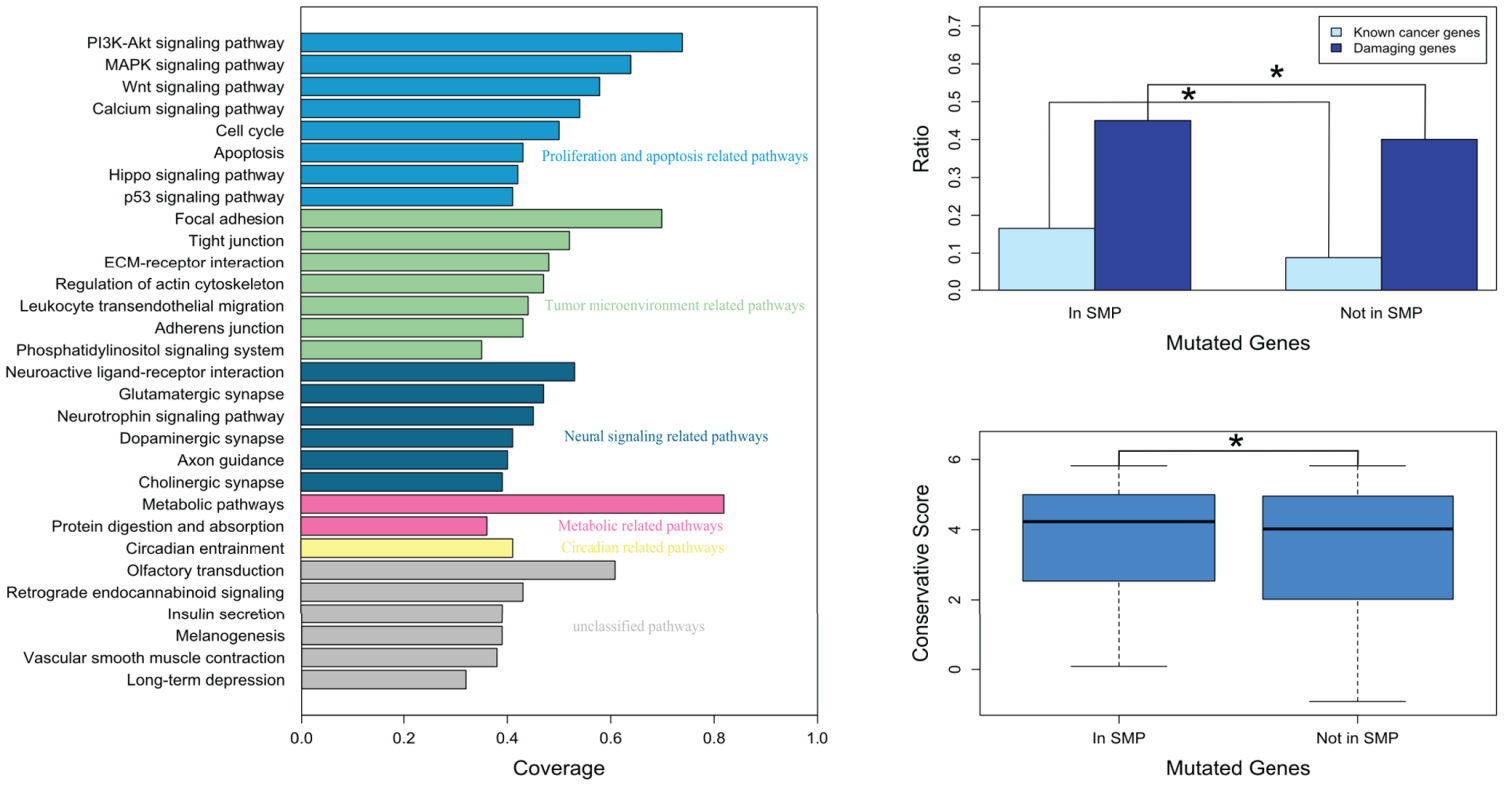
Significantly mutated pathways. **A,** Top 30 of 113 significantly mutated pathways and the difference in **B,** the percentage of known cancer genes or damaging genes and **C,** the conservative score between mutated genes in significantly mutated pathways (In SMP) and those not in significantly mutated pathways (Not In SMP). Coverage represents the fraction of tumors with at least one mutated gene in the specified pathway. Known cancer genes were obtained from the F-census database, and damaging genes were predicted using PolyPhen.

Among the 113 significantly mutated pathways identified using the pathway coverage method (Table S3), the top 30 pathways were mainly classified into five groups (Figure 2): (a) proliferation and apoptosis related pathways, (b) tumor microenvironment related pathways, (c) neural signaling related pathways, (d) metabolic related pathways, and (e) circadian related pathways.

From the total mutated genes, 2139 genes were included in 113 significantly mutated pathways (Table S4), and 4878 genes were not included. We speculated that the mutated genes implicated in these pathways were more likely to be candidate cancer genes. Thus, we compared the two sets of mutated genes (2139 vs. 4878) and found significant differences observed from three aspects, in support of our speculation (Figure 2): (1) the percentage of known cancer genes in the former was significantly higher compared to the latter (p<2.20×10^−16^); (2) the percentage of damaging genes, which contained at least one mutation predicted to affect protein function by PolyPhen, in the former was significantly higher compared to the latter (p = 1.09×10^−4^); and (3) the conservation scores of the former set of genes were significantly higher compared to those of the latter (p = 4.72×10^−4^).

**Figure 3:**
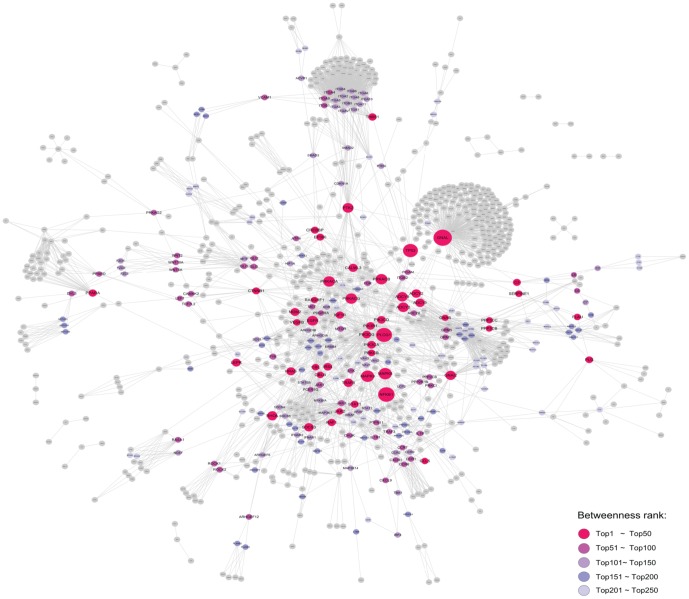
Biological network of mutated genes from significantly mutated pathways. The node size increases are proportionate to the increases in the betweenness coefficient.

**Figure 4:**
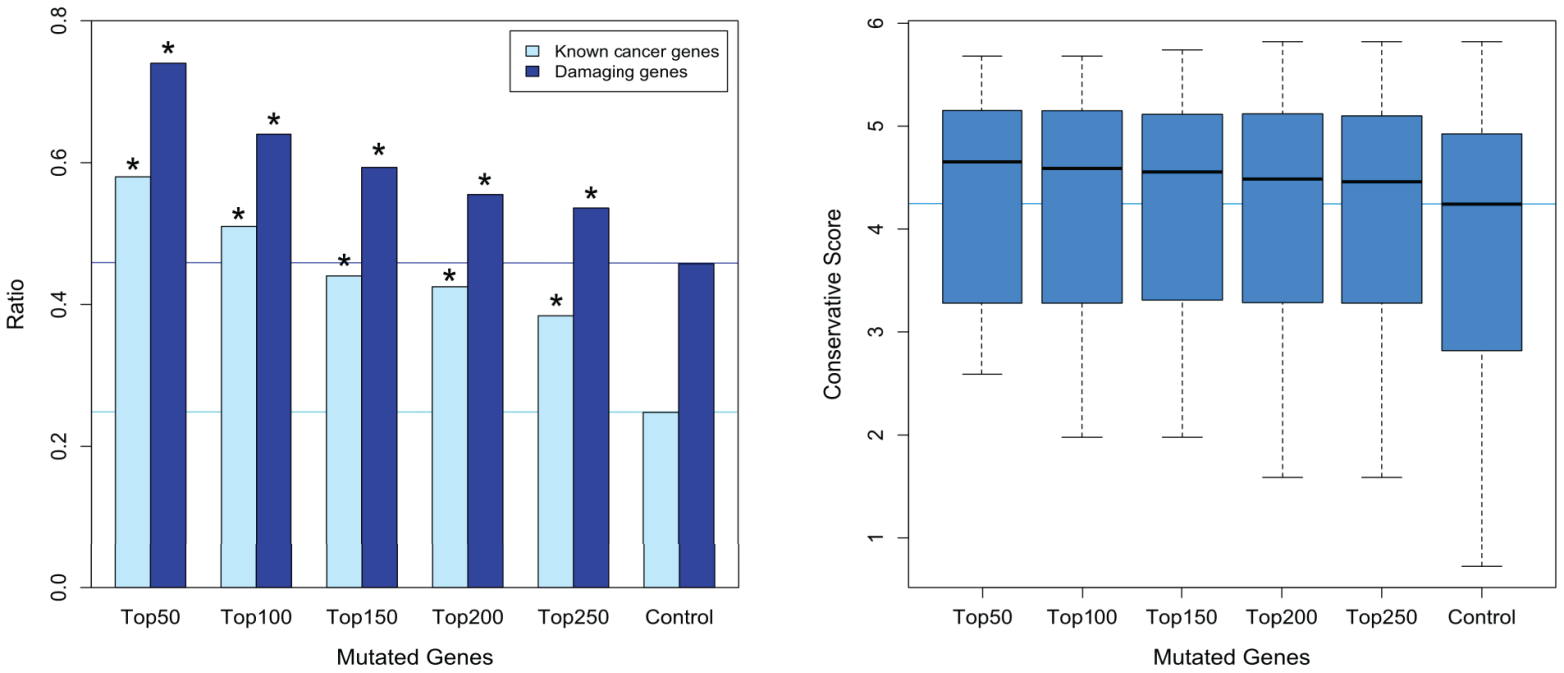
The difference in A, the percentage of known cancer genes or damaging genes and B, the conservative score between five groups of mutated genes and control genes. Five groups of mutated genes were ranked in the top 50, 100, 150, 200 and 250 by betweenness coefficient of the network. Control genes are mutated genes with a betweenness coefficient of zero. The horizontal line parallel to the x axis represents the longitudinal coordinates of the control genes. * represents a significant difference (p<0.05).

To further reveal the key mutated genes implicated in the significantly mutated pathways, we extracted the regulatory relationships of these pathways and generated an integrative signal transduction network (see Materials and Methods; Figure 3). A total of 896 mutated genes and 3108 edges in the integrative signal transduction network were obtained (Table S5). On the basis of network topology knowledge, a high betweenness coefficient or high clustering coefficient of a gene indicates its pivotal role in the network. Thus, we first ranked the mutated genes according to their betweenness coefficients and defined genes whose betweenness coefficients were zero as control genes. Next, we compared the five groups of mutated genes ranked in the top 50, 100, 150, 200 and 250 with the control genes. Our results indicated that the mutated genes with higher betweenness coefficients were more likely to play a key role in HCC on the basis of the following observations (Figure 4): (1) the percentage of known cancer genes in the five groups of mutated genes was significantly higher compared to the control genes (P-values of 3.24×10^−6^, 5.02×10^−7^, 1.33×10^−5^, 8.69×10^−6^, and 2.12×10^−4^ for the top 50, 100, 150, 200 and 250 genes, respectively); (2) the percentage of damaging genes in the five groups of mutated genes was significantly higher compared to the control genes (P-values of 1.57×10^−4^, 9.41×10^−4^, 0.005, 0.023, and 0.050, respectively, for the top 50, 100, 150, 200 and 250 genes); and (3) the conservation scores of the five groups of mutated genes were higher compared to the control genes. We also compared the top genes ranked by the clustering coefficient with the control genes, but no obvious trend was observed (data not shown).

### Clinical relevance of key mutated genes and significantly mutated pathways

To study the clinical relevance of the mutated genes significantly enriched in pathways, we calculated the correlations between the mutated genes and different clinical features of HCC patients. We found that at a significant P-value of 5%, mutations of four genes were correlated with cancer cell differentiation, mutations of four genes were correlated with metastasis, mutations of four genes were correlated with a lack of cirrhosis, and mutations of three genes were correlated with HBV infection. In addition, we found eight genes with mutations that might be associated with poorer overall survival rate of HCC patients (P≤0.1).

**Figure 5:**
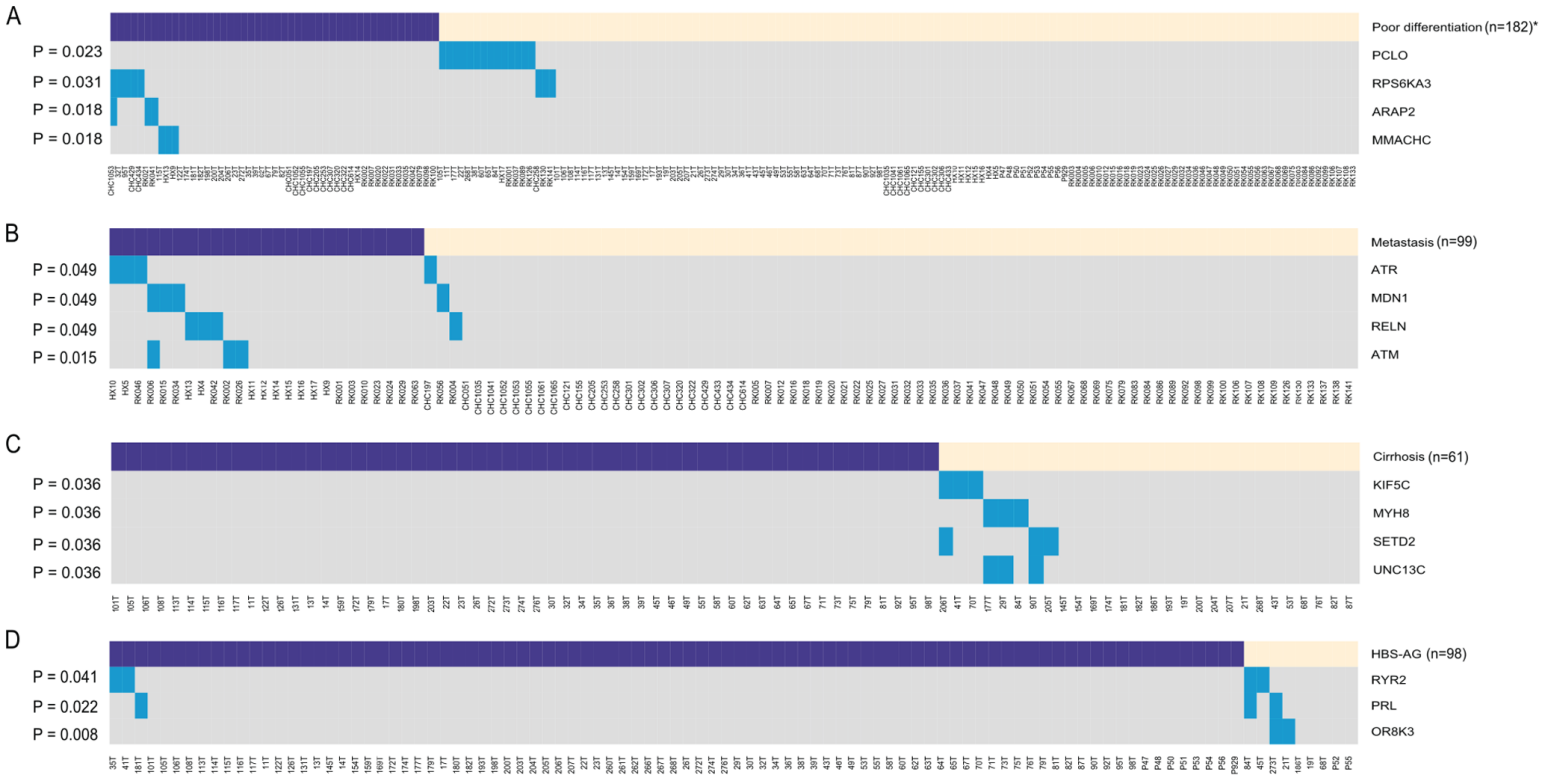
Overview of gene mutations and major associated clinical features, including A, Cell differentiation; B, Metastasis; C, Cirrhosis; and D, HBS-AG. The lower heatmap shows genes (rows) and tumors (columns) with mutations (blue). The purple (or yellow) boxes above the heatmap represent poor differentiation (or good differentiation), metastasis (or non-metastasis), cirrhosis (or non-cirrhosis) and HBS-AG positive (or HBS-AG negative), respectively. The P-value of each gene is indicated to the left. *n is the number of HCC patients with available clinical information.

As shown in Figure 5, poorly differentiated HCC demonstrated a significantly higher rate of RPS6KA3, ARAP2 and MMACHC mutations (10% vs. 2%, 6% vs. 0%, 6% vs. 0%; p = 0.031, p = 0.018, p = 0.018, respectively) and a significantly lower rate of PCLO mutations (0% vs. 10%, p = 0.023). There was also a trend toward higher rates of metastasis in HCC with ATM, ATR, MDN1 and RELN gene mutations (12% vs. 0%, 12% vs. 1%, 12% vs. 1%, 12% vs. 1%; p = 0.015, p = 0.049, p = 0.049, p = 0.049, respectively). In addition, KIF5C, MYH8, SETD2 and UNC13C mutations were only found in HCC patients in the absence of cirrhosis (0% vs. 11%, p = 0.036 for all four genes). HCC derived from hepatitis B infection demonstrated a significantly higher rate of OR8K3, PRL and RYR2 mutations (22% vs. 0%, 22% vs. 1%, 22% vs. 2%; p = 0.008, p = 0.022, p = 0.041, respectively). Moreover, patients with CACNA1I, COL5A1, CUL1, GRIA1, HERC1, MAGI1, NNT and OGT mutations tend to have poorer overall survival (median OS: 15 vs. 39 months, p = 0.084 for all eight genes), which requires further validation in a larger cohort of samples.

To study the clinical relevance of the significantly mutated pathways, we calculated the correlations between the pathway mutations and different clinical features of HCC patients. We found that at a significant P-value of 5%, two pathway mutations were correlated with cancer cell differentiation, six pathway mutations were correlated with metastasis, six pathway mutations were correlated with a lack of cirrhosis, and two pathway mutations were correlated with HBV infection. In addition, we found five pathways with mutations that might be correlated with an overall survival of HCC patients (three for poorer, two for better) (P≤0.1).

**Figure 6:**
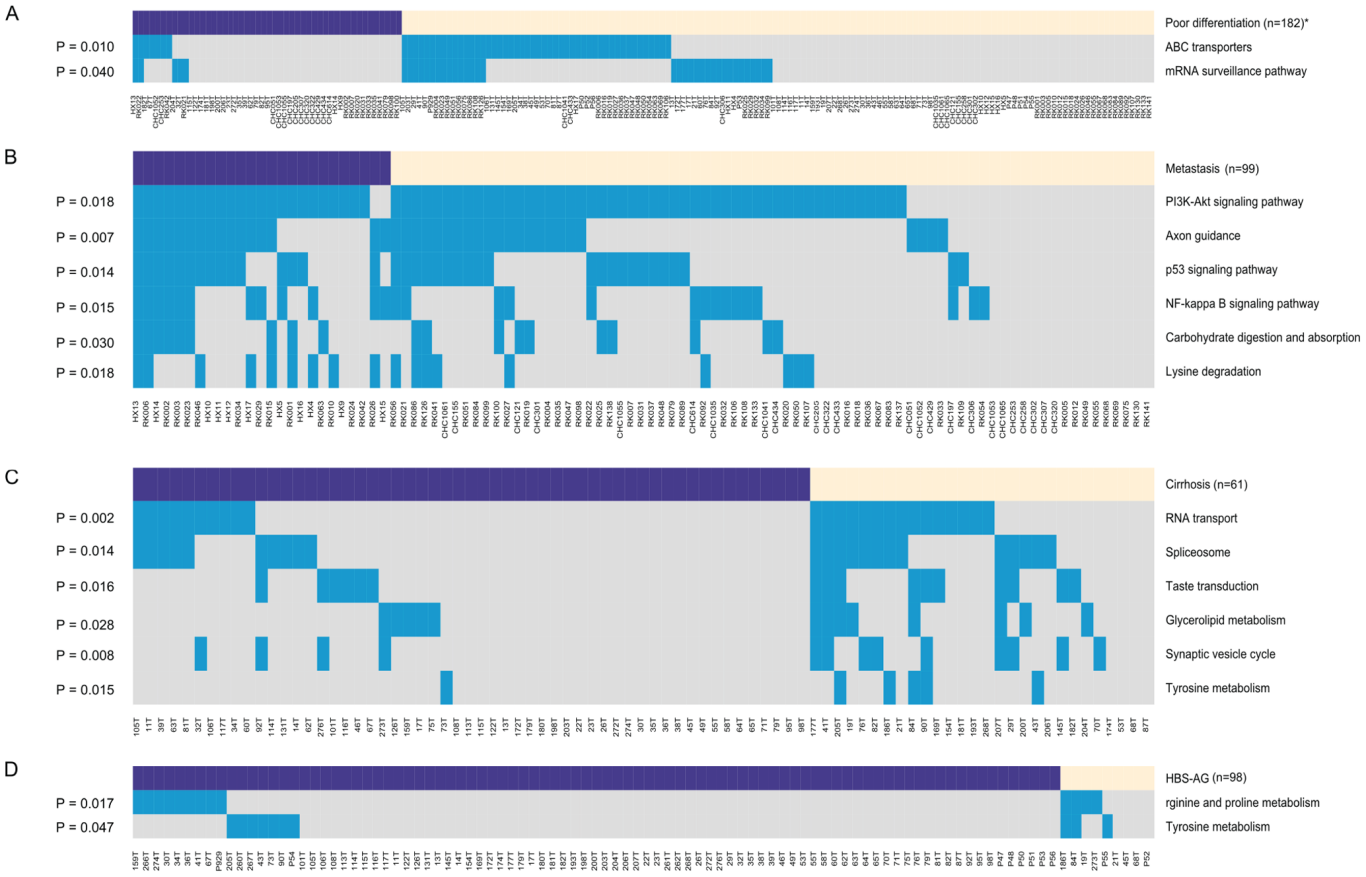
Overview of pathway mutations and major associated clinical features, including A, Cell differentiation; B, Metastasis; C, Cirrhosis; and D, HBS-AG. The lower heatmap shows pathways (rows) and tumors (columns) with mutations (blue). The purple (or yellow) boxes above the heatmap represent poor differentiation (or good differentiation), metastasis (or non-metastasis), cirrhosis (or non-cirrhosis) and HBS-AG positive (or HBS-AG negative), respectively. The P-value of each pathway is indicated to the left. *n is the number of HCC patients with available clinical information.

As shown in Figure 6, mutations in ABC transporters and mRNA surveillance pathway were associated with a trend toward better differentiation (15% vs. 36%, 10% vs. 25%; p = 0.010, 0.040, respectively). There was also a trend toward higher rates of metastasis in HCC with mutations of six pathways, including the p53 signaling pathway, PI3K-Akt signaling pathway, axon guidance, NF-kappa B signaling pathway, lysine degradation and carbohydrate digestion and absorption (60% vs. 30%, 92% vs. 68%, 64% vs. 31%, 48% vs. 20%, 36% vs. 12%, 36% vs. 14%; p = 0.014, p = 0.018, p = 0.007, p = 0.015, p = 0.018, p = 0.030, respectively). HCC that developed in the absence of cirrhosis demonstrated a significantly higher rate of mutation of six pathways, including tyrosine metabolism, glycerolipid metabolism, synaptic vesicle cycle, RNA transport, spliceosome and taste transduction (2% vs. 18%, 9% vs. 29%, 7% vs. 32%, 18% vs. 54%, 18% vs. 46%, 11% vs. 36%; p = 0.015, p = 0.028, p = 0.008, p = 0.002, p = 0.014, p = 0.016, respectively). Mutations in tyrosine metabolism and arginine and proline metabolism were associated with HCC derived from hepatitis B infection (8% vs. 33%, 10% vs. 44%; p = 0.047, p = 0.017, respectively). In addition, there was a slight tendency toward decreased overall survival status among tumors harboring mutations in the Notch signaling pathway, TGF-beta signaling pathway, and non-homologous end-joining (median OS: 15 vs. 40 months, 15 vs. 40 months, 15 vs. 39 months, p = 0.055, p = 0.059, p = 0.084, respectively) and increased overall survival status among tumors harboring mutations in the chemokine signaling pathway and pancreatic secretion (median OS: 51.5 vs. 20 months, 40 vs. 21 months, p = 0.090, p = 0.099, respectively). Such an association requires further validation in a larger cohort of samples.

## Discussion

We collected mutation data of HCC from four sources and integrated these data to increase the statistical power to pinpoint novel dominant cancer genes. We obtained a total of 7017 mutated genes, among which 28 genes were mutated in no less than 10 samples. In addition to six well-known and emerging cancer genes (TP53, CTNNB1, ARID1A, ARID2, AXIN1 and MLL3) in HCC [1], [2], [4], [6], [15], five genes (LRP1B, USH2A, ALB, GPR98 and COL11A1) were significantly mutated in HCC in the original mutation datasets analyzed in this study [4], [11] and our integrated results confirmed their high mutation frequencies. Importantly, our integrated results revealed 17 novel genes with mutational evidence to warrant experimental investigation. Among these 17 genes, previous studies have shown that five genes (MUC16, LAMA2, AHNAK, HMCN1 and FAT3) played a role in other cancer types [16], [17], [18], [19], [20]. For example, Rump and colleagues showed that binding of MUC16 to membrane-bound mesothelin mediated cancer cell adhesion in ovarian cancer and that the mesothelin-MUC16 interaction might result in the intraperitoneal dissemination of tumors [16]. Specifically, a novel nonsense mutation in exon 26 of APOB (p.K2240X) was responsible for low cholesterol and fatty liver in a large kindred, which might also be responsible for cirrhosis and liver cancer in this family [21]. However, the functional role of APOB in sporadic liver cancer is unknown. In addition, few studies have addressed the functional roles of the remaining 11 mutated genes (TTN, XIRP2, PCLO, CSMD3, FBN2, SYNE1, ABCA13, CUBN, DNAH3, DNAH8, and FSIP2) in cancer, which requires further investigation.

Next, we performed pathway analysis on the integrated mutated genes and identified 113 significantly mutated pathways. We showed that the mutated genes included in these pathways contained higher percentages of known cancer genes and damaging genes and also exhibited higher conservation scores, which indicate their important roles in HCC pathogenesis. The top 30 most significantly mutated pathways were mainly classified into five groups: (a) proliferation and apoptosis related pathways, (b) tumor microenvironment related pathways, (c) neural signaling related pathways, (d) metabolic related pathways, and (e) circadian related pathways.

For eight of the proliferation and apoptosis related pathways that were identified, it is well known that mutations of six pathways, including the cell cycle, p53 signaling pathway, apoptosis, Wnt signaling pathway, MAPK signaling pathway and PI3K-Akt signaling pathway, contribute to liver tumorigenesis [1], [2], [15]. In addition, we found that mutations of the calcium signaling pathway and Hippo signaling pathway covered 54% and 42% HCC tumors, respectively, thereby indicating that mutations in these pathways might significantly contribute to liver tumorigenesis. Indeed, it was reported that deregulation of the Hippo pathway can induce tumors in a broad range of human carcinomas, including lung, colorectal, ovarian and liver cancer, but mutations in Hippo pathway genes were rare [19]. For the calcium signaling pathway, increases in intracellular Ca2+ concentration organized in space, time and amplitude have been shown to be important in cell migration [22].

For the tumor microenvironment related pathways in which alterations play a role in the pathogenesis of HCC, most of the evidence was collected from transcriptional level experiments, but few studies have reported mutations in these pathways [23], [24]. Our analyses showed that, on average, mutations of eight tumor microenvironment related pathways covered 47% of HCC tumors. The eight pathways involved focal adhesion, ECM-receptor interaction, adherens junction, tight junction, leukocyte transendothelial migration, protein digestion and absorption, phosphatidylinositol signaling system and regulation of actin cytoskeleton; these are the mutations that are worthy of further study.

Interestingly, we also identified three groups of pathways, namely neural signaling, metabolic and circadian related pathways, in which their roles in liver tumorigenesis have not been addressed. Specifically, mutations in six neural signaling related pathways, including the neurotrophin signaling pathway, glutamatergic synapse, axon guidance, cholinergic synapse, dopaminergic synapse, and neuroactive ligand-receptor interaction, on average covered 44% of HCC tumors. It has been recognized that NGF and other neurotrophins regulate cell proliferation and invasion as well as cell death and survival, whereas dysregulation of neurotrophin signaling plays an important role in the pathogenesis of many tumors, such as breast and prostate cancer [25]. Moreover, mutations of two metabolic related pathways (metabolic pathways and protein digestion and absorption) and one circadian related pathway covered 59% and 41% HCC tumors, respectively. Recently, reprogrammed energy metabolism has been proposed as an emerging hallmark of cancer cells [26], and accumulating epidemiological and genetic evidence indicates that disruption of circadian rhythms might be directly linked to cancer [27]. Thus, the role of these mutations in neural signaling, metabolic and circadian related pathways in liver tumorigenesis merits further investigation.

Furthermore, we generated a signaling transduction network by extracting the mutated genes and the regulatory relationships of significantly mutated pathways and revealed that the mutated genes with the highest betweenness coefficients in the network might play key roles in these signaling pathways. Specifically, among the top 50 mutated genes with the highest betweenness coefficients, 11 well-known and emerging cancer genes (TP53, CTNNB1, PIK3CA, EGFR, IGF1R, JAK2, STAT1, NFKB1, LEPR, SOCS3, and HRAS) were observed in HCC [2], [15], [28], [29], [30]. For the remaining 12 genes (RAF1, TRAF6, PPARA, MAPK8, MAPK9, RHOA, MDM2, GNAS, PTK2, PLAU, VEGFB, and CCL5), there is some but not sufficient evidence to support their roles in liver tumorigenesis [31], [32], [33], [34], [35], [36], [37], [38], [39], [40], [41], which warrants further investigation. Importantly, our analyses revealed 27 novel genes with mutational evidence to warrant experimental investigation. Among the 27 genes, previous studies have reported that 19 genes (PLCG1, PRKACA, PRKACB, PRKACG, PRKCB, PIK3CD, PIK3CG, PIK3R1, C3, IRS4, EP300, CBL, CREBBP, GNAI2, THBS1, CBLB, SERPINE1, PPP3CB, and RASGRF1) play roles in cancer [42], [43], but few studies have addressed the functional roles of the remaining eight mutated genes (PLG, CALML3, PPP3CC, GNAL, ADCY3, ADCY8, ADCY2, and ADCY9).

Finally, we showed that mutations in 23 genes were correlated with the clinical features of HCC patients. The precise mechanism is unknown, but there are some clues. It has been reported that RPS6KA3 can control cell differentiation [44], which is consistent with our finding that RPS6KA3 tends to be mutated in poorly differentiated HCC. In addition, RPS6KA3 was included in the top 250 mutated genes with the highest betweenness coefficients in the signaling transduction network, and there are eight HCC samples that harbor RPS6KA3 mutations in our integrated mutation profile. Taken together, this evidence suggests that RPS6KA3 might play an important role in HCC pathogenesis. Furthermore, RELN has been suggested to be involved in ECM-receptor interaction and focal adhesion [45], which might be the mechanism underlying the high metastasis rate of HCC with RELN mutations. PRL is a growth regulator for cells of the immune system [46], and we showed that PRL mutations were associated with hepatitis B infection, suggesting that PRL might be implicated in the production of HBs-Ag. Furthermore, previous studies have shown that downregulation of MAGI1 is associated with poor prognosis of HCC [47] and that overexpression of CUL1 is associated with poor prognosis of patients with gastric cancer [48]. Our analysis demonstrated that mutations in MAGI1 and CUL1 were correlated with an overall survival status of HCC patients, indicating that these two genes might be prognostic markers in HCC. Importantly, we found that PCLO tended to be mutated in poorly differentiated HCC and that there were 14 HCC samples harboring PCLO mutations in our integrated mutation profile. Thus, the role of PCLO in HCC pathogenesis warrants further study.

At the pathway level, we identified 21 pathways in which the mutations were correlated with clinical features of HCC patients. For three of the six pathways (p53 signaling pathway, PI3K-Akt signaling pathway and NF-kappa B signaling pathway) relevant to metastasis rate, previous studies have shown that these pathways play important roles in HCC metastasis [49], [50]. As for the six pathways associated with the survival status of HCC patients (TGF-beta signaling pathway, Notch signaling pathway, non-homologous end-joining, etc.), the TGF-beta signaling pathway is well known to be activated in poor-prognosis HCC tumors [49] and downregulation of the Notch signaling pathway inhibits HCC cell invasion [51]. Interestingly, most evidence in previous studies was obtained from gene expression data; however, our results were based on mutation data. Importantly, we revealed 16 novel pathways, such as axon guidance and ABC transporters, associated with metastasis rate and cell differentiation of HCC patients, respectively. Specifically, axon guidance, a neural signaling related pathway, is one of the top 30 most significantly mutated pathways. It has been reported that an axon guidance molecule can enhance the invasion and metastasis of human gastric cancer [52], but the role of axon guidance in HCC invasion and metastasis has not been addressed, which requires further investigation.

In summary, we integrated mutation data of HCC from various sources, from which we identified that many of the signaling pathways were mutated in a significantly high percentage of HCC samples and primarily demonstrated that genes implicated in these pathways might play roles in HCC. We also generated a signal transduction network using mutated genes annotated in significantly mutated pathways and ranked these mutated genes according to their positions in the network, providing a reference for subsequent experiments. Finally, we showed that mutations of several key genes and pathways were associated with major clinical features. Our workflow illustrated the increased statistical power of integrating multiple studies of the same subject, which can provide biological insights that would otherwise be masked under individual sample sets. This type of bioinformatics approach is consistent with the necessity of making the best use of ever increasing data provided in valuable databases such as TCGA to enhance the speed of deciphering human cancers.

## Materials and Methods

### Datasets

The HCC mutation data were downloaded from ICGC (http://dcc.icgc.org/repository/current), Kan et al. (ERP001196 or www.ingenuity.com/acrg2012), Li et al. (http://www.nature.com/ng/journal/v43/n9/abs/ng.903.html#supplementary-information; Table S3) and Huang et al. (SRA053063), which detected 99, 88, 10 and 10 cancer samples, respectively. For each dataset, we first extracted non-synonymous point mutations, small insertions and deletions, filtering mutations in the non-coding region and synonymous point mutations. Next, we generated a mutation profile {X_ij_}: X_ij_ is 1 (true) if any mutation occurs in gene i in cancer sample j; otherwise, the element is 0 (false). We defined that the altered gene i covers the cancer sample j if X_ij_ is 1. In total, four mutation profiles were obtained for comparison analysis. Next, we pooled the four mutation profiles and obtained an integrated mutation profile, which consisted of 7017 genes across 207 HCC samples. Detailed clinical information of 207 patients (if available), including gender, age at diagnosis, tumor stage and grade, HBsAg, liver pathology and OS time, is listed in Table S6. These samples demonstrate typical Chinese HCC characteristics such as a high HBV infection rate and high rate of cirrhosis, among other factors.

In addition, 1654 known cancer genes were obtained from the F-census database [43], which is a collection of cancer genes from various data sources, including the Cancer Gene Census dataset [42] and Tumor Suppressor Gene database [53]. The effects of non-synonymous mutations on protein function were predicted using PolyPhen version 2 with default parameters [54], [55]. A gene with at least one damaging mutation, i.e., that is supposed to affect protein function, was referred as a damaging gene in this study. The conservation score of each mutation position was calculated using GERP++ with default parameters [56].

Pathway data were downloaded from KEGG [45]. Seven classes of pathways were collected in KEGG: metabolism, genetic information processing, environmental information processing, cellular processes, organismal systems, human diseases and drug development. We only adopted the former five classes, as the latter two classes were assembled pathways.

### Pathway analysis

Significant pathways that might be important in liver tumorigenesis were identified using the pathway coverage method and hypergeometric distribution model. We performed these two methods on five mutation profiles: four individual mutation profiles from different sources and the integrated mutation profile of the four profiles. The P-values for each analysis were adjusted using the Benjamini and Hochberg method for multiple-testing correction [57].

The pathway coverage method: A few studies have attempted to define a selected pathway as being significantly mutated if this mutated pathway (containing at least one mutated gene) covers a statistically significant percentage of all detected cancer samples [1], [12], [13]. We termed this method as “pathway coverage method” in this study. Briefly, a matrix {Y_ij_} was first built: Y_ij_ is 1 (true) if cancer sample j contains at least one mutated gene annotated in pathway i; otherwise, the element is 0 (false). We defined that the altered pathway i covers the cancer sample j if Y_ij_ is 1. The coverage (or alteration frequency) of a pathway is the number of cancer samples mutated divided by the number of all cancer samples. Considering that the coverage of a pathway is dependent on the number of genes annotated in this pathway, we assessed the empirical significance of the coverage of a pathway using simulations. For pathway i with v genes, we computed Z_i_, which is the number of cancer samples that pathway i covers. Each simulation sampled v genes without replacement from all genes on human genome and collected mutation statistics based on the mutation profile. The P-value of a pathway covering k samples is derived as the proportion of 10,000 simulations with Z_i_≥k.

The hypergeometric distribution model: According to the hypergeometric distribution model [14], a pathway is defined as being significantly mutated if this mutated pathway (containing at least one mutated gene) contains a statistically significant percentage of all detected mutated genes. Hypothetically, suppose a KEGG pathway contains M genes, k of which are genes mutated in at least one HCC patient for each dataset, then the probability of observing at least k mutated genes in this pathway by random chance is:
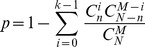
where N is the number of all genes and n is the number of all mutated genes annotated in the five classes of KEGG pathways.

### Network analysis

Network data were extracted from the KEGG database [45]. Nodes are genes, and edges represent nine regulatory relationships, including activation, inhibition, expression, repression, phosphorylation, dephosphorylation, methylation and ubiquitination. We mapped all mutated genes (2139) from significantly mutated pathways to these networks and generated an integrative signal transduction network. There were a total of 896 mutated genes and 3108 edges in the integrative signal transduction network (Table S5). Next, we calculated the betweenness coefficient and clustering coefficient of each gene in the network.

### Statistical analysis

The differences in the percentages of known cancer genes and damaging genes between two sets of mutated genes were calculated using Fisher's Exact test. The differences in the conservation scores between two sets of mutated genes were calculated using Student's t-test. Associations of the gene or pathway mutations and overall survival were identified using Cox proportional hazards regression models (log-rank test) with the R survival library. The association of gene or pathway mutations with other clinical factors was performed using the X-squared test (group size of >5) with Yates' continuity correction or Fisher's Exact test (group size of ≤5). All reported P-values were two tailed, and differences were considered significant when the P-value was less than 0.05.

## Supporting Information

Table S1 Overlap of four sets of significant pathways obtained using the hypergeometric distribution model.
(DOC)

Table S2 7017 mutated genes.
(DOC)

Table S3 113 significantly mutated pathways.
(DOC)

Table S4 Mutated genes implicated in 113 significantly mutated pathways.
(DOC)

Table S5 Mutated genes ranked by betweenness coefficient.
(DOC)

Table S6 Clinicopathological features of 207 HCC patients.
(DOC)
