# Supplementary material for: Integrated Analysis of Mutation Data from Various Sources Identifies Key Genes and Signaling Pathways in Hepatocellular Carcinoma
Source: PLoS One. 2014 Jul 2;9(7):e100854. doi: 10.1371/journal.pone.0100854 (PMC4079600; doi:10.1371/journal.pone.0100854)
Supplement: Table S1 — Overlap of four sets of significant pathways obtained using the hypergeometric distribution model. (DOC) [file pone.0100854.s001.doc]

Supplementary Table S1. Overlap of four sets of significant pathways obtained using the hypergeometric distribution model

|  | ICGC | Kan et al. | Li et al. | Huang et al. |
| --- | --- | --- | --- | --- |
| ICGC | 138 | 0.71 | **0.27** | **0.08** |
| Kan et al. | 0.91 | 108 | **0.35** | **0.09** |
| Li et al. | **0.93** | **0.95** | 40 | 0.08 |
| Huang et al. | **1** | **0.91** | 0.27 | 11 |

Note: The diagonal is the number of significant pathways. The percentages above (or below) the diagonal represent the number of the overlapping pathways divided by the number of the longer (or shorter) set of pathways. The values in bold fonts are the comparison result between the larger and smaller sample sizes.
