# Supplementary material for: Integrated Analysis of Mutation Data from Various Sources Identifies Key Genes and Signaling Pathways in Hepatocellular Carcinoma
Source: PLoS One. 2014 Jul 2;9(7):e100854. doi: 10.1371/journal.pone.0100854 (PMC4079600; doi:10.1371/journal.pone.0100854)
Supplement: Table S2 — 7017 mutated genes. (DOC) [file pone.0100854.s002.doc]

Supplementary Table S2 7017 mutated genes

| Gene Symbol | No. of mutation Samples |
| --- | --- |
| TP53 | 56 |
| CTNNB1 | 38 |
| TTN | 38 |
| MUC16 | 23 |
| LRP1B | 20 |
| USH2A | 17 |
| XIRP2 | 16 |
| APOB | 15 |
| ARID1A | 14 |
| PCLO | 14 |
| ARID2 | 13 |
| ALB | 12 |
| AXIN1 | 12 |
| GPR98 | 12 |
| COL11A1 | 11 |
| CSMD3 | 11 |
| FBN2 | 11 |
| LAMA2 | 11 |
| MLL3 | 11 |
| SYNE1 | 11 |
| ABCA13 | 10 |
| AHNAK | 10 |
| CUBN | 10 |
| DNAH3 | 10 |
| DNAH8 | 10 |
| FAT3 | 10 |
| FSIP2 | 10 |
| HMCN1 | 10 |
| EYS | 9 |
| IGF1R | 9 |
| RELN | 9 |
| ZNF804B | 9 |
| ACVR2A | 8 |
| CSMD1 | 8 |
| DNAH5 | 8 |
| DNAH9 | 8 |
| FAT4 | 8 |
| FRAS1 | 8 |
| JAK1 | 8 |
| LRRIQ1 | 8 |
| MACF1 | 8 |
| MDN1 | 8 |
| NALCN | 8 |
| OBSCN | 8 |
| PLXNA4 | 8 |
| RIMS1 | 8 |
| RPS6KA3 | 8 |
| RYR2 | 8 |
| SPTA1 | 8 |
| VAV3 | 8 |
| AHNAK2 | 7 |
| AMPH | 7 |
| ANK3 | 7 |
| ATP10B | 7 |
| ATR | 7 |
| BDP1 | 7 |
| COL24A1 | 7 |
| COL6A5 | 7 |
| DCC | 7 |
| DYNC2H1 | 7 |
| EPHA5 | 7 |
| ERBB4 | 7 |
| FAM5C | 7 |
| KEAP1 | 7 |
| LRFN5 | 7 |
| LRP2 | 7 |
| MAP1B | 7 |
| MAP2 | 7 |
| MUC4 | 7 |
| MXRA5 | 7 |
| NAV3 | 7 |
| NFE2L2 | 7 |
| PBRM1 | 7 |
| PCDH15 | 7 |
| PKHD1 | 7 |
| PKHD1L1 | 7 |
| PLCB1 | 7 |
| RYR3 | 7 |
| SCN3A | 7 |
| VPS13B | 7 |
| ADCY2 | 6 |
| BAI3 | 6 |
| BPTF | 6 |
| CACNA1C | 6 |
| CACNA2D4 | 6 |
| CHD5 | 6 |
| DNAH17 | 6 |
| DNAH7 | 6 |
| DOCK2 | 6 |
| DST | 6 |
| DYNC1H1 | 6 |
| GRIA4 | 6 |
| HELZ | 6 |
| HERC1 | 6 |
| HTT | 6 |
| HUWE1 | 6 |
| IGSF10 | 6 |
| KCNH8 | 6 |
| LAMA1 | 6 |
| MUC17 | 6 |
| MUC19 | 6 |
| MYO10 | 6 |
| OTOGL | 6 |
| PCDH7 | 6 |
| PCDHA2 | 6 |
| PLCB4 | 6 |
| PRRC2C | 6 |
| PRUNE2 | 6 |
| RB1 | 6 |
| SLITRK5 | 6 |
| SYNE2 | 6 |
| VIM | 6 |
| VWDE | 6 |
| WDFY4 | 6 |
| WWP1 | 6 |
| ADAMTS20 | 5 |
| ALK | 5 |
| ANKRD30A | 5 |
| ATM | 5 |
| BAZ2B | 5 |
| BRCA2 | 5 |
| CACNA1H | 5 |
| CACNA2D3 | 5 |
| CD5L | 5 |
| CDH7 | 5 |
| CDH9 | 5 |
| CHL1 | 5 |
| CNTN1 | 5 |
| CNTN6 | 5 |
| COL22A1 | 5 |
| COL5A1 | 5 |
| COL5A3 | 5 |
| CSMD2 | 5 |
| DDX60 | 5 |
| DMD | 5 |
| DMXL1 | 5 |
| DNAH10 | 5 |
| DNAH2 | 5 |
| DOCK3 | 5 |
| DOPEY1 | 5 |
| EPHA3 | 5 |
| FCRL3 | 5 |
| FGA | 5 |
| FLG | 5 |
| FLT3 | 5 |
| FREM1 | 5 |
| FRYL | 5 |
| FSTL4 | 5 |
| GOLGB1 | 5 |
| HIVEP3 | 5 |
| HNF1A | 5 |
| KCNB2 | 5 |
| KIAA0913 | 5 |
| KIAA1107 | 5 |
| LARP4 | 5 |
| LATS1 | 5 |
| LIPI | 5 |
| LTBP2 | 5 |
| MKI67 | 5 |
| MMRN1 | 5 |
| MYH4 | 5 |
| MYH6 | 5 |
| MYO18B | 5 |
| NEB | 5 |
| NID2 | 5 |
| NRXN1 | 5 |
| PCDH17 | 5 |
| PDZRN3 | 5 |
| PIK3CG | 5 |
| PTEN | 5 |
| PTPRB | 5 |
| PTPRN2 | 5 |
| REST | 5 |
| RP1 | 5 |
| SACS | 5 |
| SAMD9L | 5 |
| SDK1 | 5 |
| SEC23A | 5 |
| SLC39A10 | 5 |
| SRCAP | 5 |
| TAF1L | 5 |
| TECTA | 5 |
| TEX15 | 5 |
| THSD7B | 5 |
| TNR | 5 |
| TNRC6A | 5 |
| TPO | 5 |
| TRPC4 | 5 |
| UBR3 | 5 |
| UBR4 | 5 |
| UNC13C | 5 |
| UTRN | 5 |
| VCAN | 5 |
| WDR64 | 5 |
| WSCD2 | 5 |
| ZNF208 | 5 |
| ABCA12 | 4 |
| ABCC2 | 4 |
| ABCC9 | 4 |
| ACSS3 | 4 |
| ADAMTS9 | 4 |
| ADAMTSL1 | 4 |
| AKAP11 | 4 |
| AKAP13 | 4 |
| AKD1 | 4 |
| ALMS1 | 4 |
| ANKAR | 4 |
| ANKHD1-EIF4EBP3 | 4 |
| ANKS1B | 4 |
| APC | 4 |
| ARID1B | 4 |
| ASCC3 | 4 |
| ASH1L | 4 |
| ATG2B | 4 |
| BIRC6 | 4 |
| BLM | 4 |
| C18orf34 | 4 |
| C7orf63 | 4 |
| CACNA1I | 4 |
| CDH18 | 4 |
| CDH26 | 4 |
| CDH8 | 4 |
| CELF1 | 4 |
| CLCA2 | 4 |
| CNBD1 | 4 |
| COL12A1 | 4 |
| COL19A1 | 4 |
| CPNE4 | 4 |
| CSPG4 | 4 |
| CTNND1 | 4 |
| CUL9 | 4 |
| DBC1 | 4 |
| DCAF4L2 | 4 |
| DCHS2 | 4 |
| DCP1B | 4 |
| DENND1A | 4 |
| DMGDH | 4 |
| DNAH12 | 4 |
| DNAH14 | 4 |
| DOCK10 | 4 |
| DOCK6 | 4 |
| DSCAM | 4 |
| DSP | 4 |
| DYRK1A | 4 |
| EPHA4 | 4 |
| EPHA6 | 4 |
| ERCC6 | 4 |
| EVC2 | 4 |
| EXD2 | 4 |
| EXOC4 | 4 |
| FAM46D | 4 |
| FAT2 | 4 |
| FBN1 | 4 |
| FLNB | 4 |
| FLRT2 | 4 |
| FRRS1 | 4 |
| GCLC | 4 |
| GLI3 | 4 |
| GPATCH8 | 4 |
| GPR110 | 4 |
| GPR158 | 4 |
| GRID2 | 4 |
| GRIK4 | 4 |
| GRM3 | 4 |
| GRM4 | 4 |
| GTDC1 | 4 |
| HECW2 | 4 |
| HGF | 4 |
| HHIP | 4 |
| HNF4A | 4 |
| IGSF1 | 4 |
| IL6ST | 4 |
| ITPR2 | 4 |
| ITPR3 | 4 |
| KANK1 | 4 |
| KCNJ16 | 4 |
| KCNU1 | 4 |
| KDM3A | 4 |
| KDR | 4 |
| KIF5C | 4 |
| KRT82 | 4 |
| L2HGDH | 4 |
| LAMA3 | 4 |
| LIFR | 4 |
| LPHN2 | 4 |
| LPHN3 | 4 |
| LPIN2 | 4 |
| LRRC30 | 4 |
| LSS | 4 |
| LTBP1 | 4 |
| MAGI1 | 4 |
| MCTP2 | 4 |
| MECOM | 4 |
| MED12L | 4 |
| MED14 | 4 |
| MEOX2 | 4 |
| MET | 4 |
| MLL | 4 |
| MSH4 | 4 |
| MYH14 | 4 |
| MYH8 | 4 |
| MYLK | 4 |
| NBEA | 4 |
| NCAM2 | 4 |
| NCAPD3 | 4 |
| NIN | 4 |
| NLRP3 | 4 |
| NLRP7 | 4 |
| NNT | 4 |
| NOTCH2 | 4 |
| NRXN3 | 4 |
| NSMAF | 4 |
| ODZ2 | 4 |
| ODZ3 | 4 |
| ODZ4 | 4 |
| OGT | 4 |
| OR10K1 | 4 |
| OR10Z1 | 4 |
| OR4C12 | 4 |
| PAM | 4 |
| PCDH10 | 4 |
| PCDHB4 | 4 |
| PCDHGA2 | 4 |
| PDZD2 | 4 |
| PHF10 | 4 |
| PIK3R4 | 4 |
| PLCL1 | 4 |
| PLEKHG4B | 4 |
| POGZ | 4 |
| POLQ | 4 |
| PRKD1 | 4 |
| PRKG2 | 4 |
| PROM1 | 4 |
| PRPF40A | 4 |
| PRRC2B | 4 |
| PRTG | 4 |
| PTPN14 | 4 |
| PTPRD | 4 |
| PTPRQ | 4 |
| RAD54L | 4 |
| RALGAPA2 | 4 |
| RAPGEF2 | 4 |
| RERE | 4 |
| REV3L | 4 |
| RGAG1 | 4 |
| RNF17 | 4 |
| ROBO2 | 4 |
| SCN4A | 4 |
| SCN5A | 4 |
| SEMA3E | 4 |
| SENP6 | 4 |
| SETD2 | 4 |
| SETDB1 | 4 |
| SF3B1 | 4 |
| SH3RF3 | 4 |
| SLC44A5 | 4 |
| SLC5A9 | 4 |
| SLC8A1 | 4 |
| SMARCAL1 | 4 |
| SPAG17 | 4 |
| SPATA20 | 4 |
| SPECC1L | 4 |
| SPHKAP | 4 |
| SPTAN1 | 4 |
| SRP72 | 4 |
| SUPT6H | 4 |
| SYCP2 | 4 |
| TBCK | 4 |
| TMCC2 | 4 |
| TMEM132C | 4 |
| TNRC6B | 4 |
| TPR | 4 |
| TRIP11 | 4 |
| TRPS1 | 4 |
| TRPV5 | 4 |
| TSC2 | 4 |
| TTC37 | 4 |
| UBE4B | 4 |
| UBR5 | 4 |
| UHRF1BP1L | 4 |
| UNC13B | 4 |
| UTP20 | 4 |
| VCP | 4 |
| VEPH1 | 4 |
| VPS13C | 4 |
| WDR17 | 4 |
| XRN1 | 4 |
| ZAN | 4 |
| ZEB1 | 4 |
| ZFC3H1 | 4 |
| ZFPM2 | 4 |
| ZNF254 | 4 |
| ZNF268 | 4 |
| ZNF638 | 4 |
| ZNF770 | 4 |
| ZNF831 | 4 |
| ZP4 | 4 |
| A2ML1 | 3 |
| AACS | 3 |
| AADACL2 | 3 |
| ABCA5 | 3 |
| ABCB5 | 3 |
| ABCC4 | 3 |
| ABCG5 | 3 |
| ABP1 | 3 |
| ACAA2 | 3 |
| ACP2 | 3 |
| ADAM12 | 3 |
| ADAM23 | 3 |
| ADAMTS19 | 3 |
| ADAMTSL3 | 3 |
| ADCY9 | 3 |
| ADD2 | 3 |
| ADH4 | 3 |
| AFF1 | 3 |
| AFF2 | 3 |
| AHCTF1 | 3 |
| AHI1 | 3 |
| AKAP6 | 3 |
| AMPD3 | 3 |
| ANK2 | 3 |
| ANKRD26 | 3 |
| AOX1 | 3 |
| APAF1 | 3 |
| APBA2 | 3 |
| ARAP2 | 3 |
| ARFGEF1 | 3 |
| ARHGAP10 | 3 |
| ARHGAP15 | 3 |
| ARHGAP21 | 3 |
| ARHGAP35 | 3 |
| ARHGAP5 | 3 |
| ASPM | 3 |
| ASXL3 | 3 |
| ATP10A | 3 |
| ATP11B | 3 |
| ATP13A1 | 3 |
| ATP13A5 | 3 |
| ATP6V1A | 3 |
| ATP8A1 | 3 |
| ATP8B4 | 3 |
| ATRNL1 | 3 |
| ATRX | 3 |
| B4GALNT1 | 3 |
| BOC | 3 |
| BRD7 | 3 |
| BRE | 3 |
| BRWD3 | 3 |
| BSN | 3 |
| BZRAP1 | 3 |
| C10orf137 | 3 |
| C10orf90 | 3 |
| C11orf30 | 3 |
| C12orf63 | 3 |
| C16orf62 | 3 |
| C1S | 3 |
| C20orf26 | 3 |
| C3 | 3 |
| C3orf77 | 3 |
| C6orf118 | 3 |
| C6orf165 | 3 |
| CACHD1 | 3 |
| CACNA1E | 3 |
| CACNA1S | 3 |
| CADPS2 | 3 |
| CASP8AP2 | 3 |
| CBL | 3 |
| CC2D2A | 3 |
| CCBL1 | 3 |
| CCDC129 | 3 |
| CCNG1 | 3 |
| CCT8L2 | 3 |
| CD109 | 3 |
| CD163L1 | 3 |
| CD180 | 3 |
| CDC7 | 3 |
| CDH12 | 3 |
| CDH19 | 3 |
| CDKN1A | 3 |
| CENPE | 3 |
| CEP152 | 3 |
| CEP290 | 3 |
| CEP57 | 3 |
| CFTR | 3 |
| CGN | 3 |
| CHD1L | 3 |
| CHD9 | 3 |
| CHGB | 3 |
| CHRDL1 | 3 |
| CIT | 3 |
| CIZ1 | 3 |
| CLCA4 | 3 |
| CLEC7A | 3 |
| CLIP1 | 3 |
| CLIP4 | 3 |
| CLSTN2 | 3 |
| CLUL1 | 3 |
| CNTN4 | 3 |
| CNTNAP2 | 3 |
| COBL | 3 |
| COL15A1 | 3 |
| COL1A1 | 3 |
| COL21A1 | 3 |
| COL4A5 | 3 |
| CPSF6 | 3 |
| CREB3L2 | 3 |
| CUL1 | 3 |
| CUL5 | 3 |
| CYLC1 | 3 |
| DACH2 | 3 |
| DAPK1 | 3 |
| DCHS1 | 3 |
| DGKB | 3 |
| DHTKD1 | 3 |
| DHX8 | 3 |
| DIP2B | 3 |
| DIS3 | 3 |
| DIS3L2 | 3 |
| DISC1 | 3 |
| DLG5 | 3 |
| DNAH11 | 3 |
| DNAH6 | 3 |
| DNAJC11 | 3 |
| DNAJC22 | 3 |
| DNMT3A | 3 |
| DOCK11 | 3 |
| DONSON | 3 |
| DPYSL5 | 3 |
| DSCAML1 | 3 |
| DSG1 | 3 |
| DVL3 | 3 |
| EFR3A | 3 |
| EHBP1 | 3 |
| EIF2AK2 | 3 |
| EIF2C3 | 3 |
| EIF4G1 | 3 |
| ELF4 | 3 |
| ELMO1 | 3 |
| ELP2 | 3 |
| EMILIN3 | 3 |
| EMR1 | 3 |
| ENPP3 | 3 |
| EP300 | 3 |
| EPB41L3 | 3 |
| EPHA1 | 3 |
| EPHA7 | 3 |
| EPS15 | 3 |
| EPS8L1 | 3 |
| ERAP1 | 3 |
| ERRFI1 | 3 |
| F5 | 3 |
| FABP6 | 3 |
| FAM135B | 3 |
| FAM171A1 | 3 |
| FAM75D1 | 3 |
| FANCM | 3 |
| FAT1 | 3 |
| FBXO11 | 3 |
| FBXO40 | 3 |
| FCRL5 | 3 |
| FER1L5 | 3 |
| FER1L6 | 3 |
| FGFBP1 | 3 |
| FILIP1 | 3 |
| FNDC1 | 3 |
| FOLH1 | 3 |
| FOXP2 | 3 |
| FREM2 | 3 |
| FRMPD4 | 3 |
| FRY | 3 |
| FUT9 | 3 |
| G6PC | 3 |
| GABRA1 | 3 |
| GABRA2 | 3 |
| GABRB2 | 3 |
| GABRG1 | 3 |
| GABRG3 | 3 |
| GART | 3 |
| GFM1 | 3 |
| GFRAL | 3 |
| GK5 | 3 |
| GLRB | 3 |
| GLYCTK | 3 |
| GMFG | 3 |
| GNPTAB | 3 |
| GPNMB | 3 |
| GPR112 | 3 |
| GPR116 | 3 |
| GPR52 | 3 |
| GRIA1 | 3 |
| GRIN2A | 3 |
| GUCY1A2 | 3 |
| GXYLT1 | 3 |
| GYS2 | 3 |
| HACE1 | 3 |
| HCN1 | 3 |
| HDAC4 | 3 |
| HFM1 | 3 |
| HIRIP3 | 3 |
| HIST1H4B | 3 |
| HIVEP2 | 3 |
| HS3ST5 | 3 |
| HSPA9 | 3 |
| HSPG2 | 3 |
| IARS | 3 |
| ICA1 | 3 |
| IL1R1 | 3 |
| IL21R | 3 |
| IL31RA | 3 |
| IL7R | 3 |
| IMPG2 | 3 |
| INADL | 3 |
| INPP4B | 3 |
| INTS3 | 3 |
| IP6K3 | 3 |
| IRF3 | 3 |
| ITGA1 | 3 |
| ITGA8 | 3 |
| ITGAD | 3 |
| ITGAE | 3 |
| ITGAV | 3 |
| ITIH1 | 3 |
| ITPKB | 3 |
| ITPKC | 3 |
| ITPR1 | 3 |
| ITSN1 | 3 |
| ITSN2 | 3 |
| JAK2 | 3 |
| JAK3 | 3 |
| KCNH4 | 3 |
| KCNT2 | 3 |
| KDM6A | 3 |
| KIAA0240 | 3 |
| KIAA1109 | 3 |
| KIAA1549 | 3 |
| KIAA1731 | 3 |
| KIF1A | 3 |
| KIF1B | 3 |
| KIF3A | 3 |
| KIF5A | 3 |
| KIRREL | 3 |
| KLF15 | 3 |
| KPNA3 | 3 |
| KRT13 | 3 |
| KSR1 | 3 |
| LAMB1 | 3 |
| LAMC2 | 3 |
| LCORL | 3 |
| LCP2 | 3 |
| LCT | 3 |
| LCTL | 3 |
| LILRB1 | 3 |
| LINGO2 | 3 |
| LPIN1 | 3 |
| LRBA | 3 |
| LRP1 | 3 |
| LRRC16B | 3 |
| LRRC4B | 3 |
| LRRC55 | 3 |
| LRRFIP1 | 3 |
| LRRIQ3 | 3 |
| LRRTM1 | 3 |
| LUZP2 | 3 |
| LYST | 3 |
| MAGEC1 | 3 |
| MAP1A | 3 |
| MAP3K12 | 3 |
| MAP3K4 | 3 |
| 6-Mar | 3 |
| MAST4 | 3 |
| MBD5 | 3 |
| MBTPS1 | 3 |
| MC4R | 3 |
| MCTP1 | 3 |
| MEGF10 | 3 |
| MERTK | 3 |
| MIA3 | 3 |
| MLLT4 | 3 |
| MMACHC | 3 |
| MMP27 | 3 |
| MORC2 | 3 |
| MPHOSPH9 | 3 |
| MPP1 | 3 |
| MRRF | 3 |
| MS4A6A | 3 |
| MTMR1 | 3 |
| MTMR9 | 3 |
| MUC2 | 3 |
| MUSK | 3 |
| MYCBPAP | 3 |
| MYH1 | 3 |
| MYH10 | 3 |
| MYH11 | 3 |
| MYH13 | 3 |
| MYH2 | 3 |
| MYH7 | 3 |
| MYO6 | 3 |
| MYO7B | 3 |
| MYO9A | 3 |
| MYOCD | 3 |
| NACA | 3 |
| NAV2 | 3 |
| NBAS | 3 |
| NBEAL1 | 3 |
| NCAPG2 | 3 |
| NCOA6 | 3 |
| NEBL | 3 |
| NF1 | 3 |
| NFASC | 3 |
| NFAT5 | 3 |
| NLGN1 | 3 |
| NLRP1 | 3 |
| NLRP10 | 3 |
| NLRP11 | 3 |
| NMUR2 | 3 |
| NOS1 | 3 |
| NOTCH3 | 3 |
| NOX3 | 3 |
| NPAS3 | 3 |
| NPHP1 | 3 |
| NPY5R | 3 |
| NR3C2 | 3 |
| NRCAM | 3 |
| NTNG1 | 3 |
| NTRK2 | 3 |
| NWD1 | 3 |
| ODF2 | 3 |
| ODZ1 | 3 |
| OLA1 | 3 |
| OPA1 | 3 |
| OPCML | 3 |
| OR12D3 | 3 |
| OR2M5 | 3 |
| OR2T4 | 3 |
| OR5AP2 | 3 |
| OR8K3 | 3 |
| OR8K5 | 3 |
| OR9G4 | 3 |
| OSBPL7 | 3 |
| OTOP1 | 3 |
| PACS2 | 3 |
| PADI4 | 3 |
| PAN2 | 3 |
| PAPOLB | 3 |
| PAPPA | 3 |
| PCDH18 | 3 |
| PCDH20 | 3 |
| PCDH9 | 3 |
| PCDHA1 | 3 |
| PCDHA4 | 3 |
| PCDHA6 | 3 |
| PCDHB11 | 3 |
| PCDHB14 | 3 |
| PCDHGA4 | 3 |
| PCSK5 | 3 |
| PDE10A | 3 |
| PDE3B | 3 |
| PDGFRA | 3 |
| PDK4 | 3 |
| PDS5B | 3 |
| PECR | 3 |
| PGR | 3 |
| PHF20 | 3 |
| PHRF1 | 3 |
| PIK3CA | 3 |
| PIKFYVE | 3 |
| PKN2 | 3 |
| PLCE1 | 3 |
| PLCH1 | 3 |
| PLEKHH2 | 3 |
| PLG | 3 |
| PMPCB | 3 |
| POLR1A | 3 |
| POM121L12 | 3 |
| POSTN | 3 |
| PPFIA2 | 3 |
| PPP1R16B | 3 |
| PPP1R3A | 3 |
| PPP6R3 | 3 |
| PRDM5 | 3 |
| PREX2 | 3 |
| PRICKLE2 | 3 |
| PRKDC | 3 |
| PRL | 3 |
| PROX1 | 3 |
| PTGFRN | 3 |
| PTPN3 | 3 |
| PTPRJ | 3 |
| PTPRK | 3 |
| PTPRM | 3 |
| PTPRT | 3 |
| PTPRZ1 | 3 |
| PXDNL | 3 |
| RAB3GAP2 | 3 |
| RALGAPB | 3 |
| RANBP2 | 3 |
| RASGRF1 | 3 |
| RASSF9 | 3 |
| RBM34 | 3 |
| RC3H2 | 3 |
| RGS7 | 3 |
| RIMS2 | 3 |
| RNF38 | 3 |
| ROS1 | 3 |
| RPGRIP1 | 3 |
| RSPH6A | 3 |
| RTEL1 | 3 |
| RTTN | 3 |
| RUNX1T1 | 3 |
| RXRG | 3 |
| RYR1 | 3 |
| SAGE1 | 3 |
| SATB2 | 3 |
| SCAF4 | 3 |
| SCG2 | 3 |
| SCN1A | 3 |
| SCN7A | 3 |
| SCUBE2 | 3 |
| SEL1L3 | 3 |
| SEMA4B | 3 |
| SEMG1 | 3 |
| SETX | 3 |
| SF3B3 | 3 |
| SFXN2 | 3 |
| SH2D3C | 3 |
| SH3BP5 | 3 |
| SHOX2 | 3 |
| SIGLEC12 | 3 |
| SIGLEC8 | 3 |
| SIGLEC9 | 3 |
| SIPA1L1 | 3 |
| SLC10A1 | 3 |
| SLC1A6 | 3 |
| SLC27A6 | 3 |
| SLC38A6 | 3 |
| SLC43A3 | 3 |
| SLC6A15 | 3 |
| SLC6A5 | 3 |
| SLC6A7 | 3 |
| SLC7A11 | 3 |
| SLIT2 | 3 |
| SMARCA4 | 3 |
| SMARCC1 | 3 |
| SMC5 | 3 |
| SNRNP200 | 3 |
| SNX18 | 3 |
| SNX5 | 3 |
| SORL1 | 3 |
| SOX30 | 3 |
| SP3 | 3 |
| SPATA18 | 3 |
| SPEG | 3 |
| SPEN | 3 |
| SPHK2 | 3 |
| SPOCD1 | 3 |
| SRRM2 | 3 |
| STARD9 | 3 |
| STON1-GTF2A1L | 3 |
| STXBP5L | 3 |
| SUPT5H | 3 |
| SYNCRIP | 3 |
| TAS1R1 | 3 |
| TAS2R5 | 3 |
| TBC1D12 | 3 |
| TBL1XR1 | 3 |
| TCHH | 3 |
| TDRD6 | 3 |
| TENC1 | 3 |
| TET1 | 3 |
| TF | 3 |
| TFAP2B | 3 |
| TGM2 | 3 |
| THADA | 3 |
| THBS2 | 3 |
| THEMIS | 3 |
| TLL1 | 3 |
| TM7SF2 | 3 |
| TMC5 | 3 |
| TMEM168 | 3 |
| TMEM2 | 3 |
| TMEM201 | 3 |
| TNC | 3 |
| TNKS1BP1 | 3 |
| TNN | 3 |
| TOP2B | 3 |
| TPTE | 3 |
| TRANK1 | 3 |
| TRAPPC9 | 3 |
| TRIO | 3 |
| TRRAP | 3 |
| TSC1 | 3 |
| TTBK1 | 3 |
| TTC3 | 3 |
| TXLNB | 3 |
| TXNRD3 | 3 |
| UNC13A | 3 |
| UNC45A | 3 |
| UNC5C | 3 |
| USP19 | 3 |
| USP25 | 3 |
| USP29 | 3 |
| USP32 | 3 |
| USP53 | 3 |
| VPS13A | 3 |
| VPS41 | 3 |
| VWF | 3 |
| WDFY3 | 3 |
| WDR11 | 3 |
| WDR33 | 3 |
| WDR52 | 3 |
| WDR66 | 3 |
| WDR7 | 3 |
| WDR70 | 3 |
| YIPF7 | 3 |
| ZC3H13 | 3 |
| ZFHX2 | 3 |
| ZFHX3 | 3 |
| ZFHX4 | 3 |
| ZFYVE16 | 3 |
| ZNF100 | 3 |
| ZNF101 | 3 |
| ZNF131 | 3 |
| ZNF226 | 3 |
| ZNF236 | 3 |
| ZNF280D | 3 |
| ZNF438 | 3 |
| ZNF469 | 3 |
| ZNF479 | 3 |
| ZNF521 | 3 |
| ZNF536 | 3 |
| ZNF554 | 3 |
| ZNF555 | 3 |
| ZNF560 | 3 |
| ZNF573 | 3 |
| ZNF583 | 3 |
| ZNF676 | 3 |
| ZNF711 | 3 |
| ZNF730 | 3 |
| ZNF736 | 3 |
| ZNF804A | 3 |
| ZNF827 | 3 |
| ZNF93 | 3 |
| ZNF99 | 3 |
| A1CF | 2 |
| AARS2 | 2 |
| ABCA6 | 2 |
| ABCA8 | 2 |
| ABCB1 | 2 |
| ABCB4 | 2 |
| ABCC1 | 2 |
| ABCC11 | 2 |
| ABCC12 | 2 |
| ABCC3 | 2 |
| ABCD3 | 2 |
| ABHD3 | 2 |
| ABI2 | 2 |
| ABLIM1 | 2 |
| ABR | 2 |
| ACACB | 2 |
| ACAN | 2 |
| ACO2 | 2 |
| ACSBG2 | 2 |
| ACTL7A | 2 |
| ACVR1 | 2 |
| ADAD1 | 2 |
| ADAM18 | 2 |
| ADAM21 | 2 |
| ADAM22 | 2 |
| ADAM32 | 2 |
| ADAMTS1 | 2 |
| ADAMTS12 | 2 |
| ADAMTS14 | 2 |
| ADAMTS17 | 2 |
| ADAMTS18 | 2 |
| ADCY8 | 2 |
| ADCYAP1R1 | 2 |
| ADH1B | 2 |
| ADRB2 | 2 |
| ADRBK1 | 2 |
| AFAP1L2 | 2 |
| AFF3 | 2 |
| AGAP2 | 2 |
| AGBL1 | 2 |
| AGFG1 | 2 |
| AGPAT9 | 2 |
| AGTPBP1 | 2 |
| AIFM1 | 2 |
| AIMP2 | 2 |
| AKAP1 | 2 |
| AKAP2 | 2 |
| AKAP3 | 2 |
| AKAP9 | 2 |
| AKNA | 2 |
| ALDH1L1 | 2 |
| ALDOB | 2 |
| ALKBH5 | 2 |
| ALOX5 | 2 |
| AMACR | 2 |
| AMBRA1 | 2 |
| AMOT | 2 |
| AMY2A | 2 |
| ANGPTL3 | 2 |
| ANKFN1 | 2 |
| ANKHD1 | 2 |
| ANKIB1 | 2 |
| ANKK1 | 2 |
| ANKRD12 | 2 |
| ANKRD17 | 2 |
| ANKRD20A4 | 2 |
| ANKRD31 | 2 |
| ANKRD49 | 2 |
| ANKRD50 | 2 |
| ANLN | 2 |
| ANO3 | 2 |
| ANO6 | 2 |
| ANPEP | 2 |
| ANTXR2 | 2 |
| AP1M2 | 2 |
| AP4E1 | 2 |
| APLF | 2 |
| APOH | 2 |
| APOL1 | 2 |
| APPL2 | 2 |
| AQR | 2 |
| ARFGAP3 | 2 |
| ARFIP2 | 2 |
| ARHGAP20 | 2 |
| ARHGAP23 | 2 |
| ARHGAP32 | 2 |
| ARHGAP9 | 2 |
| ARHGEF10L | 2 |
| ARHGEF12 | 2 |
| ARHGEF6 | 2 |
| ARID4A | 2 |
| ARID5B | 2 |
| ARMCX1 | 2 |
| ARNT2 | 2 |
| ARSI | 2 |
| ASB17 | 2 |
| ASPH | 2 |
| ASZ1 | 2 |
| ATAD1 | 2 |
| ATAD2B | 2 |
| ATG10 | 2 |
| ATL1 | 2 |
| ATP10D | 2 |
| ATP13A4 | 2 |
| ATP2A1 | 2 |
| ATP2A2 | 2 |
| ATP2A3 | 2 |
| ATP6V0A2 | 2 |
| ATP7B | 2 |
| ATP8B3 | 2 |
| ATXN1 | 2 |
| ATXN2 | 2 |
| ATXN7L2 | 2 |
| AXIN2 | 2 |
| B3GALNT1 | 2 |
| B4GALNT2 | 2 |
| B4GALNT4 | 2 |
| B4GALT5 | 2 |
| BACE2 | 2 |
| BACH1 | 2 |
| BAHCC1 | 2 |
| BANK1 | 2 |
| BAP1 | 2 |
| BBS2 | 2 |
| BBS9 | 2 |
| BCOR | 2 |
| BCORL1 | 2 |
| BEND2 | 2 |
| BEST1 | 2 |
| BEST3 | 2 |
| BICD1 | 2 |
| BIN1 | 2 |
| BLZF1 | 2 |
| BMP3 | 2 |
| BNIP1 | 2 |
| BRCA1 | 2 |
| BRD8 | 2 |
| BRDT | 2 |
| BRF2 | 2 |
| BRIP1 | 2 |
| BRSK1 | 2 |
| BRWD1 | 2 |
| BTG4 | 2 |
| BTNL9 | 2 |
| BUB1B | 2 |
| C10orf107 | 2 |
| C10orf118 | 2 |
| C11orf63 | 2 |
| C11orf70 | 2 |
| C12orf4 | 2 |
| C12orf40 | 2 |
| C12orf45 | 2 |
| C12orf56 | 2 |
| C14orf166B | 2 |
| C14orf176 | 2 |
| C15orf55 | 2 |
| C17orf104 | 2 |
| C17orf97 | 2 |
| C18orf8 | 2 |
| C19orf44 | 2 |
| C19orf45 | 2 |
| C1orf101 | 2 |
| C1orf68 | 2 |
| C1orf94 | 2 |
| C2CD3 | 2 |
| C2orf62 | 2 |
| C2orf81 | 2 |
| C3orf30 | 2 |
| C5 | 2 |
| C5orf20 | 2 |
| C5orf34 | 2 |
| C6orf10 | 2 |
| C6orf225 | 2 |
| C6orf58 | 2 |
| C9 | 2 |
| C9orf84 | 2 |
| CACNA1D | 2 |
| CACNA2D2 | 2 |
| CAD | 2 |
| CADM2 | 2 |
| CALD1 | 2 |
| CALML4 | 2 |
| CALN1 | 2 |
| CAMKK2 | 2 |
| CAMTA2 | 2 |
| CAPN1 | 2 |
| CAPN14 | 2 |
| CAPN9 | 2 |
| CARD11 | 2 |
| CARD17 | 2 |
| CARD6 | 2 |
| CASC1 | 2 |
| CASC5 | 2 |
| CASK | 2 |
| CATSPER1 | 2 |
| CATSPER4 | 2 |
| CBLB | 2 |
| CBLN2 | 2 |
| CCBE1 | 2 |
| CCDC108 | 2 |
| CCDC136 | 2 |
| CCDC14 | 2 |
| CCDC146 | 2 |
| CCDC150 | 2 |
| CCDC157 | 2 |
| CCDC168 | 2 |
| CCDC18 | 2 |
| CCDC80 | 2 |
| CCDC82 | 2 |
| CCDC88B | 2 |
| CCDC99 | 2 |
| CCKAR | 2 |
| CCNA1 | 2 |
| CCNB3 | 2 |
| CCNO | 2 |
| CCNYL3 | 2 |
| CCR2 | 2 |
| CCR6 | 2 |
| CCT6A | 2 |
| CD207 | 2 |
| CD22 | 2 |
| CD97 | 2 |
| CDH13 | 2 |
| CDK5RAP1 | 2 |
| CDK9 | 2 |
| CDKL5 | 2 |
| CDYL | 2 |
| CEACAM6 | 2 |
| CECR2 | 2 |
| CELA1 | 2 |
| CELSR1 | 2 |
| CELSR2 | 2 |
| CELSR3 | 2 |
| CENPB | 2 |
| CENPC1 | 2 |
| CEP135 | 2 |
| CEP192 | 2 |
| CEP250 | 2 |
| CFH | 2 |
| CFHR3 | 2 |
| CHAF1A | 2 |
| CHD1 | 2 |
| CHD2 | 2 |
| CHD6 | 2 |
| CHD7 | 2 |
| CHD8 | 2 |
| CHRM1 | 2 |
| CHRNA10 | 2 |
| CHRNB4 | 2 |
| CLCN4 | 2 |
| CLEC1A | 2 |
| CLGN | 2 |
| CLIP3 | 2 |
| CLK1 | 2 |
| CLOCK | 2 |
| CLVS1 | 2 |
| CMAS | 2 |
| CMPK2 | 2 |
| CNKSR3 | 2 |
| CNNM2 | 2 |
| CNOT1 | 2 |
| CNOT2 | 2 |
| CNOT3 | 2 |
| CNRIP1 | 2 |
| CNTN5 | 2 |
| CNTNAP4 | 2 |
| CNTRL | 2 |
| COBLL1 | 2 |
| COCH | 2 |
| COG7 | 2 |
| COL10A1 | 2 |
| COL16A1 | 2 |
| COL17A1 | 2 |
| COL1A2 | 2 |
| COL27A1 | 2 |
| COL2A1 | 2 |
| COL4A1 | 2 |
| COL4A4 | 2 |
| COL6A3 | 2 |
| COL6A6 | 2 |
| COPS5 | 2 |
| COTL1 | 2 |
| CPNE5 | 2 |
| CPS1 | 2 |
| CPSF2 | 2 |
| CPSF3 | 2 |
| CPT1C | 2 |
| CR1 | 2 |
| CREBBP | 2 |
| CRISP1 | 2 |
| CSF1R | 2 |
| CSF2RB | 2 |
| CSNK1D | 2 |
| CSNK1G3 | 2 |
| CSPP1 | 2 |
| CTNNA2 | 2 |
| CTNNA3 | 2 |
| CTNND2 | 2 |
| CTTNBP2 | 2 |
| CUL4A | 2 |
| CXADR | 2 |
| CXCL12 | 2 |
| CXCR1 | 2 |
| CXorf30 | 2 |
| CYFIP1 | 2 |
| CYP2A7 | 2 |
| CYP2E1 | 2 |
| CYP2G1P | 2 |
| CYP3A5 | 2 |
| DACT1 | 2 |
| DAGLA | 2 |
| DAPP1 | 2 |
| DCAF17 | 2 |
| DCAF5 | 2 |
| DCAF8L1 | 2 |
| DCBLD2 | 2 |
| DCDC5 | 2 |
| DCN | 2 |
| DCUN1D1 | 2 |
| DCX | 2 |
| DDI1 | 2 |
| DDX1 | 2 |
| DDX59 | 2 |
| DENND3 | 2 |
| DENND5A | 2 |
| DENND5B | 2 |
| DGKZ | 2 |
| DHCR7 | 2 |
| DHX15 | 2 |
| DHX37 | 2 |
| DHX9 | 2 |
| DICER1 | 2 |
| DIRAS2 | 2 |
| DLG1 | 2 |
| DMBT1 | 2 |
| DMC1 | 2 |
| DMRT2 | 2 |
| DMTF1 | 2 |
| DNA2 | 2 |
| DNAH1 | 2 |
| DNAI2 | 2 |
| DNAJC13 | 2 |
| DNAJC6 | 2 |
| DNER | 2 |
| DOCK1 | 2 |
| DOCK4 | 2 |
| DOCK7 | 2 |
| DOCK8 | 2 |
| DOCK9 | 2 |
| DOK1 | 2 |
| DOK2 | 2 |
| DOPEY2 | 2 |
| DOT1L | 2 |
| DPP10 | 2 |
| DPP4 | 2 |
| DPP8 | 2 |
| DPYSL3 | 2 |
| DSC1 | 2 |
| DSC3 | 2 |
| DSE | 2 |
| DSG4 | 2 |
| DUSP27 | 2 |
| DUSP4 | 2 |
| DYRK1B | 2 |
| DYSF | 2 |
| EDIL3 | 2 |
| EEF2K | 2 |
| EFCAB4B | 2 |
| EFNA5 | 2 |
| EIF3A | 2 |
| EMB | 2 |
| EMID2 | 2 |
| EML5 | 2 |
| ENOX1 | 2 |
| ENTPD2 | 2 |
| EP400 | 2 |
| EPB41L1 | 2 |
| EPB41L5 | 2 |
| EPG5 | 2 |
| EPHX1 | 2 |
| EPPK1 | 2 |
| ERBB2IP | 2 |
| ERC2 | 2 |
| ERCC4 | 2 |
| ERCC5 | 2 |
| ERG | 2 |
| ERN2 | 2 |
| ESCO1 | 2 |
| ESRRA | 2 |
| ESRRG | 2 |
| ETAA1 | 2 |
| ETFDH | 2 |
| ETV6 | 2 |
| EVC | 2 |
| EVX2 | 2 |
| EXOC2 | 2 |
| EXOC5 | 2 |
| EXOC6B | 2 |
| F13B | 2 |
| F2R | 2 |
| F8 | 2 |
| FAM107B | 2 |
| FAM111B | 2 |
| FAM120B | 2 |
| FAM129C | 2 |
| FAM13A | 2 |
| FAM13B | 2 |
| FAM160A1 | 2 |
| FAM160A2 | 2 |
| FAM175A | 2 |
| FAM179B | 2 |
| FAM186A | 2 |
| FAM188B | 2 |
| FAM189A2 | 2 |
| FAM190A | 2 |
| FAM193A | 2 |
| FAM208B | 2 |
| FAM214B | 2 |
| FAM5B | 2 |
| FAM65C | 2 |
| FAM69A | 2 |
| FAM75C2 | 2 |
| FAM83B | 2 |
| FANCL | 2 |
| FASTKD3 | 2 |
| FBN3 | 2 |
| FBP1 | 2 |
| FBXL4 | 2 |
| FBXL7 | 2 |
| FBXO25 | 2 |
| FBXO42 | 2 |
| FBXO43 | 2 |
| FBXO7 | 2 |
| FCAMR | 2 |
| FCGBP | 2 |
| FCRL1 | 2 |
| FERMT2 | 2 |
| FFAR2 | 2 |
| FGFR2 | 2 |
| FGR | 2 |
| FHOD3 | 2 |
| FKBP10 | 2 |
| FLG2 | 2 |
| FLNC | 2 |
| FLT1 | 2 |
| FMNL2 | 2 |
| FMO1 | 2 |
| FNBP1L | 2 |
| FNBP4 | 2 |
| FNDC3B | 2 |
| FNIP2 | 2 |
| FOXK2 | 2 |
| FOXRED1 | 2 |
| FREM3 | 2 |
| FRK | 2 |
| FRMD4B | 2 |
| FSHR | 2 |
| FST | 2 |
| FURIN | 2 |
| FUT11 | 2 |
| FXR1 | 2 |
| FZR1 | 2 |
| GABRA6 | 2 |
| GABRR3 | 2 |
| GALE | 2 |
| GALNS | 2 |
| GALNT13 | 2 |
| GALNT14 | 2 |
| GALNTL5 | 2 |
| GALNTL6 | 2 |
| GARS | 2 |
| GAS7 | 2 |
| GBA2 | 2 |
| GCAT | 2 |
| GCFC1 | 2 |
| GCNT1 | 2 |
| GCNT4 | 2 |
| GFAP | 2 |
| GGH | 2 |
| GGNBP2 | 2 |
| GLDN | 2 |
| GLIPR1L2 | 2 |
| GLT6D1 | 2 |
| GLT8D2 | 2 |
| GNAL | 2 |
| GNAS | 2 |
| GOLGA2 | 2 |
| GOLGA4 | 2 |
| GON4L | 2 |
| GPHN | 2 |
| GPR111 | 2 |
| GPR115 | 2 |
| GPR128 | 2 |
| GPR133 | 2 |
| GPR176 | 2 |
| GPR179 | 2 |
| GPR18 | 2 |
| GPRC6A | 2 |
| GPRIN1 | 2 |
| GPS1 | 2 |
| GRAMD1B | 2 |
| GRB7 | 2 |
| GRIA2 | 2 |
| GRIK5 | 2 |
| GRIPAP1 | 2 |
| GRM1 | 2 |
| GRM8 | 2 |
| GSDMC | 2 |
| GSTM4 | 2 |
| GTF2H3 | 2 |
| GTF3C1 | 2 |
| GTPBP1 | 2 |
| GTPBP8 | 2 |
| GUCA1C | 2 |
| GUCY1A3 | 2 |
| GUCY2C | 2 |
| GUCY2F | 2 |
| H2AFY2 | 2 |
| HADHA | 2 |
| HAPLN1 | 2 |
| HAVCR1 | 2 |
| HCN4 | 2 |
| HCRTR2 | 2 |
| HDAC9 | 2 |
| HEATR5B | 2 |
| HECTD1 | 2 |
| HECW1 | 2 |
| HEG1 | 2 |
| HERC4 | 2 |
| HERC5 | 2 |
| HFE | 2 |
| HGD | 2 |
| HHLA1 | 2 |
| HHLA2 | 2 |
| HIF1AN | 2 |
| HIF3A | 2 |
| HIPK1 | 2 |
| HIPK2 | 2 |
| HIPK3 | 2 |
| HIST1H2BD | 2 |
| HIST1H3D | 2 |
| HIST1H4D | 2 |
| HIVEP1 | 2 |
| HK2 | 2 |
| HLA-A | 2 |
| HLCS | 2 |
| HMGCR | 2 |
| HMGCS1 | 2 |
| HMGXB4 | 2 |
| HNRNPU | 2 |
| HOMER1 | 2 |
| HOOK2 | 2 |
| HOXA1 | 2 |
| HPS1 | 2 |
| HPSE2 | 2 |
| HRAS | 2 |
| HRC | 2 |
| HRNR | 2 |
| HS6ST3 | 2 |
| HSPA8 | 2 |
| HSPB7 | 2 |
| HTR2B | 2 |
| HUNK | 2 |
| HUS1 | 2 |
| HYAL2 | 2 |
| IARS2 | 2 |
| IDE | 2 |
| IDH1 | 2 |
| IFNAR1 | 2 |
| IFT88 | 2 |
| IGFN1 | 2 |
| IGHMBP2 | 2 |
| IGSF3 | 2 |
| IKBIP | 2 |
| IKBKAP | 2 |
| IKZF3 | 2 |
| IL12RB2 | 2 |
| IL13RA2 | 2 |
| INCENP | 2 |
| ING3 | 2 |
| INPP5B | 2 |
| INPP5F | 2 |
| INSL4 | 2 |
| INVS | 2 |
| IQCB1 | 2 |
| IRGC | 2 |
| IRS4 | 2 |
| ITFG1 | 2 |
| ITGA2 | 2 |
| ITGA6 | 2 |
| ITGAM | 2 |
| ITGB1 | 2 |
| ITGB4 | 2 |
| ITIH2 | 2 |
| ITIH3 | 2 |
| ITIH4 | 2 |
| ITIH5 | 2 |
| ITIH6 | 2 |
| JMJD4 | 2 |
| KAT7 | 2 |
| KATNAL2 | 2 |
| KBTBD6 | 2 |
| KCNA10 | 2 |
| KCNA2 | 2 |
| KCNA5 | 2 |
| KCNB1 | 2 |
| KCND2 | 2 |
| KCNG1 | 2 |
| KCNH7 | 2 |
| KCNJ5 | 2 |
| KCNK18 | 2 |
| KCNQ3 | 2 |
| KCNQ5 | 2 |
| KCNRG | 2 |
| KCNS3 | 2 |
| KCTD16 | 2 |
| KCTD18 | 2 |
| KCTD3 | 2 |
| KDELR1 | 2 |
| KDELR2 | 2 |
| KDM2A | 2 |
| KDM5A | 2 |
| KDM5B | 2 |
| KHDRBS2 | 2 |
| KIAA0182 | 2 |
| KIAA0196 | 2 |
| KIAA0232 | 2 |
| KIAA0586 | 2 |
| KIAA0754 | 2 |
| KIAA0907 | 2 |
| KIAA1009 | 2 |
| KIAA1217 | 2 |
| KIAA1239 | 2 |
| KIAA1324L | 2 |
| KIAA1377 | 2 |
| KIAA1407 | 2 |
| KIAA1467 | 2 |
| KIAA1586 | 2 |
| KIAA1614 | 2 |
| KIAA1919 | 2 |
| KIAA2022 | 2 |
| KIF15 | 2 |
| KIF17 | 2 |
| KIF19 | 2 |
| KIF20A | 2 |
| KIF21B | 2 |
| KIF23 | 2 |
| KIF24 | 2 |
| KIF26B | 2 |
| KIF4B | 2 |
| KIRREL2 | 2 |
| KLF6 | 2 |
| KLHDC10 | 2 |
| KLHL13 | 2 |
| KLHL20 | 2 |
| KLHL23 | 2 |
| KLHL6 | 2 |
| KLK15 | 2 |
| KLK7 | 2 |
| KLKB1 | 2 |
| KLRC2 | 2 |
| KPNA2 | 2 |
| KRT17 | 2 |
| KRT20 | 2 |
| KRT3 | 2 |
| KRT5 | 2 |
| KRT73 | 2 |
| KRT74 | 2 |
| KSR2 | 2 |
| KYNU | 2 |
| L3MBTL3 | 2 |
| LAMA4 | 2 |
| LAMB3 | 2 |
| LAMC1 | 2 |
| LAMC3 | 2 |
| LANCL1 | 2 |
| LANCL3 | 2 |
| LAPTM4A | 2 |
| LCMT2 | 2 |
| LEPREL1 | 2 |
| LHX9 | 2 |
| LILRA1 | 2 |
| LILRB2 | 2 |
| LIMK2 | 2 |
| LIN54 | 2 |
| LIN9 | 2 |
| LIPC | 2 |
| LIPE | 2 |
| LMBRD1 | 2 |
| LMBRD2 | 2 |
| LMLN | 2 |
| LMOD1 | 2 |
| LOXHD1 | 2 |
| LPAR1 | 2 |
| LRCH3 | 2 |
| LRIG3 | 2 |
| LRIT2 | 2 |
| LRMP | 2 |
| LRRC1 | 2 |
| LRRC17 | 2 |
| LRRC18 | 2 |
| LRRC3B | 2 |
| LRRC40 | 2 |
| LRRC42 | 2 |
| LRRC7 | 2 |
| LRRN1 | 2 |
| LRRN2 | 2 |
| LRRTM4 | 2 |
| LTBR | 2 |
| LY75 | 2 |
| MACC1 | 2 |
| MAD2L2 | 2 |
| MADD | 2 |
| MAGEB16 | 2 |
| MAML2 | 2 |
| MAP3K13 | 2 |
| MAP3K14 | 2 |
| MAP3K9 | 2 |
| MAP4K2 | 2 |
| MAP9 | 2 |
| MAPK8 | 2 |
| MARK3 | 2 |
| MARS | 2 |
| MASP1 | 2 |
| MBL2 | 2 |
| MBTPS2 | 2 |
| MCC | 2 |
| MCF2L2 | 2 |
| MCM4 | 2 |
| MCM6 | 2 |
| MCM9 | 2 |
| MCOLN2 | 2 |
| MCPH1 | 2 |
| MDM2 | 2 |
| ME1 | 2 |
| MED13L | 2 |
| MEF2C | 2 |
| MEFV | 2 |
| MEIS1 | 2 |
| MFSD9 | 2 |
| MGAT4A | 2 |
| MGAT4C | 2 |
| MGEA5 | 2 |
| MIB1 | 2 |
| MICAL1 | 2 |
| MICAL3 | 2 |
| MKNK1 | 2 |
| MLH3 | 2 |
| MLIP | 2 |
| MLL5 | 2 |
| MLLT1 | 2 |
| MMEL1 | 2 |
| MMP10 | 2 |
| MMP16 | 2 |
| MMRN2 | 2 |
| MNDA | 2 |
| MOCS1 | 2 |
| MOCS3 | 2 |
| MON2 | 2 |
| MOSPD2 | 2 |
| MOV10 | 2 |
| MRE11A | 2 |
| MRGPRX1 | 2 |
| MRTO4 | 2 |
| MS4A12 | 2 |
| MSL3 | 2 |
| MTM1 | 2 |
| MTMR11 | 2 |
| MTPAP | 2 |
| MUC5B | 2 |
| MVK | 2 |
| MYB | 2 |
| MYBPC1 | 2 |
| MYCN | 2 |
| MYH9 | 2 |
| MYO1C | 2 |
| MYO1G | 2 |
| MYO3A | 2 |
| MYO5C | 2 |
| MYOF | 2 |
| MYOM2 | 2 |
| MYT1 | 2 |
| MYT1L | 2 |
| N4BP2 | 2 |
| N6AMT1 | 2 |
| NAA25 | 2 |
| NAPEPLD | 2 |
| NARS | 2 |
| NASP | 2 |
| NAT10 | 2 |
| NAV1 | 2 |
| NCAM1 | 2 |
| NCKAP5L | 2 |
| NCOR1 | 2 |
| NCR1 | 2 |
| NCS1 | 2 |
| NDC80 | 2 |
| NDUFA12 | 2 |
| NEDD9 | 2 |
| NEIL3 | 2 |
| NEK10 | 2 |
| NEURL4 | 2 |
| NEUROG1 | 2 |
| NFATC4 | 2 |
| NFKBIZ | 2 |
| NFX1 | 2 |
| NFXL1 | 2 |
| NHLRC3 | 2 |
| NHS | 2 |
| NHSL1 | 2 |
| NINL | 2 |
| NIPBL | 2 |
| NISCH | 2 |
| NIT2 | 2 |
| NKG7 | 2 |
| NLRC4 | 2 |
| NLRC5 | 2 |
| NLRP5 | 2 |
| NOBOX | 2 |
| NOS3 | 2 |
| NOVA1 | 2 |
| NOX1 | 2 |
| NOX5 | 2 |
| NPAT | 2 |
| NPHS1 | 2 |
| NPTX1 | 2 |
| NR1D2 | 2 |
| NR2F1 | 2 |
| NSFL1C | 2 |
| NT5DC1 | 2 |
| NUDT16 | 2 |
| NUFIP2 | 2 |
| NUMA1 | 2 |
| NUP107 | 2 |
| NUP155 | 2 |
| NUP205 | 2 |
| NUPL1 | 2 |
| OAS1 | 2 |
| OCLN | 2 |
| ODF1 | 2 |
| OLFM3 | 2 |
| OLFM4 | 2 |
| OLFML1 | 2 |
| OR10A3 | 2 |
| OR10A4 | 2 |
| OR10AB1P | 2 |
| OR10C1 | 2 |
| OR10H3 | 2 |
| OR10R2 | 2 |
| OR14A16 | 2 |
| OR14C36 | 2 |
| OR2A12 | 2 |
| OR2K2 | 2 |
| OR4C16 | 2 |
| OR4C45 | 2 |
| OR4E2 | 2 |
| OR4Q3 | 2 |
| OR51A4 | 2 |
| OR51G1 | 2 |
| OR52N2 | 2 |
| OR52N4 | 2 |
| OR5B12 | 2 |
| OR5D13 | 2 |
| OR5F1 | 2 |
| OR5H1 | 2 |
| OR5L1 | 2 |
| OR5R1 | 2 |
| OR6C75 | 2 |
| OR6N2 | 2 |
| OR7C2 | 2 |
| OR8B12 | 2 |
| OR9I1 | 2 |
| ORC2 | 2 |
| OSBPL3 | 2 |
| OSBPL8 | 2 |
| OSBPL9 | 2 |
| OSMR | 2 |
| OTOF | 2 |
| OTOG | 2 |
| OTOR | 2 |
| OTUD1 | 2 |
| OVCH1 | 2 |
| P4HA3 | 2 |
| PABPC1 | 2 |
| PABPC4 | 2 |
| PADI2 | 2 |
| PAH | 2 |
| PALLD | 2 |
| PANK4 | 2 |
| PAPPA2 | 2 |
| PARD3 | 2 |
| PARD3B | 2 |
| PARL | 2 |
| PARP4 | 2 |
| PAX8 | 2 |
| PCDH11X | 2 |
| PCDH12 | 2 |
| PCDH19 | 2 |
| PCDHAC2 | 2 |
| PCDHB1 | 2 |
| PCDHB13 | 2 |
| PCDHB3 | 2 |
| PCDHB8 | 2 |
| PCNT | 2 |
| PDE12 | 2 |
| PDE1A | 2 |
| PDE4B | 2 |
| PDE4D | 2 |
| PDE5A | 2 |
| PDE6C | 2 |
| PDIA5 | 2 |
| PDILT | 2 |
| PDLIM1 | 2 |
| PDPN | 2 |
| PDZD9 | 2 |
| PEG3 | 2 |
| PER3 | 2 |
| PEX11B | 2 |
| PFAS | 2 |
| PFKFB4 | 2 |
| PFKL | 2 |
| PFKM | 2 |
| PGM2L1 | 2 |
| PHACTR3 | 2 |
| PHC1 | 2 |
| PHC2 | 2 |
| PHEX | 2 |
| PHIP | 2 |
| PHKA1 | 2 |
| PHLDB1 | 2 |
| PHLPP2 | 2 |
| PHYHIPL | 2 |
| PI4K2B | 2 |
| PI4KB | 2 |
| PICALM | 2 |
| PIK3C2A | 2 |
| PIK3C2G | 2 |
| PIK3C3 | 2 |
| PIK3R1 | 2 |
| PINK1 | 2 |
| PIWIL1 | 2 |
| PJA2 | 2 |
| PKD1L2 | 2 |
| PKD2 | 2 |
| PKDCC | 2 |
| PKDREJ | 2 |
| PKP1 | 2 |
| PLA2G4F | 2 |
| PLAGL2 | 2 |
| PLAU | 2 |
| PLB1 | 2 |
| PLCG1 | 2 |
| PLEC | 2 |
| PLEKHA5 | 2 |
| PLEKHG5 | 2 |
| PLEKHM3 | 2 |
| PLS1 | 2 |
| PLXDC1 | 2 |
| PLXDC2 | 2 |
| PLXNA2 | 2 |
| PLXNA3 | 2 |
| PLXNB2 | 2 |
| PMPCA | 2 |
| PNLIP | 2 |
| PNLIPRP1 | 2 |
| PNMAL1 | 2 |
| PNN | 2 |
| POC1A | 2 |
| POF1B | 2 |
| POLA1 | 2 |
| POLB | 2 |
| POLN | 2 |
| POLR3GL | 2 |
| POU2F1 | 2 |
| POU6F2 | 2 |
| PPIG | 2 |
| PPM1A | 2 |
| PPM1K | 2 |
| PPP2CB | 2 |
| PPP2R1B | 2 |
| PPP2R2D | 2 |
| PPP6R2 | 2 |
| PRDM1 | 2 |
| PRDM10 | 2 |
| PRDM16 | 2 |
| PRIC285 | 2 |
| PRICKLE1 | 2 |
| PRKACB | 2 |
| PRKAG2 | 2 |
| PRKCB | 2 |
| PRKCH | 2 |
| PRKCQ | 2 |
| PRMT7 | 2 |
| PROKR2 | 2 |
| PROM2 | 2 |
| PROS1 | 2 |
| PRPF39 | 2 |
| PRR12 | 2 |
| PRSS1 | 2 |
| PRSS38 | 2 |
| PRSS46 | 2 |
| PRSS55 | 2 |
| PRTFDC1 | 2 |
| PSD2 | 2 |
| PSD3 | 2 |
| PSG2 | 2 |
| PSG9 | 2 |
| PSMD2 | 2 |
| PTCHD3 | 2 |
| PTK2 | 2 |
| PTK2B | 2 |
| PTK7 | 2 |
| PTPLA | 2 |
| PTPN13 | 2 |
| PTPN2 | 2 |
| PTPN21 | 2 |
| PTPRC | 2 |
| PTPRF | 2 |
| PTPRS | 2 |
| PUM1 | 2 |
| PVRL1 | 2 |
| PWWP2A | 2 |
| PYGL | 2 |
| PYGO2 | 2 |
| PYHIN1 | 2 |
| PZP | 2 |
| RAB3GAP1 | 2 |
| RAD50 | 2 |
| RAD51AP2 | 2 |
| RAD52 | 2 |
| RAD54L2 | 2 |
| RAF1 | 2 |
| RAG2 | 2 |
| RAI14 | 2 |
| RALGPS2 | 2 |
| RANBP17 | 2 |
| RAP1GAP | 2 |
| RAPGEF6 | 2 |
| RARS2 | 2 |
| RASGRF2 | 2 |
| RASGRP3 | 2 |
| RBBP6 | 2 |
| RBFOX1 | 2 |
| RBL1 | 2 |
| RBL2 | 2 |
| RBM14 | 2 |
| RBM15 | 2 |
| RBM41 | 2 |
| RBM47 | 2 |
| RBMX | 2 |
| RBMX2 | 2 |
| RGS9 | 2 |
| RHOT1 | 2 |
| RIC8B | 2 |
| RIF1 | 2 |
| RINT1 | 2 |
| RNASEH2B | 2 |
| RNF10 | 2 |
| RNF213 | 2 |
| RNF214 | 2 |
| RNF219 | 2 |
| RNF40 | 2 |
| RNF43 | 2 |
| ROBO3 | 2 |
| ROR2 | 2 |
| RORA | 2 |
| RORB | 2 |
| RPH3A | 2 |
| RPL36A | 2 |
| RPRD2 | 2 |
| RPS6KA5 | 2 |
| RPS6KL1 | 2 |
| RPUSD2 | 2 |
| RRP12 | 2 |
| RRP9 | 2 |
| RSF1 | 2 |
| RTN4 | 2 |
| RUNX2 | 2 |
| RUSC1 | 2 |
| RXFP1 | 2 |
| RYK | 2 |
| S1PR1 | 2 |
| SAFB | 2 |
| SAMD9 | 2 |
| SAMM50 | 2 |
| SBF1 | 2 |
| SCAF11 | 2 |
| SCFD2 | 2 |
| SCML2 | 2 |
| SCN2A | 2 |
| SCN9A | 2 |
| SCNN1A | 2 |
| SCNN1B | 2 |
| SCUBE1 | 2 |
| SDF2 | 2 |
| SDK2 | 2 |
| SDPR | 2 |
| SDR16C5 | 2 |
| SDR9C7 | 2 |
| SEC13 | 2 |
| SEC24A | 2 |
| SEC24C | 2 |
| SECISBP2 | 2 |
| SELL | 2 |
| SEMA3A | 2 |
| SEMA4G | 2 |
| SEMA6C | 2 |
| SEMA6D | 2 |
| SENP1 | 2 |
| SENP5 | 2 |
| SENP7 | 2 |
| 14-Sep | 2 |
| 6-Sep | 2 |
| SERPINB2 | 2 |
| SERPINC1 | 2 |
| SETD5 | 2 |
| SETDB2 | 2 |
| SEZ6L | 2 |
| SF3A3 | 2 |
| SF3B2 | 2 |
| SFMBT1 | 2 |
| SFRP1 | 2 |
| SGCG | 2 |
| SGSM1 | 2 |
| SGTA | 2 |
| SH2D4A | 2 |
| SHANK1 | 2 |
| SHANK2 | 2 |
| SHISA2 | 2 |
| SHPRH | 2 |
| SHROOM4 | 2 |
| SI | 2 |
| SIDT1 | 2 |
| SIGLEC1 | 2 |
| SLAMF1 | 2 |
| SLC12A1 | 2 |
| SLC12A5 | 2 |
| SLC13A5 | 2 |
| SLC16A1 | 2 |
| SLC16A13 | 2 |
| SLC16A7 | 2 |
| SLC17A6 | 2 |
| SLC1A3 | 2 |
| SLC22A6 | 2 |
| SLC23A1 | 2 |
| SLC25A13 | 2 |
| SLC25A5 | 2 |
| SLC26A4 | 2 |
| SLC2A12 | 2 |
| SLC30A1 | 2 |
| SLC30A8 | 2 |
| SLC33A1 | 2 |
| SLC35A5 | 2 |
| SLC35B3 | 2 |
| SLC35C1 | 2 |
| SLC35E1 | 2 |
| SLC36A3 | 2 |
| SLC39A11 | 2 |
| SLC43A1 | 2 |
| SLC45A1 | 2 |
| SLC45A3 | 2 |
| SLC4A4 | 2 |
| SLC4A5 | 2 |
| SLC4A8 | 2 |
| SLC5A4 | 2 |
| SLC6A1 | 2 |
| SLC6A16 | 2 |
| SLC6A19 | 2 |
| SLC6A20 | 2 |
| SLC7A14 | 2 |
| SLC8A2 | 2 |
| SLC9A2 | 2 |
| SLC9A4 | 2 |
| SLC9A5 | 2 |
| SLCO1B1 | 2 |
| SLCO1B3 | 2 |
| SLCO2B1 | 2 |
| SLIT3 | 2 |
| SLITRK2 | 2 |
| SLTM | 2 |
| SMAD3 | 2 |
| SMARCA1 | 2 |
| SMARCA2 | 2 |
| SMARCAD1 | 2 |
| SMEK2 | 2 |
| SMG7 | 2 |
| SNAP91 | 2 |
| SNCA | 2 |
| SNED1 | 2 |
| SNRK | 2 |
| SNTG2 | 2 |
| SNX19 | 2 |
| SOAT2 | 2 |
| SOCS4 | 2 |
| SON | 2 |
| SORBS2 | 2 |
| SOS2 | 2 |
| SOX5 | 2 |
| SP110 | 2 |
| SP140 | 2 |
| SP6 | 2 |
| SPAG9 | 2 |
| SPAM1 | 2 |
| SPATA17 | 2 |
| SPATS2 | 2 |
| SPATS2L | 2 |
| SPECC1 | 2 |
| SPEF2 | 2 |
| SPOCK1 | 2 |
| SPRED2 | 2 |
| SPRR1A | 2 |
| SPTB | 2 |
| SPTBN1 | 2 |
| SQSTM1 | 2 |
| SREBF1 | 2 |
| SREBF2 | 2 |
| SRPR | 2 |
| SS18L1 | 2 |
| SSFA2 | 2 |
| SSPN | 2 |
| SSPO | 2 |
| ST5 | 2 |
| ST6GALNAC5 | 2 |
| ST7 | 2 |
| ST8SIA1 | 2 |
| ST8SIA6 | 2 |
| STAB1 | 2 |
| STAG1 | 2 |
| STAM2 | 2 |
| STAP1 | 2 |
| STAU2 | 2 |
| STEAP2 | 2 |
| STIP1 | 2 |
| STK35 | 2 |
| STK39 | 2 |
| STRADA | 2 |
| STRBP | 2 |
| STXBP3 | 2 |
| SUGP2 | 2 |
| SULF1 | 2 |
| SUPV3L1 | 2 |
| SUSD4 | 2 |
| SVIL | 2 |
| SWT1 | 2 |
| SYDE2 | 2 |
| SYK | 2 |
| SYNJ1 | 2 |
| SYNJ2 | 2 |
| SYPL2 | 2 |
| SYT10 | 2 |
| SYTL2 | 2 |
| SYTL5 | 2 |
| TAAR9 | 2 |
| TANC2 | 2 |
| TAP1 | 2 |
| TAS1R2 | 2 |
| TAS2R10 | 2 |
| TAS2R9 | 2 |
| TAT | 2 |
| TBC1D15 | 2 |
| TBC1D23 | 2 |
| TBC1D8B | 2 |
| TBK1 | 2 |
| TBX22 | 2 |
| TCERG1 | 2 |
| TCHHL1 | 2 |
| TCP11L1 | 2 |
| TEX2 | 2 |
| TFIP11 | 2 |
| TFR2 | 2 |
| TG | 2 |
| TGFBR1 | 2 |
| TGIF1 | 2 |
| THBS3 | 2 |
| THBS4 | 2 |
| THOC1 | 2 |
| THUMPD3 | 2 |
| TIAL1 | 2 |
| TIGD2 | 2 |
| TJP1 | 2 |
| TLE1 | 2 |
| TLE4 | 2 |
| TLK1 | 2 |
| TLR4 | 2 |
| TLR7 | 2 |
| TLX2 | 2 |
| TM2D3 | 2 |
| TMC3 | 2 |
| TMC7 | 2 |
| TMEFF2 | 2 |
| TMEM131 | 2 |
| TMEM132B | 2 |
| TMEM132D | 2 |
| TMEM161B | 2 |
| TMEM176A | 2 |
| TMEM179B | 2 |
| TMEM181 | 2 |
| TMEM232 | 2 |
| TMEM39A | 2 |
| TMEM41A | 2 |
| TMEM45A | 2 |
| TMEM5 | 2 |
| TMEM67 | 2 |
| TMEM87A | 2 |
| TMEM8A | 2 |
| TMEM8C | 2 |
| TMLHE | 2 |
| TMPRSS11F | 2 |
| TMPRSS15 | 2 |
| TMTC2 | 2 |
| TMTC3 | 2 |
| TMTC4 | 2 |
| TNFRSF10B | 2 |
| TNFRSF19 | 2 |
| TNFRSF1B | 2 |
| TNFRSF21 | 2 |
| TNKS | 2 |
| TNPO1 | 2 |
| TNS1 | 2 |
| TNS3 | 2 |
| TOMM40L | 2 |
| TP63 | 2 |
| TPD52L3 | 2 |
| TRABD | 2 |
| TRAF6 | 2 |
| TRDN | 2 |
| TRIM10 | 2 |
| TRIM25 | 2 |
| TRIM33 | 2 |
| TRIM49 | 2 |
| TRIM68 | 2 |
| TRIM9 | 2 |
| TRIP6 | 2 |
| TRIT1 | 2 |
| TRMT5 | 2 |
| TRO | 2 |
| TRPM2 | 2 |
| TRPM3 | 2 |
| TRPM6 | 2 |
| TRPM8 | 2 |
| TRPV1 | 2 |
| TSHZ3 | 2 |
| TSKS | 2 |
| TSPAN17 | 2 |
| TSR1 | 2 |
| TTC14 | 2 |
| TTC18 | 2 |
| TTC21B | 2 |
| TTC39B | 2 |
| TTC40 | 2 |
| TTF2 | 2 |
| TTLL1 | 2 |
| TTLL2 | 2 |
| TTYH3 | 2 |
| TUBA1C | 2 |
| TUT1 | 2 |
| TYMS | 2 |
| TYRP1 | 2 |
| TYSND1 | 2 |
| TYW5 | 2 |
| U2AF1 | 2 |
| UBAC1 | 2 |
| UBE2NL | 2 |
| UBE3C | 2 |
| UBXN4 | 2 |
| UGT8 | 2 |
| UNC45B | 2 |
| UNC80 | 2 |
| USH1C | 2 |
| USP15 | 2 |
| USP26 | 2 |
| USP34 | 2 |
| USP40 | 2 |
| USP47 | 2 |
| USP6 | 2 |
| USP9X | 2 |
| USP9Y | 2 |
| VANGL1 | 2 |
| VANGL2 | 2 |
| VAPA | 2 |
| VAV2 | 2 |
| VCAM1 | 2 |
| VCL | 2 |
| VCPIP1 | 2 |
| VMO1 | 2 |
| VPRBP | 2 |
| VPS13D | 2 |
| VPS8 | 2 |
| VSIG2 | 2 |
| VWA3A | 2 |
| VWA3B | 2 |
| VWA5B1 | 2 |
| WAPAL | 2 |
| WBP4 | 2 |
| WDR12 | 2 |
| WDR26 | 2 |
| WDR27 | 2 |
| WDR3 | 2 |
| WDR36 | 2 |
| WDR41 | 2 |
| WDR49 | 2 |
| WDR60 | 2 |
| WDR72 | 2 |
| WDR75 | 2 |
| WDR93 | 2 |
| WDR96 | 2 |
| WEE1 | 2 |
| WHSC1L1 | 2 |
| WNK1 | 2 |
| WWC2 | 2 |
| XKR4 | 2 |
| XPNPEP2 | 2 |
| XPO1 | 2 |
| XPO5 | 2 |
| XPO6 | 2 |
| XPO7 | 2 |
| XPOT | 2 |
| XRCC5 | 2 |
| XRCC6 | 2 |
| XYLB | 2 |
| XYLT1 | 2 |
| YBX1 | 2 |
| YEATS2 | 2 |
| YES1 | 2 |
| YLPM1 | 2 |
| YWHAZ | 2 |
| ZBBX | 2 |
| ZBTB10 | 2 |
| ZBTB16 | 2 |
| ZBTB26 | 2 |
| ZBTB46 | 2 |
| ZC3H7A | 2 |
| ZCCHC2 | 2 |
| ZEB2 | 2 |
| ZFP112 | 2 |
| ZFP36L1 | 2 |
| ZFP90 | 2 |
| ZFR | 2 |
| ZFYVE1 | 2 |
| ZFYVE26 | 2 |
| ZFYVE9 | 2 |
| ZHX2 | 2 |
| ZIC1 | 2 |
| ZIC2 | 2 |
| ZIC3 | 2 |
| ZIM2 | 2 |
| ZIM3 | 2 |
| ZMYM2 | 2 |
| ZNF107 | 2 |
| ZNF135 | 2 |
| ZNF14 | 2 |
| ZNF148 | 2 |
| ZNF177 | 2 |
| ZNF213 | 2 |
| ZNF232 | 2 |
| ZNF239 | 2 |
| ZNF250 | 2 |
| ZNF266 | 2 |
| ZNF280B | 2 |
| ZNF318 | 2 |
| ZNF320 | 2 |
| ZNF33A | 2 |
| ZNF347 | 2 |
| ZNF35 | 2 |
| ZNF365 | 2 |
| ZNF383 | 2 |
| ZNF391 | 2 |
| ZNF419 | 2 |
| ZNF431 | 2 |
| ZNF462 | 2 |
| ZNF473 | 2 |
| ZNF488 | 2 |
| ZNF507 | 2 |
| ZNF518B | 2 |
| ZNF519 | 2 |
| ZNF569 | 2 |
| ZNF578 | 2 |
| ZNF595 | 2 |
| ZNF597 | 2 |
| ZNF600 | 2 |
| ZNF605 | 2 |
| ZNF609 | 2 |
| ZNF610 | 2 |
| ZNF615 | 2 |
| ZNF626 | 2 |
| ZNF629 | 2 |
| ZNF677 | 2 |
| ZNF700 | 2 |
| ZNF714 | 2 |
| ZNF774 | 2 |
| ZNF776 | 2 |
| ZNF799 | 2 |
| ZNF816 | 2 |
| ZNF830 | 2 |
| ZNF91 | 2 |
| ZNF92 | 2 |
| ZNRF3 | 2 |
| ZPLD1 | 2 |
| ZSWIM2 | 2 |
| ZUFSP | 2 |
| A2M | 1 |
| AAAS | 1 |
| AADAC | 1 |
| AADAT | 1 |
| AAGAB | 1 |
| AAK1 | 1 |
| AARS | 1 |
| AARSD1 | 1 |
| AASDHPPT | 1 |
| AASS | 1 |
| AATF | 1 |
| ABCA1 | 1 |
| ABCA10 | 1 |
| ABCA2 | 1 |
| ABCA3 | 1 |
| ABCA4 | 1 |
| ABCB10 | 1 |
| ABCB11 | 1 |
| ABCC5 | 1 |
| ABCC6 | 1 |
| ABCC8 | 1 |
| ABCF1 | 1 |
| ABHD1 | 1 |
| ABHD12 | 1 |
| ABHD12B | 1 |
| ABHD4 | 1 |
| ABHD5 | 1 |
| ABHD6 | 1 |
| ABI3BP | 1 |
| ABL2 | 1 |
| ABT1 | 1 |
| ABTB1 | 1 |
| ABTB2 | 1 |
| ACAA1 | 1 |
| ACACA | 1 |
| ACAD10 | 1 |
| ACAD11 | 1 |
| ACAD8 | 1 |
| ACADL | 1 |
| ACADVL | 1 |
| ACAP1 | 1 |
| ACAT1 | 1 |
| ACBD3 | 1 |
| ACBD4 | 1 |
| ACCS | 1 |
| ACCSL | 1 |
| ACE | 1 |
| ACIN1 | 1 |
| ACLY | 1 |
| ACN9 | 1 |
| ACO1 | 1 |
| ACOX1 | 1 |
| ACOX3 | 1 |
| ACPL2 | 1 |
| ACPP | 1 |
| ACSBG1 | 1 |
| ACSL1 | 1 |
| ACSL6 | 1 |
| ACSM4 | 1 |
| ACTC1 | 1 |
| ACTG2 | 1 |
| ACTL6A | 1 |
| ACTL6B | 1 |
| ACTL8 | 1 |
| ACTR2 | 1 |
| ACTR3 | 1 |
| ACTR5 | 1 |
| ACVR1C | 1 |
| ACYP2 | 1 |
| ADAM11 | 1 |
| ADAM19 | 1 |
| ADAM20 | 1 |
| ADAM28 | 1 |
| ADAM29 | 1 |
| ADAM33 | 1 |
| ADAM3A | 1 |
| ADAM8 | 1 |
| ADAM9 | 1 |
| ADAMTS3 | 1 |
| ADAMTS5 | 1 |
| ADAMTS6 | 1 |
| ADAMTS8 | 1 |
| ADAP1 | 1 |
| ADAR | 1 |
| ADAT2 | 1 |
| ADCK2 | 1 |
| ADCK4 | 1 |
| ADCY3 | 1 |
| ADCY6 | 1 |
| ADH1A | 1 |
| ADIPOR1 | 1 |
| ADNP | 1 |
| ADNP2 | 1 |
| ADORA2A | 1 |
| ADORA3 | 1 |
| ADPGK | 1 |
| ADRA1A | 1 |
| ADRA2B | 1 |
| ADRM1 | 1 |
| ADSL | 1 |
| AFAP1L1 | 1 |
| AFM | 1 |
| AFP | 1 |
| AFTPH | 1 |
| AGAP3 | 1 |
| AGAP6 | 1 |
| AGBL2 | 1 |
| AGGF1 | 1 |
| AGK | 1 |
| AGMO | 1 |
| AGPAT3 | 1 |
| AGPAT4 | 1 |
| AGPS | 1 |
| AGRN | 1 |
| AGXT | 1 |
| AHCY | 1 |
| AHCYL1 | 1 |
| AHCYL2 | 1 |
| AHR | 1 |
| AHRR | 1 |
| AHSG | 1 |
| AIDA | 1 |
| AIFM3 | 1 |
| AIM1 | 1 |
| AIM1L | 1 |
| AIPL1 | 1 |
| AK5 | 1 |
| AKAP10 | 1 |
| AKAP4 | 1 |
| AKAP8 | 1 |
| AKAP8L | 1 |
| AKNAD1 | 1 |
| AKR1D1 | 1 |
| AKR7A2 | 1 |
| ALAS1 | 1 |
| ALDH18A1 | 1 |
| ALDH1A2 | 1 |
| ALDH1A3 | 1 |
| ALDH2 | 1 |
| ALDH3A2 | 1 |
| ALDH5A1 | 1 |
| ALDH8A1 | 1 |
| ALDOA | 1 |
| ALG10 | 1 |
| ALG13 | 1 |
| ALG1L | 1 |
| ALG3 | 1 |
| ALG8 | 1 |
| ALOXE3 | 1 |
| ALPK1 | 1 |
| ALPK2 | 1 |
| ALPP | 1 |
| ALPPL2 | 1 |
| ALS2 | 1 |
| ALS2CL | 1 |
| ALX1 | 1 |
| ALX4 | 1 |
| AMBP | 1 |
| AMD1 | 1 |
| AMDHD1 | 1 |
| AMPD1 | 1 |
| AMT | 1 |
| ANAPC2 | 1 |
| ANAPC7 | 1 |
| ANGEL1 | 1 |
| ANGEL2 | 1 |
| ANGPT1 | 1 |
| ANGPT2 | 1 |
| ANGPT4 | 1 |
| ANGPTL1 | 1 |
| ANGPTL4 | 1 |
| ANGPTL7 | 1 |
| ANK1 | 1 |
| ANKDD1A | 1 |
| ANKFY1 | 1 |
| ANKLE1 | 1 |
| ANKLE2 | 1 |
| ANKRD1 | 1 |
| ANKRD10 | 1 |
| ANKRD11 | 1 |
| ANKRD18A | 1 |
| ANKRD18B | 1 |
| ANKRD2 | 1 |
| ANKRD20A1 | 1 |
| ANKRD30BL | 1 |
| ANKRD32 | 1 |
| ANKRD33 | 1 |
| ANKRD34C | 1 |
| ANKRD36B | 1 |
| ANKRD36BP1 | 1 |
| ANKRD5 | 1 |
| ANKRD55 | 1 |
| ANKRD62 | 1 |
| ANKRD7 | 1 |
| ANKS6 | 1 |
| ANKUB1 | 1 |
| ANO2 | 1 |
| ANO5 | 1 |
| ANO8 | 1 |
| ANXA1 | 1 |
| ANXA13 | 1 |
| ANXA3 | 1 |
| ANXA4 | 1 |
| ANXA6 | 1 |
| ANXA7 | 1 |
| AOAH | 1 |
| AP1G1 | 1 |
| AP1G2 | 1 |
| AP2A1 | 1 |
| AP2B1 | 1 |
| AP3B1 | 1 |
| AP3B2 | 1 |
| AP3M2 | 1 |
| APBB1 | 1 |
| APBB1IP | 1 |
| APCDD1L | 1 |
| APH1A | 1 |
| API5 | 1 |
| APLP1 | 1 |
| APLP2 | 1 |
| APOA1 | 1 |
| APOA4 | 1 |
| APOBEC3D | 1 |
| APOBEC3G | 1 |
| APOBEC3H | 1 |
| APOC2 | 1 |
| APOE | 1 |
| APOL5 | 1 |
| APOL6 | 1 |
| APP | 1 |
| APPL1 | 1 |
| APRT | 1 |
| AQP10 | 1 |
| AQP12A | 1 |
| AQP6 | 1 |
| AR | 1 |
| ARAF | 1 |
| ARAP3 | 1 |
| ARF1 | 1 |
| ARF5 | 1 |
| ARFGAP2 | 1 |
| ARFGEF2 | 1 |
| ARFIP1 | 1 |
| ARHGAP22 | 1 |
| ARHGAP24 | 1 |
| ARHGAP25 | 1 |
| ARHGAP26 | 1 |
| ARHGAP29 | 1 |
| ARHGAP31 | 1 |
| ARHGAP42 | 1 |
| ARHGAP44 | 1 |
| ARHGAP8 | 1 |
| ARHGDIA | 1 |
| ARHGDIB | 1 |
| ARHGDIG | 1 |
| ARHGEF1 | 1 |
| ARHGEF10 | 1 |
| ARHGEF17 | 1 |
| ARHGEF35 | 1 |
| ARHGEF37 | 1 |
| ARHGEF40 | 1 |
| ARHGEF5 | 1 |
| ARID3C | 1 |
| ARID4B | 1 |
| ARL3 | 1 |
| ARL4A | 1 |
| ARL5A | 1 |
| ARL5B | 1 |
| ARL6IP6 | 1 |
| ARMC1 | 1 |
| ARMC10 | 1 |
| ARMC2 | 1 |
| ARMC3 | 1 |
| ARMC4 | 1 |
| ARMC5 | 1 |
| ARMC6 | 1 |
| ARMC8 | 1 |
| ARMC9 | 1 |
| ARMCX5-GPRASP2 | 1 |
| ARMCX6 | 1 |
| ARNTL | 1 |
| ARPC1A | 1 |
| ARPC1B | 1 |
| ARPP21 | 1 |
| ARRB2 | 1 |
| ARRDC1 | 1 |
| ARSD | 1 |
| ARSF | 1 |
| ARSH | 1 |
| ARSK | 1 |
| ART1 | 1 |
| ART3 | 1 |
| ART4 | 1 |
| ARVCF | 1 |
| ASAP1 | 1 |
| ASB14 | 1 |
| ASB16 | 1 |
| ASCC1 | 1 |
| ASPN | 1 |
| ASS1P10 | 1 |
| ASTE1 | 1 |
| ASTN1 | 1 |
| ASTN2 | 1 |
| ASXL1 | 1 |
| ATAD2 | 1 |
| ATAD3B | 1 |
| ATAD3C | 1 |
| ATCAY | 1 |
| ATF2 | 1 |
| ATF4 | 1 |
| ATF5 | 1 |
| ATF6B | 1 |
| ATF7IP | 1 |
| ATG2A | 1 |
| ATG4B | 1 |
| ATG7 | 1 |
| ATG9A | 1 |
| ATHL1 | 1 |
| ATL3 | 1 |
| ATP11C | 1 |
| ATP12A | 1 |
| ATP13A3 | 1 |
| ATP1A1 | 1 |
| ATP1A3 | 1 |
| ATP1A4 | 1 |
| ATP1B4 | 1 |
| ATP2B1 | 1 |
| ATP2B2 | 1 |
| ATP2B3 | 1 |
| ATP2B4 | 1 |
| ATP2C2 | 1 |
| ATP4B | 1 |
| ATP5G1 | 1 |
| ATP5G2 | 1 |
| ATP5O | 1 |
| ATP5S | 1 |
| ATP6V0A4 | 1 |
| ATP6V1B1 | 1 |
| ATP6V1F | 1 |
| ATP8A2 | 1 |
| ATXN10 | 1 |
| ATXN3 | 1 |
| ATXN3L | 1 |
| ATXN7 | 1 |
| AVIL | 1 |
| AWAT1 | 1 |
| AZI2 | 1 |
| B3GALT2 | 1 |
| B3GALT5 | 1 |
| B3GALTL | 1 |
| BABAM1 | 1 |
| BACH2 | 1 |
| BAG3 | 1 |
| BAG4 | 1 |
| BAI2 | 1 |
| BAIAP2 | 1 |
| BAIAP3 | 1 |
| BAMBI | 1 |
| BANP | 1 |
| BARD1 | 1 |
| BARX2 | 1 |
| BASP1 | 1 |
| BATF2 | 1 |
| BAZ1B | 1 |
| BAZ2A | 1 |
| BBS1 | 1 |
| BBS12 | 1 |
| BCAN | 1 |
| BCAP29 | 1 |
| BCAR1 | 1 |
| BCAS1 | 1 |
| BCAS2 | 1 |
| BCAS3 | 1 |
| BCAS4 | 1 |
| BCAT1 | 1 |
| BCHE | 1 |
| BCKDHA | 1 |
| BCL2A1 | 1 |
| BCL2L13 | 1 |
| BCL2L15 | 1 |
| BCL3 | 1 |
| BCL6 | 1 |
| BCLAF1 | 1 |
| BCMO1 | 1 |
| BFSP1 | 1 |
| BHLHB9 | 1 |
| BHMT2 | 1 |
| BICC1 | 1 |
| BIN2 | 1 |
| BIRC2 | 1 |
| BLCAP | 1 |
| BLK | 1 |
| BLNK | 1 |
| BLVRA | 1 |
| BMI1 | 1 |
| BMP1 | 1 |
| BMP2K | 1 |
| BMP6 | 1 |
| BMP8B | 1 |
| BNC1 | 1 |
| BNC2 | 1 |
| BNIP2 | 1 |
| BOP1 | 1 |
| BPI | 1 |
| BPIFB4 | 1 |
| BPIFB6 | 1 |
| BRAF | 1 |
| BRAP | 1 |
| BRAT1 | 1 |
| BRD9 | 1 |
| BRI3 | 1 |
| BRIX1 | 1 |
| BRMS1 | 1 |
| BRMS1L | 1 |
| BROX | 1 |
| BRPF1 | 1 |
| BRSK2 | 1 |
| BSDC1 | 1 |
| BSPRY | 1 |
| BTAF1 | 1 |
| BTBD18 | 1 |
| BTBD3 | 1 |
| BTBD6 | 1 |
| BTBD7 | 1 |
| BTLA | 1 |
| BTN2A1 | 1 |
| BTN2A2 | 1 |
| BTN3A1 | 1 |
| BTN3A3 | 1 |
| BTRC | 1 |
| BUB1 | 1 |
| C10orf114 | 1 |
| C10orf126 | 1 |
| C10orf128 | 1 |
| C10orf2 | 1 |
| C10orf35 | 1 |
| C10orf67 | 1 |
| C10orf71 | 1 |
| C10orf76 | 1 |
| C10orf95 | 1 |
| C11orf31 | 1 |
| C11orf35 | 1 |
| C11orf44 | 1 |
| C11orf57 | 1 |
| C11orf58 | 1 |
| C11orf9 | 1 |
| C11orf95 | 1 |
| C12orf43 | 1 |
| C12orf5 | 1 |
| C12orf68 | 1 |
| C12orf69 | 1 |
| C13orf35 | 1 |
| C14orf101 | 1 |
| C14orf102 | 1 |
| C14orf159 | 1 |
| C14orf166 | 1 |
| C14orf28 | 1 |
| C14orf37 | 1 |
| C14orf38 | 1 |
| C14orf39 | 1 |
| C15orf23 | 1 |
| C15orf29 | 1 |
| C15orf32 | 1 |
| C15orf39 | 1 |
| C15orf59 | 1 |
| C16orf11 | 1 |
| C16orf52 | 1 |
| C16orf58 | 1 |
| C16orf71 | 1 |
| C16orf78 | 1 |
| C16orf92 | 1 |
| C17orf28 | 1 |
| C17orf51 | 1 |
| C17orf53 | 1 |
| C17orf80 | 1 |
| C18orf1 | 1 |
| C18orf54 | 1 |
| C19orf18 | 1 |
| C19orf21 | 1 |
| C19orf26 | 1 |
| C19orf43 | 1 |
| C19orf66 | 1 |
| C1QA | 1 |
| C1QTNF1 | 1 |
| C1QTNF2 | 1 |
| C1QTNF9B | 1 |
| C1R | 1 |
| C1orf112 | 1 |
| C1orf114 | 1 |
| C1orf127 | 1 |
| C1orf168 | 1 |
| C1orf194 | 1 |
| C1orf43 | 1 |
| C1orf65 | 1 |
| C1orf88 | 1 |
| C2 | 1 |
| C20orf112 | 1 |
| C20orf194 | 1 |
| C20orf197 | 1 |
| C20orf27 | 1 |
| C20orf78 | 1 |
| C21orf49 | 1 |
| C21orf59 | 1 |
| C21orf62 | 1 |
| C22orf25 | 1 |
| C22orf34 | 1 |
| C2orf16 | 1 |
| C2orf18 | 1 |
| C2orf29 | 1 |
| C2orf40 | 1 |
| C2orf42 | 1 |
| C2orf43 | 1 |
| C2orf65 | 1 |
| C2orf73 | 1 |
| C2orf88 | 1 |
| C3orf15 | 1 |
| C3orf20 | 1 |
| C3orf24 | 1 |
| C3orf27 | 1 |
| C3orf33 | 1 |
| C3orf35 | 1 |
| C3orf49 | 1 |
| C3orf52 | 1 |
| C3orf67 | 1 |
| C4orf17 | 1 |
| C4orf21 | 1 |
| C4orf27 | 1 |
| C4orf29 | 1 |
| C4orf3 | 1 |
| C4orf50 | 1 |
| C5orf17 | 1 |
| C5orf4 | 1 |
| C5orf54 | 1 |
| C5orf58 | 1 |
| C6 | 1 |
| C6orf123 | 1 |
| C6orf162 | 1 |
| C6orf163 | 1 |
| C6orf195 | 1 |
| C6orf222 | 1 |
| C6orf25 | 1 |
| C6orf70 | 1 |
| C6orf89 | 1 |
| C7 | 1 |
| C7orf10 | 1 |
| C7orf34 | 1 |
| C7orf45 | 1 |
| C7orf49 | 1 |
| C7orf50 | 1 |
| C7orf65 | 1 |
| C8B | 1 |
| C8orf34 | 1 |
| C8orf48 | 1 |
| C9orf129 | 1 |
| C9orf43 | 1 |
| C9orf47 | 1 |
| C9orf72 | 1 |
| C9orf89 | 1 |
| CA10 | 1 |
| CA11 | 1 |
| CA12 | 1 |
| CA14 | 1 |
| CA3 | 1 |
| CAB39 | 1 |
| CABIN1 | 1 |
| CABYR | 1 |
| CACNA1A | 1 |
| CACNA1B | 1 |
| CACNA1G | 1 |
| CACNA2D1 | 1 |
| CACNB1 | 1 |
| CACNB2 | 1 |
| CACNB4 | 1 |
| CACNG7 | 1 |
| CADM1 | 1 |
| CADM3 | 1 |
| CADPS | 1 |
| CALHM1 | 1 |
| CALML3 | 1 |
| CALU | 1 |
| CAMKV | 1 |
| CAMP | 1 |
| CAMSAP1 | 1 |
| CAMSAP2 | 1 |
| CAMTA1 | 1 |
| CAND1 | 1 |
| CAND2 | 1 |
| CANX | 1 |
| CAP1 | 1 |
| CAPG | 1 |
| CAPN12 | 1 |
| CAPN13 | 1 |
| CAPN8 | 1 |
| CAPNS2 | 1 |
| CAPRIN1 | 1 |
| CAPS2 | 1 |
| CAPSL | 1 |
| CAPZA2 | 1 |
| CAPZA3 | 1 |
| CARD14 | 1 |
| CARD8 | 1 |
| CARD9 | 1 |
| CARKD | 1 |
| CARM1 | 1 |
| CARTPT | 1 |
| CASKIN1 | 1 |
| CASP14 | 1 |
| CASP4 | 1 |
| CASQ2 | 1 |
| CASR | 1 |
| CATSPER3 | 1 |
| CATSPERB | 1 |
| CBFA2T2 | 1 |
| CBLL1 | 1 |
| CBLN4 | 1 |
| CBS | 1 |
| CBX6 | 1 |
| CCDC104 | 1 |
| CCDC107 | 1 |
| CCDC109B | 1 |
| CCDC112 | 1 |
| CCDC115 | 1 |
| CCDC12 | 1 |
| CCDC13 | 1 |
| CCDC138 | 1 |
| CCDC141 | 1 |
| CCDC142 | 1 |
| CCDC148 | 1 |
| CCDC15 | 1 |
| CCDC151 | 1 |
| CCDC154 | 1 |
| CCDC155 | 1 |
| CCDC158 | 1 |
| CCDC169 | 1 |
| CCDC169-SOHLH2 | 1 |
| CCDC17 | 1 |
| CCDC27 | 1 |
| CCDC33 | 1 |
| CCDC36 | 1 |
| CCDC37 | 1 |
| CCDC38 | 1 |
| CCDC40 | 1 |
| CCDC47 | 1 |
| CCDC50 | 1 |
| CCDC51 | 1 |
| CCDC54 | 1 |
| CCDC64 | 1 |
| CCDC66 | 1 |
| CCDC67 | 1 |
| CCDC7 | 1 |
| CCDC73 | 1 |
| CCDC77 | 1 |
| CCDC78 | 1 |
| CCDC8 | 1 |
| CCDC83 | 1 |
| CCDC84 | 1 |
| CCDC86 | 1 |
| CCDC87 | 1 |
| CCDC88A | 1 |
| CCDC88C | 1 |
| CCDC9 | 1 |
| CCDC90A | 1 |
| CCDC90B | 1 |
| CCIN | 1 |
| CCL11 | 1 |
| CCL23 | 1 |
| CCL5 | 1 |
| CCNB1 | 1 |
| CCNB1IP1 | 1 |
| CCNC | 1 |
| CCNG2 | 1 |
| CCNH | 1 |
| CCNJL | 1 |
| CCNT1 | 1 |
| CCNT2 | 1 |
| CCNY | 1 |
| CCNYL2 | 1 |
| CCR1 | 1 |
| CCR7 | 1 |
| CCR9 | 1 |
| CCRN4L | 1 |
| CCT3 | 1 |
| CCT8 | 1 |
| CD101 | 1 |
| CD160 | 1 |
| CD1A | 1 |
| CD1E | 1 |
| CD2 | 1 |
| CD200 | 1 |
| CD200R1 | 1 |
| CD248 | 1 |
| CD274 | 1 |
| CD2BP2 | 1 |
| CD300A | 1 |
| CD300LB | 1 |
| CD33 | 1 |
| CD40 | 1 |
| CD46 | 1 |
| CD5 | 1 |
| CD6 | 1 |
| CD72 | 1 |
| CD79A | 1 |
| CD79B | 1 |
| CD82 | 1 |
| CD84 | 1 |
| CD93 | 1 |
| CD96 | 1 |
| CDADC1 | 1 |
| CDC16 | 1 |
| CDC25C | 1 |
| CDC27 | 1 |
| CDC37 | 1 |
| CDC40 | 1 |
| CDC42BPA | 1 |
| CDC42BPB | 1 |
| CDC42BPG | 1 |
| CDC6 | 1 |
| CDCP1 | 1 |
| CDH17 | 1 |
| CDH2 | 1 |
| CDH20 | 1 |
| CDH22 | 1 |
| CDH23 | 1 |
| CDH24 | 1 |
| CDH3 | 1 |
| CDH4 | 1 |
| CDH5 | 1 |
| CDHR1 | 1 |
| CDHR2 | 1 |
| CDHR3 | 1 |
| CDIPT | 1 |
| CDK12 | 1 |
| CDK14 | 1 |
| CDK15 | 1 |
| CDK18 | 1 |
| CDK19 | 1 |
| CDK2AP1 | 1 |
| CDK2AP2 | 1 |
| CDK5R1 | 1 |
| CDK5RAP2 | 1 |
| CDK5RAP3 | 1 |
| CDKL3 | 1 |
| CDKL4 | 1 |
| CDKN2AIP | 1 |
| CDKN2C | 1 |
| CDO1 | 1 |
| CDR2L | 1 |
| CDS1 | 1 |
| CDYL2 | 1 |
| CEACAM18 | 1 |
| CEACAM19 | 1 |
| CEACAM8 | 1 |
| CEBPG | 1 |
| CECR1 | 1 |
| CECR5 | 1 |
| CELF2 | 1 |
| CENPF | 1 |
| CENPI | 1 |
| CENPT | 1 |
| CEP170 | 1 |
| CEP350 | 1 |
| CEP44 | 1 |
| CEP57L1 | 1 |
| CEP63 | 1 |
| CEP72 | 1 |
| CEP89 | 1 |
| CER1 | 1 |
| CERCAM | 1 |
| CERS6 | 1 |
| CES1 | 1 |
| CES2 | 1 |
| CES4A | 1 |
| CES5A | 1 |
| CFHR4 | 1 |
| CFHR5 | 1 |
| CGB1 | 1 |
| CHAC2 | 1 |
| CHAD | 1 |
| CHAF1B | 1 |
| CHCHD3 | 1 |
| CHD3 | 1 |
| CHD4 | 1 |
| CHEK1 | 1 |
| CHI3L1 | 1 |
| CHI3L2 | 1 |
| CHIA | 1 |
| CHID1 | 1 |
| CHM | 1 |
| CHMP1A | 1 |
| CHODL | 1 |
| CHORDC1 | 1 |
| CHPF | 1 |
| CHPF2 | 1 |
| CHRD | 1 |
| CHRDL2 | 1 |
| CHRM2 | 1 |
| CHRM3 | 1 |
| CHRNA1 | 1 |
| CHRNA4 | 1 |
| CHRNA6 | 1 |
| CHRND | 1 |
| CHST10 | 1 |
| CHST11 | 1 |
| CHST12 | 1 |
| CHST15 | 1 |
| CHST8 | 1 |
| CHTF18 | 1 |
| CHTF8 | 1 |
| CHUK | 1 |
| CIAPIN1 | 1 |
| CIC | 1 |
| CILP | 1 |
| CIR1 | 1 |
| CISD1 | 1 |
| CKAP5 | 1 |
| CKMT1B | 1 |
| CLASP1 | 1 |
| CLASRP | 1 |
| CLCC1 | 1 |
| CLCN2 | 1 |
| CLCN3 | 1 |
| CLCNKA | 1 |
| CLDN11 | 1 |
| CLDN14 | 1 |
| CLDN18 | 1 |
| CLDN20 | 1 |
| CLDN22 | 1 |
| CLDN5 | 1 |
| CLEC11A | 1 |
| CLEC14A | 1 |
| CLEC16A | 1 |
| CLEC4D | 1 |
| CLIC2 | 1 |
| CLINT1 | 1 |
| CLIP2 | 1 |
| CLK2 | 1 |
| CLK3 | 1 |
| CLK4 | 1 |
| CLMN | 1 |
| CLMP | 1 |
| CLN5 | 1 |
| CLNK | 1 |
| CLNS1A | 1 |
| CLP1 | 1 |
| CLPP | 1 |
| CLPX | 1 |
| CLSTN1 | 1 |
| CLTC | 1 |
| CLYBL | 1 |
| CMAHP | 1 |
| CMKLR1 | 1 |
| CMTM4 | 1 |
| CMTM7 | 1 |
| CMYA5 | 1 |
| CNGA2 | 1 |
| CNGB1 | 1 |
| CNGB3 | 1 |
| CNIH3 | 1 |
| CNOT10 | 1 |
| CNOT4 | 1 |
| CNR1 | 1 |
| CNR2 | 1 |
| CNST | 1 |
| CNTD1 | 1 |
| CNTFR | 1 |
| CNTN2 | 1 |
| CNTN3 | 1 |
| CNTNAP3 | 1 |
| COG1 | 1 |
| COG5 | 1 |
| COG6 | 1 |
| COIL | 1 |
| COL11A2 | 1 |
| COL13A1 | 1 |
| COL18A1 | 1 |
| COL20A1 | 1 |
| COL28A1 | 1 |
| COL3A1 | 1 |
| COL4A2 | 1 |
| COL4A2-AS2 | 1 |
| COL4A3 | 1 |
| COL4A6 | 1 |
| COL5A2 | 1 |
| COL6A1 | 1 |
| COL6A2 | 1 |
| COL7A1 | 1 |
| COL8A1 | 1 |
| COL9A2 | 1 |
| COMMD1 | 1 |
| COMMD3 | 1 |
| COPS6 | 1 |
| COPS7A | 1 |
| COPS7B | 1 |
| COPZ1 | 1 |
| COQ10A | 1 |
| COQ2 | 1 |
| CORO1C | 1 |
| CORO2A | 1 |
| CORO2B | 1 |
| COX11 | 1 |
| COX15 | 1 |
| COX19 | 1 |
| COX4I2 | 1 |
| COX6C | 1 |
| COX7B2 | 1 |
| CPA2 | 1 |
| CPA3 | 1 |
| CPA5 | 1 |
| CPB2 | 1 |
| CPD | 1 |
| CPEB1 | 1 |
| CPEB4 | 1 |
| CPM | 1 |
| CPNE1 | 1 |
| CPNE6 | 1 |
| CPO | 1 |
| CPSF3L | 1 |
| CPT1A | 1 |
| CPT2 | 1 |
| CPVL | 1 |
| CPXCR1 | 1 |
| CPXM2 | 1 |
| CPZ | 1 |
| CRAT | 1 |
| CRB1 | 1 |
| CRB2 | 1 |
| CREB5 | 1 |
| CREBZF | 1 |
| CREM | 1 |
| CRHR2 | 1 |
| CRIM1 | 1 |
| CRIP3 | 1 |
| CRNKL1 | 1 |
| CROT | 1 |
| CRTAM | 1 |
| CRY2 | 1 |
| CRYBA4 | 1 |
| CRYGN | 1 |
| CRYGS | 1 |
| CSE1L | 1 |
| CSF1 | 1 |
| CSF3R | 1 |
| CSGALNACT1 | 1 |
| CSN1S1 | 1 |
| CSNK1E | 1 |
| CSNK1G1 | 1 |
| CSNK2B | 1 |
| CSRNP3 | 1 |
| CSRP2 | 1 |
| CSRP3 | 1 |
| CST2 | 1 |
| CST5 | 1 |
| CST6 | 1 |
| CSTB | 1 |
| CSTF2 | 1 |
| CSTF3 | 1 |
| CTAGE5 | 1 |
| CTC1 | 1 |
| CTCF | 1 |
| CTDP1 | 1 |
| CTDSPL2 | 1 |
| CTIF | 1 |
| CTNNAL1 | 1 |
| CTPS2 | 1 |
| CTR9 | 1 |
| CTRL | 1 |
| CTSC | 1 |
| CTSF | 1 |
| CTSL2 | 1 |
| CTSZ | 1 |
| CTTN | 1 |
| CUL3 | 1 |
| CUL7 | 1 |
| CUX1 | 1 |
| CUZD1 | 1 |
| CWC22 | 1 |
| CWF19L1 | 1 |
| CWF19L2 | 1 |
| CWH43 | 1 |
| CXCL2 | 1 |
| CXCL9 | 1 |
| CXXC4 | 1 |
| CXorf22 | 1 |
| CXorf28 | 1 |
| CXorf59 | 1 |
| CYB5A | 1 |
| CYB5D2 | 1 |
| CYB5R4 | 1 |
| CYBA | 1 |
| CYBRD1 | 1 |
| CYFIP2 | 1 |
| CYHR1 | 1 |
| CYLC2 | 1 |
| CYP2B6 | 1 |
| CYP2C18 | 1 |
| CYP2C19 | 1 |
| CYP2C8 | 1 |
| CYP2C9 | 1 |
| CYP2F1 | 1 |
| CYP2R1 | 1 |
| CYP2W1 | 1 |
| CYP4F11 | 1 |
| CYP4F2 | 1 |
| CYP4F3 | 1 |
| CYP4F8 | 1 |
| CYP4V2 | 1 |
| CYP8B1 | 1 |
| CYSLTR1 | 1 |
| CYSLTR2 | 1 |
| CYTH3 | 1 |
| DAAM1 | 1 |
| DAAM2 | 1 |
| DAB2 | 1 |
| DAG1 | 1 |
| DAGLB | 1 |
| DAK | 1 |
| DAO | 1 |
| DAP3 | 1 |
| DAPK3 | 1 |
| DARC | 1 |
| DAZL | 1 |
| DBF4B | 1 |
| DBH | 1 |
| DBX2 | 1 |
| DCAF11 | 1 |
| DCAF15 | 1 |
| DCAF16 | 1 |
| DCAF4L1 | 1 |
| DCDC1 | 1 |
| DCDC2 | 1 |
| DCLK1 | 1 |
| DCLK3 | 1 |
| DCLRE1B | 1 |
| DCT | 1 |
| DCTN1 | 1 |
| DDAH1 | 1 |
| DDC | 1 |
| DDR1 | 1 |
| DDRGK1 | 1 |
| DDX11 | 1 |
| DDX24 | 1 |
| DDX26B | 1 |
| DDX27 | 1 |
| DDX28 | 1 |
| DDX3X | 1 |
| DDX3Y | 1 |
| DDX42 | 1 |
| DDX46 | 1 |
| DDX47 | 1 |
| DDX50 | 1 |
| DDX55 | 1 |
| DDX58 | 1 |
| DDX6 | 1 |
| DEAF1 | 1 |
| DEFB104A | 1 |
| DEFB126 | 1 |
| DEFB128 | 1 |
| DEFB129 | 1 |
| DEGS2 | 1 |
| DEK | 1 |
| DENND4A | 1 |
| DENND4C | 1 |
| DEPDC1 | 1 |
| DEPDC5 | 1 |
| DEPDC7 | 1 |
| DEPTOR | 1 |
| DERL3 | 1 |
| DFNB31 | 1 |
| DFNB59 | 1 |
| DGAT2 | 1 |
| DGKA | 1 |
| DGKD | 1 |
| DGKG | 1 |
| DGKH | 1 |
| DGKI | 1 |
| DGUOK | 1 |
| DHCR24 | 1 |
| DHPS | 1 |
| DHX29 | 1 |
| DHX32 | 1 |
| DHX34 | 1 |
| DHX35 | 1 |
| DHX36 | 1 |
| DHX58 | 1 |
| DIAPH2 | 1 |
| DIAPH3 | 1 |
| DIEXF | 1 |
| DIP2A | 1 |
| DIP2C | 1 |
| DIS3L | 1 |
| DISP1 | 1 |
| DIXDC1 | 1 |
| DKK1 | 1 |
| DKK3 | 1 |
| DKK4 | 1 |
| DKKL1 | 1 |
| DLC1 | 1 |
| DLEC1 | 1 |
| DLG4 | 1 |
| DLGAP1 | 1 |
| DLGAP2 | 1 |
| DLGAP3 | 1 |
| DLGAP5 | 1 |
| DLL3 | 1 |
| DLX5 | 1 |
| DLX6 | 1 |
| DMRT3 | 1 |
| DMRTB1 | 1 |
| DMXL2 | 1 |
| DNAJA1 | 1 |
| DNAJA3 | 1 |
| DNAJB4 | 1 |
| DNAJC10 | 1 |
| DNAJC17 | 1 |
| DNAJC25 | 1 |
| DNAJC30 | 1 |
| DNAJC5B | 1 |
| DNHD1 | 1 |
| DNM1 | 1 |
| DNMBP | 1 |
| DNMT1 | 1 |
| DOCK5 | 1 |
| DOK6 | 1 |
| DPCR1 | 1 |
| DPEP2 | 1 |
| DPF3 | 1 |
| DPH5 | 1 |
| DPM1 | 1 |
| DPP6 | 1 |
| DPT | 1 |
| DPY19L2 | 1 |
| DPY19L3 | 1 |
| DPY19L4 | 1 |
| DPYSL2 | 1 |
| DRAP1 | 1 |
| DRD3 | 1 |
| DROSHA | 1 |
| DRP2 | 1 |
| DSC2 | 1 |
| DSCR3 | 1 |
| DSG3 | 1 |
| DTHD1 | 1 |
| DTNBP1 | 1 |
| DTX1 | 1 |
| DTX3L | 1 |
| DUOX1 | 1 |
| DUSP1 | 1 |
| DUSP13 | 1 |
| DUSP16 | 1 |
| DUSP19 | 1 |
| DUSP22 | 1 |
| DYNC1I2 | 1 |
| DYNC1LI2 | 1 |
| DYNLRB2 | 1 |
| DYRK2 | 1 |
| DYTN | 1 |
| DYX1C1 | 1 |
| DZIP1 | 1 |
| E2F8 | 1 |
| EAF1 | 1 |
| EBF1 | 1 |
| ECD | 1 |
| ECHDC2 | 1 |
| ECSCR | 1 |
| ECT2 | 1 |
| EDARADD | 1 |
| EDC3 | 1 |
| EDEM1 | 1 |
| EDN3 | 1 |
| EDNRA | 1 |
| EED | 1 |
| EEF1E1 | 1 |
| EEFSEC | 1 |
| EFCAB3 | 1 |
| EFCAB6 | 1 |
| EFCAB9 | 1 |
| EFHC1 | 1 |
| EFHC2 | 1 |
| EFNB2 | 1 |
| EFS | 1 |
| EFTUD2 | 1 |
| EGFL6 | 1 |
| EGFLAM | 1 |
| EGFR | 1 |
| EGR1 | 1 |
| EHD3 | 1 |
| EHHADH | 1 |
| EID2B | 1 |
| EIF2AK1 | 1 |
| EIF2AK4 | 1 |
| EIF2B3 | 1 |
| EIF2C2 | 1 |
| EIF2C4 | 1 |
| EIF3D | 1 |
| EIF3E | 1 |
| EIF3H | 1 |
| EIF3J | 1 |
| EIF3M | 1 |
| EIF4A1 | 1 |
| EIF4E2 | 1 |
| EIF4G3 | 1 |
| EIF5B | 1 |
| ELAC2 | 1 |
| ELAVL2 | 1 |
| ELAVL4 | 1 |
| ELF3 | 1 |
| ELK1 | 1 |
| ELK3 | 1 |
| ELL | 1 |
| ELL2 | 1 |
| ELMOD1 | 1 |
| ELN | 1 |
| ELOVL4 | 1 |
| ELOVL7 | 1 |
| ELP4 | 1 |
| ELTD1 | 1 |
| EMCN | 1 |
| EMD | 1 |
| EMILIN1 | 1 |
| EMILIN2 | 1 |
| EML1 | 1 |
| EML4 | 1 |
| EMP1 | 1 |
| ENAM | 1 |
| ENG | 1 |
| ENKUR | 1 |
| ENOSF1 | 1 |
| ENPP5 | 1 |
| ENPP6 | 1 |
| ENPP7 | 1 |
| ENTHD1 | 1 |
| ENTPD3 | 1 |
| EPAS1 | 1 |
| EPB41 | 1 |
| EPB41L2 | 1 |
| EPB41L4A | 1 |
| EPB49 | 1 |
| EPC2 | 1 |
| EPCAM | 1 |
| EPGN | 1 |
| EPHA2 | 1 |
| EPHB3 | 1 |
| EPHB4 | 1 |
| EPHB6 | 1 |
| EPHX2 | 1 |
| EPM2AIP1 | 1 |
| EPN1 | 1 |
| EPS8 | 1 |
| EPS8L3 | 1 |
| EPX | 1 |
| ERBB2 | 1 |
| ERBB3 | 1 |
| ERCC2 | 1 |
| ERGIC3 | 1 |
| ERICH1 | 1 |
| ERMP1 | 1 |
| ERN1 | 1 |
| ERO1L | 1 |
| ERO1LB | 1 |
| ERP44 | 1 |
| ESAM | 1 |
| ESF1 | 1 |
| ESPN | 1 |
| ESRRB | 1 |
| ESYT3 | 1 |
| ETHE1 | 1 |
| ETNK1 | 1 |
| ETS1 | 1 |
| ETS2 | 1 |
| ETV1 | 1 |
| ETV5 | 1 |
| EVI2A | 1 |
| EVI5 | 1 |
| EVX1 | 1 |
| EWSR1 | 1 |
| EXD1 | 1 |
| EXOC8 | 1 |
| EXPH5 | 1 |
| EXT1 | 1 |
| EXTL1 | 1 |
| EYA3 | 1 |
| EZH2 | 1 |
| EZR | 1 |
| F12 | 1 |
| F2 | 1 |
| FAAH2 | 1 |
| FADS1 | 1 |
| FADS2 | 1 |
| FADS3 | 1 |
| FADS6 | 1 |
| FAHD1 | 1 |
| FAHD2B | 1 |
| FAIM2 | 1 |
| FAM102B | 1 |
| FAM105A | 1 |
| FAM108B1 | 1 |
| FAM109A | 1 |
| FAM109B | 1 |
| FAM110B | 1 |
| FAM116B | 1 |
| FAM118A | 1 |
| FAM120C | 1 |
| FAM123A | 1 |
| FAM124A | 1 |
| FAM124B | 1 |
| FAM129A | 1 |
| FAM129B | 1 |
| FAM134A | 1 |
| FAM134C | 1 |
| FAM135A | 1 |
| FAM149A | 1 |
| FAM149B1 | 1 |
| FAM151A | 1 |
| FAM155A | 1 |
| FAM161A | 1 |
| FAM161B | 1 |
| FAM169A | 1 |
| FAM169B | 1 |
| FAM171B | 1 |
| FAM178A | 1 |
| FAM179A | 1 |
| FAM184B | 1 |
| FAM188A | 1 |
| FAM189B | 1 |
| FAM18A | 1 |
| FAM194B | 1 |
| FAM199X | 1 |
| FAM19A2 | 1 |
| FAM200B | 1 |
| FAM214A | 1 |
| FAM26D | 1 |
| FAM3A | 1 |
| FAM40A | 1 |
| FAM46A | 1 |
| FAM46B | 1 |
| FAM47A | 1 |
| FAM48A | 1 |
| FAM49A | 1 |
| FAM50B | 1 |
| FAM54A | 1 |
| FAM58A | 1 |
| FAM63B | 1 |
| FAM70B | 1 |
| FAM71A | 1 |
| FAM72A | 1 |
| FAM75C1 | 1 |
| FAM78A | 1 |
| FAM78B | 1 |
| FAM81A | 1 |
| FAM83A | 1 |
| FAM84A | 1 |
| FAM84B | 1 |
| FAM86C1 | 1 |
| FAM98C | 1 |
| FAM9B | 1 |
| FANCB | 1 |
| FANCC | 1 |
| FANCD2 | 1 |
| FANCE | 1 |
| FANCG | 1 |
| FAR1 | 1 |
| FAR2 | 1 |
| FARP1 | 1 |
| FARSB | 1 |
| FASN | 1 |
| FASTKD1 | 1 |
| FASTKD2 | 1 |
| FAU | 1 |
| FBF1 | 1 |
| FBLN1 | 1 |
| FBLN7 | 1 |
| FBRS | 1 |
| FBXL12 | 1 |
| FBXO17 | 1 |
| FBXO4 | 1 |
| FBXO45 | 1 |
| FBXO47 | 1 |
| FBXO48 | 1 |
| FBXO8 | 1 |
| FBXW12 | 1 |
| FBXW4 | 1 |
| FBXW8 | 1 |
| FCAR | 1 |
| FCER1G | 1 |
| FCGR2A | 1 |
| FCHO1 | 1 |
| FCHO2 | 1 |
| FCRLA | 1 |
| FEM1B | 1 |
| FEM1C | 1 |
| FEN1 | 1 |
| FETUB | 1 |
| FEZ1 | 1 |
| FEZF1 | 1 |
| FGB | 1 |
| FGD4 | 1 |
| FGD5 | 1 |
| FGD6 | 1 |
| FGF12 | 1 |
| FGF17 | 1 |
| FGF5 | 1 |
| FGG | 1 |
| FH | 1 |
| FHDC1 | 1 |
| FIBCD1 | 1 |
| FIBP | 1 |
| FIG4 | 1 |
| FIGN | 1 |
| FILIP1L | 1 |
| FIP1L1 | 1 |
| FKBP2 | 1 |
| FKBP9 | 1 |
| FKBPL | 1 |
| FLNA | 1 |
| FLOT2 | 1 |
| FLT4 | 1 |
| FMN2 | 1 |
| FMO4 | 1 |
| FMOD | 1 |
| FMR1 | 1 |
| FMR1NB | 1 |
| FN1 | 1 |
| FNDC3A | 1 |
| FNDC4 | 1 |
| FNIP1 | 1 |
| FNTA | 1 |
| FOLR4 | 1 |
| FOXA3 | 1 |
| FOXE1 | 1 |
| FOXG1 | 1 |
| FOXI2 | 1 |
| FOXJ1 | 1 |
| FOXK1 | 1 |
| FOXM1 | 1 |
| FOXN2 | 1 |
| FOXO4 | 1 |
| FOXP1 | 1 |
| FOXR2 | 1 |
| FPGT | 1 |
| FPGT-TNNI3K | 1 |
| FPR2 | 1 |
| FPR3 | 1 |
| FRG1 | 1 |
| FRMD1 | 1 |
| FRMD3 | 1 |
| FRMD4A | 1 |
| FRMD5 | 1 |
| FRMD7 | 1 |
| FRMPD1 | 1 |
| FRMPD2 | 1 |
| FSCB | 1 |
| FSCN1 | 1 |
| FSCN2 | 1 |
| FSCN3 | 1 |
| FSD1 | 1 |
| FSTL1 | 1 |
| FSTL5 | 1 |
| FTH1 | 1 |
| FTHL17 | 1 |
| FTL | 1 |
| FTMT | 1 |
| FTSJ2 | 1 |
| FTSJD2 | 1 |
| FUT10 | 1 |
| FYB | 1 |
| FYN | 1 |
| FZD1 | 1 |
| FZD2 | 1 |
| FZD3 | 1 |
| G2E3 | 1 |
| G6PC3 | 1 |
| GAB1 | 1 |
| GAB4 | 1 |
| GABBR1 | 1 |
| GABBR2 | 1 |
| GABPA | 1 |
| GABPB2 | 1 |
| GABRA5 | 1 |
| GABRB3 | 1 |
| GABRG2 | 1 |
| GAK | 1 |
| GAL | 1 |
| GAL3ST2 | 1 |
| GALK2 | 1 |
| GALNT1 | 1 |
| GALNT5 | 1 |
| GALNT6 | 1 |
| GALNT7 | 1 |
| GALNT8 | 1 |
| GALNTL1 | 1 |
| GALNTL2 | 1 |
| GALR1 | 1 |
| GALR3 | 1 |
| GALT | 1 |
| GAMT | 1 |
| GAP43 | 1 |
| GARNL3 | 1 |
| GAS2L2 | 1 |
| GAS2L3 | 1 |
| GAS6 | 1 |
| GAST | 1 |
| GATA3 | 1 |
| GATAD1 | 1 |
| GATAD2A | 1 |
| GATAD2B | 1 |
| GBE1 | 1 |
| GBF1 | 1 |
| GBGT1 | 1 |
| GBP2 | 1 |
| GBP4 | 1 |
| GBP5 | 1 |
| GBP6 | 1 |
| GC | 1 |
| GCG | 1 |
| GCK | 1 |
| GCKR | 1 |
| GCLM | 1 |
| GCM1 | 1 |
| GCN1L1 | 1 |
| GCNT2 | 1 |
| GCOM1 | 1 |
| GDAP2 | 1 |
| GDF15 | 1 |
| GDI2 | 1 |
| GDNF | 1 |
| GDPD1 | 1 |
| GDPD5 | 1 |
| GEMIN5 | 1 |
| GEN1 | 1 |
| GFPT1 | 1 |
| GFRA1 | 1 |
| GFRA4 | 1 |
| GGA3 | 1 |
| GGCT | 1 |
| GGPS1 | 1 |
| GH2 | 1 |
| GIF | 1 |
| GIMAP1 | 1 |
| GIT2 | 1 |
| GJA1 | 1 |
| GJA5 | 1 |
| GJA8 | 1 |
| GJA9 | 1 |
| GJC1 | 1 |
| GJD2 | 1 |
| GJD3 | 1 |
| GK | 1 |
| GLB1L | 1 |
| GLB1L2 | 1 |
| GLB1L3 | 1 |
| GLCE | 1 |
| GLE1 | 1 |
| GLG1 | 1 |
| GLI1 | 1 |
| GLI2 | 1 |
| GLIS1 | 1 |
| GLIS3 | 1 |
| GLO1 | 1 |
| GLRA1 | 1 |
| GLRA3 | 1 |
| GLS2 | 1 |
| GLT25D2 | 1 |
| GLT8D1 | 1 |
| GLUL | 1 |
| GLYAT | 1 |
| GMFB | 1 |
| GML | 1 |
| GNAI2 | 1 |
| GNAZ | 1 |
| GNB2 | 1 |
| GNB5 | 1 |
| GNG11 | 1 |
| GNG5 | 1 |
| GNGT1 | 1 |
| GNGT2 | 1 |
| GNPNAT1 | 1 |
| GNRHR | 1 |
| GNS | 1 |
| GOLGA3 | 1 |
| GOLGA5 | 1 |
| GOLGA6A | 1 |
| GOLGA7 | 1 |
| GOLM1 | 1 |
| GOLPH3 | 1 |
| GOLT1A | 1 |
| GOPC | 1 |
| GORAB | 1 |
| GOSR2 | 1 |
| GOT1 | 1 |
| GP2 | 1 |
| GPAM | 1 |
| GPATCH1 | 1 |
| GPATCH2 | 1 |
| GPC5 | 1 |
| GPI | 1 |
| GPKOW | 1 |
| GPM6B | 1 |
| GPR101 | 1 |
| GPR108 | 1 |
| GPR113 | 1 |
| GPR119 | 1 |
| GPR123 | 1 |
| GPR125 | 1 |
| GPR135 | 1 |
| GPR139 | 1 |
| GPR141 | 1 |
| GPR144 | 1 |
| GPR149 | 1 |
| GPR153 | 1 |
| GPR156 | 1 |
| GPR174 | 1 |
| GPR180 | 1 |
| GPR3 | 1 |
| GPR33 | 1 |
| GPR4 | 1 |
| GPR6 | 1 |
| GPR63 | 1 |
| GPR64 | 1 |
| GPR77 | 1 |
| GPR83 | 1 |
| GPR97 | 1 |
| GPRASP1 | 1 |
| GPRASP2 | 1 |
| GPRC5A | 1 |
| GPRC5B | 1 |
| GPRIN3 | 1 |
| GPSM2 | 1 |
| GRAMD2 | 1 |
| GRB14 | 1 |
| GREB1L | 1 |
| GRHL2 | 1 |
| GRHL3 | 1 |
| GRID1 | 1 |
| GRIK2 | 1 |
| GRIK3 | 1 |
| GRIN2B | 1 |
| GRM2 | 1 |
| GRM5 | 1 |
| GRM7 | 1 |
| GRPEL2 | 1 |
| GRWD1 | 1 |
| GRXCR2 | 1 |
| GSDMA | 1 |
| GSG1 | 1 |
| GSG2 | 1 |
| GSTA1 | 1 |
| GSTA4 | 1 |
| GSTCD | 1 |
| GSTM5 | 1 |
| GSTO1 | 1 |
| GTF2A1L | 1 |
| GTF2E2 | 1 |
| GTF2IRD1 | 1 |
| GTF3C3 | 1 |
| GTPBP2 | 1 |
| GTPBP4 | 1 |
| GTSF1 | 1 |
| GUCA1A | 1 |
| GUCA1B | 1 |
| GUCY2D | 1 |
| GUF1 | 1 |
| GYG1 | 1 |
| GYPC | 1 |
| GZMK | 1 |
| H2AFY | 1 |
| H2AFZ | 1 |
| HADHB | 1 |
| HARS | 1 |
| HARS2 | 1 |
| HAT1 | 1 |
| HAUS3 | 1 |
| HBG2 | 1 |
| HBS1L | 1 |
| HCAR2 | 1 |
| HCRTR1 | 1 |
| HDAC2 | 1 |
| HDAC3 | 1 |
| HDAC7 | 1 |
| HDGFL1 | 1 |
| HDHD2 | 1 |
| HDHD3 | 1 |
| HDX | 1 |
| HEATR1 | 1 |
| HEATR5A | 1 |
| HEATR7A | 1 |
| HEATR7B2 | 1 |
| HECA | 1 |
| HECTD3 | 1 |
| HELB | 1 |
| HELQ | 1 |
| HEMGN | 1 |
| HEMK1 | 1 |
| HEPH | 1 |
| HEPHL1 | 1 |
| HERC2 | 1 |
| HERC6 | 1 |
| HEXB | 1 |
| HEXDC | 1 |
| HEY1 | 1 |
| HEY2 | 1 |
| HGSNAT | 1 |
| HIAT1 | 1 |
| HIC1 | 1 |
| HIF1A | 1 |
| HIGD1B | 1 |
| HIRA | 1 |
| HIST1H1E | 1 |
| HIST1H2AA | 1 |
| HIST1H2BC | 1 |
| HIST1H2BE | 1 |
| HIST1H2BF | 1 |
| HIST1H3H | 1 |
| HIST1H4G | 1 |
| HIST2H2AB | 1 |
| HIST2H3D | 1 |
| HIST3H3 | 1 |
| HJURP | 1 |
| HK1 | 1 |
| HKDC1 | 1 |
| HLA-E | 1 |
| HLTF | 1 |
| HLX | 1 |
| HMBOX1 | 1 |
| HMBS | 1 |
| HMGA2 | 1 |
| HMGB4 | 1 |
| HMGCL | 1 |
| HMGCLL1 | 1 |
| HMGCS2 | 1 |
| HMGXB3 | 1 |
| HMHA1 | 1 |
| HMMR | 1 |
| HNF1B | 1 |
| HNRNPA1 | 1 |
| HNRNPA2B1 | 1 |
| HNRNPAB | 1 |
| HNRNPC | 1 |
| HNRNPCL1 | 1 |
| HNRNPH1 | 1 |
| HNRNPK | 1 |
| HNRNPL | 1 |
| HNRNPR | 1 |
| HOMER2 | 1 |
| HOMEZ | 1 |
| HOPX | 1 |
| HORMAD1 | 1 |
| HOXB1 | 1 |
| HOXB9 | 1 |
| HOXC9 | 1 |
| HOXD11 | 1 |
| HOXD4 | 1 |
| HOXD8 | 1 |
| HP1BP3 | 1 |
| HPD | 1 |
| HPR | 1 |
| HPRT1 | 1 |
| HPSE | 1 |
| HRCT1 | 1 |
| HRH2 | 1 |
| HRH3 | 1 |
| HS3ST1 | 1 |
| HS3ST2 | 1 |
| HS6ST1 | 1 |
| HSD11B2 | 1 |
| HSD17B12 | 1 |
| HSD17B8 | 1 |
| HSD3B1 | 1 |
| HSDL1 | 1 |
| HSDL2 | 1 |
| HSF2BP | 1 |
| HSF4 | 1 |
| HSF5 | 1 |
| HSH2D | 1 |
| HSP90AA1 | 1 |
| HSP90AA4P | 1 |
| HSP90AB1 | 1 |
| HSPA12A | 1 |
| HSPA13 | 1 |
| HSPA14 | 1 |
| HSPA5 | 1 |
| HSPBAP1 | 1 |
| HSPD1 | 1 |
| HTR1A | 1 |
| HTR2A | 1 |
| HTR2C | 1 |
| HTR3A | 1 |
| HTR3C | 1 |
| HTR3E | 1 |
| HTR7 | 1 |
| HTRA1 | 1 |
| HTRA3 | 1 |
| IAPP | 1 |
| IBA57 | 1 |
| IBSP | 1 |
| ICAM4 | 1 |
| IDH3A | 1 |
| IDO1 | 1 |
| IDO2 | 1 |
| IER2 | 1 |
| IFFO1 | 1 |
| IFI44 | 1 |
| IFI44L | 1 |
| IFIH1 | 1 |
| IFIT5 | 1 |
| IFLTD1 | 1 |
| IFNA10 | 1 |
| IFNA7 | 1 |
| IFNAR2 | 1 |
| IFNG | 1 |
| IFNW1 | 1 |
| IFT122 | 1 |
| IFT140 | 1 |
| IFT172 | 1 |
| IFT74 | 1 |
| IGF2 | 1 |
| IGF2BP2 | 1 |
| IGF2R | 1 |
| IGFBP3 | 1 |
| IGFBP7 | 1 |
| IGFL2 | 1 |
| IGHG1 | 1 |
| IGHV3-7 | 1 |
| IGHV5-51 | 1 |
| IGLC1 | 1 |
| IGLL5 | 1 |
| IGLV11-55 | 1 |
| IGLV3-22 | 1 |
| IGSF11 | 1 |
| IGSF22 | 1 |
| IGSF9 | 1 |
| IGSF9B | 1 |
| IKZF2 | 1 |
| IL11 | 1 |
| IL12RB1 | 1 |
| IL16 | 1 |
| IL17F | 1 |
| IL17RE | 1 |
| IL17REL | 1 |
| IL1B | 1 |
| IL1RAP | 1 |
| IL1RAPL2 | 1 |
| IL1RL1 | 1 |
| IL1RL2 | 1 |
| IL20RB | 1 |
| IL21 | 1 |
| IL23R | 1 |
| IL2RB | 1 |
| IL31 | 1 |
| IL4R | 1 |
| ILDR1 | 1 |
| ILDR2 | 1 |
| IMMP2L | 1 |
| IMMT | 1 |
| IMPA2 | 1 |
| IMPDH1 | 1 |
| IMPG1 | 1 |
| ING5 | 1 |
| INHBA | 1 |
| INHBC | 1 |
| INO80B | 1 |
| INPP4A | 1 |
| INPP5A | 1 |
| INSM1 | 1 |
| INTS1 | 1 |
| INTS7 | 1 |
| INTS8 | 1 |
| INTU | 1 |
| IP6K1 | 1 |
| IP6K2 | 1 |
| IPCEF1 | 1 |
| IPMK | 1 |
| IPO4 | 1 |
| IPO5 | 1 |
| IPO7 | 1 |
| IPO8 | 1 |
| IPO9 | 1 |
| IPPK | 1 |
| IQCC | 1 |
| IQCJ-SCHIP1 | 1 |
| IQGAP1 | 1 |
| IRAK1 | 1 |
| IRAK1BP1 | 1 |
| IREB2 | 1 |
| IRF9 | 1 |
| IRX2 | 1 |
| IRX6 | 1 |
| ISM1 | 1 |
| ISY1-RAB43 | 1 |
| ITGA10 | 1 |
| ITGA11 | 1 |
| ITGA3 | 1 |
| ITGA4 | 1 |
| ITGA5 | 1 |
| ITGA9 | 1 |
| ITGAX | 1 |
| ITGB2 | 1 |
| ITGB5 | 1 |
| ITGB6 | 1 |
| ITK | 1 |
| ITLN1 | 1 |
| ITM2A | 1 |
| ITPRIP | 1 |
| IVL | 1 |
| IWS1 | 1 |
| IZUMO1 | 1 |
| IZUMO2 | 1 |
| JAG1 | 1 |
| JAKMIP1 | 1 |
| JAKMIP2 | 1 |
| JAM2 | 1 |
| JMJD1C | 1 |
| JMJD8 | 1 |
| JMY | 1 |
| JPH2 | 1 |
| KAL1 | 1 |
| KALRN | 1 |
| KARS | 1 |
| KAT6B | 1 |
| KATNAL1 | 1 |
| KAZALD1 | 1 |
| KAZN | 1 |
| KBTBD7 | 1 |
| KBTBD8 | 1 |
| KCMF1 | 1 |
| KCNA3 | 1 |
| KCNA6 | 1 |
| KCNA7 | 1 |
| KCNAB1 | 1 |
| KCNAB2 | 1 |
| KCNAB3 | 1 |
| KCNC2 | 1 |
| KCNC4 | 1 |
| KCNE4 | 1 |
| KCNF1 | 1 |
| KCNG4 | 1 |
| KCNH1 | 1 |
| KCNH2 | 1 |
| KCNH5 | 1 |
| KCNH6 | 1 |
| KCNIP2 | 1 |
| KCNIP4 | 1 |
| KCNJ6 | 1 |
| KCNJ9 | 1 |
| KCNK17 | 1 |
| KCNK5 | 1 |
| KCNK7 | 1 |
| KCNMA1 | 1 |
| KCNMB2 | 1 |
| KCNN2 | 1 |
| KCNN3 | 1 |
| KCNQ2 | 1 |
| KCNS2 | 1 |
| KCNV1 | 1 |
| KCNV2 | 1 |
| KCP | 1 |
| KCTD20 | 1 |
| KCTD8 | 1 |
| KCTD9 | 1 |
| KDELC1 | 1 |
| KDELC2 | 1 |
| KDM2B | 1 |
| KDM3B | 1 |
| KDM4A | 1 |
| KDM4B | 1 |
| KDM4C | 1 |
| KDM4D | 1 |
| KDM5D | 1 |
| KHK | 1 |
| KHNYN | 1 |
| KHSRP | 1 |
| KIAA0100 | 1 |
| KIAA0226L | 1 |
| KIAA0247 | 1 |
| KIAA0317 | 1 |
| KIAA0355 | 1 |
| KIAA0368 | 1 |
| KIAA0391 | 1 |
| KIAA0408 | 1 |
| KIAA0430 | 1 |
| KIAA0513 | 1 |
| KIAA0528 | 1 |
| KIAA0664 | 1 |
| KIAA0753 | 1 |
| KIAA0895 | 1 |
| KIAA0947 | 1 |
| KIAA1024 | 1 |
| KIAA1033 | 1 |
| KIAA1199 | 1 |
| KIAA1211 | 1 |
| KIAA1244 | 1 |
| KIAA1324 | 1 |
| KIAA1383 | 1 |
| KIAA1432 | 1 |
| KIAA1462 | 1 |
| KIAA1524 | 1 |
| KIAA1598 | 1 |
| KIAA1644 | 1 |
| KIAA1683 | 1 |
| KIAA1715 | 1 |
| KIAA1841 | 1 |
| KIAA1958 | 1 |
| KIAA1967 | 1 |
| KIAA2018 | 1 |
| KIAA2026 | 1 |
| KIF11 | 1 |
| KIF13B | 1 |
| KIF14 | 1 |
| KIF18A | 1 |
| KIF20B | 1 |
| KIF27 | 1 |
| KIF2A | 1 |
| KIF2B | 1 |
| KIF2C | 1 |
| KIF3B | 1 |
| KIF3C | 1 |
| KIF5B | 1 |
| KIR2DL4 | 1 |
| KIR2DS4 | 1 |
| KIR3DX1 | 1 |
| KIT | 1 |
| KITLG | 1 |
| KLC2 | 1 |
| KLC4 | 1 |
| KLF10 | 1 |
| KLF11 | 1 |
| KLF7 | 1 |
| KLHDC1 | 1 |
| KLHDC2 | 1 |
| KLHDC3 | 1 |
| KLHDC7B | 1 |
| KLHDC9 | 1 |
| KLHL18 | 1 |
| KLHL2 | 1 |
| KLHL21 | 1 |
| KLHL22 | 1 |
| KLHL25 | 1 |
| KLHL3 | 1 |
| KLHL32 | 1 |
| KLHL38 | 1 |
| KLHL5 | 1 |
| KLHL9 | 1 |
| KLK10 | 1 |
| KLK14 | 1 |
| KLK2 | 1 |
| KLK4 | 1 |
| KLK8 | 1 |
| KLRC4 | 1 |
| KLRF1 | 1 |
| KLRF2 | 1 |
| KNDC1 | 1 |
| KNTC1 | 1 |
| KPNA1 | 1 |
| KPNA5 | 1 |
| KPNA6 | 1 |
| KPRP | 1 |
| KREMEN1 | 1 |
| KRI1 | 1 |
| KRIT1 | 1 |
| KRR1 | 1 |
| KRT1 | 1 |
| KRT12 | 1 |
| KRT15 | 1 |
| KRT35 | 1 |
| KRT38 | 1 |
| KRT4 | 1 |
| KRT6A | 1 |
| KRT6C | 1 |
| KRT7 | 1 |
| KRT8 | 1 |
| KRT81 | 1 |
| KRT84 | 1 |
| KRT85 | 1 |
| KRT9 | 1 |
| KRTAP13-2 | 1 |
| KRTAP13-3 | 1 |
| KRTAP13-4 | 1 |
| KRTAP21-1 | 1 |
| KRTAP25-1 | 1 |
| KRTAP27-1 | 1 |
| KRTAP4-12 | 1 |
| KRTAP6-2 | 1 |
| KRTCAP2 | 1 |
| KRTCAP3 | 1 |
| KTI12 | 1 |
| KTN1 | 1 |
| L1CAM | 1 |
| L3MBTL4 | 1 |
| LACTB2 | 1 |
| LAIR2 | 1 |
| LAMA5 | 1 |
| LAMP1 | 1 |
| LANCL2 | 1 |
| LAP3 | 1 |
| LARP1B | 1 |
| LARP7 | 1 |
| LARS2 | 1 |
| LAT2 | 1 |
| LAX1 | 1 |
| LAYN | 1 |
| LBP | 1 |
| LCE1B | 1 |
| LCE1D | 1 |
| LCE1E | 1 |
| LCE2A | 1 |
| LCE3D | 1 |
| LCE6A | 1 |
| LCK | 1 |
| LCN8 | 1 |
| LDB3 | 1 |
| LDLR | 1 |
| LEF1 | 1 |
| LEPR | 1 |
| LEPRE1 | 1 |
| LGALS12 | 1 |
| LGALS3 | 1 |
| LGALS8 | 1 |
| LGALS9 | 1 |
| LGALSL | 1 |
| LGI1 | 1 |
| LGI3 | 1 |
| LGR4 | 1 |
| LGR5 | 1 |
| LHCGR | 1 |
| LHFP | 1 |
| LHFPL1 | 1 |
| LHX1 | 1 |
| LHX4 | 1 |
| LHX5 | 1 |
| LIG3 | 1 |
| LILRA5 | 1 |
| LILRA6 | 1 |
| LILRB3 | 1 |
| LIMCH1 | 1 |
| LIME1 | 1 |
| LIN28B | 1 |
| LIN7A | 1 |
| LINGO4 | 1 |
| LINS | 1 |
| LIPA | 1 |
| LIPH | 1 |
| LITAF | 1 |
| LLGL1 | 1 |
| LLGL2 | 1 |
| LMCD1 | 1 |
| LMO7 | 1 |
| LMOD2 | 1 |
| LMOD3 | 1 |
| LNPEP | 1 |
| LOC100129520 | 1 |
| LOC147670 | 1 |
| LOC402160 | 1 |
| LOC646498 | 1 |
| LOC728819 | 1 |
| LONRF1 | 1 |
| LOXL3 | 1 |
| LPA | 1 |
| LPAR3 | 1 |
| LPCAT2 | 1 |
| LPCAT3 | 1 |
| LPCAT4 | 1 |
| LPHN1 | 1 |
| LPIN3 | 1 |
| LPL | 1 |
| LPP | 1 |
| LPPR4 | 1 |
| LPXN | 1 |
| LRFN1 | 1 |
| LRFN3 | 1 |
| LRIF1 | 1 |
| LRIT3 | 1 |
| LRP10 | 1 |
| LRP11 | 1 |
| LRP4 | 1 |
| LRP6 | 1 |
| LRRC15 | 1 |
| LRRC32 | 1 |
| LRRC36 | 1 |
| LRRC37A3 | 1 |
| LRRC41 | 1 |
| LRRC57 | 1 |
| LRRC6 | 1 |
| LRRC61 | 1 |
| LRRC73 | 1 |
| LRRC8C | 1 |
| LRRC8D | 1 |
| LRRC8E | 1 |
| LRRCC1 | 1 |
| LRTM1 | 1 |
| LSAMP | 1 |
| LSM11 | 1 |
| LSM3 | 1 |
| LSP1 | 1 |
| LTA4H | 1 |
| LTBP3 | 1 |
| LTBP4 | 1 |
| LTK | 1 |
| LTN1 | 1 |
| LUC7L2 | 1 |
| LY75-CD302 | 1 |
| LYAR | 1 |
| LYG2 | 1 |
| LYNX1 | 1 |
| LZTS1 | 1 |
| LZTS2 | 1 |
| M6PR | 1 |
| MAB21L1 | 1 |
| MAFA | 1 |
| MAGEA12 | 1 |
| MAGEA3 | 1 |
| MAGEA4 | 1 |
| MAGEA8 | 1 |
| MAGEB1 | 1 |
| MAGEB18 | 1 |
| MAGEB2 | 1 |
| MAGEB6 | 1 |
| MAGEC3 | 1 |
| MAGEL2 | 1 |
| MAGI2 | 1 |
| MAK | 1 |
| MAL | 1 |
| MAMDC4 | 1 |
| MAML3 | 1 |
| MAN1A2 | 1 |
| MAN1B1 | 1 |
| MAN2A1 | 1 |
| MAN2B2 | 1 |
| MANEA | 1 |
| MANEAL | 1 |
| MANSC1 | 1 |
| MAOA | 1 |
| MAOB | 1 |
| MAP1LC3C | 1 |
| MAP2K2 | 1 |
| MAP2K3 | 1 |
| MAP3K1 | 1 |
| MAP3K10 | 1 |
| MAP3K11 | 1 |
| MAP3K3 | 1 |
| MAP3K6 | 1 |
| MAP3K7 | 1 |
| MAP3K8 | 1 |
| MAP4 | 1 |
| MAP4K4 | 1 |
| MAP4K5 | 1 |
| MAPK15 | 1 |
| MAPK4 | 1 |
| MAPK6 | 1 |
| MAPK7 | 1 |
| MAPK8IP1 | 1 |
| MAPK8IP2 | 1 |
| MAPK9 | 1 |
| MAPKAPK3 | 1 |
| MAPRE3 | 1 |
| MAPT | 1 |
| 10-Mar | 1 |
| 11-Mar | 1 |
| 3-Mar | 1 |
| 7-Mar | 1 |
| MARK1 | 1 |
| MARVELD2 | 1 |
| MASP2 | 1 |
| MASTL | 1 |
| MATN1 | 1 |
| MATN2 | 1 |
| MATN3 | 1 |
| MATR3 | 1 |
| MAVS | 1 |
| MB | 1 |
| MBD3L1 | 1 |
| MBLAC2 | 1 |
| MBOAT2 | 1 |
| MC2R | 1 |
| MC3R | 1 |
| MC5R | 1 |
| MCCC1 | 1 |
| MCF2 | 1 |
| MCHR2 | 1 |
| MCL1 | 1 |
| MCM10 | 1 |
| MCM2 | 1 |
| MCM3 | 1 |
| MCM3AP | 1 |
| MCM8 | 1 |
| MDC1 | 1 |
| MDFI | 1 |
| MDFIC | 1 |
| MDH1 | 1 |
| MDH1B | 1 |
| MDM1 | 1 |
| ME2 | 1 |
| ME3 | 1 |
| MEAF6 | 1 |
| MECR | 1 |
| MED1 | 1 |
| MED13 | 1 |
| MED17 | 1 |
| MED23 | 1 |
| MED6 | 1 |
| MED7 | 1 |
| MEF2A | 1 |
| MEGF6 | 1 |
| MEGF9 | 1 |
| MEOX1 | 1 |
| MEPCE | 1 |
| METAP1 | 1 |
| METAP2 | 1 |
| METTL16 | 1 |
| METTL18 | 1 |
| METTL21A | 1 |
| METTL21D | 1 |
| METTL4 | 1 |
| METTL7B | 1 |
| MFF | 1 |
| MFN2 | 1 |
| MFRP | 1 |
| MFSD1 | 1 |
| MFSD11 | 1 |
| MFSD2A | 1 |
| MFSD4 | 1 |
| MFSD5 | 1 |
| MFSD8 | 1 |
| MGA | 1 |
| MGAM | 1 |
| MGAT5 | 1 |
| MGLL | 1 |
| MGRN1 | 1 |
| MIA2 | 1 |
| MICA | 1 |
| MICAL2 | 1 |
| MICALCL | 1 |
| MICALL1 | 1 |
| MIER3 | 1 |
| MIIP | 1 |
| MIOS | 1 |
| MIPEP | 1 |
| MIR1279 | 1 |
| MIR4461 | 1 |
| MIS18BP1 | 1 |
| MITF | 1 |
| MKKS | 1 |
| MKL2 | 1 |
| MKLN1 | 1 |
| MKNK2 | 1 |
| MKX | 1 |
| MLEC | 1 |
| MLF1 | 1 |
| MLF1IP | 1 |
| MLH1 | 1 |
| MLKL | 1 |
| MLL2 | 1 |
| MLL4 | 1 |
| MLLT10 | 1 |
| MLLT6 | 1 |
| MLPH | 1 |
| MLXIPL | 1 |
| MMAA | 1 |
| MMAB | 1 |
| MMGT1 | 1 |
| MMP1 | 1 |
| MMP11 | 1 |
| MMP13 | 1 |
| MMP14 | 1 |
| MMP17 | 1 |
| MMP2 | 1 |
| MMP25 | 1 |
| MMP28 | 1 |
| MMP3 | 1 |
| MMP8 | 1 |
| MMP9 | 1 |
| MOB4 | 1 |
| MOCOS | 1 |
| MOGAT3 | 1 |
| MORF4L1 | 1 |
| MORN1 | 1 |
| MORN4 | 1 |
| MOXD1 | 1 |
| MPDU1 | 1 |
| MPEG1 | 1 |
| MPI | 1 |
| MPO | 1 |
| MPP2 | 1 |
| MPP4 | 1 |
| MPPED2 | 1 |
| MPV17 | 1 |
| MRGPRD | 1 |
| MRGPRE | 1 |
| MRGPRX2 | 1 |
| MRPL11 | 1 |
| MRPL16 | 1 |
| MRPL24 | 1 |
| MRPL37 | 1 |
| MRPL39 | 1 |
| MRPL43 | 1 |
| MRPL45 | 1 |
| MRPL46 | 1 |
| MRPS15 | 1 |
| MRPS22 | 1 |
| MRPS36 | 1 |
| MRPS5 | 1 |
| MS4A14 | 1 |
| MS4A3 | 1 |
| MS4A5 | 1 |
| MS4A7 | 1 |
| MSH2 | 1 |
| MSI1 | 1 |
| MSI2 | 1 |
| MSLNL | 1 |
| MSR1 | 1 |
| MSRA | 1 |
| MST1R | 1 |
| MT1E | 1 |
| MTA1 | 1 |
| MTA2 | 1 |
| MTCH1 | 1 |
| MTDH | 1 |
| MTERF | 1 |
| MTF1 | 1 |
| MTG1 | 1 |
| MTHFD1 | 1 |
| MTHFD2L | 1 |
| MTHFS | 1 |
| MTHFSD | 1 |
| MTMR2 | 1 |
| MTMR4 | 1 |
| MTMR7 | 1 |
| MTO1 | 1 |
| MTOR | 1 |
| MTRR | 1 |
| MTSS1 | 1 |
| MTSS1L | 1 |
| MTTP | 1 |
| MTUS1 | 1 |
| MUC12 | 1 |
| MUC15 | 1 |
| MUC20 | 1 |
| MUC21 | 1 |
| MUL1 | 1 |
| MURC | 1 |
| MUT | 1 |
| MVP | 1 |
| MYADML2 | 1 |
| MYBL2 | 1 |
| MYBPC2 | 1 |
| MYBPHL | 1 |
| MYCBP2 | 1 |
| MYCL1 | 1 |
| MYF6 | 1 |
| MYH15 | 1 |
| MYL12A | 1 |
| MYNN | 1 |
| MYO15B | 1 |
| MYO16 | 1 |
| MYO19 | 1 |
| MYO1A | 1 |
| MYO1B | 1 |
| MYO1D | 1 |
| MYO1E | 1 |
| MYO1H | 1 |
| MYO5B | 1 |
| MYO7A | 1 |
| MYOD1 | 1 |
| MYOT | 1 |
| MYPN | 1 |
| MYRIP | 1 |
| MYSM1 | 1 |
| N4BP2L2 | 1 |
| NAA15 | 1 |
| NAA35 | 1 |
| NAA60 | 1 |
| NAALADL1 | 1 |
| NAALADL2 | 1 |
| NAB2 | 1 |
| NACA2 | 1 |
| NAE1 | 1 |
| NAGS | 1 |
| NAIF1 | 1 |
| NAMPTL | 1 |
| NANOG | 1 |
| NAP1L2 | 1 |
| NAP1L3 | 1 |
| NARG2 | 1 |
| NARS2 | 1 |
| NBEAL2 | 1 |
| NCAN | 1 |
| NCAPG | 1 |
| NCBP1 | 1 |
| NCCRP1 | 1 |
| NCEH1 | 1 |
| NCKAP1L | 1 |
| NCKAP5 | 1 |
| NCL | 1 |
| NCOA2 | 1 |
| NCOA5 | 1 |
| NCOR2 | 1 |
| NCR2 | 1 |
| NCR3 | 1 |
| NDN | 1 |
| NDRG1 | 1 |
| NDRG2 | 1 |
| NDRG3 | 1 |
| NDST2 | 1 |
| NDST4 | 1 |
| NDUFAF3 | 1 |
| NDUFS1 | 1 |
| NDUFS2 | 1 |
| NDUFS3 | 1 |
| NECAB1 | 1 |
| NECAP1 | 1 |
| NEDD1 | 1 |
| NEDD4 | 1 |
| NEDD4L | 1 |
| NEGR1 | 1 |
| NEK1 | 1 |
| NEK2 | 1 |
| NEK8 | 1 |
| NELL1 | 1 |
| NEMF | 1 |
| NEO1 | 1 |
| NES | 1 |
| NET1 | 1 |
| NETO1 | 1 |
| NETO2 | 1 |
| NEU2 | 1 |
| NEU4 | 1 |
| NEURL | 1 |
| NEUROD4 | 1 |
| NEUROD6 | 1 |
| NEUROG2 | 1 |
| NEUROG3 | 1 |
| NEXN | 1 |
| NF2 | 1 |
| NFATC1 | 1 |
| NFATC3 | 1 |
| NFE2L1 | 1 |
| NFKB1 | 1 |
| NFKBIA | 1 |
| NFKBIL1 | 1 |
| NFRKB | 1 |
| NFU1 | 1 |
| NGEF | 1 |
| NGFR | 1 |
| NHP2L1 | 1 |
| NHSL2 | 1 |
| NID1 | 1 |
| NIF3L1 | 1 |
| NIPAL1 | 1 |
| NIPSNAP3A | 1 |
| NKAIN3 | 1 |
| NKAPL | 1 |
| NKPD1 | 1 |
| NKX2-1 | 1 |
| NLE1 | 1 |
| NLK | 1 |
| NLRC3 | 1 |
| NLRP12 | 1 |
| NLRP13 | 1 |
| NLRP2 | 1 |
| NLRP4 | 1 |
| NLRP8 | 1 |
| NME9 | 1 |
| NMI | 1 |
| NMUR1 | 1 |
| NNMT | 1 |
| NOB1 | 1 |
| NOD1 | 1 |
| NODAL | 1 |
| NOL6 | 1 |
| NOL9 | 1 |
| NOLC1 | 1 |
| NOM1 | 1 |
| NOMO1 | 1 |
| NOMO2 | 1 |
| NOP14 | 1 |
| NOS1AP | 1 |
| NOS2 | 1 |
| NOTCH4 | 1 |
| NOTO | 1 |
| NOVA2 | 1 |
| NPAS1 | 1 |
| NPAS2 | 1 |
| NPAS4 | 1 |
| NPC1 | 1 |
| NPEPPS | 1 |
| NPFFR1 | 1 |
| NPFFR2 | 1 |
| NPHP3 | 1 |
| NPHP4 | 1 |
| NPL | 1 |
| NPLOC4 | 1 |
| NPPA | 1 |
| NPR1 | 1 |
| NPTX2 | 1 |
| NPVF | 1 |
| NQO1 | 1 |
| NR1H3 | 1 |
| NR1I2 | 1 |
| NR1I3 | 1 |
| NR2C1 | 1 |
| NR2E1 | 1 |
| NR2F2 | 1 |
| NR2F6 | 1 |
| NR3C1 | 1 |
| NR4A3 | 1 |
| NR5A2 | 1 |
| NRAP | 1 |
| NRD1 | 1 |
| NRG1 | 1 |
| NRG3 | 1 |
| NRIP1 | 1 |
| NRK | 1 |
| NRP2 | 1 |
| NRSN1 | 1 |
| NRXN2 | 1 |
| NSD1 | 1 |
| NSF | 1 |
| NSRP1 | 1 |
| NSUN2 | 1 |
| NSUN4 | 1 |
| NSUN7 | 1 |
| NT5C1A | 1 |
| NTN3 | 1 |
| NTRK1 | 1 |
| NTRK3 | 1 |
| NTSR2 | 1 |
| NUAK2 | 1 |
| NUDCD1 | 1 |
| NUDT12 | 1 |
| NUDT2 | 1 |
| NUF2 | 1 |
| NUP153 | 1 |
| NUP160 | 1 |
| NUP188 | 1 |
| NUP210L | 1 |
| NUP214 | 1 |
| NUP43 | 1 |
| NUP98 | 1 |
| NUS1 | 1 |
| NXF1 | 1 |
| NXF3 | 1 |
| NXN | 1 |
| NXPH1 | 1 |
| NXT2 | 1 |
| O3FAR1 | 1 |
| OAS2 | 1 |
| OAS3 | 1 |
| OAZ2 | 1 |
| OCIAD2 | 1 |
| ODC1 | 1 |
| OFD1 | 1 |
| OGFOD2 | 1 |
| OGFRL1 | 1 |
| OIT3 | 1 |
| OLAH | 1 |
| OLFM1 | 1 |
| OLFM2 | 1 |
| OMA1 | 1 |
| ONECUT1 | 1 |
| OPN4 | 1 |
| OPRM1 | 1 |
| OPTC | 1 |
| OR10A2 | 1 |
| OR10A7 | 1 |
| OR10AG1 | 1 |
| OR10G7 | 1 |
| OR10G8 | 1 |
| OR10H4 | 1 |
| OR10H5 | 1 |
| OR10J1 | 1 |
| OR10J3 | 1 |
| OR10K2 | 1 |
| OR10V1 | 1 |
| OR11H6 | 1 |
| OR12D2 | 1 |
| OR13C3 | 1 |
| OR13J1 | 1 |
| OR1A1 | 1 |
| OR1B1 | 1 |
| OR1D2 | 1 |
| OR1E1 | 1 |
| OR1E2 | 1 |
| OR1L8 | 1 |
| OR1Q1 | 1 |
| OR1S1 | 1 |
| OR2A14 | 1 |
| OR2A5 | 1 |
| OR2AJ1 | 1 |
| OR2AK2 | 1 |
| OR2AP1 | 1 |
| OR2B11 | 1 |
| OR2C1 | 1 |
| OR2C3 | 1 |
| OR2F2 | 1 |
| OR2G2 | 1 |
| OR2G3 | 1 |
| OR2G6 | 1 |
| OR2J2 | 1 |
| OR2J3 | 1 |
| OR2L2 | 1 |
| OR2L3 | 1 |
| OR2L5 | 1 |
| OR2M3 | 1 |
| OR2T1 | 1 |
| OR2T12 | 1 |
| OR2T27 | 1 |
| OR2Y1 | 1 |
| OR2Z1 | 1 |
| OR3A1 | 1 |
| OR4A15 | 1 |
| OR4A16 | 1 |
| OR4A47 | 1 |
| OR4A5 | 1 |
| OR4C5 | 1 |
| OR4D11 | 1 |
| OR4F6 | 1 |
| OR4K1 | 1 |
| OR4K5 | 1 |
| OR4M2 | 1 |
| OR4N2 | 1 |
| OR4N5 | 1 |
| OR4X1 | 1 |
| OR51A7 | 1 |
| OR51B2 | 1 |
| OR51B5 | 1 |
| OR51B6 | 1 |
| OR51D1 | 1 |
| OR51E1 | 1 |
| OR51E2 | 1 |
| OR51G2 | 1 |
| OR51J1 | 1 |
| OR51Q1 | 1 |
| OR52A1 | 1 |
| OR52B4 | 1 |
| OR52E4 | 1 |
| OR52E6 | 1 |
| OR52I1 | 1 |
| OR52I2 | 1 |
| OR52J3 | 1 |
| OR52K1 | 1 |
| OR52N5 | 1 |
| OR52R1 | 1 |
| OR56A3 | 1 |
| OR56A4 | 1 |
| OR5A2 | 1 |
| OR5AC2 | 1 |
| OR5AQ1P | 1 |
| OR5AR1 | 1 |
| OR5AS1 | 1 |
| OR5B17 | 1 |
| OR5B3 | 1 |
| OR5H15 | 1 |
| OR5H7P | 1 |
| OR5I1 | 1 |
| OR5J2 | 1 |
| OR5L2 | 1 |
| OR5M1 | 1 |
| OR5M10 | 1 |
| OR5M3 | 1 |
| OR5M8 | 1 |
| OR5M9 | 1 |
| OR5T1 | 1 |
| OR5T2 | 1 |
| OR6B1 | 1 |
| OR6B3 | 1 |
| OR6C1 | 1 |
| OR6C2 | 1 |
| OR6C3 | 1 |
| OR6C65 | 1 |
| OR6C70 | 1 |
| OR6C74 | 1 |
| OR6C76 | 1 |
| OR6K2 | 1 |
| OR6M1 | 1 |
| OR6V1 | 1 |
| OR6Y1 | 1 |
| OR7A2P | 1 |
| OR7A5 | 1 |
| OR7G1 | 1 |
| OR7G3 | 1 |
| OR8B8 | 1 |
| OR8D2 | 1 |
| OR8D4 | 1 |
| OR8G1 | 1 |
| OR8G5 | 1 |
| OR8H1 | 1 |
| OR8H2 | 1 |
| OR8K1 | 1 |
| OR8S1 | 1 |
| OR9Q1 | 1 |
| ORAI1 | 1 |
| ORC3 | 1 |
| ORM1 | 1 |
| ORMDL3 | 1 |
| OSBP2 | 1 |
| OSBPL10 | 1 |
| OSBPL1A | 1 |
| OSBPL6 | 1 |
| OSCAR | 1 |
| OSGEP | 1 |
| OTOA | 1 |
| OTOL1 | 1 |
| OTUD6A | 1 |
| OTUD6B | 1 |
| OTUD7A | 1 |
| OXNAD1 | 1 |
| OXR1 | 1 |
| P2RX2 | 1 |
| P2RY10 | 1 |
| P2RY6 | 1 |
| P4HA1 | 1 |
| PABPN1L | 1 |
| PACS1 | 1 |
| PADI1 | 1 |
| PADI3 | 1 |
| PAFAH1B1 | 1 |
| PAFAH1B3 | 1 |
| PAGE2 | 1 |
| PAICS | 1 |
| PAIP1 | 1 |
| PAK1 | 1 |
| PAK2 | 1 |
| PAK3 | 1 |
| PAK4 | 1 |
| PAK6 | 1 |
| PAK7 | 1 |
| PALM2 | 1 |
| PALM2-AKAP2 | 1 |
| PALM3 | 1 |
| PALMD | 1 |
| PANK2 | 1 |
| PAOX | 1 |
| PAPD5 | 1 |
| PAPL | 1 |
| PAPOLA | 1 |
| PAPOLG | 1 |
| PAQR5 | 1 |
| PAQR7 | 1 |
| PARM1 | 1 |
| PARN | 1 |
| PARP1 | 1 |
| PARP14 | 1 |
| PARP15 | 1 |
| PARP2 | 1 |
| PARP3 | 1 |
| PARP6 | 1 |
| PARP9 | 1 |
| PASD1 | 1 |
| PATE3 | 1 |
| PATL1 | 1 |
| PAX1 | 1 |
| PAX5 | 1 |
| PAX6 | 1 |
| PBK | 1 |
| PBLD | 1 |
| PBX3 | 1 |
| PBX4 | 1 |
| PBXIP1 | 1 |
| PC | 1 |
| PCBP3 | 1 |
| PCCA | 1 |
| PCCB | 1 |
| PCDH11Y | 1 |
| PCDHA10 | 1 |
| PCDHA11 | 1 |
| PCDHA13 | 1 |
| PCDHA3 | 1 |
| PCDHA5 | 1 |
| PCDHA7 | 1 |
| PCDHA9 | 1 |
| PCDHB12 | 1 |
| PCDHB15 | 1 |
| PCDHB16 | 1 |
| PCDHB18 | 1 |
| PCDHB5 | 1 |
| PCDHB6 | 1 |
| PCDHB7 | 1 |
| PCDHB9 | 1 |
| PCDHGA12 | 1 |
| PCDHGA6 | 1 |
| PCDHGA9 | 1 |
| PCDHGB1 | 1 |
| PCDHGB3 | 1 |
| PCDHGB4 | 1 |
| PCDHGB6 | 1 |
| PCDHGB7 | 1 |
| PCDHGC5 | 1 |
| PCDP1 | 1 |
| PCF11 | 1 |
| PCGF5 | 1 |
| PCIF1 | 1 |
| PCK1 | 1 |
| PCM1 | 1 |
| PCNX | 1 |
| PCNXL2 | 1 |
| PCSK2 | 1 |
| PCSK9 | 1 |
| PCYOX1L | 1 |
| PDC | 1 |
| PDCD11 | 1 |
| PDCD1LG2 | 1 |
| PDCD6IP | 1 |
| PDCD7 | 1 |
| PDCL3 | 1 |
| PDE11A | 1 |
| PDE1B | 1 |
| PDE1C | 1 |
| PDE2A | 1 |
| PDE3A | 1 |
| PDE4A | 1 |
| PDE4DIP | 1 |
| PDE6B | 1 |
| PDE6H | 1 |
| PDE8A | 1 |
| PDGFD | 1 |
| PDHA2 | 1 |
| PDIA2 | 1 |
| PDK2 | 1 |
| PDLIM5 | 1 |
| PDP1 | 1 |
| PDSS1 | 1 |
| PDXDC1 | 1 |
| PEBP1 | 1 |
| PELO | 1 |
| PELP1 | 1 |
| PENK | 1 |
| PEPD | 1 |
| PER2 | 1 |
| PERP | 1 |
| PEX12 | 1 |
| PEX14 | 1 |
| PEX2 | 1 |
| PEX5 | 1 |
| PEX5L | 1 |
| PF4V1 | 1 |
| PFDN5 | 1 |
| PFKFB2 | 1 |
| PFKFB3 | 1 |
| PFKP | 1 |
| PFN2 | 1 |
| PGAP1 | 1 |
| PGBD1 | 1 |
| PGBD3 | 1 |
| PGLS | 1 |
| PGLYRP3 | 1 |
| PGM2 | 1 |
| PGM5 | 1 |
| PGS1 | 1 |
| PHACTR1 | 1 |
| PHF1 | 1 |
| PHF11 | 1 |
| PHF12 | 1 |
| PHF15 | 1 |
| PHF16 | 1 |
| PHF17 | 1 |
| PHF20L1 | 1 |
| PHF21A | 1 |
| PHF6 | 1 |
| PHF8 | 1 |
| PHGDH | 1 |
| PHKB | 1 |
| PHKG1 | 1 |
| PHLDB2 | 1 |
| PHLDB3 | 1 |
| PHLPP1 | 1 |
| PHTF1 | 1 |
| PHYHIP | 1 |
| PI4KA | 1 |
| PIEZO2 | 1 |
| PIGC | 1 |
| PIGR | 1 |
| PIGS | 1 |
| PIGT | 1 |
| PIGU | 1 |
| PIH1D2 | 1 |
| PIK3CD | 1 |
| PIK3R6 | 1 |
| PIP4K2B | 1 |
| PIP5K1A | 1 |
| PIP5K1C | 1 |
| PIPOX | 1 |
| PISD | 1 |
| PITPNC1 | 1 |
| PITPNM1 | 1 |
| PITPNM2 | 1 |
| PITRM1 | 1 |
| PIWIL3 | 1 |
| PIWIL4 | 1 |
| PKD1L1 | 1 |
| PKD1L3 | 1 |
| PKLR | 1 |
| PKNOX1 | 1 |
| PKP2 | 1 |
| PKP3 | 1 |
| PLA2G3 | 1 |
| PLA2G4A | 1 |
| PLA2G4C | 1 |
| PLA2G4D | 1 |
| PLA2G6 | 1 |
| PLA2G7 | 1 |
| PLA2R1 | 1 |
| PLAC8L1 | 1 |
| PLAG1 | 1 |
| PLCB2 | 1 |
| PLCB3 | 1 |
| PLCL2 | 1 |
| PLCXD3 | 1 |
| PLD3 | 1 |
| PLEK | 1 |
| PLEKHA6 | 1 |
| PLEKHG2 | 1 |
| PLEKHG3 | 1 |
| PLEKHG4 | 1 |
| PLEKHN1 | 1 |
| PLEKHO1 | 1 |
| PLIN2 | 1 |
| PLIN4 | 1 |
| PLK2 | 1 |
| PLK3 | 1 |
| PLOD3 | 1 |
| PLSCR4 | 1 |
| PLTP | 1 |
| PLXNA1 | 1 |
| PLXNB3 | 1 |
| PLXNC1 | 1 |
| PLXND1 | 1 |
| PMS1 | 1 |
| PMVK | 1 |
| PNKD | 1 |
| PNLDC1 | 1 |
| PNMA3 | 1 |
| PNPLA7 | 1 |
| POC1B | 1 |
| POC5 | 1 |
| PODNL1 | 1 |
| POFUT1 | 1 |
| POFUT2 | 1 |
| POLD1 | 1 |
| POLD2 | 1 |
| POLDIP2 | 1 |
| POLK | 1 |
| POLM | 1 |
| POLR1C | 1 |
| POLR2F | 1 |
| POLR2L | 1 |
| POLR3C | 1 |
| POLR3E | 1 |
| POM121 | 1 |
| POM121L2 | 1 |
| POMGNT1 | 1 |
| POMT1 | 1 |
| POMT2 | 1 |
| POP1 | 1 |
| POR | 1 |
| POT1 | 1 |
| POTEA | 1 |
| POTED | 1 |
| POU1F1 | 1 |
| POU2AF1 | 1 |
| POU2F2 | 1 |
| POU3F3 | 1 |
| POU4F1 | 1 |
| POU6F1 | 1 |
| PP2D1 | 1 |
| PPAN | 1 |
| PPAN-P2RY11 | 1 |
| PPARA | 1 |
| PPARD | 1 |
| PPARG | 1 |
| PPARGC1A | 1 |
| PPARGC1B | 1 |
| PPCS | 1 |
| PPFIA3 | 1 |
| PPFIA4 | 1 |
| PPFIBP1 | 1 |
| PPFIBP2 | 1 |
| PPIL3 | 1 |
| PPIL4 | 1 |
| PPL | 1 |
| PPM1H | 1 |
| PPM1M | 1 |
| PPP1R12A | 1 |
| PPP1R13B | 1 |
| PPP1R15B | 1 |
| PPP1R16A | 1 |
| PPP1R1B | 1 |
| PPP1R21 | 1 |
| PPP1R26 | 1 |
| PPP1R3C | 1 |
| PPP1R8 | 1 |
| PPP1R9A | 1 |
| PPP2R2A | 1 |
| PPP2R2C | 1 |
| PPP2R3A | 1 |
| PPP3CB | 1 |
| PPP3CC | 1 |
| PPP4C | 1 |
| PPP4R1L | 1 |
| PPP4R2 | 1 |
| PPP5C | 1 |
| PPP6C | 1 |
| PPP6R1 | 1 |
| PPT1 | 1 |
| PPYR1 | 1 |
| PRAC | 1 |
| PRAMEF1 | 1 |
| PRAMEF4 | 1 |
| PRCC | 1 |
| PRCP | 1 |
| PRDM2 | 1 |
| PRDM7 | 1 |
| PRDX2 | 1 |
| PRG2 | 1 |
| PRKACA | 1 |
| PRKACG | 1 |
| PRKAR1A | 1 |
| PRKAR1B | 1 |
| PRKAR2A | 1 |
| PRKCI | 1 |
| PRKCSH | 1 |
| PRKD2 | 1 |
| PRKD3 | 1 |
| PRKG1 | 1 |
| PRKRIR | 1 |
| PRMT3 | 1 |
| PRMT6 | 1 |
| PRMT8 | 1 |
| PRND | 1 |
| PRODH2 | 1 |
| PROKR1 | 1 |
| PROSC | 1 |
| PRPF3 | 1 |
| PRPF38A | 1 |
| PRPF4 | 1 |
| PRPF4B | 1 |
| PRPF6 | 1 |
| PRPH | 1 |
| PRPS2 | 1 |
| PRR14 | 1 |
| PRR14L | 1 |
| PRR16 | 1 |
| PRR23C | 1 |
| PRR25 | 1 |
| PRR5-ARHGAP8 | 1 |
| PRR5L | 1 |
| PRRC2A | 1 |
| PRRT2 | 1 |
| PRRT4 | 1 |
| PRSS23 | 1 |
| PRSS48 | 1 |
| PSD | 1 |
| PSEN1 | 1 |
| PSENEN | 1 |
| PSG1 | 1 |
| PSKH2 | 1 |
| PSMA5 | 1 |
| PSMA6 | 1 |
| PSMB6 | 1 |
| PSMC1 | 1 |
| PSMC2 | 1 |
| PSMD7 | 1 |
| PSME3 | 1 |
| PSMG2 | 1 |
| PSMG3 | 1 |
| PSTK | 1 |
| PSTPIP1 | 1 |
| PTCD2 | 1 |
| PTCH1 | 1 |
| PTCHD1 | 1 |
| PTCHD2 | 1 |
| PTCRA | 1 |
| PTGDR2 | 1 |
| PTGER2 | 1 |
| PTGER4 | 1 |
| PTGES2 | 1 |
| PTGIR | 1 |
| PTGS1 | 1 |
| PTGS2 | 1 |
| PTH1R | 1 |
| PTMS | 1 |
| PTP4A2 | 1 |
| PTP4A3 | 1 |
| PTPN1 | 1 |
| PTPN11 | 1 |
| PTPN4 | 1 |
| PTPN5 | 1 |
| PTPRA | 1 |
| PTPRG | 1 |
| PTPRH | 1 |
| PTPRN | 1 |
| PTTG2 | 1 |
| PUM2 | 1 |
| PUS7 | 1 |
| PUS7L | 1 |
| PWP1 | 1 |
| PXDN | 1 |
| PXK | 1 |
| PXMP2 | 1 |
| PYCR1 | 1 |
| PYDC2 | 1 |
| PYGO1 | 1 |
| PYROXD1 | 1 |
| QTRTD1 | 1 |
| R3HDM1 | 1 |
| RAB10 | 1 |
| RAB12 | 1 |
| RAB19 | 1 |
| RAB21 | 1 |
| RAB27A | 1 |
| RAB30 | 1 |
| RAB31 | 1 |
| RAB33A | 1 |
| RAB34 | 1 |
| RAB39B | 1 |
| RAB3C | 1 |
| RAB3D | 1 |
| RAB3IL1 | 1 |
| RAB43 | 1 |
| RAB5B | 1 |
| RAB7L1 | 1 |
| RABGGTB | 1 |
| RAD51 | 1 |
| RAG1 | 1 |
| RAI1 | 1 |
| RALGAPA1 | 1 |
| RALGPS1 | 1 |
| RANBP10 | 1 |
| RANBP6 | 1 |
| RANGAP1 | 1 |
| RAP1GDS1 | 1 |
| RAPGEF3 | 1 |
| RAPGEF4 | 1 |
| RAPH1 | 1 |
| RARRES2 | 1 |
| RASA1 | 1 |
| RASA2 | 1 |
| RASA3 | 1 |
| RASAL2 | 1 |
| RASEF | 1 |
| RASGEF1A | 1 |
| RASGEF1B | 1 |
| RASSF2 | 1 |
| RB1CC1 | 1 |
| RBAK | 1 |
| RBBP8 | 1 |
| RBM10 | 1 |
| RBM12B | 1 |
| RBM15B | 1 |
| RBM19 | 1 |
| RBM20 | 1 |
| RBM25 | 1 |
| RBM27 | 1 |
| RBM28 | 1 |
| RBM39 | 1 |
| RBM44 | 1 |
| RBM5 | 1 |
| RBMXL2 | 1 |
| RBMXL3 | 1 |
| RBP5 | 1 |
| RC3H1 | 1 |
| RCAN2 | 1 |
| RCN1 | 1 |
| RCOR2 | 1 |
| RCOR3 | 1 |
| RDBP | 1 |
| RDH11 | 1 |
| RECQL4 | 1 |
| RECQL5 | 1 |
| REEP3 | 1 |
| REEP5 | 1 |
| REG3G | 1 |
| RELL1 | 1 |
| REN | 1 |
| RENBP | 1 |
| REPIN1 | 1 |
| REPS1 | 1 |
| RET | 1 |
| REV1 | 1 |
| REXO4 | 1 |
| RFC1 | 1 |
| RFC2 | 1 |
| RFC3 | 1 |
| RFC4 | 1 |
| RFK | 1 |
| RFWD3 | 1 |
| RFX4 | 1 |
| RFX5 | 1 |
| RFX6 | 1 |
| RFX7 | 1 |
| RGL1 | 1 |
| RGS22 | 1 |
| RGS4 | 1 |
| RGS7BP | 1 |
| RGS8 | 1 |
| RGSL1 | 1 |
| RHAG | 1 |
| RHBDD2 | 1 |
| RHBDL2 | 1 |
| RHO | 1 |
| RHOA | 1 |
| RHOG | 1 |
| RHOU | 1 |
| RHOV | 1 |
| RHPN1 | 1 |
| RHPN2 | 1 |
| RICTOR | 1 |
| RILPL1 | 1 |
| RILPL2 | 1 |
| RIMBP2 | 1 |
| RIN1 | 1 |
| RING1 | 1 |
| RIOK1 | 1 |
| RIOK2 | 1 |
| RIPK1 | 1 |
| RIT1 | 1 |
| RIT2 | 1 |
| RLF | 1 |
| RMND5A | 1 |
| RMND5B | 1 |
| RNASE10 | 1 |
| RNASE4 | 1 |
| RNF111 | 1 |
| RNF133 | 1 |
| RNF139 | 1 |
| RNF14 | 1 |
| RNF148 | 1 |
| RNF157 | 1 |
| RNF166 | 1 |
| RNF167 | 1 |
| RNF168 | 1 |
| RNF169 | 1 |
| RNF182 | 1 |
| RNF20 | 1 |
| RNF212 | 1 |
| RNF216 | 1 |
| RNF217 | 1 |
| RNF220 | 1 |
| RNF4 | 1 |
| RNF8 | 1 |
| RNPC3 | 1 |
| ROBO1 | 1 |
| ROBO4 | 1 |
| ROCK1 | 1 |
| ROCK2 | 1 |
| ROMO1 | 1 |
| ROPN1L | 1 |
| RPA1 | 1 |
| RPA4 | 1 |
| RPAP3 | 1 |
| RPE | 1 |
| RPF1 | 1 |
| RPF2 | 1 |
| RPGRIP1L | 1 |
| RPL10L | 1 |
| RPL11 | 1 |
| RPL12 | 1 |
| RPL14P1 | 1 |
| RPL18A | 1 |
| RPL19 | 1 |
| RPL22 | 1 |
| RPL32 | 1 |
| RPL7 | 1 |
| RPL7L1 | 1 |
| RPN1 | 1 |
| RPRD1A | 1 |
| RPS11 | 1 |
| RPS13 | 1 |
| RPS6KB1 | 1 |
| RPS6KB2 | 1 |
| RPS6KC1 | 1 |
| RPS8 | 1 |
| RPSA | 1 |
| RPTN | 1 |
| RPTOR | 1 |
| RPUSD3 | 1 |
| RQCD1 | 1 |
| RRNAD1 | 1 |
| RRP7A | 1 |
| RSBN1 | 1 |
| RSG1 | 1 |
| RSPH1 | 1 |
| RTBDN | 1 |
| RTDR1 | 1 |
| RTL1 | 1 |
| RTN3 | 1 |
| RUFY4 | 1 |
| RUNDC3B | 1 |
| RWDD2A | 1 |
| RXFP2 | 1 |
| RXFP3 | 1 |
| RXRB | 1 |
| S100A1 | 1 |
| S100A3 | 1 |
| S100PBP | 1 |
| SAA4 | 1 |
| SACM1L | 1 |
| SALL1 | 1 |
| SALL2 | 1 |
| SAMD13 | 1 |
| SAMD3 | 1 |
| SAMD7 | 1 |
| SAP30BP | 1 |
| SAR1A | 1 |
| SAR1B | 1 |
| SARDH | 1 |
| SARM1 | 1 |
| SART1 | 1 |
| SASH1 | 1 |
| SASH3 | 1 |
| SBK2 | 1 |
| SBNO1 | 1 |
| SBNO2 | 1 |
| SCAF1 | 1 |
| SCAI | 1 |
| SCAMP4 | 1 |
| SCAND3 | 1 |
| SCAP | 1 |
| SCAPER | 1 |
| SCARB2 | 1 |
| SCARF1 | 1 |
| SCD | 1 |
| SCD5 | 1 |
| SCG5 | 1 |
| SCGB1C1 | 1 |
| SCGB2A2 | 1 |
| SCHIP1 | 1 |
| SCLT1 | 1 |
| SCN10A | 1 |
| SCN11A | 1 |
| SCN3B | 1 |
| SCO1 | 1 |
| SCP2 | 1 |
| SCRT2 | 1 |
| SCUBE3 | 1 |
| SCYL2 | 1 |
| SCYL3 | 1 |
| SDCBP | 1 |
| SDCCAG8 | 1 |
| SDHA | 1 |
| SDHAF2 | 1 |
| SDHD | 1 |
| SEBOX | 1 |
| SEC11C | 1 |
| SEC14L1 | 1 |
| SEC14L5 | 1 |
| SEC16A | 1 |
| SEC16B | 1 |
| SEC22A | 1 |
| SEC23B | 1 |
| SEC23IP | 1 |
| SEC24B | 1 |
| SEC31B | 1 |
| SEC61A1 | 1 |
| SEC62 | 1 |
| SEH1L | 1 |
| SEL1L | 1 |
| SEL1L2 | 1 |
| SELE | 1 |
| SELO | 1 |
| SELP | 1 |
| SEMA4C | 1 |
| SEMA4D | 1 |
| SEMA5A | 1 |
| SEMA6A | 1 |
| 11-Sep | 1 |
| 2-Sep | 1 |
| 7-Sep | 1 |
| SERINC1 | 1 |
| SERINC3 | 1 |
| SERINC5 | 1 |
| SERPINA1 | 1 |
| SERPINA12 | 1 |
| SERPINA3 | 1 |
| SERPINA4 | 1 |
| SERPINA6 | 1 |
| SERPINB1 | 1 |
| SERPINB13 | 1 |
| SERPINB4 | 1 |
| SERPINE1 | 1 |
| SERPINF1 | 1 |
| SERPING1 | 1 |
| SERPINH1 | 1 |
| SERPINI1 | 1 |
| SERPINI2 | 1 |
| SETBP1 | 1 |
| SETD1B | 1 |
| SETD8 | 1 |
| SEZ6 | 1 |
| SF1 | 1 |
| SF3B4 | 1 |
| SFI1 | 1 |
| SFMBT2 | 1 |
| SFN | 1 |
| SFTPA2 | 1 |
| SGCA | 1 |
| SGIP1 | 1 |
| SGK1 | 1 |
| SGK2 | 1 |
| SGMS1 | 1 |
| SGMS2 | 1 |
| SGOL1 | 1 |
| SGPL1 | 1 |
| SGSM3 | 1 |
| SGTB | 1 |
| SH2D2A | 1 |
| SH2D4B | 1 |
| SH3BGR | 1 |
| SH3D19 | 1 |
| SH3D21 | 1 |
| SH3GLB1 | 1 |
| SH3GLB2 | 1 |
| SH3KBP1 | 1 |
| SH3PXD2B | 1 |
| SH3TC1 | 1 |
| SH3TC2 | 1 |
| SHANK3 | 1 |
| SHB | 1 |
| SHC4 | 1 |
| SHCBP1 | 1 |
| SHF | 1 |
| SHISA7 | 1 |
| SHISA9 | 1 |
| SHMT2 | 1 |
| SHROOM2 | 1 |
| SIGIRR | 1 |
| SIGLEC10 | 1 |
| SIGLEC5 | 1 |
| SIGLEC6 | 1 |
| SIGLEC7 | 1 |
| SIK1 | 1 |
| SIK2 | 1 |
| SIK3 | 1 |
| SIL1 | 1 |
| SIM1 | 1 |
| SIPA1L2 | 1 |
| SIPA1L3 | 1 |
| SIRT1 | 1 |
| SIX2 | 1 |
| SIX4 | 1 |
| SKA1 | 1 |
| SKA2 | 1 |
| SKAP1 | 1 |
| SKIV2L | 1 |
| SKOR1 | 1 |
| SLA | 1 |
| SLAMF7 | 1 |
| SLC10A2 | 1 |
| SLC10A5 | 1 |
| SLC12A2 | 1 |
| SLC12A8 | 1 |
| SLC13A2 | 1 |
| SLC13A4 | 1 |
| SLC14A2 | 1 |
| SLC15A5 | 1 |
| SLC16A5 | 1 |
| SLC16A6 | 1 |
| SLC16A9 | 1 |
| SLC17A1 | 1 |
| SLC17A2 | 1 |
| SLC17A3 | 1 |
| SLC17A7 | 1 |
| SLC18A1 | 1 |
| SLC18A3 | 1 |
| SLC19A2 | 1 |
| SLC20A1 | 1 |
| SLC22A11 | 1 |
| SLC22A14 | 1 |
| SLC22A2 | 1 |
| SLC22A23 | 1 |
| SLC22A3 | 1 |
| SLC22A7 | 1 |
| SLC22A9 | 1 |
| SLC24A1 | 1 |
| SLC24A2 | 1 |
| SLC24A3 | 1 |
| SLC24A4 | 1 |
| SLC24A5 | 1 |
| SLC25A12 | 1 |
| SLC25A17 | 1 |
| SLC25A24 | 1 |
| SLC25A25 | 1 |
| SLC25A28 | 1 |
| SLC25A3 | 1 |
| SLC25A36 | 1 |
| SLC25A37 | 1 |
| SLC25A38 | 1 |
| SLC25A47 | 1 |
| SLC26A10 | 1 |
| SLC26A11 | 1 |
| SLC26A2 | 1 |
| SLC26A3 | 1 |
| SLC26A5 | 1 |
| SLC26A7 | 1 |
| SLC27A2 | 1 |
| SLC28A2 | 1 |
| SLC28A3 | 1 |
| SLC29A4 | 1 |
| SLC2A1 | 1 |
| SLC2A10 | 1 |
| SLC2A11 | 1 |
| SLC2A14 | 1 |
| SLC2A2 | 1 |
| SLC2A3 | 1 |
| SLC2A5 | 1 |
| SLC2A7 | 1 |
| SLC35F2 | 1 |
| SLC35F4 | 1 |
| SLC35G3 | 1 |
| SLC37A1 | 1 |
| SLC38A7 | 1 |
| SLC38A9 | 1 |
| SLC39A2 | 1 |
| SLC39A6 | 1 |
| SLC3A2 | 1 |
| SLC41A3 | 1 |
| SLC43A2 | 1 |
| SLC48A1 | 1 |
| SLC4A11 | 1 |
| SLC4A2 | 1 |
| SLC4A3 | 1 |
| SLC5A1 | 1 |
| SLC5A11 | 1 |
| SLC5A12 | 1 |
| SLC5A2 | 1 |
| SLC5A3 | 1 |
| SLC5A7 | 1 |
| SLC5A8 | 1 |
| SLC6A13 | 1 |
| SLC6A17 | 1 |
| SLC6A3 | 1 |
| SLC6A6 | 1 |
| SLC6A9 | 1 |
| SLC7A13 | 1 |
| SLC7A2 | 1 |
| SLC7A3 | 1 |
| SLC7A5 | 1 |
| SLC7A9 | 1 |
| SLC9A1 | 1 |
| SLC9A3 | 1 |
| SLC9A7 | 1 |
| SLC9A9 | 1 |
| SLC9B1 | 1 |
| SLCO1C1 | 1 |
| SLCO4A1 | 1 |
| SLCO4C1 | 1 |
| SLCO6A1 | 1 |
| SLFN11 | 1 |
| SLFN12 | 1 |
| SLFN13 | 1 |
| SLFN5 | 1 |
| SLFNL1 | 1 |
| SLIT1 | 1 |
| SLITRK1 | 1 |
| SLITRK6 | 1 |
| SLX4 | 1 |
| SMAD1 | 1 |
| SMAD2 | 1 |
| SMAD9 | 1 |
| SMAP1 | 1 |
| SMAP2 | 1 |
| SMARCB1 | 1 |
| SMARCD1 | 1 |
| SMARCD3 | 1 |
| SMC1B | 1 |
| SMC3 | 1 |
| SMC4 | 1 |
| SMC6 | 1 |
| SMCR7L | 1 |
| SMCR8 | 1 |
| SMEK1 | 1 |
| SMG1 | 1 |
| SMG5 | 1 |
| SMG6 | 1 |
| SMOC2 | 1 |
| SMS | 1 |
| SMTNL2 | 1 |
| SMU1 | 1 |
| SMUG1 | 1 |
| SMYD1 | 1 |
| SNAP47 | 1 |
| SNAPC1 | 1 |
| SNAPC4 | 1 |
| SNCAIP | 1 |
| SND1 | 1 |
| SNF8 | 1 |
| SNRNP35 | 1 |
| SNRPA1 | 1 |
| SNTA1 | 1 |
| SNX1 | 1 |
| SNX30 | 1 |
| SNX33 | 1 |
| SNX8 | 1 |
| SOAT1 | 1 |
| SOCS3 | 1 |
| SOCS5 | 1 |
| SOCS6 | 1 |
| SOD1 | 1 |
| SOHLH2 | 1 |
| SORCS1 | 1 |
| SOS1 | 1 |
| SOX10 | 1 |
| SOX11 | 1 |
| SOX13 | 1 |
| SOX3 | 1 |
| SOX6 | 1 |
| SOX9 | 1 |
| SP1 | 1 |
| SP140L | 1 |
| SPACA1 | 1 |
| SPACA7 | 1 |
| SPARCL1 | 1 |
| SPATA12 | 1 |
| SPATA13 | 1 |
| SPATA21 | 1 |
| SPATA25 | 1 |
| SPATA4 | 1 |
| SPATA5 | 1 |
| SPATA6 | 1 |
| SPATA8 | 1 |
| SPATS1 | 1 |
| SPDYC | 1 |
| SPG11 | 1 |
| SPG7 | 1 |
| SPIC | 1 |
| SPIRE1 | 1 |
| SPNS1 | 1 |
| SPO11 | 1 |
| SPOCK3 | 1 |
| SPOP | 1 |
| SPOPL | 1 |
| SPRED1 | 1 |
| SPTBN2 | 1 |
| SPTBN4 | 1 |
| SPTLC3 | 1 |
| SQLE | 1 |
| SRCIN1 | 1 |
| SRD5A3 | 1 |
| SREK1 | 1 |
| SRFBP1 | 1 |
| SRPK2 | 1 |
| SRRM3 | 1 |
| SRRM5 | 1 |
| SRRT | 1 |
| SRSF3 | 1 |
| SRSF4 | 1 |
| SRSF5 | 1 |
| SRSF6 | 1 |
| SSBP4 | 1 |
| SSR2 | 1 |
| SSTR4 | 1 |
| SSX6 | 1 |
| ST3GAL3 | 1 |
| ST6GAL1 | 1 |
| ST6GAL2 | 1 |
| ST6GALNAC2 | 1 |
| ST8SIA2 | 1 |
| STAB2 | 1 |
| STAC | 1 |
| STAC2 | 1 |
| STAC3 | 1 |
| STAG2 | 1 |
| STAG3 | 1 |
| STAM | 1 |
| STAMBP | 1 |
| STAMBPL1 | 1 |
| STARD13 | 1 |
| STARD7 | 1 |
| STARD8 | 1 |
| STAT1 | 1 |
| STAT2 | 1 |
| STAT5B | 1 |
| STAT6 | 1 |
| STAU1 | 1 |
| STEAP4 | 1 |
| STIM1 | 1 |
| STIM2 | 1 |
| STK17B | 1 |
| STK25 | 1 |
| STK3 | 1 |
| STK32A | 1 |
| STK36 | 1 |
| STK4 | 1 |
| STMN3 | 1 |
| STOML2 | 1 |
| STOML3 | 1 |
| STON2 | 1 |
| STOX1 | 1 |
| STRA8 | 1 |
| STRN | 1 |
| STRN3 | 1 |
| STX11 | 1 |
| STX7 | 1 |
| STX8 | 1 |
| STXBP1 | 1 |
| STXBP4 | 1 |
| STYK1 | 1 |
| SULF2 | 1 |
| SULT1C4 | 1 |
| SULT6B1 | 1 |
| SUN3 | 1 |
| SUOX | 1 |
| SUPT16H | 1 |
| SURF1 | 1 |
| SUSD2 | 1 |
| SVEP1 | 1 |
| SVOPL | 1 |
| SYAP1 | 1 |
| SYBU | 1 |
| SYCE2 | 1 |
| SYCP1 | 1 |
| SYMPK | 1 |
| SYN2 | 1 |
| SYNC | 1 |
| SYNGAP1 | 1 |
| SYNGR1 | 1 |
| SYNGR2 | 1 |
| SYNPO2 | 1 |
| SYNPO2L | 1 |
| SYNRG | 1 |
| SYT1 | 1 |
| SYT11 | 1 |
| SYT2 | 1 |
| SYT6 | 1 |
| SYTL4 | 1 |
| TAAR1 | 1 |
| TAAR6 | 1 |
| TAB1 | 1 |
| TAB2 | 1 |
| TAC1 | 1 |
| TACR3 | 1 |
| TADA2A | 1 |
| TAF1 | 1 |
| TAF11 | 1 |
| TAF15 | 1 |
| TAF1A | 1 |
| TAF3 | 1 |
| TAF6 | 1 |
| TAF8 | 1 |
| TAGLN3 | 1 |
| TAMM41 | 1 |
| TANC1 | 1 |
| TAOK1 | 1 |
| TAOK3 | 1 |
| TARBP1 | 1 |
| TARDBP | 1 |
| TARM1 | 1 |
| TARS | 1 |
| TAS2R19 | 1 |
| TAS2R3 | 1 |
| TAS2R43 | 1 |
| TAS2R46 | 1 |
| TATDN3 | 1 |
| TAX1BP1 | 1 |
| TAX1BP3 | 1 |
| TBC1D1 | 1 |
| TBC1D10C | 1 |
| TBC1D13 | 1 |
| TBC1D3F | 1 |
| TBC1D4 | 1 |
| TBC1D8 | 1 |
| TBC1D9B | 1 |
| TBCB | 1 |
| TBL2 | 1 |
| TBP | 1 |
| TBPL1 | 1 |
| TBX15 | 1 |
| TBX18 | 1 |
| TBX19 | 1 |
| TBX21 | 1 |
| TBX3 | 1 |
| TBX4 | 1 |
| TBX5 | 1 |
| TC2N | 1 |
| TCEA3 | 1 |
| TCEAL7 | 1 |
| TCEAL8 | 1 |
| TCEANC | 1 |
| TCEB3B | 1 |
| TCERG1L | 1 |
| TCF20 | 1 |
| TCF25 | 1 |
| TCF4 | 1 |
| TCF7L2 | 1 |
| TCHP | 1 |
| TCN1 | 1 |
| TCP10L | 1 |
| TCTE1 | 1 |
| TCTEX1D2 | 1 |
| TDP2 | 1 |
| TDRD1 | 1 |
| TDRD3 | 1 |
| TDRD9 | 1 |
| TDRKH | 1 |
| TEAD4 | 1 |
| TECPR1 | 1 |
| TECPR2 | 1 |
| TECRL | 1 |
| TECTB | 1 |
| TEDDM1 | 1 |
| TEK | 1 |
| TEKT3 | 1 |
| TEKT4 | 1 |
| TEP1 | 1 |
| TEPP | 1 |
| TES | 1 |
| TESK2 | 1 |
| TET2 | 1 |
| TET3 | 1 |
| TEX11 | 1 |
| TEX14 | 1 |
| TEX261 | 1 |
| TFAM | 1 |
| TFAP2A | 1 |
| TFAP2C | 1 |
| TFB1M | 1 |
| TFB2M | 1 |
| TFE3 | 1 |
| TFEC | 1 |
| TFF1 | 1 |
| TFG | 1 |
| TFPI2 | 1 |
| TGFBR3 | 1 |
| TGM4 | 1 |
| TGM5 | 1 |
| TGM6 | 1 |
| TGM7 | 1 |
| TGS1 | 1 |
| TH1L | 1 |
| THAP1 | 1 |
| THAP5 | 1 |
| THBS1 | 1 |
| THNSL2 | 1 |
| THOC2 | 1 |
| THRAP3 | 1 |
| THSD1 | 1 |
| THSD4 | 1 |
| THUMPD2 | 1 |
| TIA1 | 1 |
| TIAM1 | 1 |
| TIGD4 | 1 |
| TIMM44 | 1 |
| TIMM50 | 1 |
| TINAG | 1 |
| TINAGL1 | 1 |
| TIPARP | 1 |
| TIRAP | 1 |
| TJP2 | 1 |
| TK1 | 1 |
| TKTL1 | 1 |
| TLK2 | 1 |
| TLL2 | 1 |
| TLR1 | 1 |
| TLR10 | 1 |
| TLR3 | 1 |
| TLR5 | 1 |
| TLR9 | 1 |
| TLX1 | 1 |
| TM4SF18 | 1 |
| TM6SF2 | 1 |
| TM9SF2 | 1 |
| TM9SF4 | 1 |
| TMC1 | 1 |
| TMCC1 | 1 |
| TMCO3 | 1 |
| TMED7 | 1 |
| TMEFF1 | 1 |
| TMEM117 | 1 |
| TMEM125 | 1 |
| TMEM129 | 1 |
| TMEM134 | 1 |
| TMEM135 | 1 |
| TMEM150A | 1 |
| TMEM150B | 1 |
| TMEM154 | 1 |
| TMEM175 | 1 |
| TMEM180 | 1 |
| TMEM19 | 1 |
| TMEM194A | 1 |
| TMEM194B | 1 |
| TMEM196 | 1 |
| TMEM208 | 1 |
| TMEM214 | 1 |
| TMEM225 | 1 |
| TMEM229B | 1 |
| TMEM237 | 1 |
| TMEM26 | 1 |
| TMEM35 | 1 |
| TMEM44 | 1 |
| TMEM45B | 1 |
| TMEM48 | 1 |
| TMEM53 | 1 |
| TMEM57 | 1 |
| TMEM59 | 1 |
| TMEM59L | 1 |
| TMEM60 | 1 |
| TMEM62 | 1 |
| TMEM63A | 1 |
| TMEM66 | 1 |
| TMEM69 | 1 |
| TMEM71 | 1 |
| TMEM74 | 1 |
| TMEM79 | 1 |
| TMEM86A | 1 |
| TMEM87B | 1 |
| TMEM9 | 1 |
| TMEM92 | 1 |
| TMF1 | 1 |
| TMIGD1 | 1 |
| TMPRSS11A | 1 |
| TMPRSS11D | 1 |
| TMPRSS4 | 1 |
| TMPRSS9 | 1 |
| TMTC1 | 1 |
| TMX2 | 1 |
| TMX4 | 1 |
| TNFAIP3 | 1 |
| TNFAIP6 | 1 |
| TNFRSF10D | 1 |
| TNFRSF11A | 1 |
| TNFRSF13B | 1 |
| TNFRSF17 | 1 |
| TNFRSF8 | 1 |
| TNFSF11 | 1 |
| TNFSF12 | 1 |
| TNFSF12-TNFSF13 | 1 |
| TNFSF13 | 1 |
| TNFSF14 | 1 |
| TNFSF15 | 1 |
| TNFSF4 | 1 |
| TNIK | 1 |
| TNIP1 | 1 |
| TNMD | 1 |
| TNNT2 | 1 |
| TNNT3 | 1 |
| TNXB | 1 |
| TOB1 | 1 |
| TOB2 | 1 |
| TOE1 | 1 |
| TOLLIP | 1 |
| TONSL | 1 |
| TOP1MT | 1 |
| TOP2A | 1 |
| TOP3A | 1 |
| TOP3B | 1 |
| TOR1AIP1 | 1 |
| TOR3A | 1 |
| TOX | 1 |
| TP53BP2 | 1 |
| TP53I13 | 1 |
| TP53I3 | 1 |
| TPH1 | 1 |
| TPM4 | 1 |
| TPP1 | 1 |
| TPP2 | 1 |
| TPSG1 | 1 |
| TRA2A | 1 |
| TRAC | 1 |
| TRAF3 | 1 |
| TRAF3IP2 | 1 |
| TRAIP | 1 |
| TRAK2 | 1 |
| TRAPPC10 | 1 |
| TRAPPC12 | 1 |
| TRAPPC8 | 1 |
| TRBC2 | 1 |
| TRBV23-1 | 1 |
| TRBV28 | 1 |
| TRBV29-1 | 1 |
| TRBV5-6 | 1 |
| TRBV6-9 | 1 |
| TRBV7-6 | 1 |
| TRDMT1 | 1 |
| TREML2 | 1 |
| TRERF1 | 1 |
| TRGV3 | 1 |
| TRH | 1 |
| TRIM21 | 1 |
| TRIM22 | 1 |
| TRIM29 | 1 |
| TRIM32 | 1 |
| TRIM38 | 1 |
| TRIM42 | 1 |
| TRIM45 | 1 |
| TRIM46 | 1 |
| TRIM47 | 1 |
| TRIM56 | 1 |
| TRIM58 | 1 |
| TRIM60 | 1 |
| TRIM61 | 1 |
| TRIM66 | 1 |
| TRIM71 | 1 |
| TRIML1 | 1 |
| TRIOBP | 1 |
| TRIP10 | 1 |
| TRIP12 | 1 |
| TRIP4 | 1 |
| TRMT61B | 1 |
| TRPA1 | 1 |
| TRPC1 | 1 |
| TRPC3 | 1 |
| TRPC6 | 1 |
| TRPC7 | 1 |
| TRPM1 | 1 |
| TRPM5 | 1 |
| TRPM7 | 1 |
| TRPV4 | 1 |
| TSC22D4 | 1 |
| TSG101 | 1 |
| TSHR | 1 |
| TSHZ1 | 1 |
| TSHZ2 | 1 |
| TSN | 1 |
| TSNARE1 | 1 |
| TSPAN1 | 1 |
| TSPAN10 | 1 |
| TSPAN11 | 1 |
| TSPAN18 | 1 |
| TSPAN7 | 1 |
| TSPEAR | 1 |
| TSPY1 | 1 |
| TSPY2 | 1 |
| TSR2 | 1 |
| TSSC1 | 1 |
| TSSK4 | 1 |
| TSTD2 | 1 |
| TTBK2 | 1 |
| TTC1 | 1 |
| TTC12 | 1 |
| TTC17 | 1 |
| TTC22 | 1 |
| TTC24 | 1 |
| TTC27 | 1 |
| TTC30B | 1 |
| TTC39C | 1 |
| TTC5 | 1 |
| TTC7A | 1 |
| TTI1 | 1 |
| TTI2 | 1 |
| TTLL11 | 1 |
| TTLL3 | 1 |
| TTLL5 | 1 |
| TTLL6 | 1 |
| TTLL9 | 1 |
| TTYH1 | 1 |
| TUB | 1 |
| TUBA3C | 1 |
| TUBA3E | 1 |
| TUBAL3 | 1 |
| TUBB2A | 1 |
| TUBB7P | 1 |
| TUBGCP2 | 1 |
| TUBGCP4 | 1 |
| TUFM | 1 |
| TULP2 | 1 |
| TULP3 | 1 |
| TUSC3 | 1 |
| TWIST1 | 1 |
| TXK | 1 |
| TXLNG | 1 |
| TXN | 1 |
| TXNDC11 | 1 |
| TXNRD1 | 1 |
| TYR | 1 |
| U2AF1L4 | 1 |
| U2SURP | 1 |
| UBA5 | 1 |
| UBA6 | 1 |
| UBA7 | 1 |
| UBAC2 | 1 |
| UBAP2L | 1 |
| UBASH3B | 1 |
| UBB | 1 |
| UBE2B | 1 |
| UBE2C | 1 |
| UBE2E1 | 1 |
| UBE2E3 | 1 |
| UBE2F | 1 |
| UBE2G1 | 1 |
| UBE2N | 1 |
| UBE3B | 1 |
| UBE4A | 1 |
| UBIAD1 | 1 |
| UBL3 | 1 |
| UBN2 | 1 |
| UBQLN2 | 1 |
| UBQLN3 | 1 |
| UBR2 | 1 |
| UBXN11 | 1 |
| UBXN2A | 1 |
| UBXN6 | 1 |
| UBXN7 | 1 |
| UCHL1 | 1 |
| UCN2 | 1 |
| UGCG | 1 |
| UGT1A1 | 1 |
| UGT1A6 | 1 |
| UGT2B10 | 1 |
| UHMK1 | 1 |
| UHRF1BP1 | 1 |
| UHRF2 | 1 |
| ULBP1 | 1 |
| ULBP3 | 1 |
| ULK2 | 1 |
| ULK4 | 1 |
| UMOD | 1 |
| UMODL1 | 1 |
| UMPS | 1 |
| UNC50 | 1 |
| UNC5D | 1 |
| UNC79 | 1 |
| UNG | 1 |
| UNK | 1 |
| UNKL | 1 |
| UPF1 | 1 |
| UPF2 | 1 |
| UPK1B | 1 |
| UPK2 | 1 |
| UPP2 | 1 |
| UPRT | 1 |
| UQCRQ | 1 |
| URB1 | 1 |
| URB2 | 1 |
| URGCP | 1 |
| URI1 | 1 |
| UROC1 | 1 |
| USF2 | 1 |
| USHBP1 | 1 |
| USO1 | 1 |
| USP1 | 1 |
| USP12 | 1 |
| USP17L2 | 1 |
| USP20 | 1 |
| USP22 | 1 |
| USP24 | 1 |
| USP28 | 1 |
| USP30 | 1 |
| USP36 | 1 |
| USP37 | 1 |
| USP4 | 1 |
| USP5 | 1 |
| USP50 | 1 |
| USPL1 | 1 |
| UTP11L | 1 |
| UTP14A | 1 |
| UTP23 | 1 |
| UTP6 | 1 |
| UVRAG | 1 |
| UXS1 | 1 |
| VAC14 | 1 |
| VARS2 | 1 |
| VAV1 | 1 |
| VAX2 | 1 |
| VBP1 | 1 |
| VEGFB | 1 |
| VEZF1 | 1 |
| VEZT | 1 |
| VGF | 1 |
| VILL | 1 |
| VIT | 1 |
| VLDLR | 1 |
| VMP1 | 1 |
| VN1R2 | 1 |
| VN1R4 | 1 |
| VNN2 | 1 |
| VPREB1 | 1 |
| VPS11 | 1 |
| VPS16 | 1 |
| VPS26A | 1 |
| VPS29 | 1 |
| VPS35 | 1 |
| VPS45 | 1 |
| VPS4B | 1 |
| VPS52 | 1 |
| VPS53 | 1 |
| VSIG10 | 1 |
| VSTM1 | 1 |
| VSTM2A | 1 |
| VSTM4 | 1 |
| VSX2 | 1 |
| VTCN1 | 1 |
| VWC2 | 1 |
| VWC2L | 1 |
| VWCE | 1 |
| WAC | 1 |
| WBSCR17 | 1 |
| WDR1 | 1 |
| WDR19 | 1 |
| WDR43 | 1 |
| WDR53 | 1 |
| WDR6 | 1 |
| WDR61 | 1 |
| WDR65 | 1 |
| WDR69 | 1 |
| WDR74 | 1 |
| WDR78 | 1 |
| WDR81 | 1 |
| WDR89 | 1 |
| WDR90 | 1 |
| WDTC1 | 1 |
| WFDC11 | 1 |
| WFDC6 | 1 |
| WFDC9 | 1 |
| WFIKKN1 | 1 |
| WFS1 | 1 |
| WIPF2 | 1 |
| WISP1 | 1 |
| WISP3 | 1 |
| WNK2 | 1 |
| WNK3 | 1 |
| WNT10A | 1 |
| WNT2 | 1 |
| WNT5A | 1 |
| WRN | 1 |
| WT1 | 1 |
| WWC1 | 1 |
| WWP2 | 1 |
| XDH | 1 |
| XKR7 | 1 |
| XKR9 | 1 |
| XPO4 | 1 |
| XPR1 | 1 |
| XRCC2 | 1 |
| XRCC4 | 1 |
| XYLT2 | 1 |
| YARS | 1 |
| YDJC | 1 |
| YIF1B | 1 |
| YIPF4 | 1 |
| YME1L1 | 1 |
| YTHDC1 | 1 |
| YTHDF2 | 1 |
| YWHAB | 1 |
| YY1 | 1 |
| YY1AP1 | 1 |
| YY2 | 1 |
| ZADH2 | 1 |
| ZAR1 | 1 |
| ZBED4 | 1 |
| ZBED6 | 1 |
| ZBP1 | 1 |
| ZBTB3 | 1 |
| ZBTB39 | 1 |
| ZBTB4 | 1 |
| ZBTB40 | 1 |
| ZBTB41 | 1 |
| ZBTB48 | 1 |
| ZBTB7A | 1 |
| ZBTB7B | 1 |
| ZBTB8A | 1 |
| ZC3H10 | 1 |
| ZC3H11A | 1 |
| ZC3H14 | 1 |
| ZC3H18 | 1 |
| ZC3H4 | 1 |
| ZCCHC11 | 1 |
| ZCCHC16 | 1 |
| ZCCHC18 | 1 |
| ZCCHC24 | 1 |
| ZCCHC5 | 1 |
| ZCCHC6 | 1 |
| ZDBF2 | 1 |
| ZDHHC11 | 1 |
| ZDHHC13 | 1 |
| ZDHHC14 | 1 |
| ZDHHC15 | 1 |
| ZDHHC17 | 1 |
| ZDHHC18 | 1 |
| ZDHHC19 | 1 |
| ZDHHC22 | 1 |
| ZDHHC24 | 1 |
| ZDHHC4 | 1 |
| ZDHHC8 | 1 |
| ZER1 | 1 |
| ZFAND1 | 1 |
| ZFAND2B | 1 |
| ZFAT | 1 |
| ZFP14 | 1 |
| ZFP2 | 1 |
| ZFP28 | 1 |
| ZFP30 | 1 |
| ZFP57 | 1 |
| ZFP82 | 1 |
| ZFR2 | 1 |
| ZFX | 1 |
| ZFYVE20 | 1 |
| ZHX1 | 1 |
| ZKSCAN2 | 1 |
| ZKSCAN5 | 1 |
| ZMAT2 | 1 |
| ZMAT4 | 1 |
| ZMIZ2 | 1 |
| ZMYM1 | 1 |
| ZMYM3 | 1 |
| ZMYND10 | 1 |
| ZMYND11 | 1 |
| ZMYND15 | 1 |
| ZMYND8 | 1 |
| ZNF117 | 1 |
| ZNF141 | 1 |
| ZNF142 | 1 |
| ZNF155 | 1 |
| ZNF167 | 1 |
| ZNF175 | 1 |
| ZNF18 | 1 |
| ZNF180 | 1 |
| ZNF181 | 1 |
| ZNF184 | 1 |
| ZNF192 | 1 |
| ZNF193 | 1 |
| ZNF195 | 1 |
| ZNF197 | 1 |
| ZNF20 | 1 |
| ZNF202 | 1 |
| ZNF207 | 1 |
| ZNF211 | 1 |
| ZNF212 | 1 |
| ZNF217 | 1 |
| ZNF219 | 1 |
| ZNF222 | 1 |
| ZNF223 | 1 |
| ZNF224 | 1 |
| ZNF229 | 1 |
| ZNF230 | 1 |
| ZNF233 | 1 |
| ZNF248 | 1 |
| ZNF259 | 1 |
| ZNF260 | 1 |
| ZNF273 | 1 |
| ZNF28 | 1 |
| ZNF280A | 1 |
| ZNF281 | 1 |
| ZNF284 | 1 |
| ZNF285 | 1 |
| ZNF286B | 1 |
| ZNF287 | 1 |
| ZNF292 | 1 |
| ZNF295 | 1 |
| ZNF3 | 1 |
| ZNF302 | 1 |
| ZNF304 | 1 |
| ZNF311 | 1 |
| ZNF319 | 1 |
| ZNF32 | 1 |
| ZNF323 | 1 |
| ZNF324 | 1 |
| ZNF330 | 1 |
| ZNF331 | 1 |
| ZNF335 | 1 |
| ZNF33B | 1 |
| ZNF343 | 1 |
| ZNF354B | 1 |
| ZNF354C | 1 |
| ZNF366 | 1 |
| ZNF37A | 1 |
| ZNF382 | 1 |
| ZNF385B | 1 |
| ZNF394 | 1 |
| ZNF396 | 1 |
| ZNF397 | 1 |
| ZNF407 | 1 |
| ZNF41 | 1 |
| ZNF415 | 1 |
| ZNF420 | 1 |
| ZNF423 | 1 |
| ZNF426 | 1 |
| ZNF43 | 1 |
| ZNF430 | 1 |
| ZNF433 | 1 |
| ZNF434 | 1 |
| ZNF436 | 1 |
| ZNF439 | 1 |
| ZNF440 | 1 |
| ZNF442 | 1 |
| ZNF445 | 1 |
| ZNF449 | 1 |
| ZNF451 | 1 |
| ZNF461 | 1 |
| ZNF467 | 1 |
| ZNF468 | 1 |
| ZNF471 | 1 |
| ZNF474 | 1 |
| ZNF480 | 1 |
| ZNF493 | 1 |
| ZNF498 | 1 |
| ZNF502 | 1 |
| ZNF506 | 1 |
| ZNF510 | 1 |
| ZNF516 | 1 |
| ZNF518A | 1 |
| ZNF526 | 1 |
| ZNF527 | 1 |
| ZNF530 | 1 |
| ZNF534 | 1 |
| ZNF541 | 1 |
| ZNF543 | 1 |
| ZNF546 | 1 |
| ZNF549 | 1 |
| ZNF550 | 1 |
| ZNF558 | 1 |
| ZNF561 | 1 |
| ZNF563 | 1 |
| ZNF564 | 1 |
| ZNF567 | 1 |
| ZNF568 | 1 |
| ZNF571 | 1 |
| ZNF572 | 1 |
| ZNF574 | 1 |
| ZNF577 | 1 |
| ZNF586 | 1 |
| ZNF587 | 1 |
| ZNF589 | 1 |
| ZNF599 | 1 |
| ZNF608 | 1 |
| ZNF611 | 1 |
| ZNF614 | 1 |
| ZNF616 | 1 |
| ZNF628 | 1 |
| ZNF630 | 1 |
| ZNF641 | 1 |
| ZNF644 | 1 |
| ZNF648 | 1 |
| ZNF655 | 1 |
| ZNF667 | 1 |
| ZNF670 | 1 |
| ZNF671 | 1 |
| ZNF675 | 1 |
| ZNF679 | 1 |
| ZNF681 | 1 |
| ZNF687 | 1 |
| ZNF692 | 1 |
| ZNF695 | 1 |
| ZNF7 | 1 |
| ZNF708 | 1 |
| ZNF713 | 1 |
| ZNF716 | 1 |
| ZNF721 | 1 |
| ZNF727 | 1 |
| ZNF735 | 1 |
| ZNF740 | 1 |
| ZNF746 | 1 |
| ZNF749 | 1 |
| ZNF75D | 1 |
| ZNF761 | 1 |
| ZNF773 | 1 |
| ZNF775 | 1 |
| ZNF780B | 1 |
| ZNF787 | 1 |
| ZNF790 | 1 |
| ZNF792 | 1 |
| ZNF793 | 1 |
| ZNF813 | 1 |
| ZNF814 | 1 |
| ZNF84 | 1 |
| ZNF841 | 1 |
| ZNF85 | 1 |
| ZNF860 | 1 |
| ZNF880 | 1 |
| ZNF883 | 1 |
| ZNF90 | 1 |
| ZNFX1 | 1 |
| ZNHIT1 | 1 |
| ZNHIT6 | 1 |
| ZRANB1 | 1 |
| ZRANB3 | 1 |
| ZRSR1 | 1 |
| ZRSR2 | 1 |
| ZSCAN12 | 1 |
| ZSCAN16 | 1 |
| ZSCAN2 | 1 |
| ZSCAN4 | 1 |
| ZSCAN5A | 1 |
| ZSWIM5 | 1 |
| ZWILCH | 1 |
| ZYG11A | 1 |
| ZZEF1 | 1 |
| ZZZ3 | 1 |
