# Supplementary material for: Integrated Analysis of Mutation Data from Various Sources Identifies Key Genes and Signaling Pathways in Hepatocellular Carcinoma
Source: PLoS One. 2014 Jul 2;9(7):e100854. doi: 10.1371/journal.pone.0100854 (PMC4079600; doi:10.1371/journal.pone.0100854)
Supplement: Table S3 — 113 significantly mutated pathways. (DOC) [file pone.0100854.s003.doc]

Supplementary Table S3. 113 significantly mutated pathways

| Pathway name | Coverage | P value | FDR value |
| --- | --- | --- | --- |
| MAPK signaling pathway | 0.64 | 0 | 0 |
| Calcium signaling pathway | 0.54 | 0 | 0 |
| Cell cycle | 0.50 | 0 | 0 |
| p53 signaling pathway | 0.41 | 0 | 0 |
| PI3K-Akt signaling pathway | 0.74 | 0 | 0 |
| Apoptosis | 0.43 | 0 | 0 |
| Wnt signaling pathway | 0.58 | 0 | 0 |
| Focal adhesion | 0.70 | 0 | 0 |
| ECM-receptor interaction | 0.48 | 0 | 0 |
| Adherens junction | 0.43 | 0 | 0 |
| Tight junction | 0.52 | 0 | 0 |
| Leukocyte transendothelial migration | 0.44 | 0 | 0 |
| Neurotrophin signaling pathway | 0.45 | 0 | 0 |
| Retrograde endocannabinoid signaling | 0.43 | 0 | 0 |
| Glutamatergic synapse | 0.47 | 0 | 0 |
| Insulin secretion | 0.39 | 0 | 0 |
| Circadian entrainment | 0.41 | 0.0001 | 0.0011 |
| Olfactory transduction | 0.61 | 0.0001 | 0.0011 |
| Melanogenesis | 0.39 | 0.0001 | 0.0011 |
| Metabolic pathways | 0.82 | 0.0003 | 0.0026 |
| Neuroactive ligand-receptor interaction | 0.53 | 0.0003 | 0.0026 |
| Cholinergic synapse | 0.39 | 0.0003 | 0.0026 |
| Protein digestion and absorption | 0.36 | 0.0003 | 0.0026 |
| Dopaminergic synapse | 0.41 | 0.0004 | 0.0033 |
| Axon guidance | 0.40 | 0.0006 | 0.0048 |
| Phosphatidylinositol signaling system | 0.35 | 0.0007 | 0.0048 |
| Hippo signaling pathway | 0.42 | 0.0007 | 0.0048 |
| Long-term depression | 0.32 | 0.0007 | 0.0048 |
| Regulation of actin cytoskeleton | 0.47 | 0.0007 | 0.0048 |
| Vascular smooth muscle contraction | 0.38 | 0.0008 | 0.0052 |
| Serotonergic synapse | 0.36 | 0.0008 | 0.0052 |
| Vitamin digestion and absorption | 0.20 | 0.0009 | 0.0056 |
| Endocytosis | 0.45 | 0.0011 | 0.0061 |
| GnRH signaling pathway | 0.34 | 0.0011 | 0.0061 |
| Salivary secretion | 0.34 | 0.0011 | 0.0061 |
| Pancreatic secretion | 0.35 | 0.0011 | 0.0061 |
| Inositol phosphate metabolism | 0.29 | 0.0013 | 0.0067 |
| ABC transporters | 0.28 | 0.0013 | 0.0067 |
| Progesterone-mediated oocyte maturation | 0.31 | 0.0013 | 0.0067 |
| Cell adhesion molecules (CAMs) | 0.38 | 0.0016 | 0.0077 |
| Gap junction | 0.33 | 0.0017 | 0.0077 |
| GABAergic synapse | 0.31 | 0.0017 | 0.0077 |
| Gastric acid secretion | 0.29 | 0.0017 | 0.0077 |
| Bile secretion | 0.28 | 0.0017 | 0.0077 |
| Long-term potentiation | 0.29 | 0.0018 | 0.0080 |
| Chemokine signaling pathway | 0.43 | 0.0019 | 0.0083 |
| Insulin signaling pathway | 0.37 | 0.0021 | 0.0089 |
| Ubiquitin mediated proteolysis | 0.36 | 0.0029 | 0.0121 |
| Homologous recombination | 0.13 | 0.0032 | 0.0131 |
| TGF-beta signaling pathway | 0.25 | 0.0035 | 0.0136 |
| Tyrosine metabolism | 0.14 | 0.0036 | 0.0136 |
| ErbB signaling pathway | 0.26 | 0.0037 | 0.0136 |
| Taste transduction | 0.19 | 0.0037 | 0.0136 |
| Vasopressin-regulated water reabsorption | 0.16 | 0.0037 | 0.0136 |
| HIF-1 signaling pathway | 0.30 | 0.0038 | 0.0136 |
| Lysine degradation | 0.20 | 0.0039 | 0.0136 |
| Cardiac muscle contraction | 0.25 | 0.0039 | 0.0136 |
| T cell receptor signaling pathway | 0.28 | 0.0040 | 0.0136 |
| Endocrine and other factor-regulated calcium reabsorption | 0.19 | 0.0040 | 0.0136 |
| Phagosome | 0.35 | 0.0043 | 0.0139 |
| VEGF signaling pathway | 0.20 | 0.0043 | 0.0139 |
| Complement and coagulation cascades | 0.24 | 0.0043 | 0.0139 |
| Fc gamma R-mediated phagocytosis | 0.26 | 0.0045 | 0.0142 |
| Oocyte meiosis | 0.29 | 0.0046 | 0.0142 |
| Fanconi anemia pathway | 0.21 | 0.0047 | 0.0142 |
| Jak-STAT signaling pathway | 0.36 | 0.0047 | 0.0142 |
| Ovarian steroidogenesis | 0.17 | 0.0049 | 0.0146 |
| Carbohydrate digestion and absorption | 0.15 | 0.0050 | 0.0146 |
| Purine metabolism | 0.36 | 0.0052 | 0.0146 |
| Dorso-ventral axis formation | 0.11 | 0.0052 | 0.0146 |
| B cell receptor signaling pathway | 0.23 | 0.0052 | 0.0146 |
| NOD-like receptor signaling pathway | 0.17 | 0.0053 | 0.0147 |
| PPAR signaling pathway | 0.19 | 0.0054 | 0.0148 |
| mTOR signaling pathway | 0.19 | 0.0055 | 0.0149 |
| Adipocytokine signaling pathway | 0.19 | 0.0062 | 0.0165 |
| NF-kappa B signaling pathway | 0.24 | 0.0063 | 0.0166 |
| Cytokine-cytokine receptor interaction | 0.43 | 0.0066 | 0.0171 |
| Glycerolipid metabolism | 0.16 | 0.0067 | 0.0172 |
| Spliceosome | 0.29 | 0.0069 | 0.0175 |
| Hedgehog signaling pathway | 0.15 | 0.0070 | 0.0175 |
| Fc epsilon RI signaling pathway | 0.19 | 0.0073 | 0.0180 |
| Fructose and mannose metabolism | 0.12 | 0.0074 | 0.0180 |
| Osteoclast differentiation | 0.29 | 0.0077 | 0.0186 |
| Fat digestion and absorption | 0.14 | 0.0078 | 0.0186 |
| RNA degradation | 0.18 | 0.0083 | 0.0195 |
| Natural killer cell mediated cytotoxicity | 0.29 | 0.0084 | 0.0195 |
| Aldosterone-regulated sodium reabsorption | 0.13 | 0.0085 | 0.0195 |
| Galactose metabolism | 0.10 | 0.0089 | 0.0202 |
| mRNA surveillance pathway | 0.20 | 0.0108 | 0.0242 |
| Mineral absorption | 0.14 | 0.0109 | 0.0242 |
| Non-homologous end-joining | 0.06 | 0.0118 | 0.0259 |
| rginine and proline metabolism | 0.15 | 0.0126 | 0.0273 |
| Fatty acid metabolism | 0.13 | 0.0127 | 0.0273 |
| Hematopoietic cell lineage | 0.19 | 0.0129 | 0.0274 |
| Protein processing in endoplasmic reticulum | 0.30 | 0.0130 | 0.0274 |
| RNA transport | 0.29 | 0.0137 | 0.0285 |
| Steroid biosynthesis | 0.07 | 0.0143 | 0.0295 |
| Toll-like receptor signaling pathway | 0.22 | 0.0152 | 0.0310 |
| Tryptophan metabolism | 0.11 | 0.0163 | 0.0325 |
| Drug metabolism - cytochrome P450 | 0.16 | 0.0163 | 0.0325 |
| Synaptic vesicle cycle | 0.15 | 0.0165 | 0.0325 |
| Peroxisome | 0.18 | 0.0166 | 0.0325 |
| Circadian rhythm | 0.10 | 0.0169 | 0.0328 |
| Glycolysis / Gluconeogenesis | 0.15 | 0.0174 | 0.0335 |
| Other types of O-glycan biosynthesis | 0.09 | 0.0179 | 0.0341 |
| Ribosome biogenesis in eukaryotes | 0.17 | 0.0211 | 0.0394 |
| DNA replication | 0.10 | 0.0211 | 0.0394 |
| Butanoate metabolism | 0.09 | 0.0218 | 0.0404 |
| Notch signaling pathway | 0.12 | 0.0228 | 0.0418 |
| Valine, leucine and isoleucine degradation | 0.11 | 0.0239 | 0.0435 |
| Mucin type O-Glycan biosynthesis | 0.09 | 0.0244 | 0.0440 |
| Amino sugar and nucleotide sugar metabolism | 0.12 | 0.0277 | 0.0495 |
| Biosynthesis of unsaturated fatty acids | 0.07 | 0.0282 | 0.0499 |
