# Supplementary material for: Integrated Analysis of Mutation Data from Various Sources Identifies Key Genes and Signaling Pathways in Hepatocellular Carcinoma
Source: PLoS One. 2014 Jul 2;9(7):e100854. doi: 10.1371/journal.pone.0100854 (PMC4079600; doi:10.1371/journal.pone.0100854)
Supplement: Table S4 — Mutated genes implicated in 113 significantly mutated pathways. (DOC) [file pone.0100854.s004.doc]

Supplementary Table S4. Mutated genes implicated in 113 significantly mutated pathways

| Gene Symbol | Pathway name |
| --- | --- |
| ARRB2 | MAPK signaling pathway |
| ATF4 | MAPK signaling pathway |
| BRAF | MAPK signaling pathway |
| CACNA1A | MAPK signaling pathway |
| CACNA1B | MAPK signaling pathway |
| CACNA1C | MAPK signaling pathway |
| CACNA1D | MAPK signaling pathway |
| CACNA1E | MAPK signaling pathway |
| CACNA1S | MAPK signaling pathway |
| CACNA2D1 | MAPK signaling pathway |
| CACNB1 | MAPK signaling pathway |
| CACNB2 | MAPK signaling pathway |
| CACNB4 | MAPK signaling pathway |
| CHUK | MAPK signaling pathway |
| MAP3K8 | MAPK signaling pathway |
| ATF2 | MAPK signaling pathway |
| DUSP1 | MAPK signaling pathway |
| DUSP4 | MAPK signaling pathway |
| EGFR | MAPK signaling pathway |
| ELK1 | MAPK signaling pathway |
| MECOM | MAPK signaling pathway |
| FGF5 | MAPK signaling pathway |
| FGF12 | MAPK signaling pathway |
| FGFR2 | MAPK signaling pathway |
| FLNA | MAPK signaling pathway |
| FLNB | MAPK signaling pathway |
| FLNC | MAPK signaling pathway |
| MKNK2 | MAPK signaling pathway |
| HRAS | MAPK signaling pathway |
| HSPA8 | MAPK signaling pathway |
| IL1B | MAPK signaling pathway |
| IL1R1 | MAPK signaling pathway |
| MAPT | MAPK signaling pathway |
| MEF2C | MAPK signaling pathway |
| MAP3K1 | MAPK signaling pathway |
| MAP3K3 | MAPK signaling pathway |
| MAP3K4 | MAPK signaling pathway |
| MAP3K11 | MAPK signaling pathway |
| NF1 | MAPK signaling pathway |
| NFATC4 | MAPK signaling pathway |
| NFKB1 | MAPK signaling pathway |
| NTRK1 | MAPK signaling pathway |
| NTRK2 | MAPK signaling pathway |
| PAK1 | MAPK signaling pathway |
| PAK2 | MAPK signaling pathway |
| PDGFRA | MAPK signaling pathway |
| PLA2G4A | MAPK signaling pathway |
| PPM1A | MAPK signaling pathway |
| PPP3CB | MAPK signaling pathway |
| PPP3CC | MAPK signaling pathway |
| PPP5C | MAPK signaling pathway |
| PRKACA | MAPK signaling pathway |
| PRKACB | MAPK signaling pathway |
| PRKACG | MAPK signaling pathway |
| PRKCB | MAPK signaling pathway |
| MAPK7 | MAPK signaling pathway |
| MAPK8 | MAPK signaling pathway |
| MAPK9 | MAPK signaling pathway |
| MAP2K2 | MAPK signaling pathway |
| MAP2K3 | MAPK signaling pathway |
| MAP4K2 | MAPK signaling pathway |
| RAF1 | MAPK signaling pathway |
| RASA1 | MAPK signaling pathway |
| RASA2 | MAPK signaling pathway |
| RASGRF1 | MAPK signaling pathway |
| RASGRF2 | MAPK signaling pathway |
| RPS6KA3 | MAPK signaling pathway |
| SOS1 | MAPK signaling pathway |
| SOS2 | MAPK signaling pathway |
| STK3 | MAPK signaling pathway |
| STK4 | MAPK signaling pathway |
| MAP3K7 | MAPK signaling pathway |
| TGFBR1 | MAPK signaling pathway |
| TP53 | MAPK signaling pathway |
| TRAF6 | MAPK signaling pathway |
| MAP3K12 | MAPK signaling pathway |
| MAPKAPK3 | MAPK signaling pathway |
| MKNK1 | MAPK signaling pathway |
| PLA2G4C | MAPK signaling pathway |
| FGF17 | MAPK signaling pathway |
| CACNA1I | MAPK signaling pathway |
| CACNA1H | MAPK signaling pathway |
| CACNA1G | MAPK signaling pathway |
| MAP3K14 | MAPK signaling pathway |
| MAP3K6 | MAPK signaling pathway |
| MAP3K13 | MAPK signaling pathway |
| RPS6KA5 | MAPK signaling pathway |
| CACNA2D2 | MAPK signaling pathway |
| MAP4K4 | MAPK signaling pathway |
| MAPK8IP1 | MAPK signaling pathway |
| RAPGEF2 | MAPK signaling pathway |
| TAB1 | MAPK signaling pathway |
| TAB2 | MAPK signaling pathway |
| MAPK8IP2 | MAPK signaling pathway |
| RASGRP3 | MAPK signaling pathway |
| TAOK3 | MAPK signaling pathway |
| NLK | MAPK signaling pathway |
| CACNA2D3 | MAPK signaling pathway |
| DUSP22 | MAPK signaling pathway |
| TAOK1 | MAPK signaling pathway |
| CACNG7 | MAPK signaling pathway |
| DUSP16 | MAPK signaling pathway |
| PTPN5 | MAPK signaling pathway |
| CACNA2D4 | MAPK signaling pathway |
| PLA2G4F | MAPK signaling pathway |
| PLA2G4D | MAPK signaling pathway |
| ADCY2 | Calcium signaling pathway |
| ADCY3 | Calcium signaling pathway |
| ADCY8 | Calcium signaling pathway |
| ADCY9 | Calcium signaling pathway |
| ADORA2A | Calcium signaling pathway |
| ADRA1A | Calcium signaling pathway |
| ADRB2 | Calcium signaling pathway |
| SLC25A5 | Calcium signaling pathway |
| ATP2A1 | Calcium signaling pathway |
| ATP2A2 | Calcium signaling pathway |
| ATP2A3 | Calcium signaling pathway |
| ATP2B1 | Calcium signaling pathway |
| ATP2B2 | Calcium signaling pathway |
| ATP2B3 | Calcium signaling pathway |
| ATP2B4 | Calcium signaling pathway |
| CACNA1A | Calcium signaling pathway |
| CACNA1B | Calcium signaling pathway |
| CACNA1C | Calcium signaling pathway |
| CACNA1D | Calcium signaling pathway |
| CACNA1E | Calcium signaling pathway |
| CACNA1S | Calcium signaling pathway |
| CALML3 | Calcium signaling pathway |
| CCKAR | Calcium signaling pathway |
| CHRM1 | Calcium signaling pathway |
| CHRM2 | Calcium signaling pathway |
| CHRM3 | Calcium signaling pathway |
| EDNRA | Calcium signaling pathway |
| EGFR | Calcium signaling pathway |
| ERBB2 | Calcium signaling pathway |
| ERBB3 | Calcium signaling pathway |
| ERBB4 | Calcium signaling pathway |
| F2R | Calcium signaling pathway |
| PTK2B | Calcium signaling pathway |
| GNAL | Calcium signaling pathway |
| GNAS | Calcium signaling pathway |
| GRIN2A | Calcium signaling pathway |
| GRM1 | Calcium signaling pathway |
| GRM5 | Calcium signaling pathway |
| HRH2 | Calcium signaling pathway |
| HTR2A | Calcium signaling pathway |
| HTR2B | Calcium signaling pathway |
| HTR2C | Calcium signaling pathway |
| HTR7 | Calcium signaling pathway |
| ITPKB | Calcium signaling pathway |
| ITPR1 | Calcium signaling pathway |
| ITPR2 | Calcium signaling pathway |
| ITPR3 | Calcium signaling pathway |
| LHCGR | Calcium signaling pathway |
| MYLK | Calcium signaling pathway |
| NOS1 | Calcium signaling pathway |
| NOS2 | Calcium signaling pathway |
| NOS3 | Calcium signaling pathway |
| PDE1A | Calcium signaling pathway |
| PDE1C | Calcium signaling pathway |
| PDE1B | Calcium signaling pathway |
| PDGFRA | Calcium signaling pathway |
| PHKA1 | Calcium signaling pathway |
| PHKB | Calcium signaling pathway |
| PHKG1 | Calcium signaling pathway |
| PLCB2 | Calcium signaling pathway |
| PLCB3 | Calcium signaling pathway |
| PLCB4 | Calcium signaling pathway |
| PLCG1 | Calcium signaling pathway |
| PPP3CB | Calcium signaling pathway |
| PPP3CC | Calcium signaling pathway |
| PRKACA | Calcium signaling pathway |
| PRKACB | Calcium signaling pathway |
| PRKACG | Calcium signaling pathway |
| PRKCB | Calcium signaling pathway |
| RYR1 | Calcium signaling pathway |
| RYR2 | Calcium signaling pathway |
| RYR3 | Calcium signaling pathway |
| SLC8A2 | Calcium signaling pathway |
| SLC8A1 | Calcium signaling pathway |
| STIM1 | Calcium signaling pathway |
| TACR3 | Calcium signaling pathway |
| CACNA1I | Calcium signaling pathway |
| CACNA1H | Calcium signaling pathway |
| CACNA1G | Calcium signaling pathway |
| CYSLTR1 | Calcium signaling pathway |
| P2RX2 | Calcium signaling pathway |
| PLCB1 | Calcium signaling pathway |
| PLCE1 | Calcium signaling pathway |
| SPHK2 | Calcium signaling pathway |
| CYSLTR2 | Calcium signaling pathway |
| STIM2 | Calcium signaling pathway |
| ITPKC | Calcium signaling pathway |
| ORAI1 | Calcium signaling pathway |
| ATM | Cell cycle |
| ATR | Cell cycle |
| BUB1 | Cell cycle |
| BUB1B | Cell cycle |
| CCNB1 | Cell cycle |
| CCNH | Cell cycle |
| CDC6 | Cell cycle |
| CDC25C | Cell cycle |
| CDC27 | Cell cycle |
| CDKN1A | Cell cycle |
| CDKN2C | Cell cycle |
| CHEK1 | Cell cycle |
| CREBBP | Cell cycle |
| EP300 | Cell cycle |
| SFN | Cell cycle |
| HDAC2 | Cell cycle |
| SMAD2 | Cell cycle |
| SMAD3 | Cell cycle |
| MCM2 | Cell cycle |
| MCM3 | Cell cycle |
| MCM4 | Cell cycle |
| MCM6 | Cell cycle |
| MDM2 | Cell cycle |
| ORC2 | Cell cycle |
| PRKDC | Cell cycle |
| RB1 | Cell cycle |
| RBL1 | Cell cycle |
| RBL2 | Cell cycle |
| TP53 | Cell cycle |
| WEE1 | Cell cycle |
| YWHAB | Cell cycle |
| YWHAZ | Cell cycle |
| CDC7 | Cell cycle |
| CUL1 | Cell cycle |
| CDC16 | Cell cycle |
| CCNA1 | Cell cycle |
| SMC3 | Cell cycle |
| STAG1 | Cell cycle |
| MAD2L2 | Cell cycle |
| STAG2 | Cell cycle |
| PTTG2 | Cell cycle |
| ORC3 | Cell cycle |
| SMC1B | Cell cycle |
| ANAPC2 | Cell cycle |
| FZR1 | Cell cycle |
| ANAPC7 | Cell cycle |
| CCNB3 | Cell cycle |
| APAF1 | p53 signaling pathway |
| ATM | p53 signaling pathway |
| ATR | p53 signaling pathway |
| CCNB1 | p53 signaling pathway |
| CCNG1 | p53 signaling pathway |
| CCNG2 | p53 signaling pathway |
| CDKN1A | p53 signaling pathway |
| CHEK1 | p53 signaling pathway |
| SFN | p53 signaling pathway |
| IGFBP3 | p53 signaling pathway |
| CD82 | p53 signaling pathway |
| MDM2 | p53 signaling pathway |
| SERPINE1 | p53 signaling pathway |
| PTEN | p53 signaling pathway |
| THBS1 | p53 signaling pathway |
| TP53 | p53 signaling pathway |
| TSC2 | p53 signaling pathway |
| TNFRSF10B | p53 signaling pathway |
| TP53I3 | p53 signaling pathway |
| PERP | p53 signaling pathway |
| CCNB3 | p53 signaling pathway |
| ANGPT1 | PI3K-Akt signaling pathway |
| ANGPT2 | PI3K-Akt signaling pathway |
| ATF4 | PI3K-Akt signaling pathway |
| BRCA1 | PI3K-Akt signaling pathway |
| CDKN1A | PI3K-Akt signaling pathway |
| CHAD | PI3K-Akt signaling pathway |
| CHRM1 | PI3K-Akt signaling pathway |
| CHRM2 | PI3K-Akt signaling pathway |
| CHUK | PI3K-Akt signaling pathway |
| COL1A1 | PI3K-Akt signaling pathway |
| COL1A2 | PI3K-Akt signaling pathway |
| COL2A1 | PI3K-Akt signaling pathway |
| COL3A1 | PI3K-Akt signaling pathway |
| COL4A1 | PI3K-Akt signaling pathway |
| COL4A2 | PI3K-Akt signaling pathway |
| COL4A3 | PI3K-Akt signaling pathway |
| COL4A4 | PI3K-Akt signaling pathway |
| COL4A5 | PI3K-Akt signaling pathway |
| COL4A6 | PI3K-Akt signaling pathway |
| COL5A1 | PI3K-Akt signaling pathway |
| COL5A2 | PI3K-Akt signaling pathway |
| COL6A1 | PI3K-Akt signaling pathway |
| COL6A2 | PI3K-Akt signaling pathway |
| COL6A3 | PI3K-Akt signaling pathway |
| COL11A1 | PI3K-Akt signaling pathway |
| COL11A2 | PI3K-Akt signaling pathway |
| ATF2 | PI3K-Akt signaling pathway |
| ATF6B | PI3K-Akt signaling pathway |
| CSF1 | PI3K-Akt signaling pathway |
| CSF1R | PI3K-Akt signaling pathway |
| CSF3R | PI3K-Akt signaling pathway |
| LPAR1 | PI3K-Akt signaling pathway |
| EFNA5 | PI3K-Akt signaling pathway |
| EGFR | PI3K-Akt signaling pathway |
| EPHA2 | PI3K-Akt signaling pathway |
| F2R | PI3K-Akt signaling pathway |
| FGF5 | PI3K-Akt signaling pathway |
| FGF12 | PI3K-Akt signaling pathway |
| FGFR2 | PI3K-Akt signaling pathway |
| FLT1 | PI3K-Akt signaling pathway |
| FLT4 | PI3K-Akt signaling pathway |
| FN1 | PI3K-Akt signaling pathway |
| MTOR | PI3K-Akt signaling pathway |
| G6PC | PI3K-Akt signaling pathway |
| GH2 | PI3K-Akt signaling pathway |
| GNB2 | PI3K-Akt signaling pathway |
| GNG5 | PI3K-Akt signaling pathway |
| GNG11 | PI3K-Akt signaling pathway |
| GNGT1 | PI3K-Akt signaling pathway |
| GNGT2 | PI3K-Akt signaling pathway |
| GYS2 | PI3K-Akt signaling pathway |
| HGF | PI3K-Akt signaling pathway |
| HRAS | PI3K-Akt signaling pathway |
| HSP90AA1 | PI3K-Akt signaling pathway |
| HSP90AB1 | PI3K-Akt signaling pathway |
| TNC | PI3K-Akt signaling pathway |
| IBSP | PI3K-Akt signaling pathway |
| IFNA7 | PI3K-Akt signaling pathway |
| IFNA10 | PI3K-Akt signaling pathway |
| IFNAR1 | PI3K-Akt signaling pathway |
| IFNAR2 | PI3K-Akt signaling pathway |
| IGF1R | PI3K-Akt signaling pathway |
| IL2RB | PI3K-Akt signaling pathway |
| IL4R | PI3K-Akt signaling pathway |
| IL7R | PI3K-Akt signaling pathway |
| ITGA6 | PI3K-Akt signaling pathway |
| ITGA1 | PI3K-Akt signaling pathway |
| ITGA2 | PI3K-Akt signaling pathway |
| ITGA3 | PI3K-Akt signaling pathway |
| ITGA4 | PI3K-Akt signaling pathway |
| ITGA5 | PI3K-Akt signaling pathway |
| ITGA9 | PI3K-Akt signaling pathway |
| ITGAV | PI3K-Akt signaling pathway |
| ITGB1 | PI3K-Akt signaling pathway |
| ITGB4 | PI3K-Akt signaling pathway |
| ITGB5 | PI3K-Akt signaling pathway |
| ITGB6 | PI3K-Akt signaling pathway |
| JAK1 | PI3K-Akt signaling pathway |
| JAK2 | PI3K-Akt signaling pathway |
| JAK3 | PI3K-Akt signaling pathway |
| KDR | PI3K-Akt signaling pathway |
| KIT | PI3K-Akt signaling pathway |
| LAMA2 | PI3K-Akt signaling pathway |
| LAMA3 | PI3K-Akt signaling pathway |
| LAMA4 | PI3K-Akt signaling pathway |
| LAMA5 | PI3K-Akt signaling pathway |
| LAMB1 | PI3K-Akt signaling pathway |
| LAMB3 | PI3K-Akt signaling pathway |
| LAMC1 | PI3K-Akt signaling pathway |
| LAMC2 | PI3K-Akt signaling pathway |
| MCL1 | PI3K-Akt signaling pathway |
| MDM2 | PI3K-Akt signaling pathway |
| MET | PI3K-Akt signaling pathway |
| KITLG | PI3K-Akt signaling pathway |
| MYB | PI3K-Akt signaling pathway |
| NFKB1 | PI3K-Akt signaling pathway |
| NGFR | PI3K-Akt signaling pathway |
| NOS3 | PI3K-Akt signaling pathway |
| PCK1 | PI3K-Akt signaling pathway |
| PDGFRA | PI3K-Akt signaling pathway |
| PIK3CA | PI3K-Akt signaling pathway |
| PIK3CD | PI3K-Akt signaling pathway |
| PIK3CG | PI3K-Akt signaling pathway |
| PIK3R1 | PI3K-Akt signaling pathway |
| PPP2CB | PI3K-Akt signaling pathway |
| PPP2R1B | PI3K-Akt signaling pathway |
| PPP2R2A | PI3K-Akt signaling pathway |
| PPP2R2C | PI3K-Akt signaling pathway |
| PPP2R3A | PI3K-Akt signaling pathway |
| PKN2 | PI3K-Akt signaling pathway |
| MAP2K2 | PI3K-Akt signaling pathway |
| PRL | PI3K-Akt signaling pathway |
| RELN | PI3K-Akt signaling pathway |
| PTEN | PI3K-Akt signaling pathway |
| PTK2 | PI3K-Akt signaling pathway |
| RAF1 | PI3K-Akt signaling pathway |
| RBL2 | PI3K-Akt signaling pathway |
| RPS6KB1 | PI3K-Akt signaling pathway |
| RPS6KB2 | PI3K-Akt signaling pathway |
| SGK1 | PI3K-Akt signaling pathway |
| SOS1 | PI3K-Akt signaling pathway |
| SOS2 | PI3K-Akt signaling pathway |
| SYK | PI3K-Akt signaling pathway |
| TEK | PI3K-Akt signaling pathway |
| THBS1 | PI3K-Akt signaling pathway |
| THBS2 | PI3K-Akt signaling pathway |
| THBS3 | PI3K-Akt signaling pathway |
| THBS4 | PI3K-Akt signaling pathway |
| TLR4 | PI3K-Akt signaling pathway |
| TNR | PI3K-Akt signaling pathway |
| TNXB | PI3K-Akt signaling pathway |
| TP53 | PI3K-Akt signaling pathway |
| TSC1 | PI3K-Akt signaling pathway |
| TSC2 | PI3K-Akt signaling pathway |
| VEGFB | PI3K-Akt signaling pathway |
| VWF | PI3K-Akt signaling pathway |
| YWHAB | PI3K-Akt signaling pathway |
| YWHAZ | PI3K-Akt signaling pathway |
| ITGA10 | PI3K-Akt signaling pathway |
| ITGA8 | PI3K-Akt signaling pathway |
| FGF17 | PI3K-Akt signaling pathway |
| OSMR | PI3K-Akt signaling pathway |
| EIF4E2 | PI3K-Akt signaling pathway |
| CREB5 | PI3K-Akt signaling pathway |
| SGK2 | PI3K-Akt signaling pathway |
| LAMC3 | PI3K-Akt signaling pathway |
| GNB5 | PI3K-Akt signaling pathway |
| CDC37 | PI3K-Akt signaling pathway |
| ITGA11 | PI3K-Akt signaling pathway |
| PHLPP2 | PI3K-Akt signaling pathway |
| PHLPP1 | PI3K-Akt signaling pathway |
| LPAR3 | PI3K-Akt signaling pathway |
| COL5A3 | PI3K-Akt signaling pathway |
| ANGPT4 | PI3K-Akt signaling pathway |
| PPP2R2D | PI3K-Akt signaling pathway |
| RPTOR | PI3K-Akt signaling pathway |
| TNN | PI3K-Akt signaling pathway |
| CREB3L2 | PI3K-Akt signaling pathway |
| PDGFD | PI3K-Akt signaling pathway |
| COL27A1 | PI3K-Akt signaling pathway |
| COL6A6 | PI3K-Akt signaling pathway |
| COL24A1 | PI3K-Akt signaling pathway |
| LAMA1 | PI3K-Akt signaling pathway |
| APAF1 | Apoptosis |
| BIRC2 | Apoptosis |
| ATM | Apoptosis |
| CAPN1 | Apoptosis |
| CHUK | Apoptosis |
| CSF2RB | Apoptosis |
| IL1B | Apoptosis |
| IL1R1 | Apoptosis |
| IL1RAP | Apoptosis |
| IRAK1 | Apoptosis |
| NFKB1 | Apoptosis |
| NFKBIA | Apoptosis |
| NTRK1 | Apoptosis |
| PIK3CA | Apoptosis |
| PIK3CD | Apoptosis |
| PIK3CG | Apoptosis |
| PIK3R1 | Apoptosis |
| PPP3CB | Apoptosis |
| PPP3CC | Apoptosis |
| PRKACA | Apoptosis |
| PRKACB | Apoptosis |
| PRKACG | Apoptosis |
| PRKAR1A | Apoptosis |
| PRKAR1B | Apoptosis |
| PRKAR2A | Apoptosis |
| TP53 | Apoptosis |
| RIPK1 | Apoptosis |
| TNFRSF10D | Apoptosis |
| TNFRSF10B | Apoptosis |
| MAP3K14 | Apoptosis |
| AIFM1 | Apoptosis |
| APC | Wnt signaling pathway |
| RHOA | Wnt signaling pathway |
| CREBBP | Wnt signaling pathway |
| CSNK1E | Wnt signaling pathway |
| CSNK2B | Wnt signaling pathway |
| CTNNB1 | Wnt signaling pathway |
| DVL3 | Wnt signaling pathway |
| EP300 | Wnt signaling pathway |
| FZD2 | Wnt signaling pathway |
| LRP6 | Wnt signaling pathway |
| SMAD3 | Wnt signaling pathway |
| NFATC1 | Wnt signaling pathway |
| NFATC3 | Wnt signaling pathway |
| NFATC4 | Wnt signaling pathway |
| PLCB2 | Wnt signaling pathway |
| PLCB3 | Wnt signaling pathway |
| PLCB4 | Wnt signaling pathway |
| PPARD | Wnt signaling pathway |
| PPP3CB | Wnt signaling pathway |
| PPP3CC | Wnt signaling pathway |
| PRKACA | Wnt signaling pathway |
| PRKACB | Wnt signaling pathway |
| PRKACG | Wnt signaling pathway |
| PRKCB | Wnt signaling pathway |
| MAPK8 | Wnt signaling pathway |
| MAPK9 | Wnt signaling pathway |
| PSEN1 | Wnt signaling pathway |
| ROCK1 | Wnt signaling pathway |
| SFRP1 | Wnt signaling pathway |
| MAP3K7 | Wnt signaling pathway |
| TCF7L2 | Wnt signaling pathway |
| TP53 | Wnt signaling pathway |
| WNT2 | Wnt signaling pathway |
| WNT5A | Wnt signaling pathway |
| FZD3 | Wnt signaling pathway |
| AXIN1 | Wnt signaling pathway |
| AXIN2 | Wnt signaling pathway |
| FZD1 | Wnt signaling pathway |
| CUL1 | Wnt signaling pathway |
| BTRC | Wnt signaling pathway |
| CER1 | Wnt signaling pathway |
| ROCK2 | Wnt signaling pathway |
| NFAT5 | Wnt signaling pathway |
| DKK1 | Wnt signaling pathway |
| DAAM1 | Wnt signaling pathway |
| PLCB1 | Wnt signaling pathway |
| DAAM2 | Wnt signaling pathway |
| BAMBI | Wnt signaling pathway |
| DKK4 | Wnt signaling pathway |
| LEF1 | Wnt signaling pathway |
| NLK | Wnt signaling pathway |
| VANGL2 | Wnt signaling pathway |
| CHD8 | Wnt signaling pathway |
| TBL1XR1 | Wnt signaling pathway |
| CXXC4 | Wnt signaling pathway |
| WNT10A | Wnt signaling pathway |
| VANGL1 | Wnt signaling pathway |
| PRICKLE1 | Wnt signaling pathway |
| PRICKLE2 | Wnt signaling pathway |
| BIRC2 | Focal adhesion |
| RHOA | Focal adhesion |
| ARHGAP5 | Focal adhesion |
| BRAF | Focal adhesion |
| CHAD | Focal adhesion |
| COL1A1 | Focal adhesion |
| COL1A2 | Focal adhesion |
| COL2A1 | Focal adhesion |
| COL3A1 | Focal adhesion |
| COL4A1 | Focal adhesion |
| COL4A2 | Focal adhesion |
| COL4A3 | Focal adhesion |
| COL4A4 | Focal adhesion |
| COL4A5 | Focal adhesion |
| COL4A6 | Focal adhesion |
| COL5A1 | Focal adhesion |
| COL5A2 | Focal adhesion |
| COL6A1 | Focal adhesion |
| COL6A2 | Focal adhesion |
| COL6A3 | Focal adhesion |
| COL11A1 | Focal adhesion |
| COL11A2 | Focal adhesion |
| CTNNB1 | Focal adhesion |
| DOCK1 | Focal adhesion |
| EGFR | Focal adhesion |
| ELK1 | Focal adhesion |
| ERBB2 | Focal adhesion |
| FLNA | Focal adhesion |
| FLNB | Focal adhesion |
| FLNC | Focal adhesion |
| FLT1 | Focal adhesion |
| FLT4 | Focal adhesion |
| FN1 | Focal adhesion |
| FYN | Focal adhesion |
| ARHGAP35 | Focal adhesion |
| HGF | Focal adhesion |
| HRAS | Focal adhesion |
| TNC | Focal adhesion |
| IBSP | Focal adhesion |
| IGF1R | Focal adhesion |
| ITGA6 | Focal adhesion |
| ITGA1 | Focal adhesion |
| ITGA2 | Focal adhesion |
| ITGA3 | Focal adhesion |
| ITGA4 | Focal adhesion |
| ITGA5 | Focal adhesion |
| ITGA9 | Focal adhesion |
| ITGAV | Focal adhesion |
| ITGB1 | Focal adhesion |
| ITGB4 | Focal adhesion |
| ITGB5 | Focal adhesion |
| ITGB6 | Focal adhesion |
| KDR | Focal adhesion |
| LAMA2 | Focal adhesion |
| LAMA3 | Focal adhesion |
| LAMA4 | Focal adhesion |
| LAMA5 | Focal adhesion |
| LAMB1 | Focal adhesion |
| LAMB3 | Focal adhesion |
| LAMC1 | Focal adhesion |
| LAMC2 | Focal adhesion |
| MET | Focal adhesion |
| MYLK | Focal adhesion |
| PPP1R12A | Focal adhesion |
| PAK1 | Focal adhesion |
| PAK2 | Focal adhesion |
| PAK3 | Focal adhesion |
| PDGFRA | Focal adhesion |
| PIK3CA | Focal adhesion |
| PIK3CD | Focal adhesion |
| PIK3CG | Focal adhesion |
| PIK3R1 | Focal adhesion |
| PRKCB | Focal adhesion |
| MAPK8 | Focal adhesion |
| MAPK9 | Focal adhesion |
| RELN | Focal adhesion |
| PTEN | Focal adhesion |
| PTK2 | Focal adhesion |
| RAF1 | Focal adhesion |
| RASGRF1 | Focal adhesion |
| ROCK1 | Focal adhesion |
| SOS1 | Focal adhesion |
| SOS2 | Focal adhesion |
| THBS1 | Focal adhesion |
| THBS2 | Focal adhesion |
| THBS3 | Focal adhesion |
| THBS4 | Focal adhesion |
| TNR | Focal adhesion |
| TNXB | Focal adhesion |
| VAV1 | Focal adhesion |
| VAV2 | Focal adhesion |
| VCL | Focal adhesion |
| VEGFB | Focal adhesion |
| VWF | Focal adhesion |
| ITGA10 | Focal adhesion |
| ITGA8 | Focal adhesion |
| ROCK2 | Focal adhesion |
| BCAR1 | Focal adhesion |
| PAK4 | Focal adhesion |
| LAMC3 | Focal adhesion |
| VAV3 | Focal adhesion |
| MYL12A | Focal adhesion |
| ITGA11 | Focal adhesion |
| PIP5K1C | Focal adhesion |
| COL5A3 | Focal adhesion |
| PAK6 | Focal adhesion |
| PAK7 | Focal adhesion |
| TNN | Focal adhesion |
| PDGFD | Focal adhesion |
| COL27A1 | Focal adhesion |
| COL6A6 | Focal adhesion |
| COL24A1 | Focal adhesion |
| LAMA1 | Focal adhesion |
| SHC4 | Focal adhesion |
| CHAD | ECM-receptor interaction |
| COL1A1 | ECM-receptor interaction |
| COL1A2 | ECM-receptor interaction |
| COL2A1 | ECM-receptor interaction |
| COL3A1 | ECM-receptor interaction |
| COL4A1 | ECM-receptor interaction |
| COL4A2 | ECM-receptor interaction |
| COL4A3 | ECM-receptor interaction |
| COL4A4 | ECM-receptor interaction |
| COL4A5 | ECM-receptor interaction |
| COL4A6 | ECM-receptor interaction |
| COL5A1 | ECM-receptor interaction |
| COL5A2 | ECM-receptor interaction |
| COL6A1 | ECM-receptor interaction |
| COL6A2 | ECM-receptor interaction |
| COL6A3 | ECM-receptor interaction |
| COL11A1 | ECM-receptor interaction |
| COL11A2 | ECM-receptor interaction |
| DAG1 | ECM-receptor interaction |
| FN1 | ECM-receptor interaction |
| HMMR | ECM-receptor interaction |
| HSPG2 | ECM-receptor interaction |
| TNC | ECM-receptor interaction |
| IBSP | ECM-receptor interaction |
| ITGA6 | ECM-receptor interaction |
| ITGA1 | ECM-receptor interaction |
| ITGA2 | ECM-receptor interaction |
| ITGA3 | ECM-receptor interaction |
| ITGA4 | ECM-receptor interaction |
| ITGA5 | ECM-receptor interaction |
| ITGA9 | ECM-receptor interaction |
| ITGAV | ECM-receptor interaction |
| ITGB1 | ECM-receptor interaction |
| ITGB4 | ECM-receptor interaction |
| ITGB5 | ECM-receptor interaction |
| ITGB6 | ECM-receptor interaction |
| LAMA2 | ECM-receptor interaction |
| LAMA3 | ECM-receptor interaction |
| LAMA4 | ECM-receptor interaction |
| LAMA5 | ECM-receptor interaction |
| LAMB1 | ECM-receptor interaction |
| LAMB3 | ECM-receptor interaction |
| LAMC1 | ECM-receptor interaction |
| LAMC2 | ECM-receptor interaction |
| RELN | ECM-receptor interaction |
| THBS1 | ECM-receptor interaction |
| THBS2 | ECM-receptor interaction |
| THBS3 | ECM-receptor interaction |
| THBS4 | ECM-receptor interaction |
| TNR | ECM-receptor interaction |
| TNXB | ECM-receptor interaction |
| VWF | ECM-receptor interaction |
| ITGA10 | ECM-receptor interaction |
| ITGA8 | ECM-receptor interaction |
| LAMC3 | ECM-receptor interaction |
| ITGA11 | ECM-receptor interaction |
| COL5A3 | ECM-receptor interaction |
| TNN | ECM-receptor interaction |
| COL27A1 | ECM-receptor interaction |
| COL6A6 | ECM-receptor interaction |
| COL24A1 | ECM-receptor interaction |
| LAMA1 | ECM-receptor interaction |
| AGRN | ECM-receptor interaction |
| RHOA | Adherens junction |
| CREBBP | Adherens junction |
| CSNK2B | Adherens junction |
| CTNNA2 | Adherens junction |
| CTNNB1 | Adherens junction |
| CTNND1 | Adherens junction |
| EGFR | Adherens junction |
| EP300 | Adherens junction |
| ERBB2 | Adherens junction |
| FYN | Adherens junction |
| IGF1R | Adherens junction |
| LMO7 | Adherens junction |
| SMAD2 | Adherens junction |
| SMAD3 | Adherens junction |
| MET | Adherens junction |
| MLLT4 | Adherens junction |
| PTPN1 | Adherens junction |
| PTPRB | Adherens junction |
| PTPRF | Adherens junction |
| PTPRJ | Adherens junction |
| PTPRM | Adherens junction |
| PVRL1 | Adherens junction |
| MAP3K7 | Adherens junction |
| TCF7L2 | Adherens junction |
| TGFBR1 | Adherens junction |
| TJP1 | Adherens junction |
| VCL | Adherens junction |
| YES1 | Adherens junction |
| IQGAP1 | Adherens junction |
| BAIAP2 | Adherens junction |
| CTNNA3 | Adherens junction |
| LEF1 | Adherens junction |
| NLK | Adherens junction |
| PARD3 | Adherens junction |
| RHOA | Tight junction |
| CSNK2B | Tight junction |
| CTNNA2 | Tight junction |
| CTNNB1 | Tight junction |
| CTTN | Tight junction |
| EPB41 | Tight junction |
| EPB41L1 | Tight junction |
| EPB41L2 | Tight junction |
| GNAI2 | Tight junction |
| HRAS | Tight junction |
| LLGL2 | Tight junction |
| LLGL1 | Tight junction |
| MLLT4 | Tight junction |
| MYH1 | Tight junction |
| MYH2 | Tight junction |
| MYH4 | Tight junction |
| MYH6 | Tight junction |
| MYH7 | Tight junction |
| MYH8 | Tight junction |
| MYH9 | Tight junction |
| MYH10 | Tight junction |
| MYH11 | Tight junction |
| CLDN11 | Tight junction |
| PPP2CB | Tight junction |
| PPP2R1B | Tight junction |
| PPP2R2A | Tight junction |
| PPP2R2C | Tight junction |
| PRKCB | Tight junction |
| PRKCH | Tight junction |
| PRKCI | Tight junction |
| PRKCQ | Tight junction |
| PTEN | Tight junction |
| SPTAN1 | Tight junction |
| TJP1 | Tight junction |
| CLDN5 | Tight junction |
| YES1 | Tight junction |
| SYMPK | Tight junction |
| CASK | Tight junction |
| MYH13 | Tight junction |
| VAPA | Tight junction |
| MAGI1 | Tight junction |
| TJP2 | Tight junction |
| MAGI2 | Tight junction |
| INADL | Tight junction |
| MYL12A | Tight junction |
| MYH15 | Tight junction |
| EPB41L3 | Tight junction |
| CLDN14 | Tight junction |
| CTNNA3 | Tight junction |
| CLDN20 | Tight junction |
| CLDN18 | Tight junction |
| CLDN22 | Tight junction |
| PPP2R2D | Tight junction |
| ASH1L | Tight junction |
| PARD3 | Tight junction |
| CGN | Tight junction |
| JAM2 | Tight junction |
| EXOC4 | Tight junction |
| MYH14 | Tight junction |
| OCLN | Tight junction |
| RHOA | Leukocyte transendothelial migration |
| ARHGAP5 | Leukocyte transendothelial migration |
| CDH5 | Leukocyte transendothelial migration |
| CTNNA2 | Leukocyte transendothelial migration |
| CTNNB1 | Leukocyte transendothelial migration |
| CTNND1 | Leukocyte transendothelial migration |
| CYBA | Leukocyte transendothelial migration |
| PTK2B | Leukocyte transendothelial migration |
| GNAI2 | Leukocyte transendothelial migration |
| ARHGAP35 | Leukocyte transendothelial migration |
| ITGA4 | Leukocyte transendothelial migration |
| ITGAM | Leukocyte transendothelial migration |
| ITGB1 | Leukocyte transendothelial migration |
| ITGB2 | Leukocyte transendothelial migration |
| ITK | Leukocyte transendothelial migration |
| MLLT4 | Leukocyte transendothelial migration |
| MMP2 | Leukocyte transendothelial migration |
| MMP9 | Leukocyte transendothelial migration |
| CLDN11 | Leukocyte transendothelial migration |
| PIK3CA | Leukocyte transendothelial migration |
| PIK3CD | Leukocyte transendothelial migration |
| PIK3CG | Leukocyte transendothelial migration |
| PIK3R1 | Leukocyte transendothelial migration |
| PLCG1 | Leukocyte transendothelial migration |
| PRKCB | Leukocyte transendothelial migration |
| PTK2 | Leukocyte transendothelial migration |
| PTPN11 | Leukocyte transendothelial migration |
| ROCK1 | Leukocyte transendothelial migration |
| CXCL12 | Leukocyte transendothelial migration |
| CLDN5 | Leukocyte transendothelial migration |
| TXK | Leukocyte transendothelial migration |
| VAV1 | Leukocyte transendothelial migration |
| VAV2 | Leukocyte transendothelial migration |
| VCAM1 | Leukocyte transendothelial migration |
| VCL | Leukocyte transendothelial migration |
| EZR | Leukocyte transendothelial migration |
| ROCK2 | Leukocyte transendothelial migration |
| BCAR1 | Leukocyte transendothelial migration |
| RAPGEF3 | Leukocyte transendothelial migration |
| VAV3 | Leukocyte transendothelial migration |
| MYL12A | Leukocyte transendothelial migration |
| RAPGEF4 | Leukocyte transendothelial migration |
| CLDN14 | Leukocyte transendothelial migration |
| NOX1 | Leukocyte transendothelial migration |
| CTNNA3 | Leukocyte transendothelial migration |
| CLDN20 | Leukocyte transendothelial migration |
| NOX3 | Leukocyte transendothelial migration |
| CLDN18 | Leukocyte transendothelial migration |
| CLDN22 | Leukocyte transendothelial migration |
| JAM2 | Leukocyte transendothelial migration |
| ESAM | Leukocyte transendothelial migration |
| OCLN | Leukocyte transendothelial migration |
| RHOA | Neurotrophin signaling pathway |
| ARHGDIA | Neurotrophin signaling pathway |
| ARHGDIB | Neurotrophin signaling pathway |
| ARHGDIG | Neurotrophin signaling pathway |
| ATF4 | Neurotrophin signaling pathway |
| BRAF | Neurotrophin signaling pathway |
| CALML3 | Neurotrophin signaling pathway |
| GAB1 | Neurotrophin signaling pathway |
| HRAS | Neurotrophin signaling pathway |
| IRAK1 | Neurotrophin signaling pathway |
| MAP3K1 | Neurotrophin signaling pathway |
| MAP3K3 | Neurotrophin signaling pathway |
| NFKB1 | Neurotrophin signaling pathway |
| NFKBIA | Neurotrophin signaling pathway |
| NGFR | Neurotrophin signaling pathway |
| NTRK1 | Neurotrophin signaling pathway |
| NTRK2 | Neurotrophin signaling pathway |
| NTRK3 | Neurotrophin signaling pathway |
| PIK3CA | Neurotrophin signaling pathway |
| PIK3CD | Neurotrophin signaling pathway |
| PIK3CG | Neurotrophin signaling pathway |
| PIK3R1 | Neurotrophin signaling pathway |
| PLCG1 | Neurotrophin signaling pathway |
| MAPK7 | Neurotrophin signaling pathway |
| MAPK8 | Neurotrophin signaling pathway |
| MAPK9 | Neurotrophin signaling pathway |
| MAP2K2 | Neurotrophin signaling pathway |
| PSEN1 | Neurotrophin signaling pathway |
| PTPN11 | Neurotrophin signaling pathway |
| RAF1 | Neurotrophin signaling pathway |
| RPS6KA3 | Neurotrophin signaling pathway |
| SOS1 | Neurotrophin signaling pathway |
| SOS2 | Neurotrophin signaling pathway |
| TP53 | Neurotrophin signaling pathway |
| TRAF6 | Neurotrophin signaling pathway |
| RPS6KA5 | Neurotrophin signaling pathway |
| SHC4 | Neurotrophin signaling pathway |
| ADCY2 | Retrograde endocannabinoid signaling |
| ADCY3 | Retrograde endocannabinoid signaling |
| ADCY6 | Retrograde endocannabinoid signaling |
| ADCY8 | Retrograde endocannabinoid signaling |
| ADCY9 | Retrograde endocannabinoid signaling |
| DAGLA | Retrograde endocannabinoid signaling |
| CACNA1A | Retrograde endocannabinoid signaling |
| CACNA1B | Retrograde endocannabinoid signaling |
| CACNA1C | Retrograde endocannabinoid signaling |
| CACNA1D | Retrograde endocannabinoid signaling |
| CACNA1S | Retrograde endocannabinoid signaling |
| CNR1 | Retrograde endocannabinoid signaling |
| GABRA1 | Retrograde endocannabinoid signaling |
| GABRA2 | Retrograde endocannabinoid signaling |
| GABRA5 | Retrograde endocannabinoid signaling |
| GABRA6 | Retrograde endocannabinoid signaling |
| GABRB2 | Retrograde endocannabinoid signaling |
| GABRB3 | Retrograde endocannabinoid signaling |
| GABRG1 | Retrograde endocannabinoid signaling |
| GABRG2 | Retrograde endocannabinoid signaling |
| GABRG3 | Retrograde endocannabinoid signaling |
| GNAI2 | Retrograde endocannabinoid signaling |
| GNB2 | Retrograde endocannabinoid signaling |
| GNG5 | Retrograde endocannabinoid signaling |
| GNG11 | Retrograde endocannabinoid signaling |
| GNGT1 | Retrograde endocannabinoid signaling |
| GNGT2 | Retrograde endocannabinoid signaling |
| GRIA1 | Retrograde endocannabinoid signaling |
| GRIA2 | Retrograde endocannabinoid signaling |
| GRIA4 | Retrograde endocannabinoid signaling |
| GRM1 | Retrograde endocannabinoid signaling |
| GRM5 | Retrograde endocannabinoid signaling |
| ITPR1 | Retrograde endocannabinoid signaling |
| ITPR2 | Retrograde endocannabinoid signaling |
| ITPR3 | Retrograde endocannabinoid signaling |
| KCNJ5 | Retrograde endocannabinoid signaling |
| KCNJ6 | Retrograde endocannabinoid signaling |
| KCNJ9 | Retrograde endocannabinoid signaling |
| PLCB2 | Retrograde endocannabinoid signaling |
| PLCB3 | Retrograde endocannabinoid signaling |
| PLCB4 | Retrograde endocannabinoid signaling |
| PRKACA | Retrograde endocannabinoid signaling |
| PRKACB | Retrograde endocannabinoid signaling |
| PRKACG | Retrograde endocannabinoid signaling |
| PRKCB | Retrograde endocannabinoid signaling |
| MAPK8 | Retrograde endocannabinoid signaling |
| MAPK9 | Retrograde endocannabinoid signaling |
| PTGS2 | Retrograde endocannabinoid signaling |
| GNB5 | Retrograde endocannabinoid signaling |
| MGLL | Retrograde endocannabinoid signaling |
| RIMS1 | Retrograde endocannabinoid signaling |
| PLCB1 | Retrograde endocannabinoid signaling |
| SLC17A7 | Retrograde endocannabinoid signaling |
| SLC17A6 | Retrograde endocannabinoid signaling |
| ABHD6 | Retrograde endocannabinoid signaling |
| GABRR3 | Retrograde endocannabinoid signaling |
| DAGLB | Retrograde endocannabinoid signaling |
| NAPEPLD | Retrograde endocannabinoid signaling |
| ADCY2 | Glutamatergic synapse |
| ADCY3 | Glutamatergic synapse |
| ADCY6 | Glutamatergic synapse |
| ADCY8 | Glutamatergic synapse |
| ADCY9 | Glutamatergic synapse |
| ADRBK1 | Glutamatergic synapse |
| CACNA1A | Glutamatergic synapse |
| CACNA1C | Glutamatergic synapse |
| CACNA1D | Glutamatergic synapse |
| DLG4 | Glutamatergic synapse |
| GLUL | Glutamatergic synapse |
| GNAI2 | Glutamatergic synapse |
| GNAS | Glutamatergic synapse |
| GNB2 | Glutamatergic synapse |
| GNG5 | Glutamatergic synapse |
| GNG11 | Glutamatergic synapse |
| GNGT1 | Glutamatergic synapse |
| GNGT2 | Glutamatergic synapse |
| GRIA1 | Glutamatergic synapse |
| GRIA2 | Glutamatergic synapse |
| GRIA4 | Glutamatergic synapse |
| GRIK2 | Glutamatergic synapse |
| GRIK3 | Glutamatergic synapse |
| GRIK4 | Glutamatergic synapse |
| GRIK5 | Glutamatergic synapse |
| GRIN2A | Glutamatergic synapse |
| GRIN2B | Glutamatergic synapse |
| GRM1 | Glutamatergic synapse |
| GRM2 | Glutamatergic synapse |
| GRM3 | Glutamatergic synapse |
| GRM4 | Glutamatergic synapse |
| GRM5 | Glutamatergic synapse |
| GRM7 | Glutamatergic synapse |
| GRM8 | Glutamatergic synapse |
| ITPR1 | Glutamatergic synapse |
| ITPR2 | Glutamatergic synapse |
| ITPR3 | Glutamatergic synapse |
| PLA2G4A | Glutamatergic synapse |
| PLCB2 | Glutamatergic synapse |
| PLCB3 | Glutamatergic synapse |
| PLCB4 | Glutamatergic synapse |
| PPP3CB | Glutamatergic synapse |
| PPP3CC | Glutamatergic synapse |
| PRKACA | Glutamatergic synapse |
| PRKACB | Glutamatergic synapse |
| PRKACG | Glutamatergic synapse |
| PRKCB | Glutamatergic synapse |
| SLC1A3 | Glutamatergic synapse |
| SLC1A6 | Glutamatergic synapse |
| TRPC1 | Glutamatergic synapse |
| PLA2G4C | Glutamatergic synapse |
| DLGAP1 | Glutamatergic synapse |
| HOMER2 | Glutamatergic synapse |
| HOMER1 | Glutamatergic synapse |
| GNB5 | Glutamatergic synapse |
| SHANK2 | Glutamatergic synapse |
| PLCB1 | Glutamatergic synapse |
| GLS2 | Glutamatergic synapse |
| SHANK1 | Glutamatergic synapse |
| SLC17A7 | Glutamatergic synapse |
| SLC17A6 | Glutamatergic synapse |
| SHANK3 | Glutamatergic synapse |
| PLA2G4F | Glutamatergic synapse |
| PLA2G4D | Glutamatergic synapse |
| ADCY2 | Insulin secretion |
| ADCY3 | Insulin secretion |
| ADCY6 | Insulin secretion |
| ADCY8 | Insulin secretion |
| ADCY9 | Insulin secretion |
| ADCYAP1R1 | Insulin secretion |
| ATF4 | Insulin secretion |
| ATP1A1 | Insulin secretion |
| ATP1A3 | Insulin secretion |
| ATP1A4 | Insulin secretion |
| CACNA1C | Insulin secretion |
| CACNA1D | Insulin secretion |
| CACNA1S | Insulin secretion |
| CCKAR | Insulin secretion |
| CHRM3 | Insulin secretion |
| ATF2 | Insulin secretion |
| ATF6B | Insulin secretion |
| GCG | Insulin secretion |
| GCK | Insulin secretion |
| GNAS | Insulin secretion |
| ITPR3 | Insulin secretion |
| KCNMA1 | Insulin secretion |
| KCNN2 | Insulin secretion |
| KCNN3 | Insulin secretion |
| PLCB2 | Insulin secretion |
| PLCB3 | Insulin secretion |
| PLCB4 | Insulin secretion |
| PRKACA | Insulin secretion |
| PRKACB | Insulin secretion |
| PRKACG | Insulin secretion |
| PRKCB | Insulin secretion |
| RYR2 | Insulin secretion |
| SLC2A1 | Insulin secretion |
| SLC2A2 | Insulin secretion |
| ABCC8 | Insulin secretion |
| CREB5 | Insulin secretion |
| RIMS2 | Insulin secretion |
| KCNMB2 | Insulin secretion |
| RAPGEF4 | Insulin secretion |
| PLCB1 | Insulin secretion |
| ATP1B4 | Insulin secretion |
| PCLO | Insulin secretion |
| CREB3L2 | Insulin secretion |
| GPR119 | Insulin secretion |
| KCNU1 | Insulin secretion |
| ADCY2 | Circadian entrainment |
| ADCY3 | Circadian entrainment |
| ADCY6 | Circadian entrainment |
| ADCY8 | Circadian entrainment |
| ADCY9 | Circadian entrainment |
| ADCYAP1R1 | Circadian entrainment |
| CACNA1C | Circadian entrainment |
| CACNA1D | Circadian entrainment |
| CALML3 | Circadian entrainment |
| GNAI2 | Circadian entrainment |
| GNAS | Circadian entrainment |
| GNB2 | Circadian entrainment |
| GNG5 | Circadian entrainment |
| GNG11 | Circadian entrainment |
| GNGT1 | Circadian entrainment |
| GNGT2 | Circadian entrainment |
| GRIA1 | Circadian entrainment |
| GRIA2 | Circadian entrainment |
| GRIA4 | Circadian entrainment |
| GRIN2A | Circadian entrainment |
| GRIN2B | Circadian entrainment |
| GUCY1A2 | Circadian entrainment |
| GUCY1A3 | Circadian entrainment |
| ITPR1 | Circadian entrainment |
| ITPR3 | Circadian entrainment |
| KCNJ5 | Circadian entrainment |
| KCNJ6 | Circadian entrainment |
| KCNJ9 | Circadian entrainment |
| NOS1 | Circadian entrainment |
| PLCB2 | Circadian entrainment |
| PLCB3 | Circadian entrainment |
| PLCB4 | Circadian entrainment |
| PRKACA | Circadian entrainment |
| PRKACB | Circadian entrainment |
| PRKACG | Circadian entrainment |
| PRKCB | Circadian entrainment |
| PRKG1 | Circadian entrainment |
| PRKG2 | Circadian entrainment |
| RYR1 | Circadian entrainment |
| RYR2 | Circadian entrainment |
| RYR3 | Circadian entrainment |
| PER3 | Circadian entrainment |
| PER2 | Circadian entrainment |
| CACNA1I | Circadian entrainment |
| CACNA1H | Circadian entrainment |
| CACNA1G | Circadian entrainment |
| RPS6KA5 | Circadian entrainment |
| NOS1AP | Circadian entrainment |
| GNB5 | Circadian entrainment |
| PLCB1 | Circadian entrainment |
| ADCY3 | Olfactory transduction |
| ARRB2 | Olfactory transduction |
| CALML3 | Olfactory transduction |
| CNGB1 | Olfactory transduction |
| GNAL | Olfactory transduction |
| GUCA1A | Olfactory transduction |
| GUCA1B | Olfactory transduction |
| GUCY2D | Olfactory transduction |
| OR1D2 | Olfactory transduction |
| OR2C1 | Olfactory transduction |
| OR3A1 | Olfactory transduction |
| PDC | Olfactory transduction |
| PDE1C | Olfactory transduction |
| PRKACA | Olfactory transduction |
| PRKACB | Olfactory transduction |
| PRKACG | Olfactory transduction |
| PRKG1 | Olfactory transduction |
| PRKG2 | Olfactory transduction |
| OR1A1 | Olfactory transduction |
| OR1E1 | Olfactory transduction |
| OR1E2 | Olfactory transduction |
| GUCA1C | Olfactory transduction |
| CLCA2 | Olfactory transduction |
| OR5I1 | Olfactory transduction |
| CLCA4 | Olfactory transduction |
| OR52A1 | Olfactory transduction |
| OR2L2 | Olfactory transduction |
| OR2K2 | Olfactory transduction |
| OR5L2 | Olfactory transduction |
| OR5H1 | Olfactory transduction |
| OR10J1 | Olfactory transduction |
| OR8B8 | Olfactory transduction |
| OR8G1 | Olfactory transduction |
| OR10A3 | Olfactory transduction |
| OR12D2 | Olfactory transduction |
| OR10H3 | Olfactory transduction |
| OR7C2 | Olfactory transduction |
| OR7A5 | Olfactory transduction |
| OR4E2 | Olfactory transduction |
| OR2T1 | Olfactory transduction |
| OR2J2 | Olfactory transduction |
| OR4K5 | Olfactory transduction |
| OR51G1 | Olfactory transduction |
| OR51B2 | Olfactory transduction |
| OR4K1 | Olfactory transduction |
| OR5AC2 | Olfactory transduction |
| OR51G2 | Olfactory transduction |
| OR51E2 | Olfactory transduction |
| OR4A5 | Olfactory transduction |
| OR4A16 | Olfactory transduction |
| OR4A15 | Olfactory transduction |
| OR6N2 | Olfactory transduction |
| OR6K2 | Olfactory transduction |
| OR2L5 | Olfactory transduction |
| OR2G3 | Olfactory transduction |
| OR2G2 | Olfactory transduction |
| OR2C3 | Olfactory transduction |
| OR12D3 | Olfactory transduction |
| OR52J3 | Olfactory transduction |
| OR51A7 | Olfactory transduction |
| OR52R1 | Olfactory transduction |
| OR56A4 | Olfactory transduction |
| OR2AP1 | Olfactory transduction |
| OR10A7 | Olfactory transduction |
| OR11H6 | Olfactory transduction |
| OR7G1 | Olfactory transduction |
| OR10H4 | Olfactory transduction |
| OR2M5 | Olfactory transduction |
| OR2M3 | Olfactory transduction |
| OR2T12 | Olfactory transduction |
| OR14C36 | Olfactory transduction |
| OR2T4 | Olfactory transduction |
| OR2B11 | Olfactory transduction |
| OR10Z1 | Olfactory transduction |
| OR2Y1 | Olfactory transduction |
| OR2A14 | Olfactory transduction |
| OR6B1 | Olfactory transduction |
| OR2F2 | Olfactory transduction |
| OR13C3 | Olfactory transduction |
| OR1L8 | Olfactory transduction |
| OR52B4 | Olfactory transduction |
| OR52I2 | Olfactory transduction |
| OR51E1 | Olfactory transduction |
| OR6B3 | Olfactory transduction |
| OR1Q1 | Olfactory transduction |
| OR4C16 | Olfactory transduction |
| OR5L1 | Olfactory transduction |
| OR5AS1 | Olfactory transduction |
| OR8K5 | Olfactory transduction |
| OR5T2 | Olfactory transduction |
| OR8H1 | Olfactory transduction |
| OR8K3 | Olfactory transduction |
| OR5R1 | Olfactory transduction |
| OR5M3 | Olfactory transduction |
| OR5M8 | Olfactory transduction |
| OR5AR1 | Olfactory transduction |
| OR8B12 | Olfactory transduction |
| OR8G5 | Olfactory transduction |
| OR10G8 | Olfactory transduction |
| OR9I1 | Olfactory transduction |
| OR9Q1 | Olfactory transduction |
| OR1S1 | Olfactory transduction |
| OR5B17 | Olfactory transduction |
| OR5A2 | Olfactory transduction |
| OR4D11 | Olfactory transduction |
| OR6C74 | Olfactory transduction |
| OR6C3 | Olfactory transduction |
| OR51B5 | Olfactory transduction |
| OR10AG1 | Olfactory transduction |
| OR5J2 | Olfactory transduction |
| OR4C12 | Olfactory transduction |
| OR8D2 | Olfactory transduction |
| OR9G4 | Olfactory transduction |
| OR10A4 | Olfactory transduction |
| OR2Z1 | Olfactory transduction |
| OR10H5 | Olfactory transduction |
| OR14A16 | Olfactory transduction |
| OR8D4 | Olfactory transduction |
| OR5F1 | Olfactory transduction |
| OR5AP2 | Olfactory transduction |
| OR10A2 | Olfactory transduction |
| OR6C2 | Olfactory transduction |
| OR8S1 | Olfactory transduction |
| OR10R2 | Olfactory transduction |
| OR6V1 | Olfactory transduction |
| OR2A12 | Olfactory transduction |
| OR1B1 | Olfactory transduction |
| OR52K1 | Olfactory transduction |
| OR52I1 | Olfactory transduction |
| OR51D1 | Olfactory transduction |
| OR51B6 | Olfactory transduction |
| OR51Q1 | Olfactory transduction |
| OR52N4 | Olfactory transduction |
| OR52N5 | Olfactory transduction |
| OR52N2 | Olfactory transduction |
| OR52E6 | Olfactory transduction |
| OR52E4 | Olfactory transduction |
| OR56A3 | Olfactory transduction |
| OR4X1 | Olfactory transduction |
| OR5D13 | Olfactory transduction |
| OR8H2 | Olfactory transduction |
| OR5T1 | Olfactory transduction |
| OR8K1 | Olfactory transduction |
| OR5M9 | Olfactory transduction |
| OR5M10 | Olfactory transduction |
| OR5M1 | Olfactory transduction |
| OR5B12 | Olfactory transduction |
| OR10V1 | Olfactory transduction |
| OR6M1 | Olfactory transduction |
| OR10G7 | Olfactory transduction |
| OR6C1 | Olfactory transduction |
| OR6C75 | Olfactory transduction |
| OR6C76 | Olfactory transduction |
| OR6C70 | Olfactory transduction |
| OR4N2 | Olfactory transduction |
| OR4N5 | Olfactory transduction |
| OR4M2 | Olfactory transduction |
| OR4F6 | Olfactory transduction |
| OR7G3 | Olfactory transduction |
| OR10K2 | Olfactory transduction |
| OR10K1 | Olfactory transduction |
| OR6Y1 | Olfactory transduction |
| OR2AK2 | Olfactory transduction |
| OR2L3 | Olfactory transduction |
| OR2G6 | Olfactory transduction |
| OR13J1 | Olfactory transduction |
| OR2A5 | Olfactory transduction |
| OR51A4 | Olfactory transduction |
| OR2T27 | Olfactory transduction |
| OR4A47 | Olfactory transduction |
| OR4C45 | Olfactory transduction |
| OR6C65 | Olfactory transduction |
| OR5B3 | Olfactory transduction |
| OR4Q3 | Olfactory transduction |
| OR10J3 | Olfactory transduction |
| OR2J3 | Olfactory transduction |
| OR10C1 | Olfactory transduction |
| ADCY2 | Melanogenesis |
| ADCY3 | Melanogenesis |
| ADCY6 | Melanogenesis |
| ADCY8 | Melanogenesis |
| ADCY9 | Melanogenesis |
| CALML3 | Melanogenesis |
| CREBBP | Melanogenesis |
| CTNNB1 | Melanogenesis |
| DCT | Melanogenesis |
| DVL3 | Melanogenesis |
| EP300 | Melanogenesis |
| FZD2 | Melanogenesis |
| GNAI2 | Melanogenesis |
| GNAS | Melanogenesis |
| HRAS | Melanogenesis |
| KIT | Melanogenesis |
| KITLG | Melanogenesis |
| MITF | Melanogenesis |
| PLCB2 | Melanogenesis |
| PLCB3 | Melanogenesis |
| PLCB4 | Melanogenesis |
| PRKACA | Melanogenesis |
| PRKACB | Melanogenesis |
| PRKACG | Melanogenesis |
| PRKCB | Melanogenesis |
| MAP2K2 | Melanogenesis |
| RAF1 | Melanogenesis |
| TCF7L2 | Melanogenesis |
| TYR | Melanogenesis |
| TYRP1 | Melanogenesis |
| WNT2 | Melanogenesis |
| WNT5A | Melanogenesis |
| FZD3 | Melanogenesis |
| FZD1 | Melanogenesis |
| PLCB1 | Melanogenesis |
| LEF1 | Melanogenesis |
| CREB3L2 | Melanogenesis |
| WNT10A | Melanogenesis |
| ACAA1 | Metabolic pathways |
| ACACA | Metabolic pathways |
| ACACB | Metabolic pathways |
| ACADL | Metabolic pathways |
| ACADVL | Metabolic pathways |
| ACAT1 | Metabolic pathways |
| ACLY | Metabolic pathways |
| ACO1 | Metabolic pathways |
| ACO2 | Metabolic pathways |
| ACOX1 | Metabolic pathways |
| ADH1A | Metabolic pathways |
| ADH1B | Metabolic pathways |
| ADH4 | Metabolic pathways |
| ADSL | Metabolic pathways |
| AGXT | Metabolic pathways |
| AHCY | Metabolic pathways |
| ALAS1 | Metabolic pathways |
| ALDH2 | Metabolic pathways |
| ALDH1A3 | Metabolic pathways |
| ALDH3A2 | Metabolic pathways |
| ALDOA | Metabolic pathways |
| ALDOB | Metabolic pathways |
| ALOX5 | Metabolic pathways |
| ALPP | Metabolic pathways |
| ALPPL2 | Metabolic pathways |
| AMD1 | Metabolic pathways |
| AMPD1 | Metabolic pathways |
| AMPD3 | Metabolic pathways |
| AMT | Metabolic pathways |
| AMY2A | Metabolic pathways |
| ANPEP | Metabolic pathways |
| AOX1 | Metabolic pathways |
| APRT | Metabolic pathways |
| ATP5G1 | Metabolic pathways |
| ATP5G2 | Metabolic pathways |
| ATP6V1A | Metabolic pathways |
| ATP6V1B1 | Metabolic pathways |
| ATP5O | Metabolic pathways |
| BCAT1 | Metabolic pathways |
| BCKDHA | Metabolic pathways |
| CAD | Metabolic pathways |
| CBS | Metabolic pathways |
| CCBL1 | Metabolic pathways |
| CDO1 | Metabolic pathways |
| CDS1 | Metabolic pathways |
| CES1 | Metabolic pathways |
| CKMT1B | Metabolic pathways |
| COX6C | Metabolic pathways |
| COX11 | Metabolic pathways |
| COX15 | Metabolic pathways |
| CPS1 | Metabolic pathways |
| CYP2A7 | Metabolic pathways |
| CYP2B6 | Metabolic pathways |
| CYP2C19 | Metabolic pathways |
| CYP2C8 | Metabolic pathways |
| CYP2C9 | Metabolic pathways |
| CYP2C18 | Metabolic pathways |
| CYP2E1 | Metabolic pathways |
| CYP3A5 | Metabolic pathways |
| CYP8B1 | Metabolic pathways |
| DGKA | Metabolic pathways |
| DGKB | Metabolic pathways |
| DGKG | Metabolic pathways |
| DAO | Metabolic pathways |
| DBH | Metabolic pathways |
| DCT | Metabolic pathways |
| DDC | Metabolic pathways |
| DGUOK | Metabolic pathways |
| DHCR7 | Metabolic pathways |
| DHCR24 | Metabolic pathways |
| SARDH | Metabolic pathways |
| DNMT1 | Metabolic pathways |
| DNMT3A | Metabolic pathways |
| EHHADH | Metabolic pathways |
| EPHX2 | Metabolic pathways |
| EXT1 | Metabolic pathways |
| EXTL1 | Metabolic pathways |
| ACSL1 | Metabolic pathways |
| FASN | Metabolic pathways |
| FBP1 | Metabolic pathways |
| FH | Metabolic pathways |
| G6PC | Metabolic pathways |
| GALE | Metabolic pathways |
| B4GALNT1 | Metabolic pathways |
| GALK2 | Metabolic pathways |
| GALNS | Metabolic pathways |
| GALNT1 | Metabolic pathways |
| GALT | Metabolic pathways |
| GAMT | Metabolic pathways |
| GART | Metabolic pathways |
| GBE1 | Metabolic pathways |
| GCK | Metabolic pathways |
| GCNT1 | Metabolic pathways |
| GCNT2 | Metabolic pathways |
| GFPT1 | Metabolic pathways |
| GK | Metabolic pathways |
| GCLC | Metabolic pathways |
| GCLM | Metabolic pathways |
| GLUL | Metabolic pathways |
| GNS | Metabolic pathways |
| GOT1 | Metabolic pathways |
| GPI | Metabolic pathways |
| HADHA | Metabolic pathways |
| HADHB | Metabolic pathways |
| HEXB | Metabolic pathways |
| HGD | Metabolic pathways |
| HK1 | Metabolic pathways |
| HK2 | Metabolic pathways |
| HLCS | Metabolic pathways |
| HMBS | Metabolic pathways |
| HMGCL | Metabolic pathways |
| HMGCR | Metabolic pathways |
| HMGCS1 | Metabolic pathways |
| HMGCS2 | Metabolic pathways |
| HPD | Metabolic pathways |
| HPRT1 | Metabolic pathways |
| HSD3B1 | Metabolic pathways |
| IDH1 | Metabolic pathways |
| IDH3A | Metabolic pathways |
| IMPA2 | Metabolic pathways |
| IMPDH1 | Metabolic pathways |
| IDO1 | Metabolic pathways |
| INPP4A | Metabolic pathways |
| INPP5A | Metabolic pathways |
| INPP5B | Metabolic pathways |
| ITPKB | Metabolic pathways |
| KHK | Metabolic pathways |
| LCT | Metabolic pathways |
| LIPC | Metabolic pathways |
| LSS | Metabolic pathways |
| LTA4H | Metabolic pathways |
| CYP4F3 | Metabolic pathways |
| MAN2A1 | Metabolic pathways |
| MAOA | Metabolic pathways |
| MAOB | Metabolic pathways |
| MDH1 | Metabolic pathways |
| ME1 | Metabolic pathways |
| MGAT5 | Metabolic pathways |
| MOCS1 | Metabolic pathways |
| MPI | Metabolic pathways |
| MTHFD1 | Metabolic pathways |
| MTM1 | Metabolic pathways |
| MUT | Metabolic pathways |
| MVK | Metabolic pathways |
| NDUFS1 | Metabolic pathways |
| NDUFS2 | Metabolic pathways |
| NDUFS3 | Metabolic pathways |
| NNMT | Metabolic pathways |
| NOS1 | Metabolic pathways |
| NOS2 | Metabolic pathways |
| NOS3 | Metabolic pathways |
| ODC1 | Metabolic pathways |
| P4HA1 | Metabolic pathways |
| PAFAH1B1 | Metabolic pathways |
| PAFAH1B3 | Metabolic pathways |
| PAH | Metabolic pathways |
| PC | Metabolic pathways |
| PCCA | Metabolic pathways |
| PCCB | Metabolic pathways |
| PCK1 | Metabolic pathways |
| PDHA2 | Metabolic pathways |
| ENPP3 | Metabolic pathways |
| PFAS | Metabolic pathways |
| PFKL | Metabolic pathways |
| PFKM | Metabolic pathways |
| PFKP | Metabolic pathways |
| PIGC | Metabolic pathways |
| PIK3C2A | Metabolic pathways |
| PIK3C2G | Metabolic pathways |
| PIK3C3 | Metabolic pathways |
| PI4KA | Metabolic pathways |
| PI4KB | Metabolic pathways |
| PKLR | Metabolic pathways |
| PLA2G4A | Metabolic pathways |
| PLCB2 | Metabolic pathways |
| PLCB3 | Metabolic pathways |
| PLCB4 | Metabolic pathways |
| PLCG1 | Metabolic pathways |
| PNLIP | Metabolic pathways |
| PNLIPRP1 | Metabolic pathways |
| POLA1 | Metabolic pathways |
| POLD1 | Metabolic pathways |
| POLD2 | Metabolic pathways |
| POLR2F | Metabolic pathways |
| POLR2L | Metabolic pathways |
| PPT1 | Metabolic pathways |
| PRPS2 | Metabolic pathways |
| PTGS1 | Metabolic pathways |
| PTGS2 | Metabolic pathways |
| PYCR1 | Metabolic pathways |
| ALDH18A1 | Metabolic pathways |
| PYGL | Metabolic pathways |
| REV3L | Metabolic pathways |
| RPE | Metabolic pathways |
| RPN1 | Metabolic pathways |
| SCP2 | Metabolic pathways |
| SDHA | Metabolic pathways |
| SDHD | Metabolic pathways |
| SHMT2 | Metabolic pathways |
| SI | Metabolic pathways |
| ST6GAL1 | Metabolic pathways |
| ST3GAL3 | Metabolic pathways |
| ST8SIA1 | Metabolic pathways |
| SMS | Metabolic pathways |
| SPAM1 | Metabolic pathways |
| SQLE | Metabolic pathways |
| AKR1D1 | Metabolic pathways |
| TAT | Metabolic pathways |
| TK1 | Metabolic pathways |
| TM7SF2 | Metabolic pathways |
| TPH1 | Metabolic pathways |
| TPO | Metabolic pathways |
| TYMS | Metabolic pathways |
| TYR | Metabolic pathways |
| TYRP1 | Metabolic pathways |
| UGCG | Metabolic pathways |
| UGT2B10 | Metabolic pathways |
| UGT8 | Metabolic pathways |
| UMPS | Metabolic pathways |
| XDH | Metabolic pathways |
| ALDH5A1 | Metabolic pathways |
| HSD17B8 | Metabolic pathways |
| PLA2G7 | Metabolic pathways |
| TUSC3 | Metabolic pathways |
| TKTL1 | Metabolic pathways |
| ACOX3 | Metabolic pathways |
| PIP5K1A | Metabolic pathways |
| PLA2G6 | Metabolic pathways |
| NDST2 | Metabolic pathways |
| DGKZ | Metabolic pathways |
| DGKD | Metabolic pathways |
| CYP4F2 | Metabolic pathways |
| AGPS | Metabolic pathways |
| PLA2G4C | Metabolic pathways |
| HYAL2 | Metabolic pathways |
| B3GALNT1 | Metabolic pathways |
| B3GALT2 | Metabolic pathways |
| FPGT | Metabolic pathways |
| DPM1 | Metabolic pathways |
| INPP4B | Metabolic pathways |
| ALDH1A2 | Metabolic pathways |
| SYNJ1 | Metabolic pathways |
| SYNJ2 | Metabolic pathways |
| SGPL1 | Metabolic pathways |
| KYNU | Metabolic pathways |
| MGAM | Metabolic pathways |
| DGKI | Metabolic pathways |
| SLC33A1 | Metabolic pathways |
| ATP6V1F | Metabolic pathways |
| GGPS1 | Metabolic pathways |
| PGS1 | Metabolic pathways |
| POLR1C | Metabolic pathways |
| LPIN2 | Metabolic pathways |
| XYLB | Metabolic pathways |
| AASS | Metabolic pathways |
| ALG3 | Metabolic pathways |
| B3GALT5 | Metabolic pathways |
| CDIPT | Metabolic pathways |
| ACAA2 | Metabolic pathways |
| MTHFS | Metabolic pathways |
| PAICS | Metabolic pathways |
| POLR3C | Metabolic pathways |
| PMVK | Metabolic pathways |
| FUT9 | Metabolic pathways |
| AHCYL1 | Metabolic pathways |
| C5orf4 | Metabolic pathways |
| HPSE | Metabolic pathways |
| ME3 | Metabolic pathways |
| MAN1A2 | Metabolic pathways |
| GALNT6 | Metabolic pathways |
| GALNT5 | Metabolic pathways |
| MAN1B1 | Metabolic pathways |
| CYP4F8 | Metabolic pathways |
| MGAT4A | Metabolic pathways |
| MGLL | Metabolic pathways |
| LPIN1 | Metabolic pathways |
| ACSBG1 | Metabolic pathways |
| PLCB1 | Metabolic pathways |
| ACSL6 | Metabolic pathways |
| AHCYL2 | Metabolic pathways |
| PIP5K1C | Metabolic pathways |
| NNT | Metabolic pathways |
| ATP6V0A2 | Metabolic pathways |
| AMACR | Metabolic pathways |
| PLD3 | Metabolic pathways |
| PISD | Metabolic pathways |
| PGLS | Metabolic pathways |
| MGAT4C | Metabolic pathways |
| POLR1A | Metabolic pathways |
| DAK | Metabolic pathways |
| GLCE | Metabolic pathways |
| PHGDH | Metabolic pathways |
| AK5 | Metabolic pathways |
| GALNT8 | Metabolic pathways |
| GBGT1 | Metabolic pathways |
| ACAD8 | Metabolic pathways |
| UQCRQ | Metabolic pathways |
| GLS2 | Metabolic pathways |
| COQ2 | Metabolic pathways |
| DSE | Metabolic pathways |
| DMGDH | Metabolic pathways |
| PLA2G3 | Metabolic pathways |
| ATP6V0A4 | Metabolic pathways |
| LAP3 | Metabolic pathways |
| MECR | Metabolic pathways |
| RDH11 | Metabolic pathways |
| HSD17B12 | Metabolic pathways |
| AADAT | Metabolic pathways |
| PLCE1 | Metabolic pathways |
| PIPOX | Metabolic pathways |
| GCNT4 | Metabolic pathways |
| PIGT | Metabolic pathways |
| GALNT7 | Metabolic pathways |
| BCMO1 | Metabolic pathways |
| CHPF2 | Metabolic pathways |
| UGT1A6 | Metabolic pathways |
| UGT1A1 | Metabolic pathways |
| TRIT1 | Metabolic pathways |
| LPCAT2 | Metabolic pathways |
| PANK4 | Metabolic pathways |
| PGM2 | Metabolic pathways |
| PI4K2B | Metabolic pathways |
| OLAH | Metabolic pathways |
| SPTLC3 | Metabolic pathways |
| RFK | Metabolic pathways |
| ETNK1 | Metabolic pathways |
| AGK | Metabolic pathways |
| CSGALNACT1 | Metabolic pathways |
| CMAS | Metabolic pathways |
| NDUFA12 | Metabolic pathways |
| CTPS2 | Metabolic pathways |
| SPHK2 | Metabolic pathways |
| AGPAT3 | Metabolic pathways |
| AGPAT4 | Metabolic pathways |
| MCCC1 | Metabolic pathways |
| GALNTL1 | Metabolic pathways |
| GPAM | Metabolic pathways |
| CYP4F11 | Metabolic pathways |
| PRODH2 | Metabolic pathways |
| HPSE2 | Metabolic pathways |
| XYLT1 | Metabolic pathways |
| XYLT2 | Metabolic pathways |
| WBSCR17 | Metabolic pathways |
| NDST4 | Metabolic pathways |
| IPPK | Metabolic pathways |
| LPIN3 | Metabolic pathways |
| TRAK2 | Metabolic pathways |
| ALG8 | Metabolic pathways |
| CHPF | Metabolic pathways |
| ACSS3 | Metabolic pathways |
| GALNT14 | Metabolic pathways |
| PPCS | Metabolic pathways |
| ALG13 | Metabolic pathways |
| PANK2 | Metabolic pathways |
| PGAP1 | Metabolic pathways |
| PTGES2 | Metabolic pathways |
| UXS1 | Metabolic pathways |
| HKDC1 | Metabolic pathways |
| ITPKC | Metabolic pathways |
| ACSBG2 | Metabolic pathways |
| ST6GALNAC5 | Metabolic pathways |
| FAHD1 | Metabolic pathways |
| ADPGK | Metabolic pathways |
| POLR3GL | Metabolic pathways |
| NT5C1A | Metabolic pathways |
| ST6GAL2 | Metabolic pathways |
| DGAT2 | Metabolic pathways |
| COX4I2 | Metabolic pathways |
| AGPAT9 | Metabolic pathways |
| ALG10 | Metabolic pathways |
| PIGS | Metabolic pathways |
| GALNT13 | Metabolic pathways |
| GALNTL2 | Metabolic pathways |
| CYP2R1 | Metabolic pathways |
| DEGS2 | Metabolic pathways |
| PIGU | Metabolic pathways |
| CMPK2 | Metabolic pathways |
| MBOAT2 | Metabolic pathways |
| UROC1 | Metabolic pathways |
| GLYCTK | Metabolic pathways |
| HGSNAT | Metabolic pathways |
| UPRT | Metabolic pathways |
| AMDHD1 | Metabolic pathways |
| PLB1 | Metabolic pathways |
| UPP2 | Metabolic pathways |
| DGKH | Metabolic pathways |
| NAGS | Metabolic pathways |
| SGMS2 | Metabolic pathways |
| GALNTL5 | Metabolic pathways |
| IDO2 | Metabolic pathways |
| COX7B2 | Metabolic pathways |
| CERS6 | Metabolic pathways |
| LPCAT4 | Metabolic pathways |
| PLA2G4F | Metabolic pathways |
| SGMS1 | Metabolic pathways |
| P4HA3 | Metabolic pathways |
| PLA2G4D | Metabolic pathways |
| MMAB | Metabolic pathways |
| ENPP7 | Metabolic pathways |
| ACSM4 | Metabolic pathways |
| MOGAT3 | Metabolic pathways |
| MTHFD2L | Metabolic pathways |
| GALNTL6 | Metabolic pathways |
| ADCYAP1R1 | Neuroactive ligand-receptor interaction |
| ADORA2A | Neuroactive ligand-receptor interaction |
| ADORA3 | Neuroactive ligand-receptor interaction |
| ADRA1A | Neuroactive ligand-receptor interaction |
| ADRA2B | Neuroactive ligand-receptor interaction |
| ADRB2 | Neuroactive ligand-receptor interaction |
| CCKAR | Neuroactive ligand-receptor interaction |
| CHRM1 | Neuroactive ligand-receptor interaction |
| CHRM2 | Neuroactive ligand-receptor interaction |
| CHRM3 | Neuroactive ligand-receptor interaction |
| CHRNA1 | Neuroactive ligand-receptor interaction |
| CHRNA4 | Neuroactive ligand-receptor interaction |
| CHRNB4 | Neuroactive ligand-receptor interaction |
| CHRND | Neuroactive ligand-receptor interaction |
| CNR1 | Neuroactive ligand-receptor interaction |
| CNR2 | Neuroactive ligand-receptor interaction |
| CRHR2 | Neuroactive ligand-receptor interaction |
| DRD3 | Neuroactive ligand-receptor interaction |
| S1PR1 | Neuroactive ligand-receptor interaction |
| LPAR1 | Neuroactive ligand-receptor interaction |
| EDNRA | Neuroactive ligand-receptor interaction |
| F2 | Neuroactive ligand-receptor interaction |
| F2R | Neuroactive ligand-receptor interaction |
| FPR2 | Neuroactive ligand-receptor interaction |
| FPR3 | Neuroactive ligand-receptor interaction |
| FSHR | Neuroactive ligand-receptor interaction |
| GABBR1 | Neuroactive ligand-receptor interaction |
| GABRA1 | Neuroactive ligand-receptor interaction |
| GABRA2 | Neuroactive ligand-receptor interaction |
| GABRA5 | Neuroactive ligand-receptor interaction |
| GABRA6 | Neuroactive ligand-receptor interaction |
| GABRB2 | Neuroactive ligand-receptor interaction |
| GABRB3 | Neuroactive ligand-receptor interaction |
| GABRG1 | Neuroactive ligand-receptor interaction |
| GABRG2 | Neuroactive ligand-receptor interaction |
| GABRG3 | Neuroactive ligand-receptor interaction |
| GALR1 | Neuroactive ligand-receptor interaction |
| GH2 | Neuroactive ligand-receptor interaction |
| GLRA1 | Neuroactive ligand-receptor interaction |
| GLRB | Neuroactive ligand-receptor interaction |
| GNRHR | Neuroactive ligand-receptor interaction |
| GRIA1 | Neuroactive ligand-receptor interaction |
| GRIA2 | Neuroactive ligand-receptor interaction |
| GRIA4 | Neuroactive ligand-receptor interaction |
| GRID1 | Neuroactive ligand-receptor interaction |
| GRID2 | Neuroactive ligand-receptor interaction |
| GRIK2 | Neuroactive ligand-receptor interaction |
| GRIK3 | Neuroactive ligand-receptor interaction |
| GRIK4 | Neuroactive ligand-receptor interaction |
| GRIK5 | Neuroactive ligand-receptor interaction |
| GRIN2A | Neuroactive ligand-receptor interaction |
| GRIN2B | Neuroactive ligand-receptor interaction |
| NR3C1 | Neuroactive ligand-receptor interaction |
| GRM1 | Neuroactive ligand-receptor interaction |
| GRM2 | Neuroactive ligand-receptor interaction |
| GRM3 | Neuroactive ligand-receptor interaction |
| GRM4 | Neuroactive ligand-receptor interaction |
| GRM5 | Neuroactive ligand-receptor interaction |
| GRM7 | Neuroactive ligand-receptor interaction |
| GRM8 | Neuroactive ligand-receptor interaction |
| HCRTR1 | Neuroactive ligand-receptor interaction |
| HCRTR2 | Neuroactive ligand-receptor interaction |
| HRH2 | Neuroactive ligand-receptor interaction |
| HTR1A | Neuroactive ligand-receptor interaction |
| HTR2A | Neuroactive ligand-receptor interaction |
| HTR2B | Neuroactive ligand-receptor interaction |
| HTR2C | Neuroactive ligand-receptor interaction |
| HTR7 | Neuroactive ligand-receptor interaction |
| LEPR | Neuroactive ligand-receptor interaction |
| LHCGR | Neuroactive ligand-receptor interaction |
| MC2R | Neuroactive ligand-receptor interaction |
| MC3R | Neuroactive ligand-receptor interaction |
| MC4R | Neuroactive ligand-receptor interaction |
| MC5R | Neuroactive ligand-receptor interaction |
| NPY5R | Neuroactive ligand-receptor interaction |
| OPRM1 | Neuroactive ligand-receptor interaction |
| P2RY6 | Neuroactive ligand-receptor interaction |
| PLG | Neuroactive ligand-receptor interaction |
| PPYR1 | Neuroactive ligand-receptor interaction |
| PRL | Neuroactive ligand-receptor interaction |
| PRSS1 | Neuroactive ligand-receptor interaction |
| PTGER2 | Neuroactive ligand-receptor interaction |
| PTGER4 | Neuroactive ligand-receptor interaction |
| PTGIR | Neuroactive ligand-receptor interaction |
| PTH1R | Neuroactive ligand-receptor interaction |
| SSTR4 | Neuroactive ligand-receptor interaction |
| TACR3 | Neuroactive ligand-receptor interaction |
| TSHR | Neuroactive ligand-receptor interaction |
| TRPV1 | Neuroactive ligand-receptor interaction |
| GLRA3 | Neuroactive ligand-receptor interaction |
| GALR3 | Neuroactive ligand-receptor interaction |
| CHRNA6 | Neuroactive ligand-receptor interaction |
| GABBR2 | Neuroactive ligand-receptor interaction |
| NMUR1 | Neuroactive ligand-receptor interaction |
| CYSLTR1 | Neuroactive ligand-receptor interaction |
| NPFFR2 | Neuroactive ligand-receptor interaction |
| GPR83 | Neuroactive ligand-receptor interaction |
| HRH3 | Neuroactive ligand-receptor interaction |
| P2RX2 | Neuroactive ligand-receptor interaction |
| LPAR3 | Neuroactive ligand-receptor interaction |
| NTSR2 | Neuroactive ligand-receptor interaction |
| P2RY10 | Neuroactive ligand-receptor interaction |
| PARD3 | Neuroactive ligand-receptor interaction |
| NMUR2 | Neuroactive ligand-receptor interaction |
| CHRNA10 | Neuroactive ligand-receptor interaction |
| CYSLTR2 | Neuroactive ligand-receptor interaction |
| RXFP1 | Neuroactive ligand-receptor interaction |
| NPFFR1 | Neuroactive ligand-receptor interaction |
| MCHR2 | Neuroactive ligand-receptor interaction |
| RXFP2 | Neuroactive ligand-receptor interaction |
| TAAR9 | Neuroactive ligand-receptor interaction |
| TAAR1 | Neuroactive ligand-receptor interaction |
| GPR156 | Neuroactive ligand-receptor interaction |
| GABRR3 | Neuroactive ligand-receptor interaction |
| TAAR6 | Neuroactive ligand-receptor interaction |
| ADCY2 | Cholinergic synapse |
| ADCY3 | Cholinergic synapse |
| ADCY6 | Cholinergic synapse |
| ADCY8 | Cholinergic synapse |
| ADCY9 | Cholinergic synapse |
| ATF4 | Cholinergic synapse |
| CACNA1A | Cholinergic synapse |
| CACNA1B | Cholinergic synapse |
| CACNA1C | Cholinergic synapse |
| CACNA1D | Cholinergic synapse |
| CACNA1S | Cholinergic synapse |
| CHRM1 | Cholinergic synapse |
| CHRM2 | Cholinergic synapse |
| CHRM3 | Cholinergic synapse |
| CHRNA4 | Cholinergic synapse |
| CHRNB4 | Cholinergic synapse |
| FYN | Cholinergic synapse |
| GNAI2 | Cholinergic synapse |
| GNB2 | Cholinergic synapse |
| GNG5 | Cholinergic synapse |
| GNG11 | Cholinergic synapse |
| GNGT1 | Cholinergic synapse |
| GNGT2 | Cholinergic synapse |
| HRAS | Cholinergic synapse |
| ITPR1 | Cholinergic synapse |
| ITPR2 | Cholinergic synapse |
| ITPR3 | Cholinergic synapse |
| JAK2 | Cholinergic synapse |
| KCNJ6 | Cholinergic synapse |
| KCNQ2 | Cholinergic synapse |
| KCNQ3 | Cholinergic synapse |
| PIK3CA | Cholinergic synapse |
| PIK3CD | Cholinergic synapse |
| PIK3CG | Cholinergic synapse |
| PIK3R1 | Cholinergic synapse |
| PLCB2 | Cholinergic synapse |
| PLCB3 | Cholinergic synapse |
| PLCB4 | Cholinergic synapse |
| PRKACA | Cholinergic synapse |
| PRKACB | Cholinergic synapse |
| PRKACG | Cholinergic synapse |
| PRKCB | Cholinergic synapse |
| SLC18A3 | Cholinergic synapse |
| CHRNA6 | Cholinergic synapse |
| CREB5 | Cholinergic synapse |
| GNB5 | Cholinergic synapse |
| PLCB1 | Cholinergic synapse |
| KCNQ5 | Cholinergic synapse |
| SLC5A7 | Cholinergic synapse |
| CREB3L2 | Cholinergic synapse |
| ATP1A1 | Protein digestion and absorption |
| ATP1A3 | Protein digestion and absorption |
| ATP1A4 | Protein digestion and absorption |
| COL1A1 | Protein digestion and absorption |
| COL1A2 | Protein digestion and absorption |
| COL2A1 | Protein digestion and absorption |
| COL3A1 | Protein digestion and absorption |
| COL4A1 | Protein digestion and absorption |
| COL4A2 | Protein digestion and absorption |
| COL4A3 | Protein digestion and absorption |
| COL4A4 | Protein digestion and absorption |
| COL4A5 | Protein digestion and absorption |
| COL4A6 | Protein digestion and absorption |
| COL5A1 | Protein digestion and absorption |
| COL5A2 | Protein digestion and absorption |
| COL6A1 | Protein digestion and absorption |
| COL6A2 | Protein digestion and absorption |
| COL6A3 | Protein digestion and absorption |
| COL7A1 | Protein digestion and absorption |
| COL9A2 | Protein digestion and absorption |
| COL11A1 | Protein digestion and absorption |
| COL11A2 | Protein digestion and absorption |
| COL12A1 | Protein digestion and absorption |
| COL13A1 | Protein digestion and absorption |
| COL15A1 | Protein digestion and absorption |
| COL17A1 | Protein digestion and absorption |
| CPA2 | Protein digestion and absorption |
| CPA3 | Protein digestion and absorption |
| CPB2 | Protein digestion and absorption |
| CTRL | Protein digestion and absorption |
| DPP4 | Protein digestion and absorption |
| ELN | Protein digestion and absorption |
| PRCP | Protein digestion and absorption |
| PRSS1 | Protein digestion and absorption |
| SLC3A2 | Protein digestion and absorption |
| SLC8A2 | Protein digestion and absorption |
| SLC8A1 | Protein digestion and absorption |
| SLC9A3 | Protein digestion and absorption |
| XPNPEP2 | Protein digestion and absorption |
| KCNK5 | Protein digestion and absorption |
| SLC7A9 | Protein digestion and absorption |
| ATP1B4 | Protein digestion and absorption |
| COL5A3 | Protein digestion and absorption |
| COL18A1 | Protein digestion and absorption |
| COL21A1 | Protein digestion and absorption |
| COL27A1 | Protein digestion and absorption |
| COL6A6 | Protein digestion and absorption |
| COL22A1 | Protein digestion and absorption |
| COL24A1 | Protein digestion and absorption |
| SLC6A19 | Protein digestion and absorption |
| ARNTL | Dopaminergic synapse |
| ARRB2 | Dopaminergic synapse |
| ATF4 | Dopaminergic synapse |
| CACNA1A | Dopaminergic synapse |
| CACNA1B | Dopaminergic synapse |
| CACNA1C | Dopaminergic synapse |
| CACNA1D | Dopaminergic synapse |
| CALML3 | Dopaminergic synapse |
| ATF2 | Dopaminergic synapse |
| ATF6B | Dopaminergic synapse |
| DDC | Dopaminergic synapse |
| DRD3 | Dopaminergic synapse |
| GNAI2 | Dopaminergic synapse |
| GNAL | Dopaminergic synapse |
| GNAS | Dopaminergic synapse |
| GNB2 | Dopaminergic synapse |
| GNG5 | Dopaminergic synapse |
| GNG11 | Dopaminergic synapse |
| GNGT1 | Dopaminergic synapse |
| GNGT2 | Dopaminergic synapse |
| GRIA1 | Dopaminergic synapse |
| GRIA2 | Dopaminergic synapse |
| GRIA4 | Dopaminergic synapse |
| GRIN2A | Dopaminergic synapse |
| GRIN2B | Dopaminergic synapse |
| ITPR1 | Dopaminergic synapse |
| ITPR2 | Dopaminergic synapse |
| ITPR3 | Dopaminergic synapse |
| KCNJ5 | Dopaminergic synapse |
| KCNJ6 | Dopaminergic synapse |
| KCNJ9 | Dopaminergic synapse |
| KIF5A | Dopaminergic synapse |
| KIF5B | Dopaminergic synapse |
| KIF5C | Dopaminergic synapse |
| MAOA | Dopaminergic synapse |
| MAOB | Dopaminergic synapse |
| PLCB2 | Dopaminergic synapse |
| PLCB3 | Dopaminergic synapse |
| PLCB4 | Dopaminergic synapse |
| PPP2CB | Dopaminergic synapse |
| PPP2R1B | Dopaminergic synapse |
| PPP2R2A | Dopaminergic synapse |
| PPP2R2C | Dopaminergic synapse |
| PPP2R3A | Dopaminergic synapse |
| PPP3CB | Dopaminergic synapse |
| PPP3CC | Dopaminergic synapse |
| PRKACA | Dopaminergic synapse |
| PRKACB | Dopaminergic synapse |
| PRKACG | Dopaminergic synapse |
| PRKCB | Dopaminergic synapse |
| MAPK8 | Dopaminergic synapse |
| MAPK9 | Dopaminergic synapse |
| SCN1A | Dopaminergic synapse |
| SLC6A3 | Dopaminergic synapse |
| SLC18A1 | Dopaminergic synapse |
| CLOCK | Dopaminergic synapse |
| CREB5 | Dopaminergic synapse |
| GNB5 | Dopaminergic synapse |
| PLCB1 | Dopaminergic synapse |
| PPP2R2D | Dopaminergic synapse |
| CREB3L2 | Dopaminergic synapse |
| PPP1R1B | Dopaminergic synapse |
| RHOA | Axon guidance |
| DCC | Axon guidance |
| DPYSL2 | Axon guidance |
| EFNA5 | Axon guidance |
| EFNB2 | Axon guidance |
| EPHA2 | Axon guidance |
| EPHA1 | Axon guidance |
| EPHA3 | Axon guidance |
| EPHA4 | Axon guidance |
| EPHA5 | Axon guidance |
| EPHA7 | Axon guidance |
| EPHB3 | Axon guidance |
| EPHB4 | Axon guidance |
| EPHB6 | Axon guidance |
| FYN | Axon guidance |
| GNAI2 | Axon guidance |
| HRAS | Axon guidance |
| ITGB1 | Axon guidance |
| L1CAM | Axon guidance |
| ABLIM1 | Axon guidance |
| LIMK2 | Axon guidance |
| MET | Axon guidance |
| NFATC1 | Axon guidance |
| NFATC3 | Axon guidance |
| NFATC4 | Axon guidance |
| NTN3 | Axon guidance |
| PAK1 | Axon guidance |
| PAK2 | Axon guidance |
| PAK3 | Axon guidance |
| PLXNA1 | Axon guidance |
| PLXNA2 | Axon guidance |
| PLXNB3 | Axon guidance |
| PPP3CB | Axon guidance |
| PPP3CC | Axon guidance |
| PTK2 | Axon guidance |
| RASA1 | Axon guidance |
| ROBO1 | Axon guidance |
| ROBO2 | Axon guidance |
| ROCK1 | Axon guidance |
| CXCL12 | Axon guidance |
| SLIT1 | Axon guidance |
| SLIT3 | Axon guidance |
| UNC5C | Axon guidance |
| SEMA5A | Axon guidance |
| SLIT2 | Axon guidance |
| ROCK2 | Axon guidance |
| SEMA3E | Axon guidance |
| PLXNC1 | Axon guidance |
| PAK4 | Axon guidance |
| SEMA3A | Axon guidance |
| SEMA6C | Axon guidance |
| SEMA4D | Axon guidance |
| SEMA4B | Axon guidance |
| NFAT5 | Axon guidance |
| NTNG1 | Axon guidance |
| ARHGEF12 | Axon guidance |
| PLXNB2 | Axon guidance |
| NGEF | Axon guidance |
| SEMA4C | Axon guidance |
| PLXNA3 | Axon guidance |
| DPYSL5 | Axon guidance |
| PAK6 | Axon guidance |
| PAK7 | Axon guidance |
| SEMA6A | Axon guidance |
| SEMA4G | Axon guidance |
| ROBO3 | Axon guidance |
| SEMA6D | Axon guidance |
| UNC5D | Axon guidance |
| EPHA6 | Axon guidance |
| CALML3 | Phosphatidylinositol signaling system |
| CDS1 | Phosphatidylinositol signaling system |
| DGKA | Phosphatidylinositol signaling system |
| DGKB | Phosphatidylinositol signaling system |
| DGKG | Phosphatidylinositol signaling system |
| IMPA2 | Phosphatidylinositol signaling system |
| INPP4A | Phosphatidylinositol signaling system |
| INPP5A | Phosphatidylinositol signaling system |
| INPP5B | Phosphatidylinositol signaling system |
| ITPKB | Phosphatidylinositol signaling system |
| ITPR1 | Phosphatidylinositol signaling system |
| ITPR2 | Phosphatidylinositol signaling system |
| ITPR3 | Phosphatidylinositol signaling system |
| MTM1 | Phosphatidylinositol signaling system |
| PIK3C2A | Phosphatidylinositol signaling system |
| PIK3C2G | Phosphatidylinositol signaling system |
| PIK3C3 | Phosphatidylinositol signaling system |
| PIK3CA | Phosphatidylinositol signaling system |
| PIK3CD | Phosphatidylinositol signaling system |
| PIK3CG | Phosphatidylinositol signaling system |
| PIK3R1 | Phosphatidylinositol signaling system |
| PI4KA | Phosphatidylinositol signaling system |
| PI4KB | Phosphatidylinositol signaling system |
| PLCB2 | Phosphatidylinositol signaling system |
| PLCB3 | Phosphatidylinositol signaling system |
| PLCB4 | Phosphatidylinositol signaling system |
| PLCG1 | Phosphatidylinositol signaling system |
| PRKCB | Phosphatidylinositol signaling system |
| PTEN | Phosphatidylinositol signaling system |
| PIP5K1A | Phosphatidylinositol signaling system |
| PIP4K2B | Phosphatidylinositol signaling system |
| DGKZ | Phosphatidylinositol signaling system |
| DGKD | Phosphatidylinositol signaling system |
| INPP4B | Phosphatidylinositol signaling system |
| SYNJ1 | Phosphatidylinositol signaling system |
| SYNJ2 | Phosphatidylinositol signaling system |
| DGKI | Phosphatidylinositol signaling system |
| CDIPT | Phosphatidylinositol signaling system |
| PLCB1 | Phosphatidylinositol signaling system |
| PIP5K1C | Phosphatidylinositol signaling system |
| PLCE1 | Phosphatidylinositol signaling system |
| PI4K2B | Phosphatidylinositol signaling system |
| IPPK | Phosphatidylinositol signaling system |
| ITPKC | Phosphatidylinositol signaling system |
| DGKH | Phosphatidylinositol signaling system |
| PIKFYVE | Phosphatidylinositol signaling system |
| AFP | Hippo signaling pathway |
| APC | Hippo signaling pathway |
| BIRC2 | Hippo signaling pathway |
| BMP6 | Hippo signaling pathway |
| BMP8B | Hippo signaling pathway |
| CSNK1D | Hippo signaling pathway |
| CSNK1E | Hippo signaling pathway |
| CTNNA2 | Hippo signaling pathway |
| CTNNB1 | Hippo signaling pathway |
| DLG1 | Hippo signaling pathway |
| DLG4 | Hippo signaling pathway |
| DVL3 | Hippo signaling pathway |
| FZD2 | Hippo signaling pathway |
| GLI2 | Hippo signaling pathway |
| ITGB2 | Hippo signaling pathway |
| LLGL2 | Hippo signaling pathway |
| LLGL1 | Hippo signaling pathway |
| SMAD1 | Hippo signaling pathway |
| SMAD2 | Hippo signaling pathway |
| SMAD3 | Hippo signaling pathway |
| NF2 | Hippo signaling pathway |
| SERPINE1 | Hippo signaling pathway |
| PPP2CB | Hippo signaling pathway |
| PPP2R1B | Hippo signaling pathway |
| PPP2R2A | Hippo signaling pathway |
| PPP2R2C | Hippo signaling pathway |
| PRKCI | Hippo signaling pathway |
| STK3 | Hippo signaling pathway |
| TCF7L2 | Hippo signaling pathway |
| TEAD4 | Hippo signaling pathway |
| TGFBR1 | Hippo signaling pathway |
| TP53BP2 | Hippo signaling pathway |
| WNT2 | Hippo signaling pathway |
| WNT5A | Hippo signaling pathway |
| YWHAB | Hippo signaling pathway |
| YWHAZ | Hippo signaling pathway |
| FZD3 | Hippo signaling pathway |
| AXIN1 | Hippo signaling pathway |
| AXIN2 | Hippo signaling pathway |
| FZD1 | Hippo signaling pathway |
| BTRC | Hippo signaling pathway |
| LATS1 | Hippo signaling pathway |
| INADL | Hippo signaling pathway |
| WWC1 | Hippo signaling pathway |
| CRB1 | Hippo signaling pathway |
| CTNNA3 | Hippo signaling pathway |
| LEF1 | Hippo signaling pathway |
| PPP2R2D | Hippo signaling pathway |
| PARD3 | Hippo signaling pathway |
| FRMD1 | Hippo signaling pathway |
| WNT10A | Hippo signaling pathway |
| AMOT | Hippo signaling pathway |
| CRB2 | Hippo signaling pathway |
| ARAF | Long-term depression |
| BRAF | Long-term depression |
| CACNA1A | Long-term depression |
| GNAI2 | Long-term depression |
| GNAS | Long-term depression |
| GNAZ | Long-term depression |
| GRIA1 | Long-term depression |
| GRIA2 | Long-term depression |
| GRID2 | Long-term depression |
| GRM1 | Long-term depression |
| GUCY1A2 | Long-term depression |
| GUCY1A3 | Long-term depression |
| HRAS | Long-term depression |
| IGF1R | Long-term depression |
| ITPR1 | Long-term depression |
| ITPR2 | Long-term depression |
| ITPR3 | Long-term depression |
| NOS1 | Long-term depression |
| PLA2G4A | Long-term depression |
| PLCB2 | Long-term depression |
| PLCB3 | Long-term depression |
| PLCB4 | Long-term depression |
| PPP2CB | Long-term depression |
| PPP2R1B | Long-term depression |
| PRKCB | Long-term depression |
| PRKG1 | Long-term depression |
| PRKG2 | Long-term depression |
| MAP2K2 | Long-term depression |
| RAF1 | Long-term depression |
| RYR1 | Long-term depression |
| PLA2G4C | Long-term depression |
| PLCB1 | Long-term depression |
| PLA2G4F | Long-term depression |
| PLA2G4D | Long-term depression |
| APC | Regulation of actin cytoskeleton |
| ARAF | Regulation of actin cytoskeleton |
| RHOA | Regulation of actin cytoskeleton |
| BRAF | Regulation of actin cytoskeleton |
| CHRM1 | Regulation of actin cytoskeleton |
| CHRM2 | Regulation of actin cytoskeleton |
| CHRM3 | Regulation of actin cytoskeleton |
| DIAPH2 | Regulation of actin cytoskeleton |
| DOCK1 | Regulation of actin cytoskeleton |
| EGFR | Regulation of actin cytoskeleton |
| F2 | Regulation of actin cytoskeleton |
| F2R | Regulation of actin cytoskeleton |
| FGF5 | Regulation of actin cytoskeleton |
| FGF12 | Regulation of actin cytoskeleton |
| FGFR2 | Regulation of actin cytoskeleton |
| FN1 | Regulation of actin cytoskeleton |
| ARHGAP35 | Regulation of actin cytoskeleton |
| NCKAP1L | Regulation of actin cytoskeleton |
| HRAS | Regulation of actin cytoskeleton |
| ITGA6 | Regulation of actin cytoskeleton |
| ITGA1 | Regulation of actin cytoskeleton |
| ITGA2 | Regulation of actin cytoskeleton |
| ITGA3 | Regulation of actin cytoskeleton |
| ITGA4 | Regulation of actin cytoskeleton |
| ITGA5 | Regulation of actin cytoskeleton |
| ITGA9 | Regulation of actin cytoskeleton |
| ITGAD | Regulation of actin cytoskeleton |
| ITGAE | Regulation of actin cytoskeleton |
| ITGAM | Regulation of actin cytoskeleton |
| ITGAV | Regulation of actin cytoskeleton |
| ITGAX | Regulation of actin cytoskeleton |
| ITGB1 | Regulation of actin cytoskeleton |
| ITGB2 | Regulation of actin cytoskeleton |
| ITGB4 | Regulation of actin cytoskeleton |
| ITGB5 | Regulation of actin cytoskeleton |
| ITGB6 | Regulation of actin cytoskeleton |
| LIMK2 | Regulation of actin cytoskeleton |
| MYH9 | Regulation of actin cytoskeleton |
| MYH10 | Regulation of actin cytoskeleton |
| MYLK | Regulation of actin cytoskeleton |
| PPP1R12A | Regulation of actin cytoskeleton |
| PAK1 | Regulation of actin cytoskeleton |
| PAK2 | Regulation of actin cytoskeleton |
| PAK3 | Regulation of actin cytoskeleton |
| PDGFRA | Regulation of actin cytoskeleton |
| PFN2 | Regulation of actin cytoskeleton |
| PIK3CA | Regulation of actin cytoskeleton |
| PIK3CD | Regulation of actin cytoskeleton |
| PIK3CG | Regulation of actin cytoskeleton |
| PIK3R1 | Regulation of actin cytoskeleton |
| MAP2K2 | Regulation of actin cytoskeleton |
| PTK2 | Regulation of actin cytoskeleton |
| RAF1 | Regulation of actin cytoskeleton |
| ROCK1 | Regulation of actin cytoskeleton |
| SLC9A1 | Regulation of actin cytoskeleton |
| SOS1 | Regulation of actin cytoskeleton |
| SOS2 | Regulation of actin cytoskeleton |
| TIAM1 | Regulation of actin cytoskeleton |
| VAV1 | Regulation of actin cytoskeleton |
| VAV2 | Regulation of actin cytoskeleton |
| VCL | Regulation of actin cytoskeleton |
| EZR | Regulation of actin cytoskeleton |
| PIP5K1A | Regulation of actin cytoskeleton |
| PIP4K2B | Regulation of actin cytoskeleton |
| ITGA10 | Regulation of actin cytoskeleton |
| ITGA8 | Regulation of actin cytoskeleton |
| FGF17 | Regulation of actin cytoskeleton |
| IQGAP1 | Regulation of actin cytoskeleton |
| ARHGEF1 | Regulation of actin cytoskeleton |
| ARHGEF6 | Regulation of actin cytoskeleton |
| ROCK2 | Regulation of actin cytoskeleton |
| BCAR1 | Regulation of actin cytoskeleton |
| ARPC1B | Regulation of actin cytoskeleton |
| ABI2 | Regulation of actin cytoskeleton |
| PAK4 | Regulation of actin cytoskeleton |
| VAV3 | Regulation of actin cytoskeleton |
| BAIAP2 | Regulation of actin cytoskeleton |
| ARPC1A | Regulation of actin cytoskeleton |
| MYL12A | Regulation of actin cytoskeleton |
| ITGA11 | Regulation of actin cytoskeleton |
| CYFIP1 | Regulation of actin cytoskeleton |
| ARHGEF12 | Regulation of actin cytoskeleton |
| PIP5K1C | Regulation of actin cytoskeleton |
| CYFIP2 | Regulation of actin cytoskeleton |
| PAK6 | Regulation of actin cytoskeleton |
| PAK7 | Regulation of actin cytoskeleton |
| MYH14 | Regulation of actin cytoskeleton |
| PDGFD | Regulation of actin cytoskeleton |
| DIAPH3 | Regulation of actin cytoskeleton |
| PIKFYVE | Regulation of actin cytoskeleton |
| ACTG2 | Vascular smooth muscle contraction |
| ADCY2 | Vascular smooth muscle contraction |
| ADCY3 | Vascular smooth muscle contraction |
| ADCY6 | Vascular smooth muscle contraction |
| ADCY8 | Vascular smooth muscle contraction |
| ADCY9 | Vascular smooth muscle contraction |
| ADORA2A | Vascular smooth muscle contraction |
| ADRA1A | Vascular smooth muscle contraction |
| ARAF | Vascular smooth muscle contraction |
| RHOA | Vascular smooth muscle contraction |
| BRAF | Vascular smooth muscle contraction |
| CACNA1C | Vascular smooth muscle contraction |
| CACNA1D | Vascular smooth muscle contraction |
| CACNA1S | Vascular smooth muscle contraction |
| CALD1 | Vascular smooth muscle contraction |
| CALML3 | Vascular smooth muscle contraction |
| EDNRA | Vascular smooth muscle contraction |
| GNAS | Vascular smooth muscle contraction |
| GUCY1A2 | Vascular smooth muscle contraction |
| GUCY1A3 | Vascular smooth muscle contraction |
| ITPR1 | Vascular smooth muscle contraction |
| ITPR2 | Vascular smooth muscle contraction |
| ITPR3 | Vascular smooth muscle contraction |
| KCNMA1 | Vascular smooth muscle contraction |
| MYH11 | Vascular smooth muscle contraction |
| MYLK | Vascular smooth muscle contraction |
| PPP1R12A | Vascular smooth muscle contraction |
| NPR1 | Vascular smooth muscle contraction |
| PLA2G4A | Vascular smooth muscle contraction |
| PLCB2 | Vascular smooth muscle contraction |
| PLCB3 | Vascular smooth muscle contraction |
| PLCB4 | Vascular smooth muscle contraction |
| PRKACA | Vascular smooth muscle contraction |
| PRKACB | Vascular smooth muscle contraction |
| PRKACG | Vascular smooth muscle contraction |
| PRKCB | Vascular smooth muscle contraction |
| PRKCH | Vascular smooth muscle contraction |
| PRKCQ | Vascular smooth muscle contraction |
| PRKG1 | Vascular smooth muscle contraction |
| MAP2K2 | Vascular smooth muscle contraction |
| PTGIR | Vascular smooth muscle contraction |
| RAF1 | Vascular smooth muscle contraction |
| ROCK1 | Vascular smooth muscle contraction |
| PLA2G6 | Vascular smooth muscle contraction |
| PLA2G4C | Vascular smooth muscle contraction |
| ARHGEF1 | Vascular smooth muscle contraction |
| ROCK2 | Vascular smooth muscle contraction |
| KCNMB2 | Vascular smooth muscle contraction |
| PLCB1 | Vascular smooth muscle contraction |
| ARHGEF12 | Vascular smooth muscle contraction |
| PLA2G3 | Vascular smooth muscle contraction |
| KCNU1 | Vascular smooth muscle contraction |
| PLA2G4F | Vascular smooth muscle contraction |
| PLA2G4D | Vascular smooth muscle contraction |
| ALOX5 | Serotonergic synapse |
| APP | Serotonergic synapse |
| ARAF | Serotonergic synapse |
| BRAF | Serotonergic synapse |
| CACNA1A | Serotonergic synapse |
| CACNA1B | Serotonergic synapse |
| CACNA1C | Serotonergic synapse |
| CACNA1D | Serotonergic synapse |
| CACNA1S | Serotonergic synapse |
| CYP2C19 | Serotonergic synapse |
| CYP2C8 | Serotonergic synapse |
| CYP2C9 | Serotonergic synapse |
| CYP2C18 | Serotonergic synapse |
| DDC | Serotonergic synapse |
| DUSP1 | Serotonergic synapse |
| GABRB2 | Serotonergic synapse |
| GABRB3 | Serotonergic synapse |
| GNAI2 | Serotonergic synapse |
| GNAS | Serotonergic synapse |
| GNB2 | Serotonergic synapse |
| GNG5 | Serotonergic synapse |
| GNG11 | Serotonergic synapse |
| GNGT1 | Serotonergic synapse |
| GNGT2 | Serotonergic synapse |
| HRAS | Serotonergic synapse |
| HTR1A | Serotonergic synapse |
| HTR2A | Serotonergic synapse |
| HTR2B | Serotonergic synapse |
| HTR2C | Serotonergic synapse |
| HTR3A | Serotonergic synapse |
| HTR7 | Serotonergic synapse |
| ITPR1 | Serotonergic synapse |
| ITPR2 | Serotonergic synapse |
| ITPR3 | Serotonergic synapse |
| KCND2 | Serotonergic synapse |
| KCNJ5 | Serotonergic synapse |
| KCNJ6 | Serotonergic synapse |
| KCNJ9 | Serotonergic synapse |
| KCNN2 | Serotonergic synapse |
| MAOA | Serotonergic synapse |
| MAOB | Serotonergic synapse |
| PLA2G4A | Serotonergic synapse |
| PLCB2 | Serotonergic synapse |
| PLCB3 | Serotonergic synapse |
| PLCB4 | Serotonergic synapse |
| PRKACA | Serotonergic synapse |
| PRKACB | Serotonergic synapse |
| PRKACG | Serotonergic synapse |
| PRKCB | Serotonergic synapse |
| PTGS1 | Serotonergic synapse |
| PTGS2 | Serotonergic synapse |
| RAF1 | Serotonergic synapse |
| SLC18A1 | Serotonergic synapse |
| TPH1 | Serotonergic synapse |
| TRPC1 | Serotonergic synapse |
| PLA2G4C | Serotonergic synapse |
| RAPGEF3 | Serotonergic synapse |
| GNB5 | Serotonergic synapse |
| PLCB1 | Serotonergic synapse |
| HTR3C | Serotonergic synapse |
| PLA2G4F | Serotonergic synapse |
| PLA2G4D | Serotonergic synapse |
| HTR3E | Serotonergic synapse |
| APOA1 | Vitamin digestion and absorption |
| APOA4 | Vitamin digestion and absorption |
| APOB | Vitamin digestion and absorption |
| FOLH1 | Vitamin digestion and absorption |
| GIF | Vitamin digestion and absorption |
| ABCC1 | Vitamin digestion and absorption |
| PNLIP | Vitamin digestion and absorption |
| CUBN | Vitamin digestion and absorption |
| SLC23A1 | Vitamin digestion and absorption |
| SLC19A2 | Vitamin digestion and absorption |
| MMACHC | Vitamin digestion and absorption |
| LMBRD1 | Vitamin digestion and absorption |
| PLB1 | Vitamin digestion and absorption |
| ADRB2 | Endocytosis |
| ADRBK1 | Endocytosis |
| AP2A1 | Endocytosis |
| AP2B1 | Endocytosis |
| RHOA | Endocytosis |
| ARRB2 | Endocytosis |
| CBL | Endocytosis |
| CBLB | Endocytosis |
| CLTC | Endocytosis |
| CSF1R | Endocytosis |
| DAB2 | Endocytosis |
| DNM1 | Endocytosis |
| EGFR | Endocytosis |
| EPS15 | Endocytosis |
| ERBB3 | Endocytosis |
| ERBB4 | Endocytosis |
| F2R | Endocytosis |
| FGFR2 | Endocytosis |
| FLT1 | Endocytosis |
| HLA-A | Endocytosis |
| HLA-E | Endocytosis |
| HRAS | Endocytosis |
| HSPA8 | Endocytosis |
| IGF1R | Endocytosis |
| IL2RB | Endocytosis |
| CXCR1 | Endocytosis |
| KDR | Endocytosis |
| KIT | Endocytosis |
| LDLR | Endocytosis |
| SMAD2 | Endocytosis |
| SMAD3 | Endocytosis |
| MDM2 | Endocytosis |
| MET | Endocytosis |
| NEDD4 | Endocytosis |
| NTRK1 | Endocytosis |
| PDGFRA | Endocytosis |
| PRKCI | Endocytosis |
| PSD | Endocytosis |
| RAB5B | Endocytosis |
| RET | Endocytosis |
| TGFBR1 | Endocytosis |
| TRAF6 | Endocytosis |
| TSG101 | Endocytosis |
| STAM | Endocytosis |
| PIP5K1A | Endocytosis |
| ZFYVE9 | Endocytosis |
| VPS4B | Endocytosis |
| ACAP1 | Endocytosis |
| ZFYVE16 | Endocytosis |
| GIT2 | Endocytosis |
| DNAJC6 | Endocytosis |
| PDCD6IP | Endocytosis |
| STAM2 | Endocytosis |
| STAMBP | Endocytosis |
| RAB31 | Endocytosis |
| WWP1 | Endocytosis |
| SNF8 | Endocytosis |
| VPS45 | Endocytosis |
| NEDD4L | Endocytosis |
| PSD3 | Endocytosis |
| PIP5K1C | Endocytosis |
| ARFGAP3 | Endocytosis |
| EPN1 | Endocytosis |
| SH3KBP1 | Endocytosis |
| EHD3 | Endocytosis |
| ASAP1 | Endocytosis |
| SH3GLB1 | Endocytosis |
| PARD3 | Endocytosis |
| SH3GLB2 | Endocytosis |
| SMAP1 | Endocytosis |
| ZFYVE20 | Endocytosis |
| ARAP3 | Endocytosis |
| SMAP2 | Endocytosis |
| PSD2 | Endocytosis |
| ARFGAP2 | Endocytosis |
| ARAP2 | Endocytosis |
| AGAP2 | Endocytosis |
| AGAP3 | Endocytosis |
| FOLR4 | Endocytosis |
| ADCY2 | GnRH signaling pathway |
| ADCY3 | GnRH signaling pathway |
| ADCY6 | GnRH signaling pathway |
| ADCY8 | GnRH signaling pathway |
| ADCY9 | GnRH signaling pathway |
| ATF4 | GnRH signaling pathway |
| CACNA1C | GnRH signaling pathway |
| CACNA1D | GnRH signaling pathway |
| CACNA1S | GnRH signaling pathway |
| CALML3 | GnRH signaling pathway |
| EGFR | GnRH signaling pathway |
| ELK1 | GnRH signaling pathway |
| PTK2B | GnRH signaling pathway |
| GNAS | GnRH signaling pathway |
| GNRHR | GnRH signaling pathway |
| HRAS | GnRH signaling pathway |
| ITPR1 | GnRH signaling pathway |
| ITPR2 | GnRH signaling pathway |
| ITPR3 | GnRH signaling pathway |
| MAP3K1 | GnRH signaling pathway |
| MAP3K3 | GnRH signaling pathway |
| MAP3K4 | GnRH signaling pathway |
| MMP2 | GnRH signaling pathway |
| MMP14 | GnRH signaling pathway |
| PLA2G4A | GnRH signaling pathway |
| PLCB2 | GnRH signaling pathway |
| PLCB3 | GnRH signaling pathway |
| PLCB4 | GnRH signaling pathway |
| PRKACA | GnRH signaling pathway |
| PRKACB | GnRH signaling pathway |
| PRKACG | GnRH signaling pathway |
| PRKCB | GnRH signaling pathway |
| MAPK7 | GnRH signaling pathway |
| MAPK8 | GnRH signaling pathway |
| MAPK9 | GnRH signaling pathway |
| MAP2K2 | GnRH signaling pathway |
| MAP2K3 | GnRH signaling pathway |
| RAF1 | GnRH signaling pathway |
| SOS1 | GnRH signaling pathway |
| SOS2 | GnRH signaling pathway |
| PLA2G4C | GnRH signaling pathway |
| PLCB1 | GnRH signaling pathway |
| PLA2G4F | GnRH signaling pathway |
| PLA2G4D | GnRH signaling pathway |
| ADCY2 | Salivary secretion |
| ADCY3 | Salivary secretion |
| ADCY6 | Salivary secretion |
| ADCY8 | Salivary secretion |
| ADCY9 | Salivary secretion |
| ADRA1A | Salivary secretion |
| ADRB2 | Salivary secretion |
| ATP1A1 | Salivary secretion |
| ATP1A3 | Salivary secretion |
| ATP1A4 | Salivary secretion |
| ATP2B1 | Salivary secretion |
| ATP2B2 | Salivary secretion |
| ATP2B3 | Salivary secretion |
| ATP2B4 | Salivary secretion |
| CALML3 | Salivary secretion |
| CAMP | Salivary secretion |
| CHRM3 | Salivary secretion |
| CST2 | Salivary secretion |
| CST5 | Salivary secretion |
| DMBT1 | Salivary secretion |
| GNAS | Salivary secretion |
| GUCY1A2 | Salivary secretion |
| GUCY1A3 | Salivary secretion |
| ITPR1 | Salivary secretion |
| ITPR2 | Salivary secretion |
| ITPR3 | Salivary secretion |
| KCNMA1 | Salivary secretion |
| NOS1 | Salivary secretion |
| PLCB2 | Salivary secretion |
| PLCB3 | Salivary secretion |
| PLCB4 | Salivary secretion |
| PRKACA | Salivary secretion |
| PRKACB | Salivary secretion |
| PRKACG | Salivary secretion |
| PRKCB | Salivary secretion |
| PRKG1 | Salivary secretion |
| PRKG2 | Salivary secretion |
| RYR3 | Salivary secretion |
| SLC4A2 | Salivary secretion |
| SLC9A1 | Salivary secretion |
| SLC12A2 | Salivary secretion |
| PLCB1 | Salivary secretion |
| ATP1B4 | Salivary secretion |
| MUC5B | Salivary secretion |
| ADCY2 | Pancreatic secretion |
| ADCY3 | Pancreatic secretion |
| ADCY6 | Pancreatic secretion |
| ADCY8 | Pancreatic secretion |
| ADCY9 | Pancreatic secretion |
| AMY2A | Pancreatic secretion |
| RHOA | Pancreatic secretion |
| ATP1A1 | Pancreatic secretion |
| ATP1A3 | Pancreatic secretion |
| ATP1A4 | Pancreatic secretion |
| ATP2A1 | Pancreatic secretion |
| ATP2A2 | Pancreatic secretion |
| ATP2A3 | Pancreatic secretion |
| ATP2B1 | Pancreatic secretion |
| ATP2B2 | Pancreatic secretion |
| ATP2B3 | Pancreatic secretion |
| ATP2B4 | Pancreatic secretion |
| CCKAR | Pancreatic secretion |
| CFTR | Pancreatic secretion |
| CHRM3 | Pancreatic secretion |
| CPA2 | Pancreatic secretion |
| CPA3 | Pancreatic secretion |
| CPB2 | Pancreatic secretion |
| CTRL | Pancreatic secretion |
| SLC26A3 | Pancreatic secretion |
| GNAS | Pancreatic secretion |
| ITPR1 | Pancreatic secretion |
| ITPR2 | Pancreatic secretion |
| ITPR3 | Pancreatic secretion |
| KCNMA1 | Pancreatic secretion |
| PLCB2 | Pancreatic secretion |
| PLCB3 | Pancreatic secretion |
| PLCB4 | Pancreatic secretion |
| PNLIP | Pancreatic secretion |
| PNLIPRP1 | Pancreatic secretion |
| PRKCB | Pancreatic secretion |
| PRSS1 | Pancreatic secretion |
| RYR2 | Pancreatic secretion |
| SLC4A2 | Pancreatic secretion |
| SLC9A1 | Pancreatic secretion |
| SLC12A2 | Pancreatic secretion |
| TRPC1 | Pancreatic secretion |
| SLC4A4 | Pancreatic secretion |
| RAB3D | Pancreatic secretion |
| CLCA2 | Pancreatic secretion |
| CLCA4 | Pancreatic secretion |
| PLCB1 | Pancreatic secretion |
| ATP1B4 | Pancreatic secretion |
| PLA2G3 | Pancreatic secretion |
| IMPA2 | Inositol phosphate metabolism |
| INPP4A | Inositol phosphate metabolism |
| INPP5A | Inositol phosphate metabolism |
| INPP5B | Inositol phosphate metabolism |
| ITPKB | Inositol phosphate metabolism |
| MTM1 | Inositol phosphate metabolism |
| PIK3C2A | Inositol phosphate metabolism |
| PIK3C2G | Inositol phosphate metabolism |
| PIK3C3 | Inositol phosphate metabolism |
| PIK3CA | Inositol phosphate metabolism |
| PIK3CD | Inositol phosphate metabolism |
| PIK3CG | Inositol phosphate metabolism |
| PI4KA | Inositol phosphate metabolism |
| PI4KB | Inositol phosphate metabolism |
| PLCB2 | Inositol phosphate metabolism |
| PLCB3 | Inositol phosphate metabolism |
| PLCB4 | Inositol phosphate metabolism |
| PLCG1 | Inositol phosphate metabolism |
| PTEN | Inositol phosphate metabolism |
| PIP5K1A | Inositol phosphate metabolism |
| PIP4K2B | Inositol phosphate metabolism |
| INPP4B | Inositol phosphate metabolism |
| SYNJ1 | Inositol phosphate metabolism |
| SYNJ2 | Inositol phosphate metabolism |
| CDIPT | Inositol phosphate metabolism |
| PLCB1 | Inositol phosphate metabolism |
| PIP5K1C | Inositol phosphate metabolism |
| PLCE1 | Inositol phosphate metabolism |
| PI4K2B | Inositol phosphate metabolism |
| IPPK | Inositol phosphate metabolism |
| ITPKC | Inositol phosphate metabolism |
| PIKFYVE | Inositol phosphate metabolism |
| IPMK | Inositol phosphate metabolism |
| ABCA1 | ABC transporters |
| ABCA2 | ABC transporters |
| ABCA3 | ABC transporters |
| ABCA4 | ABC transporters |
| ABCC6 | ABC transporters |
| CFTR | ABC transporters |
| ABCC2 | ABC transporters |
| ABCC1 | ABC transporters |
| ABCB1 | ABC transporters |
| ABCB4 | ABC transporters |
| ABCD3 | ABC transporters |
| ABCC8 | ABC transporters |
| TAP1 | ABC transporters |
| ABCB11 | ABC transporters |
| ABCC3 | ABC transporters |
| ABCC5 | ABC transporters |
| ABCC9 | ABC transporters |
| ABCC4 | ABC transporters |
| ABCA10 | ABC transporters |
| ABCA8 | ABC transporters |
| ABCB10 | ABC transporters |
| ABCA6 | ABC transporters |
| ABCA5 | ABC transporters |
| ABCA12 | ABC transporters |
| ABCG5 | ABC transporters |
| ABCC11 | ABC transporters |
| ABCC12 | ABC transporters |
| ABCA13 | ABC transporters |
| ABCB5 | ABC transporters |
| ADCY2 | Progesterone-mediated oocyte maturation |
| ADCY3 | Progesterone-mediated oocyte maturation |
| ADCY6 | Progesterone-mediated oocyte maturation |
| ADCY8 | Progesterone-mediated oocyte maturation |
| ADCY9 | Progesterone-mediated oocyte maturation |
| ARAF | Progesterone-mediated oocyte maturation |
| BRAF | Progesterone-mediated oocyte maturation |
| BUB1 | Progesterone-mediated oocyte maturation |
| CCNB1 | Progesterone-mediated oocyte maturation |
| CDC25C | Progesterone-mediated oocyte maturation |
| CDC27 | Progesterone-mediated oocyte maturation |
| GNAI2 | Progesterone-mediated oocyte maturation |
| HSP90AA1 | Progesterone-mediated oocyte maturation |
| HSP90AB1 | Progesterone-mediated oocyte maturation |
| IGF1R | Progesterone-mediated oocyte maturation |
| PDE3A | Progesterone-mediated oocyte maturation |
| PDE3B | Progesterone-mediated oocyte maturation |
| PGR | Progesterone-mediated oocyte maturation |
| PIK3CA | Progesterone-mediated oocyte maturation |
| PIK3CD | Progesterone-mediated oocyte maturation |
| PIK3CG | Progesterone-mediated oocyte maturation |
| PIK3R1 | Progesterone-mediated oocyte maturation |
| PRKACA | Progesterone-mediated oocyte maturation |
| PRKACB | Progesterone-mediated oocyte maturation |
| PRKACG | Progesterone-mediated oocyte maturation |
| MAPK8 | Progesterone-mediated oocyte maturation |
| MAPK9 | Progesterone-mediated oocyte maturation |
| RAF1 | Progesterone-mediated oocyte maturation |
| RPS6KA3 | Progesterone-mediated oocyte maturation |
| CDC16 | Progesterone-mediated oocyte maturation |
| CCNA1 | Progesterone-mediated oocyte maturation |
| MAD2L2 | Progesterone-mediated oocyte maturation |
| ANAPC2 | Progesterone-mediated oocyte maturation |
| FZR1 | Progesterone-mediated oocyte maturation |
| ANAPC7 | Progesterone-mediated oocyte maturation |
| CPEB1 | Progesterone-mediated oocyte maturation |
| CCNB3 | Progesterone-mediated oocyte maturation |
| SPDYC | Progesterone-mediated oocyte maturation |
| CD2 | Cell adhesion molecules (CAMs) |
| CD6 | Cell adhesion molecules (CAMs) |
| CD22 | Cell adhesion molecules (CAMs) |
| CD40 | Cell adhesion molecules (CAMs) |
| CDH2 | Cell adhesion molecules (CAMs) |
| CDH3 | Cell adhesion molecules (CAMs) |
| CDH4 | Cell adhesion molecules (CAMs) |
| CDH5 | Cell adhesion molecules (CAMs) |
| CNTN1 | Cell adhesion molecules (CAMs) |
| VCAN | Cell adhesion molecules (CAMs) |
| GLG1 | Cell adhesion molecules (CAMs) |
| HLA-A | Cell adhesion molecules (CAMs) |
| HLA-E | Cell adhesion molecules (CAMs) |
| ITGA6 | Cell adhesion molecules (CAMs) |
| ITGA4 | Cell adhesion molecules (CAMs) |
| ITGA9 | Cell adhesion molecules (CAMs) |
| ITGAM | Cell adhesion molecules (CAMs) |
| ITGAV | Cell adhesion molecules (CAMs) |
| ITGB1 | Cell adhesion molecules (CAMs) |
| ITGB2 | Cell adhesion molecules (CAMs) |
| L1CAM | Cell adhesion molecules (CAMs) |
| NCAM1 | Cell adhesion molecules (CAMs) |
| NCAM2 | Cell adhesion molecules (CAMs) |
| NEO1 | Cell adhesion molecules (CAMs) |
| NRCAM | Cell adhesion molecules (CAMs) |
| CLDN11 | Cell adhesion molecules (CAMs) |
| PTPRC | Cell adhesion molecules (CAMs) |
| PTPRF | Cell adhesion molecules (CAMs) |
| PTPRM | Cell adhesion molecules (CAMs) |
| PVRL1 | Cell adhesion molecules (CAMs) |
| SELE | Cell adhesion molecules (CAMs) |
| SELL | Cell adhesion molecules (CAMs) |
| SELP | Cell adhesion molecules (CAMs) |
| SIGLEC1 | Cell adhesion molecules (CAMs) |
| CNTN2 | Cell adhesion molecules (CAMs) |
| CLDN5 | Cell adhesion molecules (CAMs) |
| VCAM1 | Cell adhesion molecules (CAMs) |
| ITGA8 | Cell adhesion molecules (CAMs) |
| NRXN3 | Cell adhesion molecules (CAMs) |
| NRXN1 | Cell adhesion molecules (CAMs) |
| NRXN2 | Cell adhesion molecules (CAMs) |
| NTNG1 | Cell adhesion molecules (CAMs) |
| NLGN1 | Cell adhesion molecules (CAMs) |
| NFASC | Cell adhesion molecules (CAMs) |
| CLDN14 | Cell adhesion molecules (CAMs) |
| CADM1 | Cell adhesion molecules (CAMs) |
| CNTNAP2 | Cell adhesion molecules (CAMs) |
| CD274 | Cell adhesion molecules (CAMs) |
| CLDN20 | Cell adhesion molecules (CAMs) |
| CLDN18 | Cell adhesion molecules (CAMs) |
| CLDN22 | Cell adhesion molecules (CAMs) |
| CADM3 | Cell adhesion molecules (CAMs) |
| JAM2 | Cell adhesion molecules (CAMs) |
| VTCN1 | Cell adhesion molecules (CAMs) |
| PDCD1LG2 | Cell adhesion molecules (CAMs) |
| ESAM | Cell adhesion molecules (CAMs) |
| LRRC4B | Cell adhesion molecules (CAMs) |
| NEGR1 | Cell adhesion molecules (CAMs) |
| OCLN | Cell adhesion molecules (CAMs) |
| ADCY2 | Gap junction |
| ADCY3 | Gap junction |
| ADCY6 | Gap junction |
| ADCY8 | Gap junction |
| ADCY9 | Gap junction |
| CSNK1D | Gap junction |
| LPAR1 | Gap junction |
| EGFR | Gap junction |
| GJA1 | Gap junction |
| GNAI2 | Gap junction |
| GNAS | Gap junction |
| GRM1 | Gap junction |
| GRM5 | Gap junction |
| GUCY1A2 | Gap junction |
| GUCY1A3 | Gap junction |
| HRAS | Gap junction |
| HTR2A | Gap junction |
| HTR2B | Gap junction |
| HTR2C | Gap junction |
| ITPR1 | Gap junction |
| ITPR2 | Gap junction |
| ITPR3 | Gap junction |
| PDGFRA | Gap junction |
| PLCB2 | Gap junction |
| PLCB3 | Gap junction |
| PLCB4 | Gap junction |
| PRKACA | Gap junction |
| PRKACB | Gap junction |
| PRKACG | Gap junction |
| PRKCB | Gap junction |
| PRKG1 | Gap junction |
| PRKG2 | Gap junction |
| MAPK7 | Gap junction |
| MAP2K2 | Gap junction |
| RAF1 | Gap junction |
| SOS1 | Gap junction |
| SOS2 | Gap junction |
| TJP1 | Gap junction |
| TUBA3C | Gap junction |
| TUBB2A | Gap junction |
| PLCB1 | Gap junction |
| GJD2 | Gap junction |
| TUBAL3 | Gap junction |
| PDGFD | Gap junction |
| TUBA1C | Gap junction |
| TUBA3E | Gap junction |
| ADCY2 | GABAergic synapse |
| ADCY3 | GABAergic synapse |
| ADCY6 | GABAergic synapse |
| ADCY8 | GABAergic synapse |
| ADCY9 | GABAergic synapse |
| CACNA1A | GABAergic synapse |
| CACNA1B | GABAergic synapse |
| CACNA1C | GABAergic synapse |
| CACNA1D | GABAergic synapse |
| CACNA1S | GABAergic synapse |
| GABBR1 | GABAergic synapse |
| GABRA1 | GABAergic synapse |
| GABRA2 | GABAergic synapse |
| GABRA5 | GABAergic synapse |
| GABRA6 | GABAergic synapse |
| GABRB2 | GABAergic synapse |
| GABRB3 | GABAergic synapse |
| GABRG1 | GABAergic synapse |
| GABRG2 | GABAergic synapse |
| GABRG3 | GABAergic synapse |
| GLUL | GABAergic synapse |
| GNAI2 | GABAergic synapse |
| GNB2 | GABAergic synapse |
| GNG5 | GABAergic synapse |
| GNG11 | GABAergic synapse |
| GNGT1 | GABAergic synapse |
| GNGT2 | GABAergic synapse |
| KCNJ6 | GABAergic synapse |
| NSF | GABAergic synapse |
| PLCL1 | GABAergic synapse |
| PRKACA | GABAergic synapse |
| PRKACB | GABAergic synapse |
| PRKACG | GABAergic synapse |
| PRKCB | GABAergic synapse |
| SLC6A1 | GABAergic synapse |
| SLC6A13 | GABAergic synapse |
| GABBR2 | GABAergic synapse |
| GPHN | GABAergic synapse |
| GNB5 | GABAergic synapse |
| GLS2 | GABAergic synapse |
| SLC12A5 | GABAergic synapse |
| TRAK2 | GABAergic synapse |
| GABRR3 | GABAergic synapse |
| ADCY2 | Gastric acid secretion |
| ADCY3 | Gastric acid secretion |
| ADCY6 | Gastric acid secretion |
| ADCY8 | Gastric acid secretion |
| ADCY9 | Gastric acid secretion |
| ATP1A1 | Gastric acid secretion |
| ATP1A3 | Gastric acid secretion |
| ATP1A4 | Gastric acid secretion |
| ATP4B | Gastric acid secretion |
| CALML3 | Gastric acid secretion |
| CFTR | Gastric acid secretion |
| CHRM3 | Gastric acid secretion |
| GAST | Gastric acid secretion |
| GNAI2 | Gastric acid secretion |
| GNAS | Gastric acid secretion |
| HRH2 | Gastric acid secretion |
| ITPR1 | Gastric acid secretion |
| ITPR2 | Gastric acid secretion |
| ITPR3 | Gastric acid secretion |
| KCNJ16 | Gastric acid secretion |
| MYLK | Gastric acid secretion |
| PLCB2 | Gastric acid secretion |
| PLCB3 | Gastric acid secretion |
| PLCB4 | Gastric acid secretion |
| PRKACA | Gastric acid secretion |
| PRKACB | Gastric acid secretion |
| PRKACG | Gastric acid secretion |
| PRKCB | Gastric acid secretion |
| SLC4A2 | Gastric acid secretion |
| SLC9A1 | Gastric acid secretion |
| EZR | Gastric acid secretion |
| PLCB1 | Gastric acid secretion |
| ATP1B4 | Gastric acid secretion |
| SLC26A7 | Gastric acid secretion |
| SLC9A4 | Gastric acid secretion |
| ADCY2 | Bile secretion |
| ADCY3 | Bile secretion |
| ADCY6 | Bile secretion |
| ADCY8 | Bile secretion |
| ADCY9 | Bile secretion |
| ATP1A1 | Bile secretion |
| ATP1A3 | Bile secretion |
| ATP1A4 | Bile secretion |
| CFTR | Bile secretion |
| ABCC2 | Bile secretion |
| EPHX1 | Bile secretion |
| GNAS | Bile secretion |
| HMGCR | Bile secretion |
| KCNN2 | Bile secretion |
| LDLR | Bile secretion |
| ABCB1 | Bile secretion |
| ABCB4 | Bile secretion |
| PRKACA | Bile secretion |
| PRKACB | Bile secretion |
| PRKACG | Bile secretion |
| SLC2A1 | Bile secretion |
| SLC4A2 | Bile secretion |
| SLC5A1 | Bile secretion |
| SLC9A1 | Bile secretion |
| SLC9A3 | Bile secretion |
| SLC10A1 | Bile secretion |
| SLC10A2 | Bile secretion |
| ABCB11 | Bile secretion |
| SLC4A4 | Bile secretion |
| ABCC3 | Bile secretion |
| ABCC4 | Bile secretion |
| SLCO1B1 | Bile secretion |
| SLC22A7 | Bile secretion |
| ATP1B4 | Bile secretion |
| SLCO1B3 | Bile secretion |
| NCEH1 | Bile secretion |
| SLC4A5 | Bile secretion |
| ABCG5 | Bile secretion |
| ADCY8 | Long-term potentiation |
| ARAF | Long-term potentiation |
| ATF4 | Long-term potentiation |
| BRAF | Long-term potentiation |
| CACNA1C | Long-term potentiation |
| CALML3 | Long-term potentiation |
| CREBBP | Long-term potentiation |
| EP300 | Long-term potentiation |
| GRIA1 | Long-term potentiation |
| GRIA2 | Long-term potentiation |
| GRIN2A | Long-term potentiation |
| GRIN2B | Long-term potentiation |
| GRM1 | Long-term potentiation |
| GRM5 | Long-term potentiation |
| HRAS | Long-term potentiation |
| ITPR1 | Long-term potentiation |
| ITPR2 | Long-term potentiation |
| ITPR3 | Long-term potentiation |
| PPP1R12A | Long-term potentiation |
| PLCB2 | Long-term potentiation |
| PLCB3 | Long-term potentiation |
| PLCB4 | Long-term potentiation |
| PPP3CB | Long-term potentiation |
| PPP3CC | Long-term potentiation |
| PRKACA | Long-term potentiation |
| PRKACB | Long-term potentiation |
| PRKACG | Long-term potentiation |
| PRKCB | Long-term potentiation |
| MAP2K2 | Long-term potentiation |
| RAF1 | Long-term potentiation |
| RPS6KA3 | Long-term potentiation |
| RAPGEF3 | Long-term potentiation |
| PLCB1 | Long-term potentiation |
| ADCY2 | Chemokine signaling pathway |
| ADCY3 | Chemokine signaling pathway |
| ADCY6 | Chemokine signaling pathway |
| ADCY8 | Chemokine signaling pathway |
| ADCY9 | Chemokine signaling pathway |
| ADRBK1 | Chemokine signaling pathway |
| RHOA | Chemokine signaling pathway |
| ARRB2 | Chemokine signaling pathway |
| BRAF | Chemokine signaling pathway |
| CHUK | Chemokine signaling pathway |
| CCR1 | Chemokine signaling pathway |
| CCR6 | Chemokine signaling pathway |
| CCR7 | Chemokine signaling pathway |
| DOCK2 | Chemokine signaling pathway |
| PTK2B | Chemokine signaling pathway |
| FGR | Chemokine signaling pathway |
| GNAI2 | Chemokine signaling pathway |
| GNB2 | Chemokine signaling pathway |
| GNG5 | Chemokine signaling pathway |
| GNG11 | Chemokine signaling pathway |
| GNGT1 | Chemokine signaling pathway |
| GNGT2 | Chemokine signaling pathway |
| CXCL2 | Chemokine signaling pathway |
| HRAS | Chemokine signaling pathway |
| CXCR1 | Chemokine signaling pathway |
| ITK | Chemokine signaling pathway |
| JAK2 | Chemokine signaling pathway |
| JAK3 | Chemokine signaling pathway |
| CXCL9 | Chemokine signaling pathway |
| NFKB1 | Chemokine signaling pathway |
| NFKBIA | Chemokine signaling pathway |
| PAK1 | Chemokine signaling pathway |
| PF4V1 | Chemokine signaling pathway |
| PIK3CA | Chemokine signaling pathway |
| PIK3CD | Chemokine signaling pathway |
| PIK3CG | Chemokine signaling pathway |
| PIK3R1 | Chemokine signaling pathway |
| PLCB2 | Chemokine signaling pathway |
| PLCB3 | Chemokine signaling pathway |
| PLCB4 | Chemokine signaling pathway |
| PRKACA | Chemokine signaling pathway |
| PRKACB | Chemokine signaling pathway |
| PRKACG | Chemokine signaling pathway |
| PRKCB | Chemokine signaling pathway |
| PTK2 | Chemokine signaling pathway |
| RAF1 | Chemokine signaling pathway |
| ROCK1 | Chemokine signaling pathway |
| CCL5 | Chemokine signaling pathway |
| CCL11 | Chemokine signaling pathway |
| CCL23 | Chemokine signaling pathway |
| CXCL12 | Chemokine signaling pathway |
| SOS1 | Chemokine signaling pathway |
| SOS2 | Chemokine signaling pathway |
| STAT1 | Chemokine signaling pathway |
| STAT2 | Chemokine signaling pathway |
| STAT5B | Chemokine signaling pathway |
| TIAM1 | Chemokine signaling pathway |
| VAV1 | Chemokine signaling pathway |
| VAV2 | Chemokine signaling pathway |
| ROCK2 | Chemokine signaling pathway |
| BCAR1 | Chemokine signaling pathway |
| ELMO1 | Chemokine signaling pathway |
| VAV3 | Chemokine signaling pathway |
| GNB5 | Chemokine signaling pathway |
| CCR9 | Chemokine signaling pathway |
| PLCB1 | Chemokine signaling pathway |
| PARD3 | Chemokine signaling pathway |
| SHC4 | Chemokine signaling pathway |
| CCR2 | Chemokine signaling pathway |
| ACACA | Insulin signaling pathway |
| ACACB | Insulin signaling pathway |
| ARAF | Insulin signaling pathway |
| BRAF | Insulin signaling pathway |
| CALML3 | Insulin signaling pathway |
| CBL | Insulin signaling pathway |
| CBLB | Insulin signaling pathway |
| ELK1 | Insulin signaling pathway |
| FASN | Insulin signaling pathway |
| FBP1 | Insulin signaling pathway |
| FLOT2 | Insulin signaling pathway |
| MTOR | Insulin signaling pathway |
| G6PC | Insulin signaling pathway |
| GCK | Insulin signaling pathway |
| MKNK2 | Insulin signaling pathway |
| GYS2 | Insulin signaling pathway |
| HK1 | Insulin signaling pathway |
| HK2 | Insulin signaling pathway |
| HRAS | Insulin signaling pathway |
| LIPE | Insulin signaling pathway |
| PCK1 | Insulin signaling pathway |
| PDE3A | Insulin signaling pathway |
| PDE3B | Insulin signaling pathway |
| PHKA1 | Insulin signaling pathway |
| PHKB | Insulin signaling pathway |
| PHKG1 | Insulin signaling pathway |
| PIK3CA | Insulin signaling pathway |
| PIK3CD | Insulin signaling pathway |
| PIK3CG | Insulin signaling pathway |
| PIK3R1 | Insulin signaling pathway |
| PKLR | Insulin signaling pathway |
| PPP1R3A | Insulin signaling pathway |
| PPP1R3C | Insulin signaling pathway |
| PRKACA | Insulin signaling pathway |
| PRKACB | Insulin signaling pathway |
| PRKACG | Insulin signaling pathway |
| PRKAR1A | Insulin signaling pathway |
| PRKAR1B | Insulin signaling pathway |
| PRKAR2A | Insulin signaling pathway |
| PRKCI | Insulin signaling pathway |
| MAPK8 | Insulin signaling pathway |
| MAPK9 | Insulin signaling pathway |
| MAP2K2 | Insulin signaling pathway |
| PTPN1 | Insulin signaling pathway |
| PTPRF | Insulin signaling pathway |
| PYGL | Insulin signaling pathway |
| RAF1 | Insulin signaling pathway |
| RPS6KB1 | Insulin signaling pathway |
| RPS6KB2 | Insulin signaling pathway |
| SOS1 | Insulin signaling pathway |
| SOS2 | Insulin signaling pathway |
| SREBF1 | Insulin signaling pathway |
| TSC1 | Insulin signaling pathway |
| TSC2 | Insulin signaling pathway |
| IRS4 | Insulin signaling pathway |
| MKNK1 | Insulin signaling pathway |
| SOCS3 | Insulin signaling pathway |
| TRIP10 | Insulin signaling pathway |
| EIF4E2 | Insulin signaling pathway |
| PPARGC1A | Insulin signaling pathway |
| PRKAG2 | Insulin signaling pathway |
| RPTOR | Insulin signaling pathway |
| HKDC1 | Insulin signaling pathway |
| SOCS4 | Insulin signaling pathway |
| SHC4 | Insulin signaling pathway |
| BIRC2 | Ubiquitin mediated proteolysis |
| BRCA1 | Ubiquitin mediated proteolysis |
| CBL | Ubiquitin mediated proteolysis |
| CBLB | Ubiquitin mediated proteolysis |
| CDC27 | Ubiquitin mediated proteolysis |
| MDM2 | Ubiquitin mediated proteolysis |
| MAP3K1 | Ubiquitin mediated proteolysis |
| NEDD4 | Ubiquitin mediated proteolysis |
| TRAF6 | Ubiquitin mediated proteolysis |
| UBA7 | Ubiquitin mediated proteolysis |
| UBE2B | Ubiquitin mediated proteolysis |
| UBE2E1 | Ubiquitin mediated proteolysis |
| UBE2G1 | Ubiquitin mediated proteolysis |
| UBE2N | Ubiquitin mediated proteolysis |
| CUL5 | Ubiquitin mediated proteolysis |
| CUL4A | Ubiquitin mediated proteolysis |
| CUL3 | Ubiquitin mediated proteolysis |
| CUL1 | Ubiquitin mediated proteolysis |
| CDC16 | Ubiquitin mediated proteolysis |
| HERC2 | Ubiquitin mediated proteolysis |
| HERC1 | Ubiquitin mediated proteolysis |
| BTRC | Ubiquitin mediated proteolysis |
| SOCS3 | Ubiquitin mediated proteolysis |
| TRIP12 | Ubiquitin mediated proteolysis |
| UBE4A | Ubiquitin mediated proteolysis |
| UBE3C | Ubiquitin mediated proteolysis |
| KEAP1 | Ubiquitin mediated proteolysis |
| CUL7 | Ubiquitin mediated proteolysis |
| HUWE1 | Ubiquitin mediated proteolysis |
| UBE4B | Ubiquitin mediated proteolysis |
| UBE2E3 | Ubiquitin mediated proteolysis |
| WWP1 | Ubiquitin mediated proteolysis |
| WWP2 | Ubiquitin mediated proteolysis |
| UBE2C | Ubiquitin mediated proteolysis |
| TRIM32 | Ubiquitin mediated proteolysis |
| MGRN1 | Ubiquitin mediated proteolysis |
| NEDD4L | Ubiquitin mediated proteolysis |
| HERC4 | Ubiquitin mediated proteolysis |
| FBXW8 | Ubiquitin mediated proteolysis |
| FBXO4 | Ubiquitin mediated proteolysis |
| ANAPC2 | Ubiquitin mediated proteolysis |
| FZR1 | Ubiquitin mediated proteolysis |
| UBR5 | Ubiquitin mediated proteolysis |
| ANAPC7 | Ubiquitin mediated proteolysis |
| FANCL | Ubiquitin mediated proteolysis |
| UBA6 | Ubiquitin mediated proteolysis |
| KLHL9 | Ubiquitin mediated proteolysis |
| BIRC6 | Ubiquitin mediated proteolysis |
| UBE3B | Ubiquitin mediated proteolysis |
| KLHL13 | Ubiquitin mediated proteolysis |
| UBE2F | Ubiquitin mediated proteolysis |
| UBE2NL | Ubiquitin mediated proteolysis |
| BLM | Homologous recombination |
| BRCA2 | Homologous recombination |
| MRE11A | Homologous recombination |
| POLD1 | Homologous recombination |
| POLD2 | Homologous recombination |
| RAD51 | Homologous recombination |
| RAD52 | Homologous recombination |
| RPA1 | Homologous recombination |
| TOP3A | Homologous recombination |
| XRCC2 | Homologous recombination |
| RAD54L | Homologous recombination |
| TOP3B | Homologous recombination |
| RAD50 | Homologous recombination |
| RPA4 | Homologous recombination |
| ACVR1 | TGF-beta signaling pathway |
| ACVR2A | TGF-beta signaling pathway |
| RHOA | TGF-beta signaling pathway |
| BMP6 | TGF-beta signaling pathway |
| BMP8B | TGF-beta signaling pathway |
| CREBBP | TGF-beta signaling pathway |
| DCN | TGF-beta signaling pathway |
| EP300 | TGF-beta signaling pathway |
| IFNG | TGF-beta signaling pathway |
| INHBA | TGF-beta signaling pathway |
| INHBC | TGF-beta signaling pathway |
| LTBP1 | TGF-beta signaling pathway |
| SMAD1 | TGF-beta signaling pathway |
| SMAD2 | TGF-beta signaling pathway |
| SMAD3 | TGF-beta signaling pathway |
| SMAD9 | TGF-beta signaling pathway |
| NODAL | TGF-beta signaling pathway |
| PPP2CB | TGF-beta signaling pathway |
| PPP2R1B | TGF-beta signaling pathway |
| RBL1 | TGF-beta signaling pathway |
| ROCK1 | TGF-beta signaling pathway |
| RPS6KB1 | TGF-beta signaling pathway |
| RPS6KB2 | TGF-beta signaling pathway |
| SP1 | TGF-beta signaling pathway |
| TGFBR1 | TGF-beta signaling pathway |
| THBS1 | TGF-beta signaling pathway |
| CUL1 | TGF-beta signaling pathway |
| CHRD | TGF-beta signaling pathway |
| ZFYVE9 | TGF-beta signaling pathway |
| ROCK2 | TGF-beta signaling pathway |
| ZFYVE16 | TGF-beta signaling pathway |
| FST | TGF-beta signaling pathway |
| BAMBI | TGF-beta signaling pathway |
| ACVR1C | TGF-beta signaling pathway |
| ADH1A | Tyrosine metabolism |
| ADH1B | Tyrosine metabolism |
| ADH4 | Tyrosine metabolism |
| ALDH1A3 | Tyrosine metabolism |
| AOX1 | Tyrosine metabolism |
| DBH | Tyrosine metabolism |
| DCT | Tyrosine metabolism |
| DDC | Tyrosine metabolism |
| GOT1 | Tyrosine metabolism |
| HGD | Tyrosine metabolism |
| HPD | Tyrosine metabolism |
| MAOA | Tyrosine metabolism |
| MAOB | Tyrosine metabolism |
| TAT | Tyrosine metabolism |
| TPO | Tyrosine metabolism |
| TYR | Tyrosine metabolism |
| TYRP1 | Tyrosine metabolism |
| HEMK1 | Tyrosine metabolism |
| FAHD1 | Tyrosine metabolism |
| ABL2 | ErbB signaling pathway |
| ARAF | ErbB signaling pathway |
| BRAF | ErbB signaling pathway |
| CBL | ErbB signaling pathway |
| CBLB | ErbB signaling pathway |
| CDKN1A | ErbB signaling pathway |
| EGFR | ErbB signaling pathway |
| ELK1 | ErbB signaling pathway |
| ERBB2 | ErbB signaling pathway |
| ERBB3 | ErbB signaling pathway |
| ERBB4 | ErbB signaling pathway |
| MTOR | ErbB signaling pathway |
| GAB1 | ErbB signaling pathway |
| NRG1 | ErbB signaling pathway |
| HRAS | ErbB signaling pathway |
| PAK1 | ErbB signaling pathway |
| PAK2 | ErbB signaling pathway |
| PAK3 | ErbB signaling pathway |
| PIK3CA | ErbB signaling pathway |
| PIK3CD | ErbB signaling pathway |
| PIK3CG | ErbB signaling pathway |
| PIK3R1 | ErbB signaling pathway |
| PLCG1 | ErbB signaling pathway |
| PRKCB | ErbB signaling pathway |
| MAPK8 | ErbB signaling pathway |
| MAPK9 | ErbB signaling pathway |
| MAP2K2 | ErbB signaling pathway |
| PTK2 | ErbB signaling pathway |
| RAF1 | ErbB signaling pathway |
| RPS6KB1 | ErbB signaling pathway |
| RPS6KB2 | ErbB signaling pathway |
| SOS1 | ErbB signaling pathway |
| SOS2 | ErbB signaling pathway |
| STAT5B | ErbB signaling pathway |
| PAK4 | ErbB signaling pathway |
| NRG3 | ErbB signaling pathway |
| PAK6 | ErbB signaling pathway |
| PAK7 | ErbB signaling pathway |
| SHC4 | ErbB signaling pathway |
| ADCY6 | Taste transduction |
| ADCY8 | Taste transduction |
| CACNA1A | Taste transduction |
| CACNA1B | Taste transduction |
| GNAS | Taste transduction |
| GRM4 | Taste transduction |
| ITPR3 | Taste transduction |
| KCNB1 | Taste transduction |
| PDE1A | Taste transduction |
| PLCB2 | Taste transduction |
| PRKACA | Taste transduction |
| PRKACB | Taste transduction |
| PRKACG | Taste transduction |
| SCNN1A | Taste transduction |
| SCNN1B | Taste transduction |
| TRPM5 | Taste transduction |
| TAS2R3 | Taste transduction |
| TAS2R9 | Taste transduction |
| TAS2R10 | Taste transduction |
| TAS2R5 | Taste transduction |
| TAS1R2 | Taste transduction |
| TAS1R1 | Taste transduction |
| TAS2R43 | Taste transduction |
| TAS2R46 | Taste transduction |
| TAS2R19 | Taste transduction |
| ADCY3 | Vasopressin-regulated water reabsorption |
| ADCY6 | Vasopressin-regulated water reabsorption |
| ADCY9 | Vasopressin-regulated water reabsorption |
| ARHGDIA | Vasopressin-regulated water reabsorption |
| ARHGDIB | Vasopressin-regulated water reabsorption |
| ARHGDIG | Vasopressin-regulated water reabsorption |
| DCTN1 | Vasopressin-regulated water reabsorption |
| DYNC1H1 | Vasopressin-regulated water reabsorption |
| DYNC1I2 | Vasopressin-regulated water reabsorption |
| DYNC1LI2 | Vasopressin-regulated water reabsorption |
| GNAS | Vasopressin-regulated water reabsorption |
| NSF | Vasopressin-regulated water reabsorption |
| PRKACA | Vasopressin-regulated water reabsorption |
| PRKACB | Vasopressin-regulated water reabsorption |
| PRKACG | Vasopressin-regulated water reabsorption |
| RAB5B | Vasopressin-regulated water reabsorption |
| CREB5 | Vasopressin-regulated water reabsorption |
| CREB3L2 | Vasopressin-regulated water reabsorption |
| DYNC2H1 | Vasopressin-regulated water reabsorption |
| ALDOA | HIF-1 signaling pathway |
| ANGPT1 | HIF-1 signaling pathway |
| ANGPT2 | HIF-1 signaling pathway |
| CDKN1A | HIF-1 signaling pathway |
| CREBBP | HIF-1 signaling pathway |
| EGFR | HIF-1 signaling pathway |
| EP300 | HIF-1 signaling pathway |
| ERBB2 | HIF-1 signaling pathway |
| FLT1 | HIF-1 signaling pathway |
| MTOR | HIF-1 signaling pathway |
| MKNK2 | HIF-1 signaling pathway |
| HIF1A | HIF-1 signaling pathway |
| HK1 | HIF-1 signaling pathway |
| HK2 | HIF-1 signaling pathway |
| IFNG | HIF-1 signaling pathway |
| IGF1R | HIF-1 signaling pathway |
| LTBR | HIF-1 signaling pathway |
| NFKB1 | HIF-1 signaling pathway |
| NOS2 | HIF-1 signaling pathway |
| NOS3 | HIF-1 signaling pathway |
| NPPA | HIF-1 signaling pathway |
| SERPINE1 | HIF-1 signaling pathway |
| PDHA2 | HIF-1 signaling pathway |
| PFKFB2 | HIF-1 signaling pathway |
| PFKFB3 | HIF-1 signaling pathway |
| PFKFB4 | HIF-1 signaling pathway |
| PFKL | HIF-1 signaling pathway |
| PIK3CA | HIF-1 signaling pathway |
| PIK3CD | HIF-1 signaling pathway |
| PIK3CG | HIF-1 signaling pathway |
| PIK3R1 | HIF-1 signaling pathway |
| PLCG1 | HIF-1 signaling pathway |
| PRKCB | HIF-1 signaling pathway |
| MAP2K2 | HIF-1 signaling pathway |
| RPS6KB1 | HIF-1 signaling pathway |
| RPS6KB2 | HIF-1 signaling pathway |
| SLC2A1 | HIF-1 signaling pathway |
| TEK | HIF-1 signaling pathway |
| TF | HIF-1 signaling pathway |
| TLR4 | HIF-1 signaling pathway |
| MKNK1 | HIF-1 signaling pathway |
| EIF4E2 | HIF-1 signaling pathway |
| NOX1 | HIF-1 signaling pathway |
| NOX3 | HIF-1 signaling pathway |
| ANGPT4 | HIF-1 signaling pathway |
| HKDC1 | HIF-1 signaling pathway |
| ACAT1 | Lysine degradation |
| ALDH2 | Lysine degradation |
| ALDH3A2 | Lysine degradation |
| EHHADH | Lysine degradation |
| HADHA | Lysine degradation |
| MLL | Lysine degradation |
| MLL2 | Lysine degradation |
| PLOD3 | Lysine degradation |
| MLL4 | Lysine degradation |
| SETDB1 | Lysine degradation |
| AASS | Lysine degradation |
| SETD1B | Lysine degradation |
| GLT25D2 | Lysine degradation |
| SETD2 | Lysine degradation |
| AADAT | Lysine degradation |
| PIPOX | Lysine degradation |
| WHSC1L1 | Lysine degradation |
| TMLHE | Lysine degradation |
| ASH1L | Lysine degradation |
| MLL5 | Lysine degradation |
| MLL3 | Lysine degradation |
| NSD1 | Lysine degradation |
| SETDB2 | Lysine degradation |
| DOT1L | Lysine degradation |
| SETD8 | Lysine degradation |
| ACTC1 | Cardiac muscle contraction |
| ATP1A1 | Cardiac muscle contraction |
| ATP1A3 | Cardiac muscle contraction |
| ATP1A4 | Cardiac muscle contraction |
| ATP2A2 | Cardiac muscle contraction |
| CACNA1C | Cardiac muscle contraction |
| CACNA1D | Cardiac muscle contraction |
| CACNA1S | Cardiac muscle contraction |
| CACNA2D1 | Cardiac muscle contraction |
| CACNB1 | Cardiac muscle contraction |
| CACNB2 | Cardiac muscle contraction |
| CACNB4 | Cardiac muscle contraction |
| COX6C | Cardiac muscle contraction |
| MYH6 | Cardiac muscle contraction |
| MYH7 | Cardiac muscle contraction |
| RYR2 | Cardiac muscle contraction |
| SLC8A1 | Cardiac muscle contraction |
| SLC9A1 | Cardiac muscle contraction |
| TNNT2 | Cardiac muscle contraction |
| TPM4 | Cardiac muscle contraction |
| CACNA2D2 | Cardiac muscle contraction |
| ATP1B4 | Cardiac muscle contraction |
| UQCRQ | Cardiac muscle contraction |
| CACNA2D3 | Cardiac muscle contraction |
| CACNG7 | Cardiac muscle contraction |
| COX4I2 | Cardiac muscle contraction |
| CACNA2D4 | Cardiac muscle contraction |
| COX7B2 | Cardiac muscle contraction |
| RHOA | T cell receptor signaling pathway |
| CBL | T cell receptor signaling pathway |
| CBLB | T cell receptor signaling pathway |
| CHUK | T cell receptor signaling pathway |
| MAP3K8 | T cell receptor signaling pathway |
| DLG1 | T cell receptor signaling pathway |
| FYN | T cell receptor signaling pathway |
| HRAS | T cell receptor signaling pathway |
| IFNG | T cell receptor signaling pathway |
| ITK | T cell receptor signaling pathway |
| LCK | T cell receptor signaling pathway |
| LCP2 | T cell receptor signaling pathway |
| NFATC1 | T cell receptor signaling pathway |
| NFATC3 | T cell receptor signaling pathway |
| NFATC4 | T cell receptor signaling pathway |
| NFKB1 | T cell receptor signaling pathway |
| NFKBIA | T cell receptor signaling pathway |
| PAK1 | T cell receptor signaling pathway |
| PAK2 | T cell receptor signaling pathway |
| PAK3 | T cell receptor signaling pathway |
| PIK3CA | T cell receptor signaling pathway |
| PIK3CD | T cell receptor signaling pathway |
| PIK3CG | T cell receptor signaling pathway |
| PIK3R1 | T cell receptor signaling pathway |
| PLCG1 | T cell receptor signaling pathway |
| PPP3CB | T cell receptor signaling pathway |
| PPP3CC | T cell receptor signaling pathway |
| PRKCQ | T cell receptor signaling pathway |
| MAPK9 | T cell receptor signaling pathway |
| MAP2K2 | T cell receptor signaling pathway |
| PTPRC | T cell receptor signaling pathway |
| RAF1 | T cell receptor signaling pathway |
| SOS1 | T cell receptor signaling pathway |
| SOS2 | T cell receptor signaling pathway |
| MAP3K7 | T cell receptor signaling pathway |
| VAV1 | T cell receptor signaling pathway |
| VAV2 | T cell receptor signaling pathway |
| MAP3K14 | T cell receptor signaling pathway |
| PAK4 | T cell receptor signaling pathway |
| VAV3 | T cell receptor signaling pathway |
| NFAT5 | T cell receptor signaling pathway |
| PAK6 | T cell receptor signaling pathway |
| PAK7 | T cell receptor signaling pathway |
| CARD11 | T cell receptor signaling pathway |
| ADCY6 | Endocrine and other factor-regulated calcium reabsorption |
| ADCY9 | Endocrine and other factor-regulated calcium reabsorption |
| AP2A1 | Endocrine and other factor-regulated calcium reabsorption |
| AP2B1 | Endocrine and other factor-regulated calcium reabsorption |
| ATP1A1 | Endocrine and other factor-regulated calcium reabsorption |
| ATP1A3 | Endocrine and other factor-regulated calcium reabsorption |
| ATP1A4 | Endocrine and other factor-regulated calcium reabsorption |
| ATP2B1 | Endocrine and other factor-regulated calcium reabsorption |
| CLTC | Endocrine and other factor-regulated calcium reabsorption |
| DNM1 | Endocrine and other factor-regulated calcium reabsorption |
| GNAS | Endocrine and other factor-regulated calcium reabsorption |
| KLK2 | Endocrine and other factor-regulated calcium reabsorption |
| PLCB2 | Endocrine and other factor-regulated calcium reabsorption |
| PLCB3 | Endocrine and other factor-regulated calcium reabsorption |
| PLCB4 | Endocrine and other factor-regulated calcium reabsorption |
| PRKACA | Endocrine and other factor-regulated calcium reabsorption |
| PRKACB | Endocrine and other factor-regulated calcium reabsorption |
| PRKACG | Endocrine and other factor-regulated calcium reabsorption |
| PRKCB | Endocrine and other factor-regulated calcium reabsorption |
| PTH1R | Endocrine and other factor-regulated calcium reabsorption |
| SLC8A1 | Endocrine and other factor-regulated calcium reabsorption |
| PLCB1 | Endocrine and other factor-regulated calcium reabsorption |
| ATP1B4 | Endocrine and other factor-regulated calcium reabsorption |
| TRPV5 | Endocrine and other factor-regulated calcium reabsorption |
| ATP6V1A | Phagosome |
| ATP6V1B1 | Phagosome |
| C1R | Phagosome |
| C3 | Phagosome |
| CANX | Phagosome |
| CYBA | Phagosome |
| DYNC1H1 | Phagosome |
| DYNC1I2 | Phagosome |
| DYNC1LI2 | Phagosome |
| FCAR | Phagosome |
| FCGR2A | Phagosome |
| HLA-A | Phagosome |
| HLA-E | Phagosome |
| ITGA2 | Phagosome |
| ITGA5 | Phagosome |
| ITGAM | Phagosome |
| ITGAV | Phagosome |
| ITGB1 | Phagosome |
| ITGB2 | Phagosome |
| ITGB5 | Phagosome |
| LAMP1 | Phagosome |
| M6PR | Phagosome |
| MBL2 | Phagosome |
| MPO | Phagosome |
| MSR1 | Phagosome |
| NOS1 | Phagosome |
| PIK3C3 | Phagosome |
| RAB5B | Phagosome |
| TAP1 | Phagosome |
| THBS1 | Phagosome |
| THBS2 | Phagosome |
| THBS3 | Phagosome |
| THBS4 | Phagosome |
| TLR4 | Phagosome |
| TUBA3C | Phagosome |
| TUBB2A | Phagosome |
| STX7 | Phagosome |
| ATP6V1F | Phagosome |
| PLA2R1 | Phagosome |
| ATP6V0A2 | Phagosome |
| NOX1 | Phagosome |
| SEC61A1 | Phagosome |
| NOX3 | Phagosome |
| ATP6V0A4 | Phagosome |
| CLEC7A | Phagosome |
| DYNC2H1 | Phagosome |
| TUBAL3 | Phagosome |
| TUBA1C | Phagosome |
| TUBA3E | Phagosome |
| PIKFYVE | Phagosome |
| SFTPA2 | Phagosome |
| HRAS | VEGF signaling pathway |
| KDR | VEGF signaling pathway |
| NFATC1 | VEGF signaling pathway |
| NFATC3 | VEGF signaling pathway |
| NFATC4 | VEGF signaling pathway |
| NOS3 | VEGF signaling pathway |
| PIK3CA | VEGF signaling pathway |
| PIK3CD | VEGF signaling pathway |
| PIK3CG | VEGF signaling pathway |
| PIK3R1 | VEGF signaling pathway |
| PLA2G4A | VEGF signaling pathway |
| PLCG1 | VEGF signaling pathway |
| PPP3CB | VEGF signaling pathway |
| PPP3CC | VEGF signaling pathway |
| PRKCB | VEGF signaling pathway |
| MAP2K2 | VEGF signaling pathway |
| PTGS2 | VEGF signaling pathway |
| PTK2 | VEGF signaling pathway |
| RAF1 | VEGF signaling pathway |
| MAPKAPK3 | VEGF signaling pathway |
| PLA2G4C | VEGF signaling pathway |
| SH2D2A | VEGF signaling pathway |
| NFAT5 | VEGF signaling pathway |
| SPHK2 | VEGF signaling pathway |
| PLA2G4F | VEGF signaling pathway |
| PLA2G4D | VEGF signaling pathway |
| A2M | Complement and coagulation cascades |
| SERPINC1 | Complement and coagulation cascades |
| SERPING1 | Complement and coagulation cascades |
| C1QA | Complement and coagulation cascades |
| C1R | Complement and coagulation cascades |
| C1S | Complement and coagulation cascades |
| C2 | Complement and coagulation cascades |
| C3 | Complement and coagulation cascades |
| C5 | Complement and coagulation cascades |
| C6 | Complement and coagulation cascades |
| C7 | Complement and coagulation cascades |
| C8B | Complement and coagulation cascades |
| C9 | Complement and coagulation cascades |
| CPB2 | Complement and coagulation cascades |
| CR1 | Complement and coagulation cascades |
| F2 | Complement and coagulation cascades |
| F2R | Complement and coagulation cascades |
| F5 | Complement and coagulation cascades |
| F8 | Complement and coagulation cascades |
| F12 | Complement and coagulation cascades |
| F13B | Complement and coagulation cascades |
| FGA | Complement and coagulation cascades |
| FGB | Complement and coagulation cascades |
| FGG | Complement and coagulation cascades |
| CFH | Complement and coagulation cascades |
| KLKB1 | Complement and coagulation cascades |
| MBL2 | Complement and coagulation cascades |
| CD46 | Complement and coagulation cascades |
| SERPINE1 | Complement and coagulation cascades |
| SERPINA1 | Complement and coagulation cascades |
| PLAU | Complement and coagulation cascades |
| PLG | Complement and coagulation cascades |
| PROS1 | Complement and coagulation cascades |
| MASP1 | Complement and coagulation cascades |
| VWF | Complement and coagulation cascades |
| MASP2 | Complement and coagulation cascades |
| AMPH | Fc gamma R-mediated phagocytosis |
| DNM1 | Fc gamma R-mediated phagocytosis |
| DOCK2 | Fc gamma R-mediated phagocytosis |
| FCGR2A | Fc gamma R-mediated phagocytosis |
| LIMK2 | Fc gamma R-mediated phagocytosis |
| MYO10 | Fc gamma R-mediated phagocytosis |
| PAK1 | Fc gamma R-mediated phagocytosis |
| PIK3CA | Fc gamma R-mediated phagocytosis |
| PIK3CD | Fc gamma R-mediated phagocytosis |
| PIK3CG | Fc gamma R-mediated phagocytosis |
| PIK3R1 | Fc gamma R-mediated phagocytosis |
| PLA2G4A | Fc gamma R-mediated phagocytosis |
| PLCG1 | Fc gamma R-mediated phagocytosis |
| PRKCB | Fc gamma R-mediated phagocytosis |
| PTPRC | Fc gamma R-mediated phagocytosis |
| RAF1 | Fc gamma R-mediated phagocytosis |
| RPS6KB1 | Fc gamma R-mediated phagocytosis |
| RPS6KB2 | Fc gamma R-mediated phagocytosis |
| SYK | Fc gamma R-mediated phagocytosis |
| VAV1 | Fc gamma R-mediated phagocytosis |
| VAV2 | Fc gamma R-mediated phagocytosis |
| PIP5K1A | Fc gamma R-mediated phagocytosis |
| PLA2G6 | Fc gamma R-mediated phagocytosis |
| ARPC1B | Fc gamma R-mediated phagocytosis |
| VAV3 | Fc gamma R-mediated phagocytosis |
| ARPC1A | Fc gamma R-mediated phagocytosis |
| PIP5K1C | Fc gamma R-mediated phagocytosis |
| ASAP1 | Fc gamma R-mediated phagocytosis |
| SPHK2 | Fc gamma R-mediated phagocytosis |
| PLA2G4F | Fc gamma R-mediated phagocytosis |
| PLA2G4D | Fc gamma R-mediated phagocytosis |
| ADCY2 | Oocyte meiosis |
| ADCY3 | Oocyte meiosis |
| ADCY6 | Oocyte meiosis |
| ADCY8 | Oocyte meiosis |
| ADCY9 | Oocyte meiosis |
| AR | Oocyte meiosis |
| BUB1 | Oocyte meiosis |
| CALML3 | Oocyte meiosis |
| CCNB1 | Oocyte meiosis |
| CDC25C | Oocyte meiosis |
| CDC27 | Oocyte meiosis |
| IGF1R | Oocyte meiosis |
| ITPR1 | Oocyte meiosis |
| ITPR2 | Oocyte meiosis |
| ITPR3 | Oocyte meiosis |
| PGR | Oocyte meiosis |
| PPP2CB | Oocyte meiosis |
| PPP2R1B | Oocyte meiosis |
| PPP3CB | Oocyte meiosis |
| PPP3CC | Oocyte meiosis |
| PRKACA | Oocyte meiosis |
| PRKACB | Oocyte meiosis |
| PRKACG | Oocyte meiosis |
| RPS6KA3 | Oocyte meiosis |
| YWHAB | Oocyte meiosis |
| YWHAZ | Oocyte meiosis |
| CUL1 | Oocyte meiosis |
| CDC16 | Oocyte meiosis |
| BTRC | Oocyte meiosis |
| SMC3 | Oocyte meiosis |
| MAD2L2 | Oocyte meiosis |
| STAG3 | Oocyte meiosis |
| PTTG2 | Oocyte meiosis |
| SMC1B | Oocyte meiosis |
| ANAPC2 | Oocyte meiosis |
| ANAPC7 | Oocyte meiosis |
| CPEB1 | Oocyte meiosis |
| SGOL1 | Oocyte meiosis |
| FBXO43 | Oocyte meiosis |
| SPDYC | Oocyte meiosis |
| ATR | Fanconi anemia pathway |
| BLM | Fanconi anemia pathway |
| BRCA1 | Fanconi anemia pathway |
| BRCA2 | Fanconi anemia pathway |
| ERCC4 | Fanconi anemia pathway |
| FANCC | Fanconi anemia pathway |
| FANCD2 | Fanconi anemia pathway |
| FANCE | Fanconi anemia pathway |
| FANCB | Fanconi anemia pathway |
| FANCG | Fanconi anemia pathway |
| MLH1 | Fanconi anemia pathway |
| RAD51 | Fanconi anemia pathway |
| REV3L | Fanconi anemia pathway |
| RPA1 | Fanconi anemia pathway |
| TOP3A | Fanconi anemia pathway |
| USP1 | Fanconi anemia pathway |
| TOP3B | Fanconi anemia pathway |
| RPA4 | Fanconi anemia pathway |
| POLK | Fanconi anemia pathway |
| REV1 | Fanconi anemia pathway |
| FANCL | Fanconi anemia pathway |
| FANCM | Fanconi anemia pathway |
| BRIP1 | Fanconi anemia pathway |
| SLX4 | Fanconi anemia pathway |
| POLN | Fanconi anemia pathway |
| CBL | Jak-STAT signaling pathway |
| CBLB | Jak-STAT signaling pathway |
| CNTFR | Jak-STAT signaling pathway |
| CREBBP | Jak-STAT signaling pathway |
| CSF2RB | Jak-STAT signaling pathway |
| CSF3R | Jak-STAT signaling pathway |
| EP300 | Jak-STAT signaling pathway |
| GH2 | Jak-STAT signaling pathway |
| IFNA7 | Jak-STAT signaling pathway |
| IFNA10 | Jak-STAT signaling pathway |
| IFNAR1 | Jak-STAT signaling pathway |
| IFNAR2 | Jak-STAT signaling pathway |
| IFNG | Jak-STAT signaling pathway |
| IFNW1 | Jak-STAT signaling pathway |
| IL2RB | Jak-STAT signaling pathway |
| IL4R | Jak-STAT signaling pathway |
| IL6ST | Jak-STAT signaling pathway |
| IL7R | Jak-STAT signaling pathway |
| IL11 | Jak-STAT signaling pathway |
| IL12RB1 | Jak-STAT signaling pathway |
| IL12RB2 | Jak-STAT signaling pathway |
| IL13RA2 | Jak-STAT signaling pathway |
| JAK1 | Jak-STAT signaling pathway |
| JAK2 | Jak-STAT signaling pathway |
| JAK3 | Jak-STAT signaling pathway |
| LEPR | Jak-STAT signaling pathway |
| LIFR | Jak-STAT signaling pathway |
| PIK3CA | Jak-STAT signaling pathway |
| PIK3CD | Jak-STAT signaling pathway |
| PIK3CG | Jak-STAT signaling pathway |
| PIK3R1 | Jak-STAT signaling pathway |
| PRL | Jak-STAT signaling pathway |
| PTPN11 | Jak-STAT signaling pathway |
| SOS1 | Jak-STAT signaling pathway |
| SOS2 | Jak-STAT signaling pathway |
| STAT1 | Jak-STAT signaling pathway |
| STAT2 | Jak-STAT signaling pathway |
| STAT5B | Jak-STAT signaling pathway |
| STAT6 | Jak-STAT signaling pathway |
| TPO | Jak-STAT signaling pathway |
| STAM | Jak-STAT signaling pathway |
| SOCS3 | Jak-STAT signaling pathway |
| OSMR | Jak-STAT signaling pathway |
| SOCS5 | Jak-STAT signaling pathway |
| STAM2 | Jak-STAT signaling pathway |
| IRF9 | Jak-STAT signaling pathway |
| IL21R | Jak-STAT signaling pathway |
| IL20RB | Jak-STAT signaling pathway |
| IL21 | Jak-STAT signaling pathway |
| SOCS4 | Jak-STAT signaling pathway |
| IL23R | Jak-STAT signaling pathway |
| SPRED1 | Jak-STAT signaling pathway |
| SPRED2 | Jak-STAT signaling pathway |
| ADCY2 | Ovarian steroidogenesis |
| ADCY3 | Ovarian steroidogenesis |
| ADCY6 | Ovarian steroidogenesis |
| ADCY8 | Ovarian steroidogenesis |
| ADCY9 | Ovarian steroidogenesis |
| ALOX5 | Ovarian steroidogenesis |
| BMP6 | Ovarian steroidogenesis |
| FSHR | Ovarian steroidogenesis |
| GNAS | Ovarian steroidogenesis |
| HSD3B1 | Ovarian steroidogenesis |
| IGF1R | Ovarian steroidogenesis |
| LDLR | Ovarian steroidogenesis |
| LHCGR | Ovarian steroidogenesis |
| PLA2G4A | Ovarian steroidogenesis |
| PRKACA | Ovarian steroidogenesis |
| PRKACB | Ovarian steroidogenesis |
| PRKACG | Ovarian steroidogenesis |
| PTGS2 | Ovarian steroidogenesis |
| PLA2G4C | Ovarian steroidogenesis |
| PLA2G4F | Ovarian steroidogenesis |
| PLA2G4D | Ovarian steroidogenesis |
| AMY2A | Carbohydrate digestion and absorption |
| ATP1A1 | Carbohydrate digestion and absorption |
| ATP1A3 | Carbohydrate digestion and absorption |
| ATP1A4 | Carbohydrate digestion and absorption |
| CACNA1D | Carbohydrate digestion and absorption |
| G6PC | Carbohydrate digestion and absorption |
| HK1 | Carbohydrate digestion and absorption |
| HK2 | Carbohydrate digestion and absorption |
| LCT | Carbohydrate digestion and absorption |
| PIK3CA | Carbohydrate digestion and absorption |
| PIK3CD | Carbohydrate digestion and absorption |
| PIK3CG | Carbohydrate digestion and absorption |
| PIK3R1 | Carbohydrate digestion and absorption |
| PLCB2 | Carbohydrate digestion and absorption |
| PRKCB | Carbohydrate digestion and absorption |
| SI | Carbohydrate digestion and absorption |
| SLC2A2 | Carbohydrate digestion and absorption |
| SLC2A5 | Carbohydrate digestion and absorption |
| SLC5A1 | Carbohydrate digestion and absorption |
| MGAM | Carbohydrate digestion and absorption |
| ATP1B4 | Carbohydrate digestion and absorption |
| HKDC1 | Carbohydrate digestion and absorption |
| TAS1R2 | Carbohydrate digestion and absorption |
| ADCY2 | Purine metabolism |
| ADCY3 | Purine metabolism |
| ADCY6 | Purine metabolism |
| ADCY8 | Purine metabolism |
| ADCY9 | Purine metabolism |
| ADSL | Purine metabolism |
| AMPD1 | Purine metabolism |
| AMPD3 | Purine metabolism |
| NUDT2 | Purine metabolism |
| APRT | Purine metabolism |
| ENTPD2 | Purine metabolism |
| ENTPD3 | Purine metabolism |
| DGUOK | Purine metabolism |
| GART | Purine metabolism |
| GUCY1A2 | Purine metabolism |
| GUCY1A3 | Purine metabolism |
| GUCY2C | Purine metabolism |
| GUCY2F | Purine metabolism |
| GUCY2D | Purine metabolism |
| HPRT1 | Purine metabolism |
| IMPDH1 | Purine metabolism |
| NPR1 | Purine metabolism |
| PDE1A | Purine metabolism |
| PDE1C | Purine metabolism |
| PDE2A | Purine metabolism |
| PDE3A | Purine metabolism |
| PDE3B | Purine metabolism |
| PDE4A | Purine metabolism |
| PDE4B | Purine metabolism |
| PDE4D | Purine metabolism |
| PDE6C | Purine metabolism |
| PDE6H | Purine metabolism |
| PDE8A | Purine metabolism |
| PDE1B | Purine metabolism |
| PDE6B | Purine metabolism |
| ENPP3 | Purine metabolism |
| PFAS | Purine metabolism |
| PKLR | Purine metabolism |
| POLA1 | Purine metabolism |
| POLD1 | Purine metabolism |
| POLD2 | Purine metabolism |
| POLR2F | Purine metabolism |
| POLR2L | Purine metabolism |
| PRPS2 | Purine metabolism |
| XDH | Purine metabolism |
| PDE5A | Purine metabolism |
| POLR1C | Purine metabolism |
| PAICS | Purine metabolism |
| POLR3C | Purine metabolism |
| PDE10A | Purine metabolism |
| POLR1A | Purine metabolism |
| AK5 | Purine metabolism |
| PDE11A | Purine metabolism |
| PGM2 | Purine metabolism |
| POLR3E | Purine metabolism |
| POLR3GL | Purine metabolism |
| NT5C1A | Purine metabolism |
| NUDT16 | Purine metabolism |
| EGFR | Dorso-ventral axis formation |
| ETS1 | Dorso-ventral axis formation |
| ETS2 | Dorso-ventral axis formation |
| ETV6 | Dorso-ventral axis formation |
| NOTCH2 | Dorso-ventral axis formation |
| NOTCH3 | Dorso-ventral axis formation |
| NOTCH4 | Dorso-ventral axis formation |
| SOS1 | Dorso-ventral axis formation |
| SOS2 | Dorso-ventral axis formation |
| PIWIL1 | Dorso-ventral axis formation |
| FMN2 | Dorso-ventral axis formation |
| SPIRE1 | Dorso-ventral axis formation |
| CPEB1 | Dorso-ventral axis formation |
| PIWIL4 | Dorso-ventral axis formation |
| PIWIL3 | Dorso-ventral axis formation |
| CD22 | B cell receptor signaling pathway |
| CD72 | B cell receptor signaling pathway |
| CD79A | B cell receptor signaling pathway |
| CD79B | B cell receptor signaling pathway |
| CHUK | B cell receptor signaling pathway |
| HRAS | B cell receptor signaling pathway |
| NFATC1 | B cell receptor signaling pathway |
| NFATC3 | B cell receptor signaling pathway |
| NFATC4 | B cell receptor signaling pathway |
| NFKB1 | B cell receptor signaling pathway |
| NFKBIA | B cell receptor signaling pathway |
| PIK3CA | B cell receptor signaling pathway |
| PIK3CD | B cell receptor signaling pathway |
| PIK3CG | B cell receptor signaling pathway |
| PIK3R1 | B cell receptor signaling pathway |
| PPP3CB | B cell receptor signaling pathway |
| PPP3CC | B cell receptor signaling pathway |
| PRKCB | B cell receptor signaling pathway |
| MAP2K2 | B cell receptor signaling pathway |
| RAF1 | B cell receptor signaling pathway |
| SOS1 | B cell receptor signaling pathway |
| SOS2 | B cell receptor signaling pathway |
| SYK | B cell receptor signaling pathway |
| VAV1 | B cell receptor signaling pathway |
| VAV2 | B cell receptor signaling pathway |
| VAV3 | B cell receptor signaling pathway |
| NFAT5 | B cell receptor signaling pathway |
| LILRB3 | B cell receptor signaling pathway |
| RASGRP3 | B cell receptor signaling pathway |
| DAPP1 | B cell receptor signaling pathway |
| BLNK | B cell receptor signaling pathway |
| CARD11 | B cell receptor signaling pathway |
| BIRC2 | NOD-like receptor signaling pathway |
| CHUK | NOD-like receptor signaling pathway |
| CXCL2 | NOD-like receptor signaling pathway |
| HSP90AA1 | NOD-like receptor signaling pathway |
| HSP90AB1 | NOD-like receptor signaling pathway |
| IL1B | NOD-like receptor signaling pathway |
| MEFV | NOD-like receptor signaling pathway |
| NFKB1 | NOD-like receptor signaling pathway |
| NFKBIA | NOD-like receptor signaling pathway |
| MAPK8 | NOD-like receptor signaling pathway |
| MAPK9 | NOD-like receptor signaling pathway |
| CCL5 | NOD-like receptor signaling pathway |
| MAP3K7 | NOD-like receptor signaling pathway |
| TNFAIP3 | NOD-like receptor signaling pathway |
| TRAF6 | NOD-like receptor signaling pathway |
| TRIP6 | NOD-like receptor signaling pathway |
| PSTPIP1 | NOD-like receptor signaling pathway |
| NOD1 | NOD-like receptor signaling pathway |
| TAB1 | NOD-like receptor signaling pathway |
| NLRP1 | NOD-like receptor signaling pathway |
| CARD8 | NOD-like receptor signaling pathway |
| TAB2 | NOD-like receptor signaling pathway |
| ERBB2IP | NOD-like receptor signaling pathway |
| NLRC4 | NOD-like receptor signaling pathway |
| CARD9 | NOD-like receptor signaling pathway |
| CARD6 | NOD-like receptor signaling pathway |
| NLRP3 | NOD-like receptor signaling pathway |
| ACAA1 | PPAR signaling pathway |
| ACADL | PPAR signaling pathway |
| ACOX1 | PPAR signaling pathway |
| APOA1 | PPAR signaling pathway |
| CPT1A | PPAR signaling pathway |
| CPT2 | PPAR signaling pathway |
| CYP8B1 | PPAR signaling pathway |
| EHHADH | PPAR signaling pathway |
| FABP6 | PPAR signaling pathway |
| ACSL1 | PPAR signaling pathway |
| GK | PPAR signaling pathway |
| HMGCS2 | PPAR signaling pathway |
| LPL | PPAR signaling pathway |
| ME1 | PPAR signaling pathway |
| MMP1 | PPAR signaling pathway |
| PCK1 | PPAR signaling pathway |
| PLTP | PPAR signaling pathway |
| PPARA | PPAR signaling pathway |
| PPARD | PPAR signaling pathway |
| PPARG | PPAR signaling pathway |
| RXRB | PPAR signaling pathway |
| RXRG | PPAR signaling pathway |
| SCD | PPAR signaling pathway |
| SCP2 | PPAR signaling pathway |
| ACOX3 | PPAR signaling pathway |
| FADS2 | PPAR signaling pathway |
| NR1H3 | PPAR signaling pathway |
| SLC27A2 | PPAR signaling pathway |
| ACSBG1 | PPAR signaling pathway |
| ACSL6 | PPAR signaling pathway |
| SLC27A6 | PPAR signaling pathway |
| ANGPTL4 | PPAR signaling pathway |
| SCD5 | PPAR signaling pathway |
| ACSBG2 | PPAR signaling pathway |
| CPT1C | PPAR signaling pathway |
| BRAF | mTOR signaling pathway |
| MTOR | mTOR signaling pathway |
| HIF1A | mTOR signaling pathway |
| PIK3CA | mTOR signaling pathway |
| PIK3CD | mTOR signaling pathway |
| PIK3CG | mTOR signaling pathway |
| PIK3R1 | mTOR signaling pathway |
| PRKCB | mTOR signaling pathway |
| PTEN | mTOR signaling pathway |
| RPS6KA3 | mTOR signaling pathway |
| RPS6KB1 | mTOR signaling pathway |
| RPS6KB2 | mTOR signaling pathway |
| TSC1 | mTOR signaling pathway |
| TSC2 | mTOR signaling pathway |
| EIF4E2 | mTOR signaling pathway |
| ULK2 | mTOR signaling pathway |
| CAB39 | mTOR signaling pathway |
| RPTOR | mTOR signaling pathway |
| STRADA | mTOR signaling pathway |
| RICTOR | mTOR signaling pathway |
| ACACB | Adipocytokine signaling pathway |
| CHUK | Adipocytokine signaling pathway |
| CPT1A | Adipocytokine signaling pathway |
| ACSL1 | Adipocytokine signaling pathway |
| MTOR | Adipocytokine signaling pathway |
| G6PC | Adipocytokine signaling pathway |
| JAK2 | Adipocytokine signaling pathway |
| LEPR | Adipocytokine signaling pathway |
| NFKB1 | Adipocytokine signaling pathway |
| NFKBIA | Adipocytokine signaling pathway |
| PCK1 | Adipocytokine signaling pathway |
| PPARA | Adipocytokine signaling pathway |
| PRKCQ | Adipocytokine signaling pathway |
| MAPK8 | Adipocytokine signaling pathway |
| MAPK9 | Adipocytokine signaling pathway |
| PTPN11 | Adipocytokine signaling pathway |
| RXRB | Adipocytokine signaling pathway |
| RXRG | Adipocytokine signaling pathway |
| SLC2A1 | Adipocytokine signaling pathway |
| TNFRSF1B | Adipocytokine signaling pathway |
| IRS4 | Adipocytokine signaling pathway |
| SOCS3 | Adipocytokine signaling pathway |
| CAMKK2 | Adipocytokine signaling pathway |
| PPARGC1A | Adipocytokine signaling pathway |
| ACSBG1 | Adipocytokine signaling pathway |
| ACSL6 | Adipocytokine signaling pathway |
| ADIPOR1 | Adipocytokine signaling pathway |
| PRKAG2 | Adipocytokine signaling pathway |
| ACSBG2 | Adipocytokine signaling pathway |
| CPT1C | Adipocytokine signaling pathway |
| PARP1 | NF-kappa B signaling pathway |
| BIRC2 | NF-kappa B signaling pathway |
| ATM | NF-kappa B signaling pathway |
| BCL2A1 | NF-kappa B signaling pathway |
| CD40 | NF-kappa B signaling pathway |
| CHUK | NF-kappa B signaling pathway |
| CSNK2B | NF-kappa B signaling pathway |
| CXCL2 | NF-kappa B signaling pathway |
| IL1B | NF-kappa B signaling pathway |
| IL1R1 | NF-kappa B signaling pathway |
| IRAK1 | NF-kappa B signaling pathway |
| LBP | NF-kappa B signaling pathway |
| LCK | NF-kappa B signaling pathway |
| LTBR | NF-kappa B signaling pathway |
| NFKB1 | NF-kappa B signaling pathway |
| NFKBIA | NF-kappa B signaling pathway |
| PLAU | NF-kappa B signaling pathway |
| PLCG1 | NF-kappa B signaling pathway |
| PRKCB | NF-kappa B signaling pathway |
| PRKCQ | NF-kappa B signaling pathway |
| PTGS2 | NF-kappa B signaling pathway |
| CXCL12 | NF-kappa B signaling pathway |
| SYK | NF-kappa B signaling pathway |
| MAP3K7 | NF-kappa B signaling pathway |
| TLR4 | NF-kappa B signaling pathway |
| TNFAIP3 | NF-kappa B signaling pathway |
| TRAF3 | NF-kappa B signaling pathway |
| TRAF6 | NF-kappa B signaling pathway |
| VCAM1 | NF-kappa B signaling pathway |
| TRIM25 | NF-kappa B signaling pathway |
| TNFSF11 | NF-kappa B signaling pathway |
| RIPK1 | NF-kappa B signaling pathway |
| TNFSF14 | NF-kappa B signaling pathway |
| TNFRSF11A | NF-kappa B signaling pathway |
| MAP3K14 | NF-kappa B signaling pathway |
| TAB1 | NF-kappa B signaling pathway |
| TAB2 | NF-kappa B signaling pathway |
| DDX58 | NF-kappa B signaling pathway |
| BLNK | NF-kappa B signaling pathway |
| CARD11 | NF-kappa B signaling pathway |
| TIRAP | NF-kappa B signaling pathway |
| ACVR1 | Cytokine-cytokine receptor interaction |
| ACVR2A | Cytokine-cytokine receptor interaction |
| TNFRSF17 | Cytokine-cytokine receptor interaction |
| TNFRSF8 | Cytokine-cytokine receptor interaction |
| CD40 | Cytokine-cytokine receptor interaction |
| CCR1 | Cytokine-cytokine receptor interaction |
| CCR6 | Cytokine-cytokine receptor interaction |
| CCR7 | Cytokine-cytokine receptor interaction |
| CNTFR | Cytokine-cytokine receptor interaction |
| CSF1 | Cytokine-cytokine receptor interaction |
| CSF1R | Cytokine-cytokine receptor interaction |
| CSF2RB | Cytokine-cytokine receptor interaction |
| CSF3R | Cytokine-cytokine receptor interaction |
| EGFR | Cytokine-cytokine receptor interaction |
| FLT1 | Cytokine-cytokine receptor interaction |
| FLT3 | Cytokine-cytokine receptor interaction |
| FLT4 | Cytokine-cytokine receptor interaction |
| GH2 | Cytokine-cytokine receptor interaction |
| CXCL2 | Cytokine-cytokine receptor interaction |
| HGF | Cytokine-cytokine receptor interaction |
| IFNA7 | Cytokine-cytokine receptor interaction |
| IFNA10 | Cytokine-cytokine receptor interaction |
| IFNAR1 | Cytokine-cytokine receptor interaction |
| IFNAR2 | Cytokine-cytokine receptor interaction |
| IFNG | Cytokine-cytokine receptor interaction |
| IFNW1 | Cytokine-cytokine receptor interaction |
| IL1B | Cytokine-cytokine receptor interaction |
| IL1R1 | Cytokine-cytokine receptor interaction |
| IL1RAP | Cytokine-cytokine receptor interaction |
| IL2RB | Cytokine-cytokine receptor interaction |
| IL4R | Cytokine-cytokine receptor interaction |
| IL6ST | Cytokine-cytokine receptor interaction |
| IL7R | Cytokine-cytokine receptor interaction |
| CXCR1 | Cytokine-cytokine receptor interaction |
| IL11 | Cytokine-cytokine receptor interaction |
| IL12RB1 | Cytokine-cytokine receptor interaction |
| IL12RB2 | Cytokine-cytokine receptor interaction |
| INHBA | Cytokine-cytokine receptor interaction |
| INHBC | Cytokine-cytokine receptor interaction |
| KDR | Cytokine-cytokine receptor interaction |
| KIT | Cytokine-cytokine receptor interaction |
| LEPR | Cytokine-cytokine receptor interaction |
| LIFR | Cytokine-cytokine receptor interaction |
| LTBR | Cytokine-cytokine receptor interaction |
| MET | Cytokine-cytokine receptor interaction |
| KITLG | Cytokine-cytokine receptor interaction |
| CXCL9 | Cytokine-cytokine receptor interaction |
| NGFR | Cytokine-cytokine receptor interaction |
| PDGFRA | Cytokine-cytokine receptor interaction |
| PF4V1 | Cytokine-cytokine receptor interaction |
| PRL | Cytokine-cytokine receptor interaction |
| CCL5 | Cytokine-cytokine receptor interaction |
| CCL11 | Cytokine-cytokine receptor interaction |
| CCL23 | Cytokine-cytokine receptor interaction |
| CXCL12 | Cytokine-cytokine receptor interaction |
| TGFBR1 | Cytokine-cytokine receptor interaction |
| TNFRSF1B | Cytokine-cytokine receptor interaction |
| TPO | Cytokine-cytokine receptor interaction |
| TNFSF4 | Cytokine-cytokine receptor interaction |
| VEGFB | Cytokine-cytokine receptor interaction |
| TNFSF11 | Cytokine-cytokine receptor interaction |
| TNFSF14 | Cytokine-cytokine receptor interaction |
| TNFSF13 | Cytokine-cytokine receptor interaction |
| TNFSF12 | Cytokine-cytokine receptor interaction |
| TNFRSF11A | Cytokine-cytokine receptor interaction |
| TNFRSF10D | Cytokine-cytokine receptor interaction |
| TNFRSF10B | Cytokine-cytokine receptor interaction |
| OSMR | Cytokine-cytokine receptor interaction |
| TNFSF15 | Cytokine-cytokine receptor interaction |
| CCR9 | Cytokine-cytokine receptor interaction |
| TNFRSF13B | Cytokine-cytokine receptor interaction |
| TNFRSF21 | Cytokine-cytokine receptor interaction |
| IL21R | Cytokine-cytokine receptor interaction |
| IL20RB | Cytokine-cytokine receptor interaction |
| TNFRSF19 | Cytokine-cytokine receptor interaction |
| IL21 | Cytokine-cytokine receptor interaction |
| IL23R | Cytokine-cytokine receptor interaction |
| CCR2 | Cytokine-cytokine receptor interaction |
| ALDH2 | Glycerolipid metabolism |
| ALDH3A2 | Glycerolipid metabolism |
| DGKA | Glycerolipid metabolism |
| DGKB | Glycerolipid metabolism |
| DGKG | Glycerolipid metabolism |
| GK | Glycerolipid metabolism |
| LIPC | Glycerolipid metabolism |
| LPL | Glycerolipid metabolism |
| PNLIP | Glycerolipid metabolism |
| PNLIPRP1 | Glycerolipid metabolism |
| DGKZ | Glycerolipid metabolism |
| DGKD | Glycerolipid metabolism |
| DGKI | Glycerolipid metabolism |
| LPIN2 | Glycerolipid metabolism |
| MGLL | Glycerolipid metabolism |
| LPIN1 | Glycerolipid metabolism |
| DAK | Glycerolipid metabolism |
| AGK | Glycerolipid metabolism |
| AGPAT3 | Glycerolipid metabolism |
| AGPAT4 | Glycerolipid metabolism |
| GPAM | Glycerolipid metabolism |
| LPIN3 | Glycerolipid metabolism |
| DGAT2 | Glycerolipid metabolism |
| AGPAT9 | Glycerolipid metabolism |
| MBOAT2 | Glycerolipid metabolism |
| GLYCTK | Glycerolipid metabolism |
| DGKH | Glycerolipid metabolism |
| MOGAT3 | Glycerolipid metabolism |
| DHX8 | Spliceosome |
| DHX15 | Spliceosome |
| HNRNPA1 | Spliceosome |
| HNRNPC | Spliceosome |
| HNRNPK | Spliceosome |
| HNRNPU | Spliceosome |
| HSPA8 | Spliceosome |
| NCBP1 | Spliceosome |
| NHP2L1 | Spliceosome |
| SRSF3 | Spliceosome |
| SRSF4 | Spliceosome |
| SRSF5 | Spliceosome |
| SRSF6 | Spliceosome |
| SNRPA1 | Spliceosome |
| U2AF1 | Spliceosome |
| SART1 | Spliceosome |
| PRPF4 | Spliceosome |
| PRPF3 | Spliceosome |
| EFTUD2 | Spliceosome |
| AQR | Spliceosome |
| DDX46 | Spliceosome |
| THOC1 | Spliceosome |
| SF3B4 | Spliceosome |
| BCAS2 | Spliceosome |
| TCERG1 | Spliceosome |
| SF3A3 | Spliceosome |
| SF3B2 | Spliceosome |
| DDX42 | Spliceosome |
| ACIN1 | Spliceosome |
| SNRNP200 | Spliceosome |
| U2SURP | Spliceosome |
| SF3B3 | Spliceosome |
| SF3B1 | Spliceosome |
| PRPF6 | Spliceosome |
| LSM3 | Spliceosome |
| RBMXL2 | Spliceosome |
| RBMX | Spliceosome |
| TRA2A | Spliceosome |
| CRNKL1 | Spliceosome |
| CDC40 | Spliceosome |
| PRPF40A | Spliceosome |
| THOC2 | Spliceosome |
| RBM25 | Spliceosome |
| PRPF38A | Spliceosome |
| RBMXL3 | Spliceosome |
| CCDC12 | Spliceosome |
| ZMAT2 | Spliceosome |
| ISY1-RAB43 | Spliceosome |
| CSNK1D | Hedgehog signaling pathway |
| CSNK1E | Hedgehog signaling pathway |
| CSNK1G3 | Hedgehog signaling pathway |
| GLI1 | Hedgehog signaling pathway |
| GLI2 | Hedgehog signaling pathway |
| GLI3 | Hedgehog signaling pathway |
| LRP2 | Hedgehog signaling pathway |
| PRKACA | Hedgehog signaling pathway |
| PRKACB | Hedgehog signaling pathway |
| PRKACG | Hedgehog signaling pathway |
| PTCH1 | Hedgehog signaling pathway |
| WNT2 | Hedgehog signaling pathway |
| WNT5A | Hedgehog signaling pathway |
| ZIC2 | Hedgehog signaling pathway |
| BTRC | Hedgehog signaling pathway |
| STK36 | Hedgehog signaling pathway |
| CSNK1G1 | Hedgehog signaling pathway |
| HHIP | Hedgehog signaling pathway |
| WNT10A | Hedgehog signaling pathway |
| FCER1G | Fc epsilon RI signaling pathway |
| FYN | Fc epsilon RI signaling pathway |
| HRAS | Fc epsilon RI signaling pathway |
| LCP2 | Fc epsilon RI signaling pathway |
| PIK3CA | Fc epsilon RI signaling pathway |
| PIK3CD | Fc epsilon RI signaling pathway |
| PIK3CG | Fc epsilon RI signaling pathway |
| PIK3R1 | Fc epsilon RI signaling pathway |
| PLA2G4A | Fc epsilon RI signaling pathway |
| PLCG1 | Fc epsilon RI signaling pathway |
| PRKCB | Fc epsilon RI signaling pathway |
| MAPK8 | Fc epsilon RI signaling pathway |
| MAPK9 | Fc epsilon RI signaling pathway |
| MAP2K2 | Fc epsilon RI signaling pathway |
| MAP2K3 | Fc epsilon RI signaling pathway |
| RAF1 | Fc epsilon RI signaling pathway |
| SOS1 | Fc epsilon RI signaling pathway |
| SOS2 | Fc epsilon RI signaling pathway |
| SYK | Fc epsilon RI signaling pathway |
| VAV1 | Fc epsilon RI signaling pathway |
| VAV2 | Fc epsilon RI signaling pathway |
| PLA2G4C | Fc epsilon RI signaling pathway |
| VAV3 | Fc epsilon RI signaling pathway |
| PLA2G4F | Fc epsilon RI signaling pathway |
| PLA2G4D | Fc epsilon RI signaling pathway |
| ALDOA | Fructose and mannose metabolism |
| ALDOB | Fructose and mannose metabolism |
| FBP1 | Fructose and mannose metabolism |
| HK1 | Fructose and mannose metabolism |
| HK2 | Fructose and mannose metabolism |
| KHK | Fructose and mannose metabolism |
| MPI | Fructose and mannose metabolism |
| PFKFB2 | Fructose and mannose metabolism |
| PFKFB3 | Fructose and mannose metabolism |
| PFKFB4 | Fructose and mannose metabolism |
| PFKL | Fructose and mannose metabolism |
| PFKM | Fructose and mannose metabolism |
| PFKP | Fructose and mannose metabolism |
| MTMR1 | Fructose and mannose metabolism |
| FPGT | Fructose and mannose metabolism |
| MTMR2 | Fructose and mannose metabolism |
| MTMR7 | Fructose and mannose metabolism |
| C12orf5 | Fructose and mannose metabolism |
| HKDC1 | Fructose and mannose metabolism |
| CHUK | Osteoclast differentiation |
| CSF1 | Osteoclast differentiation |
| CSF1R | Osteoclast differentiation |
| CYBA | Osteoclast differentiation |
| FCGR2A | Osteoclast differentiation |
| FYN | Osteoclast differentiation |
| IFNAR1 | Osteoclast differentiation |
| IFNAR2 | Osteoclast differentiation |
| IFNG | Osteoclast differentiation |
| IL1B | Osteoclast differentiation |
| IL1R1 | Osteoclast differentiation |
| JAK1 | Osteoclast differentiation |
| LCK | Osteoclast differentiation |
| LCP2 | Osteoclast differentiation |
| MITF | Osteoclast differentiation |
| NFATC1 | Osteoclast differentiation |
| NFKB1 | Osteoclast differentiation |
| NFKBIA | Osteoclast differentiation |
| PIK3CA | Osteoclast differentiation |
| PIK3CD | Osteoclast differentiation |
| PIK3CG | Osteoclast differentiation |
| PIK3R1 | Osteoclast differentiation |
| PPARG | Osteoclast differentiation |
| PPP3CB | Osteoclast differentiation |
| PPP3CC | Osteoclast differentiation |
| MAPK8 | Osteoclast differentiation |
| MAPK9 | Osteoclast differentiation |
| STAT1 | Osteoclast differentiation |
| STAT2 | Osteoclast differentiation |
| SYK | Osteoclast differentiation |
| MAP3K7 | Osteoclast differentiation |
| TGFBR1 | Osteoclast differentiation |
| TRAF6 | Osteoclast differentiation |
| TNFSF11 | Osteoclast differentiation |
| TNFRSF11A | Osteoclast differentiation |
| SQSTM1 | Osteoclast differentiation |
| MAP3K14 | Osteoclast differentiation |
| SOCS3 | Osteoclast differentiation |
| LILRB2 | Osteoclast differentiation |
| IRF9 | Osteoclast differentiation |
| TAB1 | Osteoclast differentiation |
| LILRB1 | Osteoclast differentiation |
| LILRA1 | Osteoclast differentiation |
| LILRB3 | Osteoclast differentiation |
| TAB2 | Osteoclast differentiation |
| NOX1 | Osteoclast differentiation |
| BLNK | Osteoclast differentiation |
| NOX3 | Osteoclast differentiation |
| LILRA6 | Osteoclast differentiation |
| OSCAR | Osteoclast differentiation |
| LILRA5 | Osteoclast differentiation |
| ABCA1 | Fat digestion and absorption |
| APOA1 | Fat digestion and absorption |
| APOA4 | Fat digestion and absorption |
| APOB | Fat digestion and absorption |
| MTTP | Fat digestion and absorption |
| PNLIP | Fat digestion and absorption |
| PNLIPRP1 | Fat digestion and absorption |
| PLA2G3 | Fat digestion and absorption |
| ABCG5 | Fat digestion and absorption |
| DGAT2 | Fat digestion and absorption |
| MOGAT3 | Fat digestion and absorption |
| DDX6 | RNA degradation |
| HSPA9 | RNA degradation |
| HSPD1 | RNA degradation |
| CNOT2 | RNA degradation |
| CNOT3 | RNA degradation |
| CNOT4 | RNA degradation |
| PARN | RNA degradation |
| SKIV2L | RNA degradation |
| PABPC4 | RNA degradation |
| RQCD1 | RNA degradation |
| TTC37 | RNA degradation |
| PAN2 | RNA degradation |
| TOB1 | RNA degradation |
| TOB2 | RNA degradation |
| DIS3 | RNA degradation |
| CNOT1 | RNA degradation |
| CNOT10 | RNA degradation |
| PABPC1 | RNA degradation |
| LSM3 | RNA degradation |
| XRN1 | RNA degradation |
| BTG4 | RNA degradation |
| EDC3 | RNA degradation |
| WDR61 | RNA degradation |
| NUDT16 | RNA degradation |
| DHX36 | RNA degradation |
| DCP1B | RNA degradation |
| PATL1 | RNA degradation |
| ARAF | Natural killer cell mediated cytotoxicity |
| BRAF | Natural killer cell mediated cytotoxicity |
| PTK2B | Natural killer cell mediated cytotoxicity |
| FCER1G | Natural killer cell mediated cytotoxicity |
| FYN | Natural killer cell mediated cytotoxicity |
| HLA-A | Natural killer cell mediated cytotoxicity |
| HLA-E | Natural killer cell mediated cytotoxicity |
| HRAS | Natural killer cell mediated cytotoxicity |
| IFNA7 | Natural killer cell mediated cytotoxicity |
| IFNA10 | Natural killer cell mediated cytotoxicity |
| IFNAR1 | Natural killer cell mediated cytotoxicity |
| IFNAR2 | Natural killer cell mediated cytotoxicity |
| IFNG | Natural killer cell mediated cytotoxicity |
| ITGB2 | Natural killer cell mediated cytotoxicity |
| KIR2DL4 | Natural killer cell mediated cytotoxicity |
| KIR2DS4 | Natural killer cell mediated cytotoxicity |
| KLRC2 | Natural killer cell mediated cytotoxicity |
| LCK | Natural killer cell mediated cytotoxicity |
| LCP2 | Natural killer cell mediated cytotoxicity |
| NFATC1 | Natural killer cell mediated cytotoxicity |
| NFATC3 | Natural killer cell mediated cytotoxicity |
| NFATC4 | Natural killer cell mediated cytotoxicity |
| PAK1 | Natural killer cell mediated cytotoxicity |
| PIK3CA | Natural killer cell mediated cytotoxicity |
| PIK3CD | Natural killer cell mediated cytotoxicity |
| PIK3CG | Natural killer cell mediated cytotoxicity |
| PIK3R1 | Natural killer cell mediated cytotoxicity |
| PLCG1 | Natural killer cell mediated cytotoxicity |
| PPP3CB | Natural killer cell mediated cytotoxicity |
| PPP3CC | Natural killer cell mediated cytotoxicity |
| PRKCB | Natural killer cell mediated cytotoxicity |
| MAP2K2 | Natural killer cell mediated cytotoxicity |
| PTPN11 | Natural killer cell mediated cytotoxicity |
| RAF1 | Natural killer cell mediated cytotoxicity |
| SOS1 | Natural killer cell mediated cytotoxicity |
| SOS2 | Natural killer cell mediated cytotoxicity |
| SYK | Natural killer cell mediated cytotoxicity |
| VAV1 | Natural killer cell mediated cytotoxicity |
| VAV2 | Natural killer cell mediated cytotoxicity |
| TNFRSF10D | Natural killer cell mediated cytotoxicity |
| TNFRSF10B | Natural killer cell mediated cytotoxicity |
| NCR2 | Natural killer cell mediated cytotoxicity |
| NCR1 | Natural killer cell mediated cytotoxicity |
| VAV3 | Natural killer cell mediated cytotoxicity |
| NFAT5 | Natural killer cell mediated cytotoxicity |
| ULBP3 | Natural killer cell mediated cytotoxicity |
| ULBP1 | Natural killer cell mediated cytotoxicity |
| NCR3 | Natural killer cell mediated cytotoxicity |
| SHC4 | Natural killer cell mediated cytotoxicity |
| MICA | Natural killer cell mediated cytotoxicity |
| ATP1A1 | Aldosterone-regulated sodium reabsorption |
| ATP1A3 | Aldosterone-regulated sodium reabsorption |
| ATP1A4 | Aldosterone-regulated sodium reabsorption |
| SFN | Aldosterone-regulated sodium reabsorption |
| HSD11B2 | Aldosterone-regulated sodium reabsorption |
| NR3C2 | Aldosterone-regulated sodium reabsorption |
| PIK3CA | Aldosterone-regulated sodium reabsorption |
| PIK3CD | Aldosterone-regulated sodium reabsorption |
| PIK3CG | Aldosterone-regulated sodium reabsorption |
| PIK3R1 | Aldosterone-regulated sodium reabsorption |
| PRKCB | Aldosterone-regulated sodium reabsorption |
| SCNN1A | Aldosterone-regulated sodium reabsorption |
| SCNN1B | Aldosterone-regulated sodium reabsorption |
| SGK1 | Aldosterone-regulated sodium reabsorption |
| NEDD4L | Aldosterone-regulated sodium reabsorption |
| ATP1B4 | Aldosterone-regulated sodium reabsorption |
| G6PC | Galactose metabolism |
| GALE | Galactose metabolism |
| GALK2 | Galactose metabolism |
| GALT | Galactose metabolism |
| GCK | Galactose metabolism |
| HK1 | Galactose metabolism |
| HK2 | Galactose metabolism |
| LCT | Galactose metabolism |
| PFKL | Galactose metabolism |
| PFKM | Galactose metabolism |
| PFKP | Galactose metabolism |
| SI | Galactose metabolism |
| MGAM | Galactose metabolism |
| PGM2 | Galactose metabolism |
| HKDC1 | Galactose metabolism |
| CSTF2 | mRNA surveillance pathway |
| CSTF3 | mRNA surveillance pathway |
| MSI1 | mRNA surveillance pathway |
| NCBP1 | mRNA surveillance pathway |
| PNN | mRNA surveillance pathway |
| PPP2CB | mRNA surveillance pathway |
| PPP2R1B | mRNA surveillance pathway |
| PPP2R2A | mRNA surveillance pathway |
| PPP2R2C | mRNA surveillance pathway |
| PPP2R3A | mRNA surveillance pathway |
| UPF1 | mRNA surveillance pathway |
| SYMPK | mRNA surveillance pathway |
| PABPC4 | mRNA surveillance pathway |
| SMG7 | mRNA surveillance pathway |
| NXF1 | mRNA surveillance pathway |
| HBS1L | mRNA surveillance pathway |
| PAPOLA | mRNA surveillance pathway |
| CLP1 | mRNA surveillance pathway |
| CPSF6 | mRNA surveillance pathway |
| ACIN1 | mRNA surveillance pathway |
| SMG1 | mRNA surveillance pathway |
| SMG6 | mRNA surveillance pathway |
| SMG5 | mRNA surveillance pathway |
| UPF2 | mRNA surveillance pathway |
| PABPC1 | mRNA surveillance pathway |
| PCF11 | mRNA surveillance pathway |
| CPSF3 | mRNA surveillance pathway |
| PELO | mRNA surveillance pathway |
| CPSF2 | mRNA surveillance pathway |
| WDR33 | mRNA surveillance pathway |
| PPP2R2D | mRNA surveillance pathway |
| NXT2 | mRNA surveillance pathway |
| NXF3 | mRNA surveillance pathway |
| PAPOLB | mRNA surveillance pathway |
| PAPOLG | mRNA surveillance pathway |
| FIP1L1 | mRNA surveillance pathway |
| MSI2 | mRNA surveillance pathway |
| PABPN1L | mRNA surveillance pathway |
| ATP1A1 | Mineral absorption |
| ATP1A3 | Mineral absorption |
| ATP1A4 | Mineral absorption |
| ATP2B1 | Mineral absorption |
| CLCN2 | Mineral absorption |
| SLC26A3 | Mineral absorption |
| FTH1 | Mineral absorption |
| FTL | Mineral absorption |
| MT1E | Mineral absorption |
| SLC5A1 | Mineral absorption |
| SLC8A1 | Mineral absorption |
| SLC9A3 | Mineral absorption |
| TF | Mineral absorption |
| SLC30A1 | Mineral absorption |
| HEPH | Mineral absorption |
| ATP1B4 | Mineral absorption |
| TRPM7 | Mineral absorption |
| CYBRD1 | Mineral absorption |
| FTMT | Mineral absorption |
| TRPM6 | Mineral absorption |
| STEAP2 | Mineral absorption |
| SLC6A19 | Mineral absorption |
| FEN1 | Non-homologous end-joining |
| XRCC6 | Non-homologous end-joining |
| MRE11A | Non-homologous end-joining |
| PRKDC | Non-homologous end-joining |
| XRCC4 | Non-homologous end-joining |
| XRCC5 | Non-homologous end-joining |
| RAD50 | Non-homologous end-joining |
| POLM | Non-homologous end-joining |
| ABP1 | rginine and proline metabolism |
| ALDH2 | rginine and proline metabolism |
| ALDH3A2 | rginine and proline metabolism |
| AMD1 | rginine and proline metabolism |
| CKMT1B | rginine and proline metabolism |
| CPS1 | rginine and proline metabolism |
| DAO | rginine and proline metabolism |
| GAMT | rginine and proline metabolism |
| GLUL | rginine and proline metabolism |
| GOT1 | rginine and proline metabolism |
| MAOA | rginine and proline metabolism |
| MAOB | rginine and proline metabolism |
| NOS1 | rginine and proline metabolism |
| NOS2 | rginine and proline metabolism |
| NOS3 | rginine and proline metabolism |
| ODC1 | rginine and proline metabolism |
| P4HA1 | rginine and proline metabolism |
| PYCR1 | rginine and proline metabolism |
| ALDH18A1 | rginine and proline metabolism |
| SMS | rginine and proline metabolism |
| GLS2 | rginine and proline metabolism |
| LAP3 | rginine and proline metabolism |
| PRODH2 | rginine and proline metabolism |
| NAGS | rginine and proline metabolism |
| P4HA3 | rginine and proline metabolism |
| ACAA1 | Fatty acid metabolism |
| ACADL | Fatty acid metabolism |
| ACADVL | Fatty acid metabolism |
| ACAT1 | Fatty acid metabolism |
| ACOX1 | Fatty acid metabolism |
| ADH1A | Fatty acid metabolism |
| ADH1B | Fatty acid metabolism |
| ADH4 | Fatty acid metabolism |
| ALDH2 | Fatty acid metabolism |
| ALDH3A2 | Fatty acid metabolism |
| CPT1A | Fatty acid metabolism |
| CPT2 | Fatty acid metabolism |
| EHHADH | Fatty acid metabolism |
| ACSL1 | Fatty acid metabolism |
| HADHA | Fatty acid metabolism |
| HADHB | Fatty acid metabolism |
| ACOX3 | Fatty acid metabolism |
| ACAA2 | Fatty acid metabolism |
| ACSBG1 | Fatty acid metabolism |
| ACSL6 | Fatty acid metabolism |
| ACSBG2 | Fatty acid metabolism |
| CPT1C | Fatty acid metabolism |
| ANPEP | Hematopoietic cell lineage |
| CD1A | Hematopoietic cell lineage |
| CD1E | Hematopoietic cell lineage |
| CD2 | Hematopoietic cell lineage |
| CD5 | Hematopoietic cell lineage |
| CD22 | Hematopoietic cell lineage |
| CD33 | Hematopoietic cell lineage |
| CR1 | Hematopoietic cell lineage |
| CSF1 | Hematopoietic cell lineage |
| CSF1R | Hematopoietic cell lineage |
| CSF3R | Hematopoietic cell lineage |
| FLT3 | Hematopoietic cell lineage |
| IL1B | Hematopoietic cell lineage |
| IL1R1 | Hematopoietic cell lineage |
| IL4R | Hematopoietic cell lineage |
| IL7R | Hematopoietic cell lineage |
| IL11 | Hematopoietic cell lineage |
| ITGA6 | Hematopoietic cell lineage |
| ITGA1 | Hematopoietic cell lineage |
| ITGA2 | Hematopoietic cell lineage |
| ITGA3 | Hematopoietic cell lineage |
| ITGA4 | Hematopoietic cell lineage |
| ITGA5 | Hematopoietic cell lineage |
| ITGAM | Hematopoietic cell lineage |
| KIT | Hematopoietic cell lineage |
| KITLG | Hematopoietic cell lineage |
| TPO | Hematopoietic cell lineage |
| ATF4 | Protein processing in endoplasmic reticulum |
| CANX | Protein processing in endoplasmic reticulum |
| CAPN1 | Protein processing in endoplasmic reticulum |
| ATF6B | Protein processing in endoplasmic reticulum |
| ERN1 | Protein processing in endoplasmic reticulum |
| DNAJA1 | Protein processing in endoplasmic reticulum |
| HSPA5 | Protein processing in endoplasmic reticulum |
| HSPA8 | Protein processing in endoplasmic reticulum |
| HSP90AA1 | Protein processing in endoplasmic reticulum |
| HSP90AB1 | Protein processing in endoplasmic reticulum |
| ATXN3 | Protein processing in endoplasmic reticulum |
| NFE2L2 | Protein processing in endoplasmic reticulum |
| PRKCSH | Protein processing in endoplasmic reticulum |
| MAPK8 | Protein processing in endoplasmic reticulum |
| MAPK9 | Protein processing in endoplasmic reticulum |
| EIF2AK2 | Protein processing in endoplasmic reticulum |
| RPN1 | Protein processing in endoplasmic reticulum |
| SEC13 | Protein processing in endoplasmic reticulum |
| SEL1L | Protein processing in endoplasmic reticulum |
| SSR2 | Protein processing in endoplasmic reticulum |
| SEC62 | Protein processing in endoplasmic reticulum |
| UBE2E1 | Protein processing in endoplasmic reticulum |
| UBE2G1 | Protein processing in endoplasmic reticulum |
| VCP | Protein processing in endoplasmic reticulum |
| WFS1 | Protein processing in endoplasmic reticulum |
| TUSC3 | Protein processing in endoplasmic reticulum |
| CUL1 | Protein processing in endoplasmic reticulum |
| MBTPS1 | Protein processing in endoplasmic reticulum |
| SEC24C | Protein processing in endoplasmic reticulum |
| EDEM1 | Protein processing in endoplasmic reticulum |
| UBE4B | Protein processing in endoplasmic reticulum |
| 6-Mar | Protein processing in endoplasmic reticulum |
| SEC24B | Protein processing in endoplasmic reticulum |
| UBE2E3 | Protein processing in endoplasmic reticulum |
| SEC23B | Protein processing in endoplasmic reticulum |
| SEC23A | Protein processing in endoplasmic reticulum |
| SEC24A | Protein processing in endoplasmic reticulum |
| MAN1A2 | Protein processing in endoplasmic reticulum |
| MAN1B1 | Protein processing in endoplasmic reticulum |
| SEC31B | Protein processing in endoplasmic reticulum |
| EIF2AK1 | Protein processing in endoplasmic reticulum |
| SEC61A1 | Protein processing in endoplasmic reticulum |
| UBQLN2 | Protein processing in endoplasmic reticulum |
| ERO1L | Protein processing in endoplasmic reticulum |
| UBQLN3 | Protein processing in endoplasmic reticulum |
| SAR1B | Protein processing in endoplasmic reticulum |
| MBTPS2 | Protein processing in endoplasmic reticulum |
| DNAJC10 | Protein processing in endoplasmic reticulum |
| NPLOC4 | Protein processing in endoplasmic reticulum |
| NSFL1C | Protein processing in endoplasmic reticulum |
| ERO1LB | Protein processing in endoplasmic reticulum |
| SAR1A | Protein processing in endoplasmic reticulum |
| SIL1 | Protein processing in endoplasmic reticulum |
| UBXN6 | Protein processing in endoplasmic reticulum |
| DNAJC5B | Protein processing in endoplasmic reticulum |
| DERL3 | Protein processing in endoplasmic reticulum |
| ATXN3L | Protein processing in endoplasmic reticulum |
| EIF2AK4 | Protein processing in endoplasmic reticulum |
| CLNS1A | RNA transport |
| EIF4A1 | RNA transport |
| EIF4G1 | RNA transport |
| FMR1 | RNA transport |
| EIF3E | RNA transport |
| NCBP1 | RNA transport |
| NUP98 | RNA transport |
| PNN | RNA transport |
| RANBP2 | RNA transport |
| RANGAP1 | RNA transport |
| UPF1 | RNA transport |
| SEC13 | RNA transport |
| TPR | RNA transport |
| XPO1 | RNA transport |
| NUP214 | RNA transport |
| AAAS | RNA transport |
| FXR1 | RNA transport |
| EIF3A | RNA transport |
| EIF3D | RNA transport |
| EIF3H | RNA transport |
| EIF3J | RNA transport |
| EIF4G3 | RNA transport |
| PABPC4 | RNA transport |
| EIF2B3 | RNA transport |
| EIF4E2 | RNA transport |
| NUP155 | RNA transport |
| EIF5B | RNA transport |
| NUPL1 | RNA transport |
| POM121 | RNA transport |
| NUP153 | RNA transport |
| THOC1 | RNA transport |
| NXF1 | RNA transport |
| PAIP1 | RNA transport |
| POP1 | RNA transport |
| XPOT | RNA transport |
| ACIN1 | RNA transport |
| NUP205 | RNA transport |
| CYFIP1 | RNA transport |
| NUP160 | RNA transport |
| NUP188 | RNA transport |
| GEMIN5 | RNA transport |
| UPF2 | RNA transport |
| PABPC1 | RNA transport |
| CYFIP2 | RNA transport |
| TMEM48 | RNA transport |
| NXT2 | RNA transport |
| NXF3 | RNA transport |
| NUP107 | RNA transport |
| THOC2 | RNA transport |
| XPO5 | RNA transport |
| ELAC2 | RNA transport |
| SEH1L | RNA transport |
| NUP210L | RNA transport |
| POM121L2 | RNA transport |
| TGS1 | RNA transport |
| NUP43 | RNA transport |
| DHCR7 | Steroid biosynthesis |
| DHCR24 | Steroid biosynthesis |
| LIPA | Steroid biosynthesis |
| LSS | Steroid biosynthesis |
| SOAT1 | Steroid biosynthesis |
| TM7SF2 | Steroid biosynthesis |
| SOAT2 | Steroid biosynthesis |
| C5orf4 | Steroid biosynthesis |
| CYP2R1 | Steroid biosynthesis |
| CD40 | Toll-like receptor signaling pathway |
| CHUK | Toll-like receptor signaling pathway |
| MAP3K8 | Toll-like receptor signaling pathway |
| IFNA7 | Toll-like receptor signaling pathway |
| IFNA10 | Toll-like receptor signaling pathway |
| IFNAR1 | Toll-like receptor signaling pathway |
| IFNAR2 | Toll-like receptor signaling pathway |
| IL1B | Toll-like receptor signaling pathway |
| IRAK1 | Toll-like receptor signaling pathway |
| IRF3 | Toll-like receptor signaling pathway |
| LBP | Toll-like receptor signaling pathway |
| CXCL9 | Toll-like receptor signaling pathway |
| NFKB1 | Toll-like receptor signaling pathway |
| NFKBIA | Toll-like receptor signaling pathway |
| PIK3CA | Toll-like receptor signaling pathway |
| PIK3CD | Toll-like receptor signaling pathway |
| PIK3CG | Toll-like receptor signaling pathway |
| PIK3R1 | Toll-like receptor signaling pathway |
| MAPK8 | Toll-like receptor signaling pathway |
| MAPK9 | Toll-like receptor signaling pathway |
| MAP2K2 | Toll-like receptor signaling pathway |
| MAP2K3 | Toll-like receptor signaling pathway |
| CCL5 | Toll-like receptor signaling pathway |
| STAT1 | Toll-like receptor signaling pathway |
| MAP3K7 | Toll-like receptor signaling pathway |
| TLR1 | Toll-like receptor signaling pathway |
| TLR3 | Toll-like receptor signaling pathway |
| TLR4 | Toll-like receptor signaling pathway |
| TLR5 | Toll-like receptor signaling pathway |
| TRAF3 | Toll-like receptor signaling pathway |
| TRAF6 | Toll-like receptor signaling pathway |
| RIPK1 | Toll-like receptor signaling pathway |
| TAB1 | Toll-like receptor signaling pathway |
| TAB2 | Toll-like receptor signaling pathway |
| TBK1 | Toll-like receptor signaling pathway |
| TLR7 | Toll-like receptor signaling pathway |
| TLR9 | Toll-like receptor signaling pathway |
| TOLLIP | Toll-like receptor signaling pathway |
| TIRAP | Toll-like receptor signaling pathway |
| ABP1 | Tryptophan metabolism |
| ACAT1 | Tryptophan metabolism |
| ALDH2 | Tryptophan metabolism |
| ALDH3A2 | Tryptophan metabolism |
| AOX1 | Tryptophan metabolism |
| CCBL1 | Tryptophan metabolism |
| DDC | Tryptophan metabolism |
| EHHADH | Tryptophan metabolism |
| HADHA | Tryptophan metabolism |
| IDO1 | Tryptophan metabolism |
| MAOA | Tryptophan metabolism |
| MAOB | Tryptophan metabolism |
| TPH1 | Tryptophan metabolism |
| KYNU | Tryptophan metabolism |
| AADAT | Tryptophan metabolism |
| IDO2 | Tryptophan metabolism |
| ADH1A | Drug metabolism - cytochrome P450 |
| ADH1B | Drug metabolism - cytochrome P450 |
| ADH4 | Drug metabolism - cytochrome P450 |
| ALDH1A3 | Drug metabolism - cytochrome P450 |
| AOX1 | Drug metabolism - cytochrome P450 |
| CYP2A7 | Drug metabolism - cytochrome P450 |
| CYP2B6 | Drug metabolism - cytochrome P450 |
| CYP2C19 | Drug metabolism - cytochrome P450 |
| CYP2C8 | Drug metabolism - cytochrome P450 |
| CYP2C9 | Drug metabolism - cytochrome P450 |
| CYP2C18 | Drug metabolism - cytochrome P450 |
| CYP2E1 | Drug metabolism - cytochrome P450 |
| CYP3A5 | Drug metabolism - cytochrome P450 |
| FMO1 | Drug metabolism - cytochrome P450 |
| FMO4 | Drug metabolism - cytochrome P450 |
| GSTA1 | Drug metabolism - cytochrome P450 |
| GSTA4 | Drug metabolism - cytochrome P450 |
| GSTM4 | Drug metabolism - cytochrome P450 |
| GSTM5 | Drug metabolism - cytochrome P450 |
| MAOA | Drug metabolism - cytochrome P450 |
| MAOB | Drug metabolism - cytochrome P450 |
| UGT2B10 | Drug metabolism - cytochrome P450 |
| GSTO1 | Drug metabolism - cytochrome P450 |
| UGT1A6 | Drug metabolism - cytochrome P450 |
| UGT1A1 | Drug metabolism - cytochrome P450 |
| AP2A1 | Synaptic vesicle cycle |
| AP2B1 | Synaptic vesicle cycle |
| ATP6V1A | Synaptic vesicle cycle |
| ATP6V1B1 | Synaptic vesicle cycle |
| CACNA1A | Synaptic vesicle cycle |
| CACNA1B | Synaptic vesicle cycle |
| CLTC | Synaptic vesicle cycle |
| DNM1 | Synaptic vesicle cycle |
| NSF | Synaptic vesicle cycle |
| SLC18A1 | Synaptic vesicle cycle |
| SLC18A3 | Synaptic vesicle cycle |
| STXBP1 | Synaptic vesicle cycle |
| SYT1 | Synaptic vesicle cycle |
| ATP6V1F | Synaptic vesicle cycle |
| UNC13B | Synaptic vesicle cycle |
| RIMS1 | Synaptic vesicle cycle |
| UNC13A | Synaptic vesicle cycle |
| ATP6V0A2 | Synaptic vesicle cycle |
| ATP6V0A4 | Synaptic vesicle cycle |
| SLC17A7 | Synaptic vesicle cycle |
| SLC17A6 | Synaptic vesicle cycle |
| UNC13C | Synaptic vesicle cycle |
| ACAA1 | Peroxisome |
| ACOX1 | Peroxisome |
| AGXT | Peroxisome |
| CRAT | Peroxisome |
| DAO | Peroxisome |
| EHHADH | Peroxisome |
| EPHX2 | Peroxisome |
| ACSL1 | Peroxisome |
| HMGCL | Peroxisome |
| IDH1 | Peroxisome |
| MPV17 | Peroxisome |
| MVK | Peroxisome |
| NOS2 | Peroxisome |
| PEX12 | Peroxisome |
| PEX14 | Peroxisome |
| ABCD3 | Peroxisome |
| PXMP2 | Peroxisome |
| PEX2 | Peroxisome |
| PEX5 | Peroxisome |
| SCP2 | Peroxisome |
| SOD1 | Peroxisome |
| XDH | Peroxisome |
| ACOX3 | Peroxisome |
| AGPS | Peroxisome |
| PEX11B | Peroxisome |
| SLC25A17 | Peroxisome |
| PMVK | Peroxisome |
| SLC27A2 | Peroxisome |
| ACSL6 | Peroxisome |
| AMACR | Peroxisome |
| PIPOX | Peroxisome |
| CROT | Peroxisome |
| FAR2 | Peroxisome |
| PECR | Peroxisome |
| NUDT12 | Peroxisome |
| FAR1 | Peroxisome |
| PAOX | Peroxisome |
| ARNTL | Circadian rhythm |
| CRY2 | Circadian rhythm |
| CSNK1D | Circadian rhythm |
| CSNK1E | Circadian rhythm |
| NPAS2 | Circadian rhythm |
| RORA | Circadian rhythm |
| RORB | Circadian rhythm |
| CUL1 | Circadian rhythm |
| PER3 | Circadian rhythm |
| PER2 | Circadian rhythm |
| BTRC | Circadian rhythm |
| CLOCK | Circadian rhythm |
| PRKAG2 | Circadian rhythm |
| ADH1A | Glycolysis / Gluconeogenesis |
| ADH1B | Glycolysis / Gluconeogenesis |
| ADH4 | Glycolysis / Gluconeogenesis |
| ALDH2 | Glycolysis / Gluconeogenesis |
| ALDH1A3 | Glycolysis / Gluconeogenesis |
| ALDH3A2 | Glycolysis / Gluconeogenesis |
| ALDOA | Glycolysis / Gluconeogenesis |
| ALDOB | Glycolysis / Gluconeogenesis |
| FBP1 | Glycolysis / Gluconeogenesis |
| G6PC | Glycolysis / Gluconeogenesis |
| GCK | Glycolysis / Gluconeogenesis |
| GPI | Glycolysis / Gluconeogenesis |
| HK1 | Glycolysis / Gluconeogenesis |
| HK2 | Glycolysis / Gluconeogenesis |
| PCK1 | Glycolysis / Gluconeogenesis |
| PDHA2 | Glycolysis / Gluconeogenesis |
| PFKL | Glycolysis / Gluconeogenesis |
| PFKM | Glycolysis / Gluconeogenesis |
| PFKP | Glycolysis / Gluconeogenesis |
| PKLR | Glycolysis / Gluconeogenesis |
| PGM2 | Glycolysis / Gluconeogenesis |
| HKDC1 | Glycolysis / Gluconeogenesis |
| ADPGK | Glycolysis / Gluconeogenesis |
| ST6GAL1 | Other types of O-glycan biosynthesis |
| ST3GAL3 | Other types of O-glycan biosynthesis |
| OGT | Other types of O-glycan biosynthesis |
| PLOD3 | Other types of O-glycan biosynthesis |
| CHST10 | Other types of O-glycan biosynthesis |
| POMT1 | Other types of O-glycan biosynthesis |
| FUT9 | Other types of O-glycan biosynthesis |
| GLT25D2 | Other types of O-glycan biosynthesis |
| POFUT2 | Other types of O-glycan biosynthesis |
| POFUT1 | Other types of O-glycan biosynthesis |
| POMT2 | Other types of O-glycan biosynthesis |
| POMGNT1 | Other types of O-glycan biosynthesis |
| ST6GAL2 | Other types of O-glycan biosynthesis |
| B3GALTL | Other types of O-glycan biosynthesis |
| GXYLT1 | Other types of O-glycan biosynthesis |
| CSNK2B | Ribosome biogenesis in eukaryotes |
| NHP2L1 | Ribosome biogenesis in eukaryotes |
| XPO1 | Ribosome biogenesis in eukaryotes |
| NXF1 | Ribosome biogenesis in eukaryotes |
| UTP14A | Ribosome biogenesis in eukaryotes |
| WDR3 | Ribosome biogenesis in eukaryotes |
| POP1 | Ribosome biogenesis in eukaryotes |
| WDR43 | Ribosome biogenesis in eukaryotes |
| MDN1 | Ribosome biogenesis in eukaryotes |
| GTPBP4 | Ribosome biogenesis in eukaryotes |
| RRP7A | Ribosome biogenesis in eukaryotes |
| NOB1 | Ribosome biogenesis in eukaryotes |
| DROSHA | Ribosome biogenesis in eukaryotes |
| XRN1 | Ribosome biogenesis in eukaryotes |
| HEATR1 | Ribosome biogenesis in eukaryotes |
| RBM28 | Ribosome biogenesis in eukaryotes |
| NAT10 | Ribosome biogenesis in eukaryotes |
| RIOK2 | Ribosome biogenesis in eukaryotes |
| UTP6 | Ribosome biogenesis in eukaryotes |
| NXT2 | Ribosome biogenesis in eukaryotes |
| NXF3 | Ribosome biogenesis in eukaryotes |
| NOL6 | Ribosome biogenesis in eukaryotes |
| RIOK1 | Ribosome biogenesis in eukaryotes |
| WDR75 | Ribosome biogenesis in eukaryotes |
| WDR36 | Ribosome biogenesis in eukaryotes |
| SPATA5 | Ribosome biogenesis in eukaryotes |
| DNA2 | DNA replication |
| FEN1 | DNA replication |
| MCM2 | DNA replication |
| MCM3 | DNA replication |
| MCM4 | DNA replication |
| MCM6 | DNA replication |
| POLA1 | DNA replication |
| POLD1 | DNA replication |
| POLD2 | DNA replication |
| RFC1 | DNA replication |
| RFC2 | DNA replication |
| RFC3 | DNA replication |
| RFC4 | DNA replication |
| RPA1 | DNA replication |
| RPA4 | DNA replication |
| RNASEH2B | DNA replication |
| ACAT1 | Butanoate metabolism |
| EHHADH | Butanoate metabolism |
| HADHA | Butanoate metabolism |
| HMGCL | Butanoate metabolism |
| HMGCS1 | Butanoate metabolism |
| HMGCS2 | Butanoate metabolism |
| PDHA2 | Butanoate metabolism |
| ALDH5A1 | Butanoate metabolism |
| AACS | Butanoate metabolism |
| L2HGDH | Butanoate metabolism |
| ACSM4 | Butanoate metabolism |
| JAG1 | Notch signaling pathway |
| CREBBP | Notch signaling pathway |
| DTX1 | Notch signaling pathway |
| DVL3 | Notch signaling pathway |
| EP300 | Notch signaling pathway |
| HDAC2 | Notch signaling pathway |
| NOTCH2 | Notch signaling pathway |
| NOTCH3 | Notch signaling pathway |
| NOTCH4 | Notch signaling pathway |
| PSEN1 | Notch signaling pathway |
| CIR1 | Notch signaling pathway |
| NCOR2 | Notch signaling pathway |
| DLL3 | Notch signaling pathway |
| APH1A | Notch signaling pathway |
| MAML3 | Notch signaling pathway |
| PSENEN | Notch signaling pathway |
| MAML2 | Notch signaling pathway |
| DTX3L | Notch signaling pathway |
| PTCRA | Notch signaling pathway |
| ACAA1 | Valine, leucine and isoleucine degradation |
| ACAT1 | Valine, leucine and isoleucine degradation |
| ALDH2 | Valine, leucine and isoleucine degradation |
| ALDH3A2 | Valine, leucine and isoleucine degradation |
| AOX1 | Valine, leucine and isoleucine degradation |
| BCAT1 | Valine, leucine and isoleucine degradation |
| BCKDHA | Valine, leucine and isoleucine degradation |
| EHHADH | Valine, leucine and isoleucine degradation |
| HADHA | Valine, leucine and isoleucine degradation |
| HADHB | Valine, leucine and isoleucine degradation |
| HMGCL | Valine, leucine and isoleucine degradation |
| HMGCS1 | Valine, leucine and isoleucine degradation |
| HMGCS2 | Valine, leucine and isoleucine degradation |
| MUT | Valine, leucine and isoleucine degradation |
| PCCA | Valine, leucine and isoleucine degradation |
| PCCB | Valine, leucine and isoleucine degradation |
| ACAA2 | Valine, leucine and isoleucine degradation |
| ACAD8 | Valine, leucine and isoleucine degradation |
| MCCC1 | Valine, leucine and isoleucine degradation |
| GALNT1 | Mucin type O-Glycan biosynthesis |
| GCNT1 | Mucin type O-Glycan biosynthesis |
| B4GALT5 | Mucin type O-Glycan biosynthesis |
| GALNT6 | Mucin type O-Glycan biosynthesis |
| GALNT5 | Mucin type O-Glycan biosynthesis |
| GALNT8 | Mucin type O-Glycan biosynthesis |
| GCNT4 | Mucin type O-Glycan biosynthesis |
| GALNT7 | Mucin type O-Glycan biosynthesis |
| GALNTL1 | Mucin type O-Glycan biosynthesis |
| WBSCR17 | Mucin type O-Glycan biosynthesis |
| GALNT14 | Mucin type O-Glycan biosynthesis |
| GALNT13 | Mucin type O-Glycan biosynthesis |
| GALNTL2 | Mucin type O-Glycan biosynthesis |
| GALNTL5 | Mucin type O-Glycan biosynthesis |
| GALNTL6 | Mucin type O-Glycan biosynthesis |
| CHI3L1 | Amino sugar and nucleotide sugar metabolism |
| CHI3L2 | Amino sugar and nucleotide sugar metabolism |
| GALE | Amino sugar and nucleotide sugar metabolism |
| GALK2 | Amino sugar and nucleotide sugar metabolism |
| GALT | Amino sugar and nucleotide sugar metabolism |
| GCK | Amino sugar and nucleotide sugar metabolism |
| GFPT1 | Amino sugar and nucleotide sugar metabolism |
| GPI | Amino sugar and nucleotide sugar metabolism |
| HEXB | Amino sugar and nucleotide sugar metabolism |
| HK1 | Amino sugar and nucleotide sugar metabolism |
| HK2 | Amino sugar and nucleotide sugar metabolism |
| MPI | Amino sugar and nucleotide sugar metabolism |
| RENBP | Amino sugar and nucleotide sugar metabolism |
| FPGT | Amino sugar and nucleotide sugar metabolism |
| CHIA | Amino sugar and nucleotide sugar metabolism |
| PGM2 | Amino sugar and nucleotide sugar metabolism |
| CMAS | Amino sugar and nucleotide sugar metabolism |
| GNPNAT1 | Amino sugar and nucleotide sugar metabolism |
| UXS1 | Amino sugar and nucleotide sugar metabolism |
| HKDC1 | Amino sugar and nucleotide sugar metabolism |
| NPL | Amino sugar and nucleotide sugar metabolism |
| ACAA1 | Biosynthesis of unsaturated fatty acids |
| ACOX1 | Biosynthesis of unsaturated fatty acids |
| HADHA | Biosynthesis of unsaturated fatty acids |
| FADS1 | Biosynthesis of unsaturated fatty acids |
| SCD | Biosynthesis of unsaturated fatty acids |
| ACOX3 | Biosynthesis of unsaturated fatty acids |
| PTPLA | Biosynthesis of unsaturated fatty acids |
| FADS2 | Biosynthesis of unsaturated fatty acids |
| HSD17B12 | Biosynthesis of unsaturated fatty acids |
| PECR | Biosynthesis of unsaturated fatty acids |
| SCD5 | Biosynthesis of unsaturated fatty acids |
