# Supplementary material for: Integrated Analysis of Mutation Data from Various Sources Identifies Key Genes and Signaling Pathways in Hepatocellular Carcinoma
Source: PLoS One. 2014 Jul 2;9(7):e100854. doi: 10.1371/journal.pone.0100854 (PMC4079600; doi:10.1371/journal.pone.0100854)
Supplement: Table S5 — Mutated genes ranked by betweenness coefficient. (DOC) [file pone.0100854.s005.doc]

Supplementary Table S5. Mutated genes ranked by betweenness coefficient

| Gene symbol | No. of mutation samples | Betweenness coefficient |
| --- | --- | --- |
| GNAL | 2 | 59391.54 |
| NFKB1 | 1 | 50415.38 |
| PLCG1 | 2 | 47832.30 |
| TP53 | 56 | 42647.17 |
| MAPK8 | 2 | 33683.88 |
| MAPK9 | 1 | 33683.88 |
| PRKACA | 1 | 28222.10 |
| PRKACB | 2 | 28222.10 |
| PRKACG | 1 | 28222.10 |
| EGFR | 1 | 24095.89 |
| PTK2 | 2 | 23786.58 |
| ADCY3 | 1 | 23702.72 |
| ADCY8 | 2 | 22965.72 |
| RHOA | 1 | 21724.32 |
| GNAI2 | 1 | 21691.78 |
| ADCY9 | 3 | 17397.88 |
| ADCY2 | 6 | 17397.88 |
| TRAF6 | 2 | 17211.48 |
| SOCS3 | 1 | 16986.92 |
| CALML3 | 1 | 15833.63 |
| HRAS | 2 | 15266.47 |
| LEPR | 1 | 14511.25 |
| MDM2 | 2 | 14375.71 |
| PIK3CA | 3 | 13194.00 |
| PIK3CD | 1 | 13194.00 |
| PIK3CG | 5 | 13194.00 |
| PIK3R1 | 2 | 13194.00 |
| IRS4 | 2 | 12924.16 |
| THBS1 | 1 | 11372.42 |
| IGF1R | 9 | 11284.07 |
| RAF1 | 2 | 10784.47 |
| PPARA | 1 | 10706.62 |
| STAT1 | 1 | 10081.48 |
| C3 | 3 | 9933.37 |
| CREBBP | 2 | 9238.59 |
| EP300 | 3 | 9238.59 |
| CTNNB1 | 38 | 9207.55 |
| PLG | 3 | 8122.74 |
| PRKCB | 2 | 7747.57 |
| CBL | 3 | 7395.13 |
| CBLB | 2 | 7395.13 |
| CCL5 | 1 | 7086.98 |
| SERPINE1 | 1 | 6988.24 |
| VEGFB | 1 | 6849.98 |
| PLAU | 2 | 6812.24 |
| PPP3CB | 1 | 5872.67 |
| PPP3CC | 1 | 5872.67 |
| JAK2 | 3 | 5758.01 |
| GNAS | 2 | 5673.94 |
| RASGRF1 | 3 | 5585.55 |
| JAK1 | 8 | 5402.53 |
| FYN | 1 | 4717.03 |
| JAK3 | 3 | 4645.01 |
| ITGB2 | 1 | 3322.55 |
| GLI1 | 1 | 2942.33 |
| GLI2 | 1 | 2942.33 |
| GLI3 | 4 | 2942.33 |
| MTOR | 1 | 2900.80 |
| DVL3 | 3 | 2815.00 |
| CAMKK2 | 2 | 2762.63 |
| VCAM1 | 2 | 2738.00 |
| PTPN11 | 1 | 2634.38 |
| ADCY6 | 1 | 2618.12 |
| IL1B | 1 | 2587.24 |
| ROCK1 | 1 | 2573.81 |
| ROCK2 | 1 | 2573.81 |
| ARHGEF12 | 2 | 2565.94 |
| RASA1 | 1 | 2565.21 |
| CHUK | 1 | 2495.21 |
| PPARD | 1 | 2489.38 |
| PPP2CB | 2 | 2487.80 |
| PPP2R1B | 2 | 2487.80 |
| GRM1 | 2 | 2438.88 |
| GRM5 | 1 | 2438.88 |
| CXCL9 | 1 | 2285.06 |
| PRKAG2 | 2 | 2219.63 |
| C5 | 2 | 2192.00 |
| ITGA4 | 1 | 2062.83 |
| ITGA9 | 1 | 2062.83 |
| ITGB1 | 2 | 2062.83 |
| ITGAM | 2 | 2047.36 |
| SYK | 2 | 1852.73 |
| IL1R1 | 3 | 1815.62 |
| MET | 4 | 1798.80 |
| TCF7L2 | 1 | 1754.55 |
| LEF1 | 1 | 1754.55 |
| PRKCI | 1 | 1687.78 |
| FGR | 2 | 1681.88 |
| TRAF3 | 1 | 1671.00 |
| WNT2 | 1 | 1666.33 |
| WNT5A | 1 | 1666.33 |
| WNT10A | 1 | 1666.33 |
| FCER1G | 1 | 1656.73 |
| C6 | 1 | 1647.00 |
| ATF4 | 1 | 1619.70 |
| PDGFRA | 3 | 1564.98 |
| CCR1 | 1 | 1513.10 |
| CCR6 | 2 | 1513.10 |
| CCR7 | 1 | 1513.10 |
| CXCR1 | 2 | 1513.10 |
| CCR9 | 1 | 1513.10 |
| CCR2 | 2 | 1513.10 |
| ARHGDIA | 1 | 1495.21 |
| ARHGDIB | 1 | 1495.21 |
| NFKBIA | 1 | 1491.15 |
| C2 | 1 | 1488.00 |
| STAT5B | 1 | 1434.86 |
| SOCS4 | 2 | 1418.49 |
| ARHGEF6 | 2 | 1396.88 |
| SOCS5 | 1 | 1368.43 |
| MITF | 1 | 1322.74 |
| VAV1 | 1 | 1319.22 |
| VAV2 | 2 | 1319.22 |
| VAV3 | 8 | 1319.22 |
| SMAD2 | 1 | 1310.00 |
| SMAD3 | 2 | 1310.00 |
| HIF1A | 1 | 1291.04 |
| NGEF | 1 | 1230.17 |
| PTEN | 5 | 1212.36 |
| MAP3K14 | 2 | 1166.75 |
| ERBB4 | 7 | 1161.00 |
| KDR | 4 | 1156.96 |
| ACVR1 | 2 | 1131.00 |
| FZD2 | 1 | 1118.33 |
| FZD3 | 1 | 1118.33 |
| FZD1 | 1 | 1118.33 |
| TBK1 | 2 | 1109.45 |
| CDKN1A | 3 | 1105.00 |
| C7 | 1 | 1100.00 |
| MAP3K7 | 1 | 1034.99 |
| LCP2 | 3 | 1006.78 |
| IFNAR1 | 2 | 996.92 |
| IFNAR2 | 1 | 996.92 |
| STAT2 | 1 | 992.89 |
| ITGA6 | 2 | 973.49 |
| ITGA1 | 3 | 973.49 |
| ITGA2 | 2 | 973.49 |
| ITGA3 | 1 | 973.49 |
| ITGA5 | 1 | 973.49 |
| ITGAV | 3 | 973.49 |
| ITGB4 | 2 | 973.49 |
| ITGB5 | 1 | 973.49 |
| ITGB6 | 1 | 973.49 |
| ITGA10 | 1 | 973.49 |
| ITGA8 | 3 | 973.49 |
| ITGA11 | 1 | 973.49 |
| IRF3 | 3 | 933.45 |
| ARAF | 1 | 922.41 |
| BRAF | 1 | 922.41 |
| SOS1 | 1 | 835.34 |
| SOS2 | 2 | 835.34 |
| MAP3K3 | 1 | 821.20 |
| KIT | 1 | 757.19 |
| TLR4 | 2 | 746.00 |
| NEDD4L | 1 | 746.00 |
| PLXNB3 | 1 | 744.00 |
| PLXNB2 | 2 | 744.00 |
| KIR2DL4 | 1 | 744.00 |
| IRAK1 | 1 | 737.20 |
| SFN | 1 | 706.43 |
| ERBB3 | 1 | 609.40 |
| CCNA1 | 2 | 559.00 |
| C8B | 1 | 551.00 |
| STAT6 | 1 | 549.00 |
| DIAPH2 | 1 | 548.00 |
| FGFR2 | 2 | 537.22 |
| ITK | 1 | 526.54 |
| PPP2R2A | 1 | 506.69 |
| PPP2R2C | 1 | 506.69 |
| PPP2R2D | 2 | 506.69 |
| MAP3K1 | 1 | 488.95 |
| TXK | 1 | 455.30 |
| CSF1R | 2 | 445.40 |
| NTRK1 | 1 | 426.01 |
| ERBB2 | 1 | 410.61 |
| CDC27 | 1 | 407.87 |
| CDC16 | 1 | 407.87 |
| ANAPC2 | 1 | 407.87 |
| ANAPC7 | 1 | 407.87 |
| NOTCH2 | 4 | 379.33 |
| NOTCH3 | 3 | 379.33 |
| NOTCH4 | 1 | 379.33 |
| RPS6KA3 | 8 | 374.00 |
| SGK1 | 1 | 373.00 |
| TNFRSF11A | 1 | 373.00 |
| CARD9 | 1 | 372.01 |
| LTBR | 2 | 372.00 |
| DDX58 | 1 | 372.00 |
| IL6ST | 4 | 371.00 |
| IL21R | 3 | 371.00 |
| CYBA | 1 | 365.33 |
| NOX1 | 2 | 365.33 |
| NOX3 | 3 | 365.33 |
| MYLK | 4 | 354.71 |
| ARHGEF1 | 1 | 339.94 |
| CREB3L2 | 3 | 286.09 |
| GNGT1 | 1 | 277.22 |
| GNB2 | 1 | 267.22 |
| GNG5 | 1 | 267.22 |
| GNG11 | 1 | 267.22 |
| GNGT2 | 1 | 267.22 |
| GNB5 | 1 | 267.22 |
| IL2RB | 1 | 252.63 |
| PLCB2 | 1 | 197.10 |
| PLCB3 | 1 | 197.10 |
| PLCB4 | 6 | 197.10 |
| PLCB1 | 7 | 197.10 |
| INHBA | 1 | 190.00 |
| INHBC | 1 | 190.00 |
| NFATC4 | 2 | 162.18 |
| ELK1 | 1 | 155.89 |
| FLT1 | 2 | 154.23 |
| NLK | 1 | 150.66 |
| CCNB1 | 1 | 146.19 |
| LCK | 1 | 142.65 |
| C1QA | 1 | 124.33 |
| C1R | 1 | 124.33 |
| C1S | 3 | 124.33 |
| PARD3 | 2 | 98.66 |
| TAB1 | 1 | 75.23 |
| TAB2 | 1 | 75.23 |
| FCGR2A | 1 | 74.40 |
| GAB1 | 1 | 56.23 |
| TIRAP | 1 | 55.72 |
| CD40 | 1 | 48.29 |
| IL1RAP | 1 | 44.62 |
| ADRBK1 | 2 | 42.00 |
| BCAR1 | 1 | 38.00 |
| FLT4 | 1 | 33.17 |
| SLC9A1 | 1 | 32.33 |
| NFATC1 | 1 | 32.28 |
| NFATC3 | 1 | 32.28 |
| NFAT5 | 3 | 32.28 |
| GNAZ | 1 | 26.75 |
| MAD2L2 | 2 | 26.43 |
| ITGAX | 1 | 25.00 |
| ADRB2 | 2 | 23.42 |
| ARNTL | 1 | 20.00 |
| F2 | 1 | 19.50 |
| BUB1 | 1 | 19.43 |
| FANCD2 | 1 | 16.00 |
| PER3 | 2 | 9.07 |
| PER2 | 1 | 9.07 |
| BRCA2 | 5 | 9.00 |
| NPAS2 | 1 | 8.00 |
| CLOCK | 2 | 8.00 |
| CR1 | 2 | 5.46 |
| ELMO1 | 3 | 3.48 |
| EPHA2 | 1 | 3.28 |
| EPHA1 | 3 | 3.28 |
| EPHA3 | 5 | 3.28 |
| EPHA4 | 4 | 3.28 |
| EPHA5 | 7 | 3.28 |
| EPHA7 | 3 | 3.28 |
| EPHA6 | 4 | 3.28 |
| CRY2 | 1 | 3.07 |
| FANCL | 2 | 3.00 |
| OR1D2 | 1 | 2.39 |
| OR2C1 | 1 | 2.39 |
| OR3A1 | 1 | 2.39 |
| OR1A1 | 1 | 2.39 |
| OR1E1 | 1 | 2.39 |
| OR1E2 | 1 | 2.39 |
| OR5I1 | 1 | 2.39 |
| OR52A1 | 1 | 2.39 |
| OR2L2 | 1 | 2.39 |
| OR2K2 | 2 | 2.39 |
| OR5L2 | 1 | 2.39 |
| OR10J1 | 1 | 2.39 |
| OR8B8 | 1 | 2.39 |
| OR8G1 | 1 | 2.39 |
| OR10A3 | 2 | 2.39 |
| OR12D2 | 1 | 2.39 |
| OR10H3 | 2 | 2.39 |
| OR7C2 | 2 | 2.39 |
| OR7A5 | 1 | 2.39 |
| OR4E2 | 2 | 2.39 |
| OR2T1 | 1 | 2.39 |
| OR2J2 | 1 | 2.39 |
| OR4K5 | 1 | 2.39 |
| OR51G1 | 2 | 2.39 |
| OR51B2 | 1 | 2.39 |
| OR4K1 | 1 | 2.39 |
| OR5AC2 | 1 | 2.39 |
| OR51G2 | 1 | 2.39 |
| OR51E2 | 1 | 2.39 |
| OR4A5 | 1 | 2.39 |
| OR4A16 | 1 | 2.39 |
| OR4A15 | 1 | 2.39 |
| OR6N2 | 2 | 2.39 |
| OR6K2 | 1 | 2.39 |
| OR2G3 | 1 | 2.39 |
| OR2G2 | 1 | 2.39 |
| OR2C3 | 1 | 2.39 |
| OR12D3 | 3 | 2.39 |
| OR52J3 | 1 | 2.39 |
| OR51A7 | 1 | 2.39 |
| OR52R1 | 1 | 2.39 |
| OR56A4 | 1 | 2.39 |
| OR10A7 | 1 | 2.39 |
| OR11H6 | 1 | 2.39 |
| OR7G1 | 1 | 2.39 |
| OR10H4 | 1 | 2.39 |
| OR2M5 | 3 | 2.39 |
| OR2M3 | 1 | 2.39 |
| OR2T12 | 1 | 2.39 |
| OR14C36 | 2 | 2.39 |
| OR2T4 | 3 | 2.39 |
| OR2B11 | 1 | 2.39 |
| OR10Z1 | 4 | 2.39 |
| OR2Y1 | 1 | 2.39 |
| OR2A14 | 1 | 2.39 |
| OR6B1 | 1 | 2.39 |
| OR2F2 | 1 | 2.39 |
| OR13C3 | 1 | 2.39 |
| OR1L8 | 1 | 2.39 |
| OR52B4 | 1 | 2.39 |
| OR52I2 | 1 | 2.39 |
| OR51E1 | 1 | 2.39 |
| OR6B3 | 1 | 2.39 |
| OR1Q1 | 1 | 2.39 |
| OR4C16 | 2 | 2.39 |
| OR5L1 | 2 | 2.39 |
| OR5AS1 | 1 | 2.39 |
| OR8K5 | 3 | 2.39 |
| OR5T2 | 1 | 2.39 |
| OR8H1 | 1 | 2.39 |
| OR8K3 | 3 | 2.39 |
| OR5R1 | 2 | 2.39 |
| OR5M3 | 1 | 2.39 |
| OR5M8 | 1 | 2.39 |
| OR5AR1 | 1 | 2.39 |
| OR8B12 | 2 | 2.39 |
| OR8G5 | 1 | 2.39 |
| OR10G8 | 1 | 2.39 |
| OR9I1 | 2 | 2.39 |
| OR9Q1 | 1 | 2.39 |
| OR1S1 | 1 | 2.39 |
| OR5B17 | 1 | 2.39 |
| OR5A2 | 1 | 2.39 |
| OR4D11 | 1 | 2.39 |
| OR6C74 | 1 | 2.39 |
| OR6C3 | 1 | 2.39 |
| OR51B5 | 1 | 2.39 |
| OR10AG1 | 1 | 2.39 |
| OR5J2 | 1 | 2.39 |
| OR4C12 | 4 | 2.39 |
| OR8D2 | 1 | 2.39 |
| OR9G4 | 3 | 2.39 |
| OR10A4 | 2 | 2.39 |
| OR2Z1 | 1 | 2.39 |
| OR10H5 | 1 | 2.39 |
| OR14A16 | 2 | 2.39 |
| OR8D4 | 1 | 2.39 |
| OR5F1 | 2 | 2.39 |
| OR5AP2 | 3 | 2.39 |
| OR10A2 | 1 | 2.39 |
| OR6C2 | 1 | 2.39 |
| OR8S1 | 1 | 2.39 |
| OR10R2 | 2 | 2.39 |
| OR6V1 | 1 | 2.39 |
| OR2A12 | 2 | 2.39 |
| OR1B1 | 1 | 2.39 |
| OR52K1 | 1 | 2.39 |
| OR52I1 | 1 | 2.39 |
| OR51D1 | 1 | 2.39 |
| OR51B6 | 1 | 2.39 |
| OR51Q1 | 1 | 2.39 |
| OR52N4 | 2 | 2.39 |
| OR52N5 | 1 | 2.39 |
| OR52N2 | 2 | 2.39 |
| OR52E6 | 1 | 2.39 |
| OR52E4 | 1 | 2.39 |
| OR56A3 | 1 | 2.39 |
| OR4X1 | 1 | 2.39 |
| OR5D13 | 2 | 2.39 |
| OR8H2 | 1 | 2.39 |
| OR5T1 | 1 | 2.39 |
| OR8K1 | 1 | 2.39 |
| OR5M9 | 1 | 2.39 |
| OR5M10 | 1 | 2.39 |
| OR5M1 | 1 | 2.39 |
| OR5B12 | 2 | 2.39 |
| OR10V1 | 1 | 2.39 |
| OR6M1 | 1 | 2.39 |
| OR10G7 | 1 | 2.39 |
| OR6C1 | 1 | 2.39 |
| OR6C75 | 2 | 2.39 |
| OR6C76 | 1 | 2.39 |
| OR6C70 | 1 | 2.39 |
| OR4N2 | 1 | 2.39 |
| OR4N5 | 1 | 2.39 |
| OR4M2 | 1 | 2.39 |
| OR4F6 | 1 | 2.39 |
| OR7G3 | 1 | 2.39 |
| OR10K2 | 1 | 2.39 |
| OR10K1 | 4 | 2.39 |
| OR6Y1 | 1 | 2.39 |
| OR2AK2 | 1 | 2.39 |
| OR2L3 | 1 | 2.39 |
| OR2G6 | 1 | 2.39 |
| OR13J1 | 1 | 2.39 |
| OR2A5 | 1 | 2.39 |
| OR51A4 | 2 | 2.39 |
| OR2T27 | 1 | 2.39 |
| OR4A47 | 1 | 2.39 |
| OR4C45 | 2 | 2.39 |
| OR6C65 | 1 | 2.39 |
| OR5B3 | 1 | 2.39 |
| OR4Q3 | 2 | 2.39 |
| OR10J3 | 1 | 2.39 |
| OR2J3 | 1 | 2.39 |
| OR10C1 | 2 | 2.39 |
| FZR1 | 2 | 2.29 |
| CHEK1 | 1 | 2.00 |
| PROS1 | 2 | 2.00 |
| FANCG | 1 | 1.50 |
| FANCM | 3 | 1.50 |
| PPP1R12A | 1 | 1.00 |
| PHKA1 | 2 | 0.50 |
| PHKB | 1 | 0.50 |
| PHKG1 | 1 | 0.50 |
| RORA | 2 | 0.40 |
| RORB | 2 | 0.40 |
| ATP1A1 | 1 | 0.25 |
| ATP1A3 | 1 | 0.25 |
| ATP1A4 | 1 | 0.25 |
| ATP1B4 | 1 | 0.25 |
| BUB1B | 2 | 0 |
| CSNK1G3 | 2 | 0 |
| PTCH1 | 1 | 0 |
| PTTG2 | 1 | 0 |
| CSNK1G1 | 1 | 0 |
| HHIP | 4 | 0 |
| SLC12A2 | 1 | 0 |
| CCNB3 | 2 | 0 |
| ATM | 5 | 0 |
| BMP6 | 1 | 0 |
| BMP8B | 1 | 0 |
| F12 | 1 | 0 |
| ITGAD | 3 | 0 |
| ITGAE | 3 | 0 |
| KLKB1 | 2 | 0 |
| YES1 | 2 | 0 |
| RIPK1 | 1 | 0 |
| ACVR2A | 8 | 0 |
| NODAL | 1 | 0 |
| PIP5K1C | 1 | 0 |
| RASGRP3 | 2 | 0 |
| CSNK1E | 1 | 0 |
| ATF2 | 1 | 0 |
| SFRP1 | 2 | 0 |
| CREB5 | 1 | 0 |
| CNTFR | 1 | 0 |
| CSF2RB | 2 | 0 |
| CSF3R | 1 | 0 |
| IL4R | 1 | 0 |
| IL7R | 3 | 0 |
| IL12RB1 | 1 | 0 |
| IL12RB2 | 2 | 0 |
| IL13RA2 | 2 | 0 |
| LIFR | 4 | 0 |
| OSMR | 2 | 0 |
| IL20RB | 1 | 0 |
| IL23R | 1 | 0 |
| CSNK1D | 2 | 0 |
| NOS2 | 1 | 0 |
| A2M | 1 | 0 |
| MAP3K8 | 1 | 0 |
| DOCK1 | 2 | 0 |
| MYL12A | 1 | 0 |
| PRKCQ | 2 | 0 |
| BTRC | 1 | 0 |
| PLA2G4A | 1 | 0 |
| PLA2G6 | 1 | 0 |
| PLA2G3 | 1 | 0 |
| EFNA5 | 2 | 0 |
| CUL1 | 3 | 0 |
| JAM2 | 1 | 0 |
| ATR | 7 | 0 |
| PRKCH | 2 | 0 |
| SHC4 | 1 | 0 |
| CTNND1 | 4 | 0 |
| PAK1 | 1 | 0 |
| MAP2K2 | 1 | 0 |
| ACAA1 | 1 | 0 |
| ACACA | 1 | 0 |
| ACACB | 2 | 0 |
| ACADL | 1 | 0 |
| ACOX1 | 1 | 0 |
| ADORA2A | 1 | 0 |
| JAG1 | 1 | 0 |
| AMPH | 7 | 0 |
| BIRC2 | 1 | 0 |
| APOA1 | 1 | 0 |
| AR | 1 | 0 |
| ARHGAP5 | 3 | 0 |
| ARRB2 | 1 | 0 |
| SERPINC1 | 2 | 0 |
| TNFRSF17 | 1 | 0 |
| BRCA1 | 2 | 0 |
| SERPING1 | 1 | 0 |
| C9 | 2 | 0 |
| CACNA1A | 1 | 0 |
| CACNA1B | 1 | 0 |
| CACNA1C | 6 | 0 |
| CACNA1D | 2 | 0 |
| CACNA1S | 3 | 0 |
| CACNA2D1 | 1 | 0 |
| CACNB1 | 1 | 0 |
| CACNB2 | 1 | 0 |
| CACNB4 | 1 | 0 |
| CCNG1 | 3 | 0 |
| CCNG2 | 1 | 0 |
| CDC6 | 1 | 0 |
| CDC25C | 1 | 0 |
| CFTR | 3 | 0 |
| CHAD | 1 | 0 |
| CHRM1 | 2 | 0 |
| CHRM2 | 1 | 0 |
| CHRM3 | 1 | 0 |
| CHRNA4 | 1 | 0 |
| CHRNB4 | 2 | 0 |
| CNGB1 | 1 | 0 |
| CNTN1 | 5 | 0 |
| COL1A1 | 3 | 0 |
| COL1A2 | 2 | 0 |
| COL2A1 | 2 | 0 |
| COL3A1 | 1 | 0 |
| COL4A1 | 2 | 0 |
| COL4A2 | 1 | 0 |
| COL4A4 | 2 | 0 |
| COL4A5 | 3 | 0 |
| COL4A6 | 1 | 0 |
| COL5A1 | 5 | 0 |
| COL5A2 | 1 | 0 |
| COL6A1 | 1 | 0 |
| COL6A2 | 1 | 0 |
| COL6A3 | 2 | 0 |
| COL11A1 | 11 | 0 |
| COL11A2 | 1 | 0 |
| CPT1A | 1 | 0 |
| CPT2 | 1 | 0 |
| ATF6B | 1 | 0 |
| CSF1 | 1 | 0 |
| CSNK2B | 1 | 0 |
| CYP8B1 | 1 | 0 |
| DAG1 | 1 | 0 |
| DCC | 7 | 0 |
| DCT | 1 | 0 |
| DNM1 | 1 | 0 |
| DOCK2 | 6 | 0 |
| DRD3 | 1 | 0 |
| DTX1 | 1 | 0 |
| DUSP1 | 1 | 0 |
| DUSP4 | 2 | 0 |
| LPAR1 | 2 | 0 |
| EFNB2 | 1 | 0 |
| EHHADH | 1 | 0 |
| CTTN | 1 | 0 |
| EPHB3 | 1 | 0 |
| EPHB4 | 1 | 0 |
| EPHB6 | 1 | 0 |
| ERN1 | 1 | 0 |
| ETS1 | 1 | 0 |
| MECOM | 4 | 0 |
| F2R | 2 | 0 |
| F5 | 3 | 0 |
| F8 | 2 | 0 |
| F13B | 2 | 0 |
| FANCC | 1 | 0 |
| FANCE | 1 | 0 |
| ACSL1 | 1 | 0 |
| PTK2B | 2 | 0 |
| FANCB | 1 | 0 |
| FASN | 1 | 0 |
| FGA | 5 | 0 |
| FGB | 1 | 0 |
| FGF5 | 1 | 0 |
| FGF12 | 1 | 0 |
| FGG | 1 | 0 |
| FLT3 | 5 | 0 |
| FN1 | 1 | 0 |
| G6PC | 3 | 0 |
| GABBR1 | 1 | 0 |
| GCK | 1 | 0 |
| GJA1 | 1 | 0 |
| GK | 1 | 0 |
| GNRHR | 1 | 0 |
| MKNK2 | 1 | 0 |
| GRIA1 | 3 | 0 |
| GRIA2 | 2 | 0 |
| GRIA4 | 6 | 0 |
| GRIN2A | 3 | 0 |
| GRIN2B | 1 | 0 |
| ARHGAP35 | 3 | 0 |
| GRM2 | 1 | 0 |
| GRM3 | 4 | 0 |
| GRM4 | 4 | 0 |
| GRM7 | 1 | 0 |
| GRM8 | 2 | 0 |
| CXCL2 | 1 | 0 |
| GUCA1A | 1 | 0 |
| GUCA1B | 1 | 0 |
| GUCY2F | 2 | 0 |
| GYS2 | 3 | 0 |
| GUCY2D | 1 | 0 |
| HDAC2 | 1 | 0 |
| CFH | 2 | 0 |
| HGF | 4 | 0 |
| NRG1 | 1 | 0 |
| HK1 | 1 | 0 |
| HK2 | 2 | 0 |
| HLA-A | 2 | 0 |
| HLA-E | 1 | 0 |
| HMGCS2 | 1 | 0 |
| HRH2 | 1 | 0 |
| HSPA8 | 2 | 0 |
| HSPA9 | 3 | 0 |
| HSP90AA1 | 1 | 0 |
| HSP90AB1 | 1 | 0 |
| HSPD1 | 1 | 0 |
| HSPG2 | 3 | 0 |
| HTR1A | 1 | 0 |
| HTR2A | 1 | 0 |
| HTR2B | 2 | 0 |
| HTR2C | 1 | 0 |
| HTR7 | 1 | 0 |
| TNC | 3 | 0 |
| IBSP | 1 | 0 |
| IFNA7 | 1 | 0 |
| IFNA10 | 1 | 0 |
| IFNG | 1 | 0 |
| IFNW1 | 1 | 0 |
| IGFBP3 | 1 | 0 |
| IL11 | 1 | 0 |
| IDO1 | 1 | 0 |
| ITPKB | 3 | 0 |
| ITPR1 | 3 | 0 |
| ITPR2 | 4 | 0 |
| ITPR3 | 4 | 0 |
| CD82 | 1 | 0 |
| KCNB1 | 2 | 0 |
| KCNJ5 | 2 | 0 |
| KCNJ6 | 1 | 0 |
| KCNJ9 | 1 | 0 |
| KCNMA1 | 1 | 0 |
| KIR2DS4 | 1 | 0 |
| KLRC2 | 2 | 0 |
| LAMA2 | 11 | 0 |
| LAMA3 | 4 | 0 |
| LAMA4 | 2 | 0 |
| LAMA5 | 1 | 0 |
| LAMB1 | 3 | 0 |
| LAMB3 | 2 | 0 |
| LAMC1 | 2 | 0 |
| LAMC2 | 3 | 0 |
| LIMK2 | 2 | 0 |
| LIPE | 2 | 0 |
| LLGL2 | 1 | 0 |
| LLGL1 | 1 | 0 |
| LPL | 1 | 0 |
| LRP6 | 1 | 0 |
| LTBP1 | 4 | 0 |
| MCM2 | 1 | 0 |
| MCM3 | 1 | 0 |
| MCM4 | 2 | 0 |
| MCM6 | 2 | 0 |
| CD46 | 1 | 0 |
| ME1 | 2 | 0 |
| MAP3K4 | 3 | 0 |
| KITLG | 1 | 0 |
| MLH1 | 1 | 0 |
| MLLT4 | 3 | 0 |
| NR3C2 | 3 | 0 |
| MMP1 | 1 | 0 |
| MMP2 | 1 | 0 |
| MMP9 | 1 | 0 |
| MYH9 | 2 | 0 |
| MYH10 | 3 | 0 |
| NCAM1 | 2 | 0 |
| NCAM2 | 4 | 0 |
| NEDD4 | 1 | 0 |
| NF1 | 3 | 0 |
| NFE2L2 | 7 | 0 |
| NGFR | 1 | 0 |
| NOS1 | 3 | 0 |
| NOS3 | 2 | 0 |
| NRCAM | 3 | 0 |
| NTRK2 | 3 | 0 |
| NTRK3 | 1 | 0 |
| PAK2 | 1 | 0 |
| PAK3 | 1 | 0 |
| PCK1 | 1 | 0 |
| PDC | 1 | 0 |
| PDE1A | 2 | 0 |
| PDE1C | 1 | 0 |
| PDE1B | 1 | 0 |
| PDE6B | 1 | 0 |
| PF4V1 | 1 | 0 |
| PFN2 | 1 | 0 |
| PGR | 3 | 0 |
| SERPINA1 | 1 | 0 |
| PKLR | 1 | 0 |
| PLTP | 1 | 0 |
| PLXNA1 | 1 | 0 |
| PLXNA2 | 2 | 0 |
| PPARG | 1 | 0 |
| PPM1A | 2 | 0 |
| PPP1R3A | 3 | 0 |
| PPP1R3C | 1 | 0 |
| PRKAR1A | 1 | 0 |
| PRKAR1B | 1 | 0 |
| PRKAR2A | 1 | 0 |
| PKN2 | 3 | 0 |
| PRKDC | 3 | 0 |
| PRKG1 | 1 | 0 |
| PRKG2 | 4 | 0 |
| MAPK7 | 1 | 0 |
| MAP2K3 | 1 | 0 |
| EIF2AK2 | 3 | 0 |
| PRSS1 | 2 | 0 |
| RELN | 9 | 0 |
| PSEN1 | 1 | 0 |
| PTGIR | 1 | 0 |
| PTGS2 | 1 | 0 |
| PTH1R | 1 | 0 |
| PTPN1 | 1 | 0 |
| PTPRB | 5 | 0 |
| PTPRC | 2 | 0 |
| PTPRF | 2 | 0 |
| PTPRJ | 3 | 0 |
| PTPRM | 3 | 0 |
| PVRL1 | 2 | 0 |
| PYGL | 2 | 0 |
| RAD51 | 1 | 0 |
| RASA2 | 1 | 0 |
| RASGRF2 | 2 | 0 |
| RB1 | 6 | 0 |
| RBL1 | 2 | 0 |
| RBL2 | 2 | 0 |
| RET | 1 | 0 |
| ROBO1 | 1 | 0 |
| ROBO2 | 4 | 0 |
| RPS6KB1 | 1 | 0 |
| RPS6KB2 | 1 | 0 |
| RXRB | 1 | 0 |
| RXRG | 3 | 0 |
| RYR2 | 8 | 0 |
| SCD | 1 | 0 |
| SCN1A | 3 | 0 |
| SCNN1A | 2 | 0 |
| SCNN1B | 2 | 0 |
| SCP2 | 1 | 0 |
| CCL11 | 1 | 0 |
| CCL23 | 1 | 0 |
| CXCL12 | 2 | 0 |
| SLC2A1 | 1 | 0 |
| SLC2A2 | 1 | 0 |
| SLC8A1 | 4 | 0 |
| SLIT1 | 1 | 0 |
| SP1 | 1 | 0 |
| SREBF1 | 2 | 0 |
| TGFBR1 | 2 | 0 |
| THBS2 | 3 | 0 |
| THBS3 | 2 | 0 |
| THBS4 | 2 | 0 |
| TJP1 | 2 | 0 |
| TLR1 | 1 | 0 |
| TLR3 | 1 | 0 |
| TNFAIP3 | 1 | 0 |
| TNR | 5 | 0 |
| TNXB | 1 | 0 |
| TRPC1 | 1 | 0 |
| TSC2 | 4 | 0 |
| TYR | 1 | 0 |
| TYRP1 | 2 | 0 |
| USP1 | 1 | 0 |
| VCP | 4 | 0 |
| EZR | 1 | 0 |
| VWF | 3 | 0 |
| TRIM25 | 2 | 0 |
| MAPKAPK3 | 1 | 0 |
| STAM | 1 | 0 |
| ACOX3 | 1 | 0 |
| CDC7 | 3 | 0 |
| PIP5K1A | 1 | 0 |
| PIP4K2B | 1 | 0 |
| MKNK1 | 2 | 0 |
| TNFSF11 | 1 | 0 |
| CHRD | 1 | 0 |
| TNFSF14 | 1 | 0 |
| TNFSF13 | 1 | 0 |
| TNFRSF10B | 2 | 0 |
| FGF17 | 1 | 0 |
| IQGAP1 | 1 | 0 |
| CHRNA6 | 1 | 0 |
| SH2D2A | 1 | 0 |
| MAP3K6 | 1 | 0 |
| RPS6KA5 | 2 | 0 |
| CACNA2D2 | 2 | 0 |
| CER1 | 1 | 0 |
| SLIT2 | 3 | 0 |
| NRXN3 | 4 | 0 |
| NRXN1 | 5 | 0 |
| NRXN2 | 1 | 0 |
| FADS2 | 1 | 0 |
| MAP4K4 | 1 | 0 |
| EIF4E2 | 1 | 0 |
| TP53I3 | 1 | 0 |
| GABBR2 | 1 | 0 |
| GUCA1C | 2 | 0 |
| RAPGEF2 | 4 | 0 |
| ULK2 | 1 | 0 |
| MAGI2 | 1 | 0 |
| NR1H3 | 1 | 0 |
| KCNMB2 | 1 | 0 |
| STAM2 | 2 | 0 |
| PAK4 | 1 | 0 |
| LAMC3 | 2 | 0 |
| NOD1 | 1 | 0 |
| RAPGEF3 | 1 | 0 |
| BAIAP2 | 1 | 0 |
| FST | 2 | 0 |
| SEMA4D | 1 | 0 |
| SEMA4B | 3 | 0 |
| DLL3 | 1 | 0 |
| NRG3 | 1 | 0 |
| WWP1 | 6 | 0 |
| RAPGEF4 | 1 | 0 |
| NLGN1 | 3 | 0 |
| DKK1 | 1 | 0 |
| CYFIP1 | 2 | 0 |
| ACSBG1 | 1 | 0 |
| ACSL6 | 1 | 0 |
| TNFRSF13B | 1 | 0 |
| CYFIP2 | 1 | 0 |
| EIF2AK1 | 1 | 0 |
| DKK4 | 1 | 0 |
| BLNK | 1 | 0 |
| COL5A3 | 5 | 0 |
| ADIPOR1 | 1 | 0 |
| APH1A | 1 | 0 |
| ANGPTL4 | 1 | 0 |
| SEMA4C | 1 | 0 |
| PLXNA3 | 2 | 0 |
| CACNA2D3 | 5 | 0 |
| PSENEN | 1 | 0 |
| NSFL1C | 2 | 0 |
| TRPV5 | 4 | 0 |
| SPHK2 | 3 | 0 |
| PAK6 | 1 | 0 |
| DUSP22 | 1 | 0 |
| PAK7 | 1 | 0 |
| GJD2 | 1 | 0 |
| CHD8 | 2 | 0 |
| SEMA4G | 2 | 0 |
| NLRC4 | 2 | 0 |
| IL21 | 1 | 0 |
| CACNG7 | 1 | 0 |
| TNN | 3 | 0 |
| PERP | 1 | 0 |
| ROBO3 | 2 | 0 |
| CLEC7A | 3 | 0 |
| TBL1XR1 | 3 | 0 |
| MYH14 | 4 | 0 |
| SCD5 | 1 | 0 |
| HKDC1 | 1 | 0 |
| PDGFD | 1 | 0 |
| CXXC4 | 1 | 0 |
| UBXN6 | 1 | 0 |
| DUSP16 | 1 | 0 |
| TAS1R2 | 2 | 0 |
| ACSBG2 | 2 | 0 |
| DIAPH3 | 1 | 0 |
| BRIP1 | 2 | 0 |
| PPP1R1B | 1 | 0 |
| CARD11 | 2 | 0 |
| PTPN5 | 1 | 0 |
| CACNA2D4 | 6 | 0 |
| NLRP3 | 4 | 0 |
| CPT1C | 2 | 0 |
| ACVR1C | 1 | 0 |
| COL6A6 | 2 | 0 |
| DTX3L | 1 | 0 |
| KCNU1 | 4 | 0 |
| SPRED1 | 1 | 0 |
| IDO2 | 1 | 0 |
| PIKFYVE | 3 | 0 |
| SPRED2 | 2 | 0 |
| LAMA1 | 6 | 0 |
| FBXO43 | 2 | 0 |
| AGRN | 1 | 0 |
| SPDYC | 1 | 0 |
| EIF2AK4 | 1 | 0 |
| OCLN | 2 | 0 |
