# Supplementary material for: Integrated Analysis of Mutation Data from Various Sources Identifies Key Genes and Signaling Pathways in Hepatocellular Carcinoma
Source: PLoS One. 2014 Jul 2;9(7):e100854. doi: 10.1371/journal.pone.0100854 (PMC4079600; doi:10.1371/journal.pone.0100854)
Supplement: Table S6 — Clinicopathological features of 207 HCC patients. (DOC) [file pone.0100854.s006.doc]

**Supplementary Table S6. Clinicopathological features of 207 HCC patients.**

|  | 207 HCC Patients |
| --- | --- |
| **Gender** (n^*^=207) |  |
| Male | 158 |
| Female | 49 |
| **Age** (n=119) |  |
| <60 | 37 |
| ≥60 | 82 |
| **Grade** (n=182) |  |
| Edmonson I-II | 134 |
| Edmonson III-IV | 48 |
| **Stage** (n=99) |  |
| Metastasis | 25 |
| non-metastasis | 74 |
| **HBsAg** (n=98) |  |
| Positive | 89 |
| Negative | 9 |
| **Liver pathology** (n=61) |  |
| cirrhotic  non-cirrhotic | 55  6 |
| **OS time (Months)** (n=20) |  |
| Range | 1-60 |
| Median | 37 |

* n is the number of HCC patients with available clinical information.
